# Catalytic discrimination between formyl groups in regio- and stereoselective intramolecular cross-aldol reactions†
†Electronic supplementary information (ESI) available: Experimental procedures, characterization data and DFT studies. See DOI: 10.1039/c5sc04594k


**DOI:** 10.1039/c5sc04594k

**Published:** 2016-02-22

**Authors:** Tomonori Baba, Junya Yamamoto, Kazuhiro Hayashi, Makoto Sato, Masahiro Yamanaka, Takeo Kawabata, Takumi Furuta

**Affiliations:** a Institute for Chemical Research , Kyoto University , Uji , Kyoto 611-0011 , Japan . Email: furuta@fos.kuicr.kyoto-u.ac.jp; b Department of Chemistry and Research Center for Smart Molecules , Faculty of Science , Rikkyo University , 3-34-1 Nish-Ikebukuro, Toshima-ku , Tokyo 171-8501 , Japan

## Abstract

Catalytic discrimination between inequivalent formyl groups was achieved using an aniline-type acid–base catalyst for the regio-, diastereo-, and enantioselective intramolecular cross-aldol reactions of enolizable dials. Although l-proline gave a mixture of the regio- and stereoisomeric products in the presence of an *N*-containing 1,6-dial, the aniline-type catalyst afforded *anti*-3,4-disubstituted pyrrolidine in high regio-, and stereoselectivity beyond the background reaction, which led to the regioisomeric 2,3-disubstituted products. The mild reactivity of the aniline-type amine facilitated catalytic discrimination between the inequivalent formyl groups. Kinetic isotope effect studies and reductive amination experiments suggested that the regioselectivity was controlled under the enamine-forming steps.

## Abstract 2

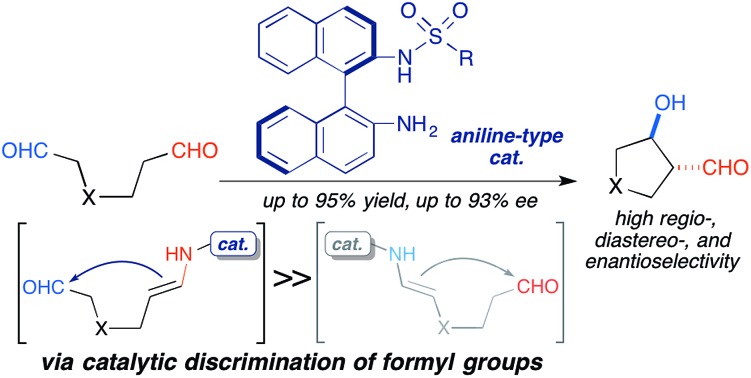
Catalytic discrimination between inequivalent formyl groups was achieved by virtue of the mild reactivity of an aniline-type acid–base catalyst for the regio-, diastereo-, and enantioselective intramolecular cross-aldol reactions.

## Introduction

**Fig. 1:**
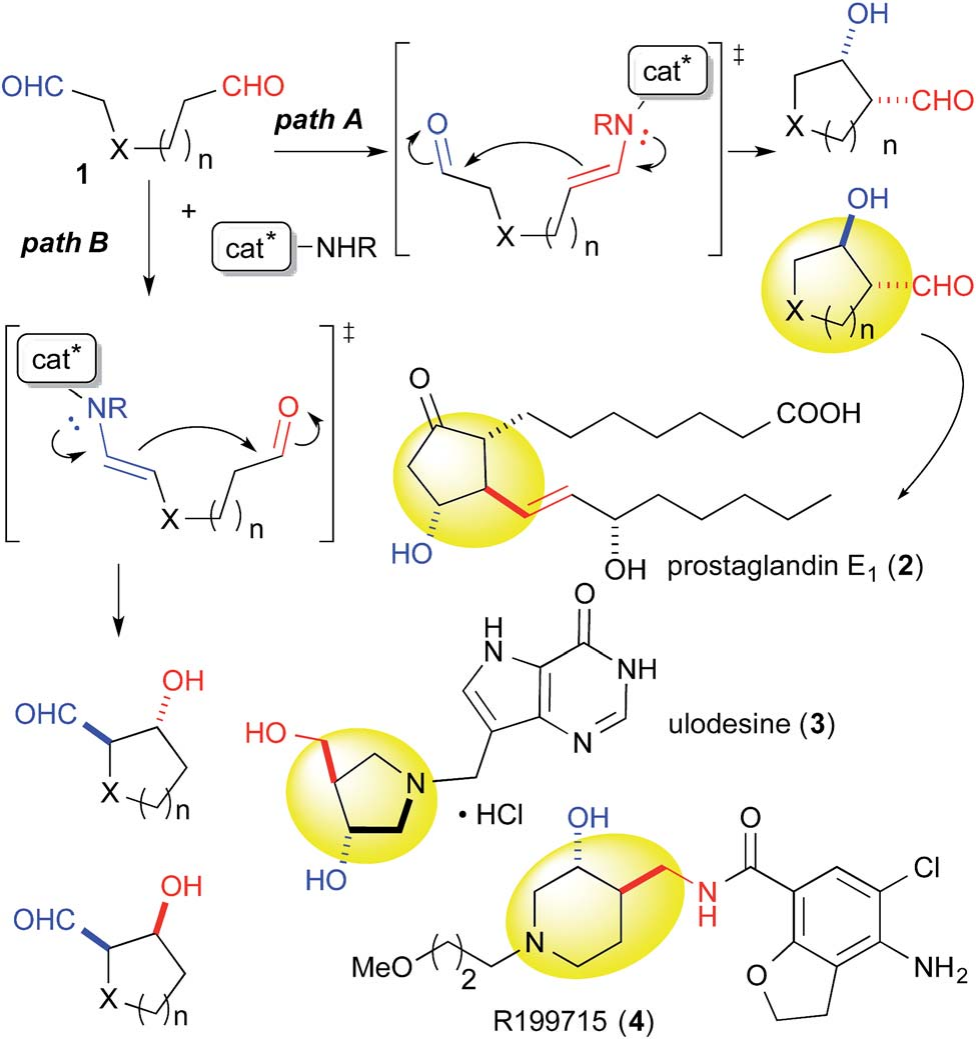
Possible regio- and stereoisomeric products from the intramolecular cross-aldol reaction of an enolizable aliphatic dial, and potentially preparable bioactive compounds bearing carbo- and heterocycles.

Catalytic discrimination among similarly reactive functional groups is a key to realizing unique and efficient chemo- and regioselective transformations of multi-functionalized molecules;^1^ however, formidable challenges to such discrimination remain. Intramolecular cross-aldol reactions of enolizable unsymmetric dial **1** require catalytic discrimination between the formyl groups bearing similar reactivities (Fig. 1). Although the reaction provides special versatility toward the production of cyclic β-hydroxy aldehydes potentially found in prostaglandins **2** and nucleic acid medicines **3**, control over this reaction is quite challenging due to the production of eight isomers from two regioisomers (from path A and B), including diastereomers (*anti*/*syn*), and enantiomers of each isomer (Fig. 1). Reaction selectively may only be achieved by controlling the diastereo- and enantioselectivities, in addition to controlling the regioselectivity of the products (path A *vs.* path B). In the amine-catalyzed reactions based on an enamine mechanism, a high regioselectivity is expected only under conditions that favor precise discrimination by the amine catalyst between two enolizable formyl groups. Under such conditions, these groups may be individually and selectively converted to the enamine component and the carbonyl component (Fig. 1).

In addition to these intramolecular reactions, selective direct intermolecular cross-aldol reactions between enolizable aliphatic aldehydes have been achieved.^2^,^3^ The slow addition of a donor or acceptor aldehyde using a syringe pump and/or the addition of excess amounts of one aldehyde to the other are indispensable for obtaining a good yield of the desired cross-aldol product in the case of α-unbranched aldehydes.^4^ Because these techniques cannot be employed in the intramolecular reaction of **1**, the success of the reaction relies purely on the potential of a catalyst to discriminate between formyl groups. For these reasons, intramolecular cross-aldol reactions are highly challenging transformations.

Indeed, we encountered difficulties in our efforts to apply the intramolecular cross-aldol reaction. An examination of the reaction of *N*-Ts dial **1a** in the presence of l-proline (5 mol%) toward an efficient synthesis of the chiral pyrrolidine (Table 2, entry 6) revealed that the reaction yielded an undesirable mixture of products. After NaBH_4_ reduction, the reaction mixture afforded nearly all possible regio- and stereoisomeric products, [*anti*-**5a** (9%, 6% ee), *syn*-**6** (31%, >99% ee), dehydrated **9** (2%)] and [*anti*-**7** (5%, 42% ee), *syn*-**8** (17%, 53% ee)], from the enamines of the C(6)- and C(1)-formyl groups, as well as the diol **10** (20%), which corresponded to residual starting material. The regioselectivity of the reaction (**5a** + **6** + **9**) : (**7** + **8**), was found to be 1.8 : 1. This undesirable result indicated that l-proline could not discriminate between the formyl groups of **1a**.

Herein, we describe the catalytic discrimination between formyl groups by aniline-type acid–base catalysts based on their distinct mild reactivities. This approach yielded the first examples of regio-, diastereo-, and enantioselective intramolecular cross-aldol reactions of enolizable unsymmetric dials.^5^

## Results and discussion

**Fig. 2:**
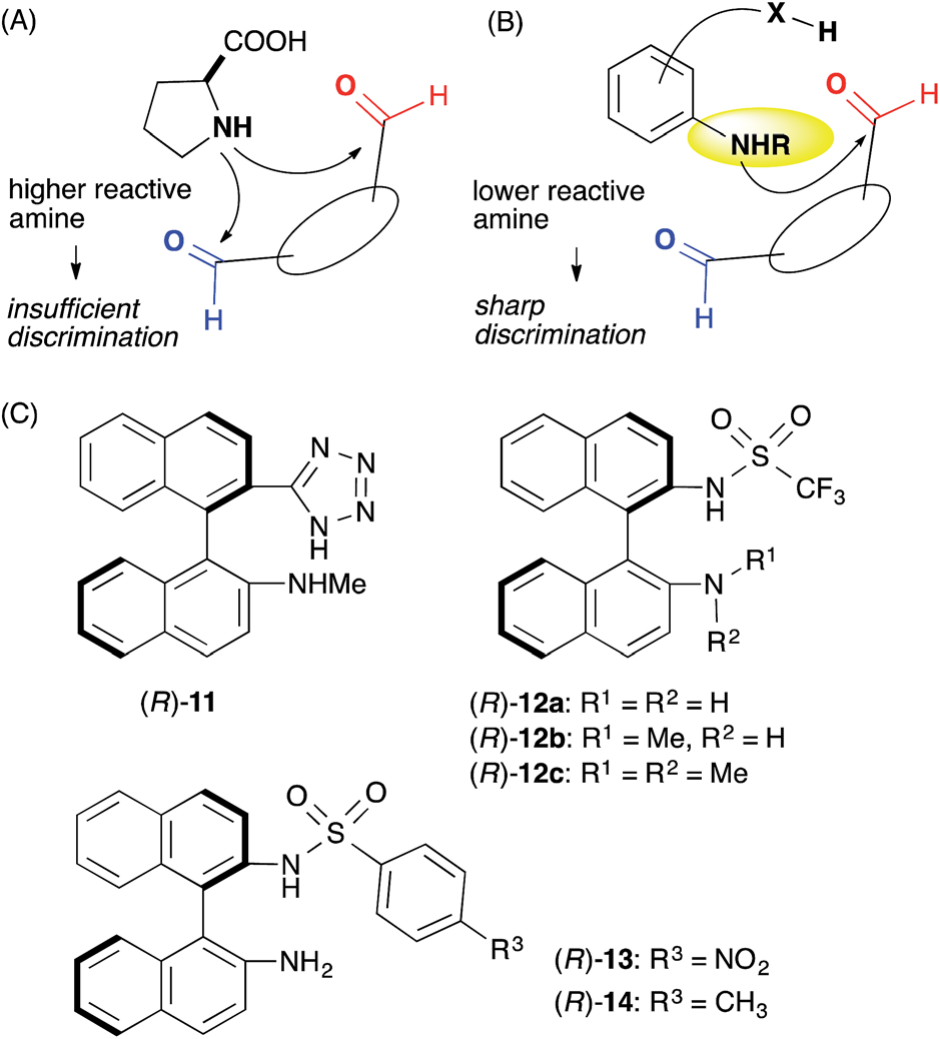
Working hypothesis underlying formyl group discrimination. (A) Aliphatic amino acid with a high reactivity. (B) Aniline-type acid–base catalyst with a low reactivity. (C) Axially chiral anilines bearing an acidic moiety.

We assumed that l-proline was too reactive to discriminate between formyl groups bearing similar reactivities (Fig. 2A). Therefore, we hypothesized that an acid–base catalyst bearing a mildly reactive amine could be advantageous in discriminating between the different formyl groups.^6^ Our focus centered on an aniline-type amine as an amine with one of the lowest reactivities (Fig. 2B). We were particularly interested in aniline-type axially chiral amino acids,^7^ and therefore we prepared (*R*)-**11** and (*R*)-**12**, **13**, **14**, which possessed tetrazole and sulfonamide groups as acidic moieties, respectively (Fig. 2C).^8^ Although aniline derivatives have been frequently employed as organocatalysts toward iminium activation,^9^ their application toward enamine catalysis has not received significant attention due to their weak basic and nucleophilic properties.^10^ Therefore, we initially evaluated the catalytic activities of these aniline catalysts by performing intramolecular enolexo-intramolecular aldol reaction of 1,6-hexanedial (**15**) (Table 1).^11^

**Table 1:** Enolexo-intramolecular aldol reaction of 1,6-hexanedial (**15**)

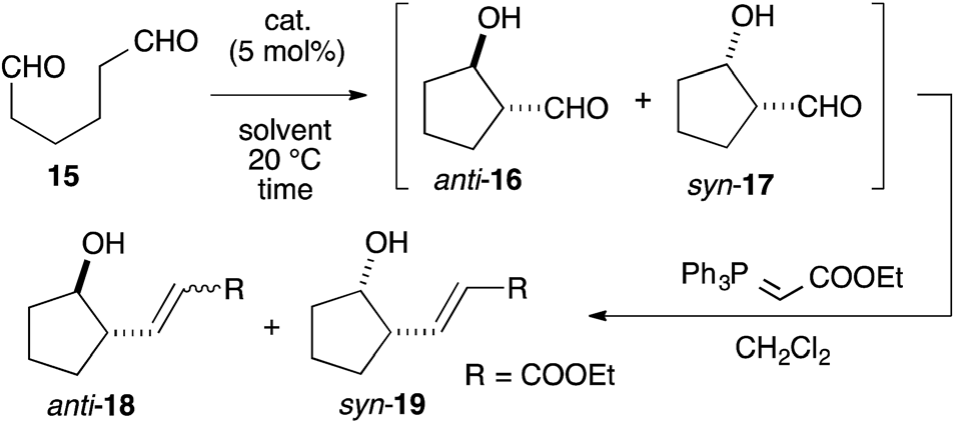					
Entry	Catalyst	Solvent	Time (h)	Yield^*a*^ (%) **18**^*b*^, **19**	d.r. (ee%) **18** (ee%) : **19**^*e*^ (ee%)
1	(*R*)-**11**	DMSO	96	40, 52	1 (50^*c*^) : 1.3 (93)
2	(*R*)-**12a**	DMSO	4	58, 9	6.4 (87^*d*^) : 1 (13)
3	(*R*)-**12a**	DMF	4	53, 12	4.4 (95^*d*^) : 1 (37)
4	(*R*)-**12a**	Acetone	24	62, 8	7.8 (95^*d*^) : 1 (34)
5	(*R*)-**12a**	THF	36	74, 5	15 (97^*d*^) : 1 (12)
6	(*R*)-**13**	DMSO	192	80, 5	16 (87^*d*^) : 1 (29)
7	l-Proline	DMSO	6	13, 59	1 (67^*d*^) : 4.5 (19)

^*a*^Determined by the integration of the ^1^H NMR signals in the presence of dibenzyl ether as an internal standard.

^*b*^The combined yield of the *E*/*Z* isomers.

^*c*^The absolute configurations of the major enantiomers of *anti*-**18** for entry 1 were determined to be (1*S*,2*R*).

^*d*^The absolute configurations of the major enantiomers of *anti*-**18** for entries 2–7 were determined to be (1*R*,2*S*).

^*e*^The absolute configuration of the major enantiomer of *syn*-**19** was determined to be (1*S*,2*S*).

To our delight, cat. (*R*)-**11** exhibited sufficient catalytic activity toward the reactions.^12^ In the presence of 5 mol% cat. (*R*)-**11**, the 5-membered ring-forming enolexo-intramolecular aldolization of **15** proceeded smoothly to afford *anti*-**16** and *syn*-**17** in DMSO. A subsequent Wittig olefination gave the corresponding *anti*-**18** and *syn*-**19** with a high enantioselectivity toward the *syn* isomer (93% ee), although the diastereoselectivity of the products was moderate (*anti*-**18** : *syn*-**19** = 1 : 1.3) (Table 1, entry 1). A survey of catalysts and solvents (see ESI†) identified the primary amine catalyst (*R*)-**12a**, which possessed a triflic amide, as capable of affording *anti*-**18** as the major product in a 58% yield with a high diastereo- (*anti* : *syn* = 6.4 : 1) and enantioselectivity (87% ee) (entry 2). DMF and acetone were good solvents for this reaction and improved the ee of *anti*-**18** (entries 3 and 4). Changing the solvent to THF increased the diastereo- and enantioselectivities as well as the chemical yield of *anti*-**18**, giving an *anti* : *syn* ratio of 15 : 1, an ee of 97%, and a 74% yield after 36 h (entry 5). The catalyst (*R*)-**13**, which bore a *p*-Ns group, was also active in this reaction and yielded *anti*-**18** in an 80% yield with an 87% ee in a high diastereoselectivity (*anti* : *syn* = 16 : 1) (entry 6). The opposite diastereo- and moderate enantioselectivities obtained in the presence of l-proline^13^ revealed that these axially chiral anilines provided a unique chiral environment suitable for the 1,6-dial (entry 7). The tertiary amine (*R*)-**12c** did not promote the reaction, suggesting that the aniline-type catalysts promoted the reaction *via* enamine catalysis. The catalytic activities listed in Table 1 verified that the anilines are useful organocatalysts, even in enamine catalysis.

**Fig. 3:**
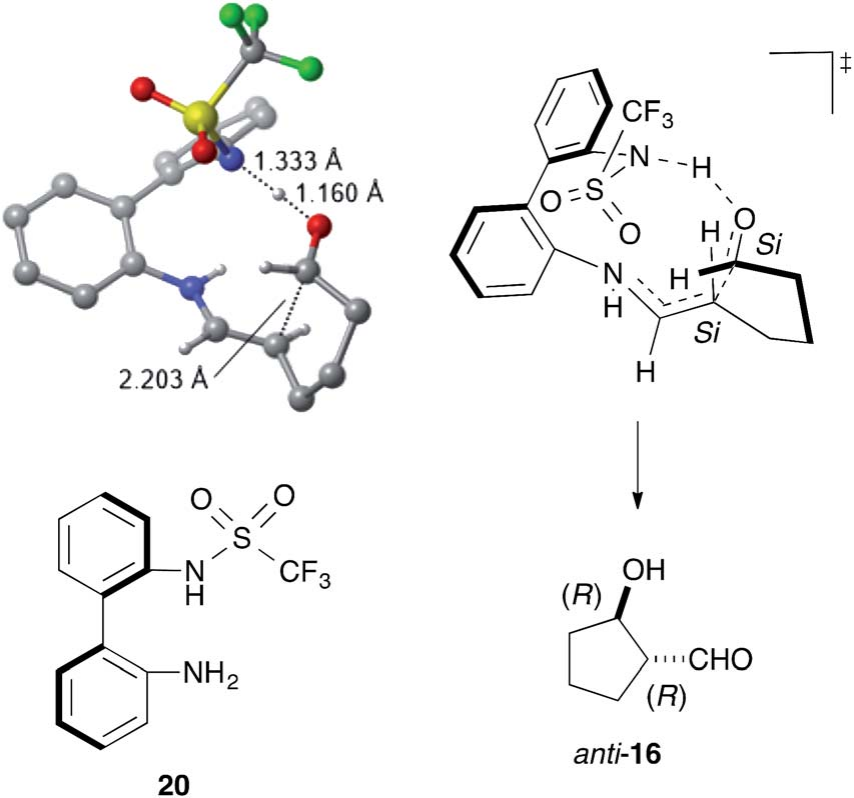
The most stable transition state for the stereo-determining C–C bond formation. Unimportant hydrogen atoms were omitted for clarify.

DFT calculations using the model catalyst **20** and the dial **15** also supported the enamine mechanism, including the rate-determining iminium-to-enamine transformation (see ESI†). The most stable transition state (TS) for the stereo-determining C–C bond-forming step explained the stereochemistry of *anti*-**16***via* C–C bond formation between the *Si* faces of the enamine and the formyl group (Fig. 3). The structural and electronic factors in the TS played a crucial role in controlling the stereoselectivity. The structurally favored conformation of the enamine and the C–C bond-forming moieties caused strong hydrogen bonds to form between the sulfonamide NH and the formyl carbonyl group, thereby facilitating a fit into the chiral space and stabilizing the TS for (1*R*,2*R*)-**16**.

With effective catalysts in hand, we moved again to examine the cross-aldol reaction of **1a** (Table 2). Although the reaction in the presence of cat. (*R*)-**12a** gave dehydrated **9** as the major product (see ESI†), the milder acidic (*R*)-**13** afforded 3,4-disubstituted *anti*-**5a** (59%) as the major product in 89% ee with a high diastereoselectivity (*anti*-**5a** : *syn*-**6** = 12 : 1) and the concomitant formation of the regioisomer, 2,3-disubstituted *anti*-**7** (8%) (Table 2, entry 1). The regioselectivity of the reaction, (**5a** + **6** + **9**) : (**7** + **8**), was found to be 8.0 : 1. This regioselectivity contrasts significantly with the corresponding value associated with the l-proline catalyzed reaction (entry 6). This selectivity indicated that cat. (*R*)-**13** discriminated between the different formyl groups, which could not be distinguished by l-proline, and converted the C(6)-formyl group into the enamine component and the C(1)-formyl group into the carbonyl component, as shown in Fig. 1 (path A). The reaction in DMF improved the diastereoselectivity to 18 : 1 and the enantioselectivity to 93% ee, although the regioselectivity decreased slightly (entry 2). During this survey, we found that **1a** was labile in DMSO and gave the opposite regioisomeric adducts *anti*-**7** and *syn*-**8** in the absence of a catalyst (regioselectivity, (**5a** + **6** + **9**) : (**7** + **8**) = 1 : 8.8) (entry 7). These results revealed that cat. (*R*)-**13** overcame the background reaction to predominantly yield *anti*-**5a**. We also tested the primary alkyl amine catalyst, l-isoleucine, which was successfully employed in the intermolecular cross-aldol reaction with an α-branched substrate (entry 5).3*j* This catalyst predominantly gave *anti*-**7** and *syn*-**8**; however, no significant ee value was obtained in *syn-***8**, and a diastereoselectivity (**7** : **8** = 1 : 1.2) similar to that of the background reaction (**7** : **8** = 1 : 1.1) (entry 7) suggested that this catalyst did not overcome the background reaction. The combination of aniline (**21**) and *p*-Ns aniline (**22**) also afforded *anti*-**7** and **8** as the major products (entry 4). These results indicated that cooperative activation of the substrate by acidic and basic moieties in biaryl framework was required for regioselectivity toward *anti*-**5a**.

**Table 2:** Intramolecular cross-aldol reaction of **1a**

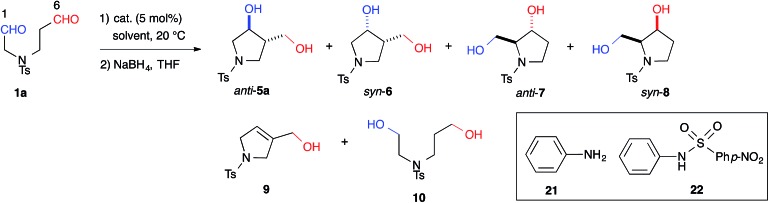									
Entry	Catalyst	Solvent	*t* (h)	Yield^*a*^ (%) **5a**^*b*^, **6**^*c*^, **7**^*c*^, **8**^*c*^, **9**, **10**	Regioselectivity (**5a** + **6** + **9**) : (**7** + **8**)	d.r. **5a** : **6**	d.r. **7** : **8**	ee (%) **5a**	ee (%) **8**
1	(*R*)-**13**	DMSO-*d*_6_	72	59, 5, 8^*d*^, 0, 0, 25	8.0 : 1	12 : 1	>99 : 1	89	—
2	(*R*)-**13**	DMF	72	54, 3, 10, 0, 0, 28	5.7 : 1	18 : 1	>99 : 1	93	—
3	(*R*)-**13**	THF	72	18, 4, 6, 0, 0, 50	3.7 : 1	4.5 : 1	>99 : 1	82	—
4	**21** + **22**	DMSO-*d*_6_	48	5, 6, 22, 34, 0, 14	1 : 5.1	1 : 1.2	1 : 1.9	—	—
5	l-Isoleucine	DMSO-*d*_6_	56	5, 6, 27, 31, 4, 15	1 : 3.8	1 : 1.3	1 : 1.2	n.d.	6
6	l-Proline	DMSO-*d*_6_	24	9^*e*^, 31^*e*^, 5^*e*^, 17, 2, 20	1.8 : 1	1 : 3.4	1 : 3.4	6	53^*f*^
7	—	DMSO-*d*_6_	72	3, 3, 37, 42, 0, 3	1 : 8.8	1 : 1.0	1 : 1.1	—	—

^*a*^Determined by the integration of the ^1^H NMR signals in the presence of dibenzyl ether as an internal standard.

^*b*^The absolute configurations of the major enantiomers of *anti*-**5a** for entries 1–3 were determined to be (3*S*,4*S*).

^*c*^The relative stereochemistry of all isomers was determined. The absolute configurations are tentatively based on the assumption that both products were generated from the same enamine geometry for *anti*-**5a**.

^*d*^74% ee was observed.

^*e*^6% ee, >99% ee, 42% ee, were observed for **5a**, **6**, and **7**, respectively.

^*f*^The absolute configurations of the major enantiomers was determined to be (2*R*,3*R*).

The yield of *anti*-**5a** was improved to 75% through elongation of the reaction time without decreasing the enantioselectivity (Table 3). The *N*-Alloc-, *N*-Cbz-, and *N*-Boc-protected dials **1b–1d** were applicable in this reaction and afforded *anti*-**5b–5d** in 90% ee (Table 3). It should be noted that **5d** was isolated as the sole product in 95% yield, suggesting that the formyl groups of **1d** were perfectly distinguished by cat. (*R*)-**13**. *Anti*-**5d** is an intermediate to the phosphorylase inhibitor, ulodesine (**3**, Fig. 1).^14^

**Table 3:** Intramolecular cross-aldol reaction to *N*-protected pyrrolidines

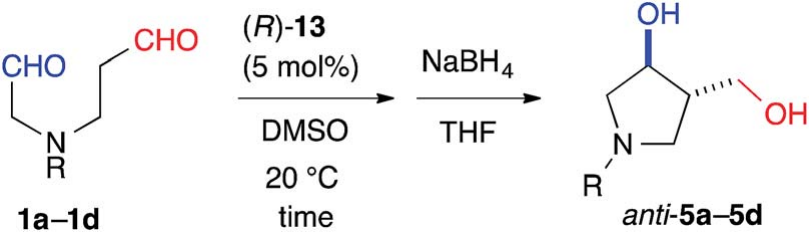
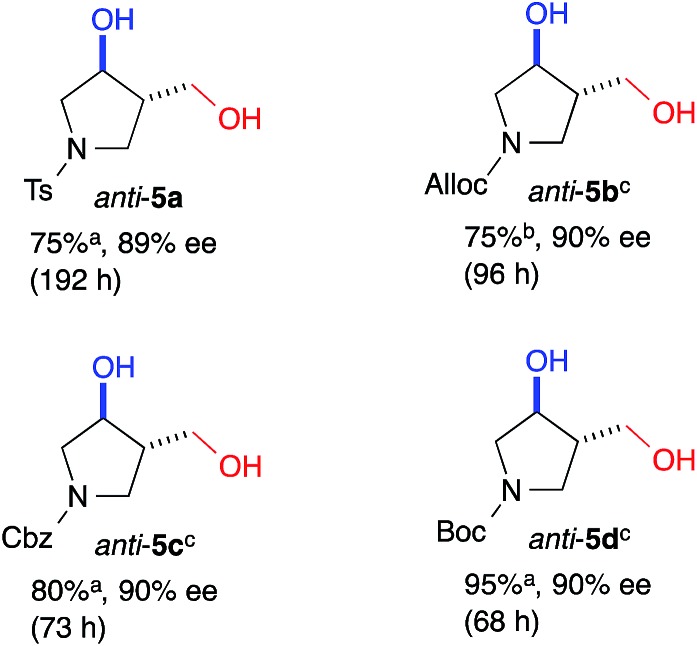

^*a*^Determined by the integration of the ^1^H NMR signals in the presence of dibenzyl ether as an internal standard.

^*b*^Determined by the integration of the ^1^H NMR signals in the presence of 1,3-dinitrobenzene as an internal standard.

^*c*^The absolute configurations of *anti*-(3*S*,4*S*)-**5b**–**5d** were determined by transforming into *anti*-**5a**.

The 6-membered ring-forming cross-aldol reactions were further examined under the conditions used for the 5-membered ring formation (Table 4). As expected, 3,4-*anti* disubstituted piperidine (**24**) was obtained from the reaction of *N*-Boc 1,7-dial (**23**) in the presence of 5 mol% cat. (*R*)-**13**, followed by successive NaBH_4_ reduction with high regio- and diastereoselectivities, although the enantioselectivity was low (entry 1). Unlike the five-membered ring formation, cat. (*R*)-**12a** did not promote dehydration and gave **24** as the major product, with a slightly better enantioselectivity (entry 2). Changing the solvent to THF and lowering the temperature to 0 °C in the presence of cat. (*R*)-**12a** improved the enantioselectivity to 86% ee with excellent regioselectivity (entry 3). The high regioselectivity indicated that the formyl groups of **23** were distinguished by the catalyst, leading to a reaction from the enamine of the C(7)-formyl group. This reaction provided a promising tool for constructing a 3,4-*anti* disubstituted chiral piperidine, such as R199715 (**4**, Fig. 1), previously prepared from **24**.^15^

**Table 4:** Intramolecular cross-aldol reaction to *N*-protected piperidine

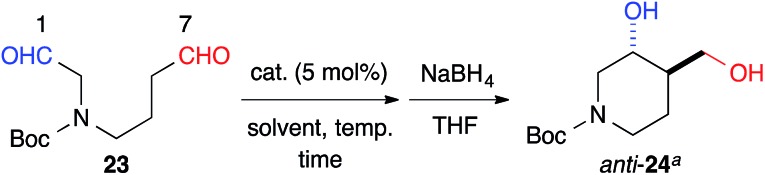						
Entry	Catalyst	Solvent	Temp (°C)	Time (h)	Yield^*b*^ (%)	ee (%)
1	(*R*)-**13**	DMSO	20	120	82	30
2	(*R*)-**12a**	DMSO	20	1.5	91	40
3	(*R*)-**12a**	THF	0	36	96	86

^*a*^The absolute configurations of *anti*-**24** was determined to be (3*R*,4*R*).

^*b*^Determined by the integration of the ^1^H NMR signals in the presence of 1,3-dinitrobenzene as an internal standard.

**Scheme 1:**
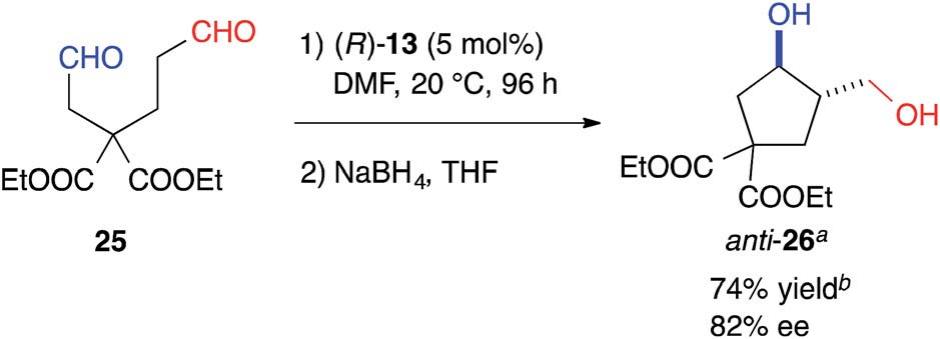
Intramolecular cross-aldol reaction to a chiral cyclopentane. ^*a*^The absolute configuration of *anti*-**26** was determined to be (3*R*,4*S*). ^*b*^Determined by the integration of the ^1^H NMR signals in the presence of 1,3-dinitrobenzene as an internal standard.

The formyl groups of **25** without a heteroatom were also discriminated well by cat. (*R*)-**13** to afford cyclopentane, *anti*-**26**, regioselectively in 74% yield with 82% ee (Scheme 1).

**Fig. 4:**
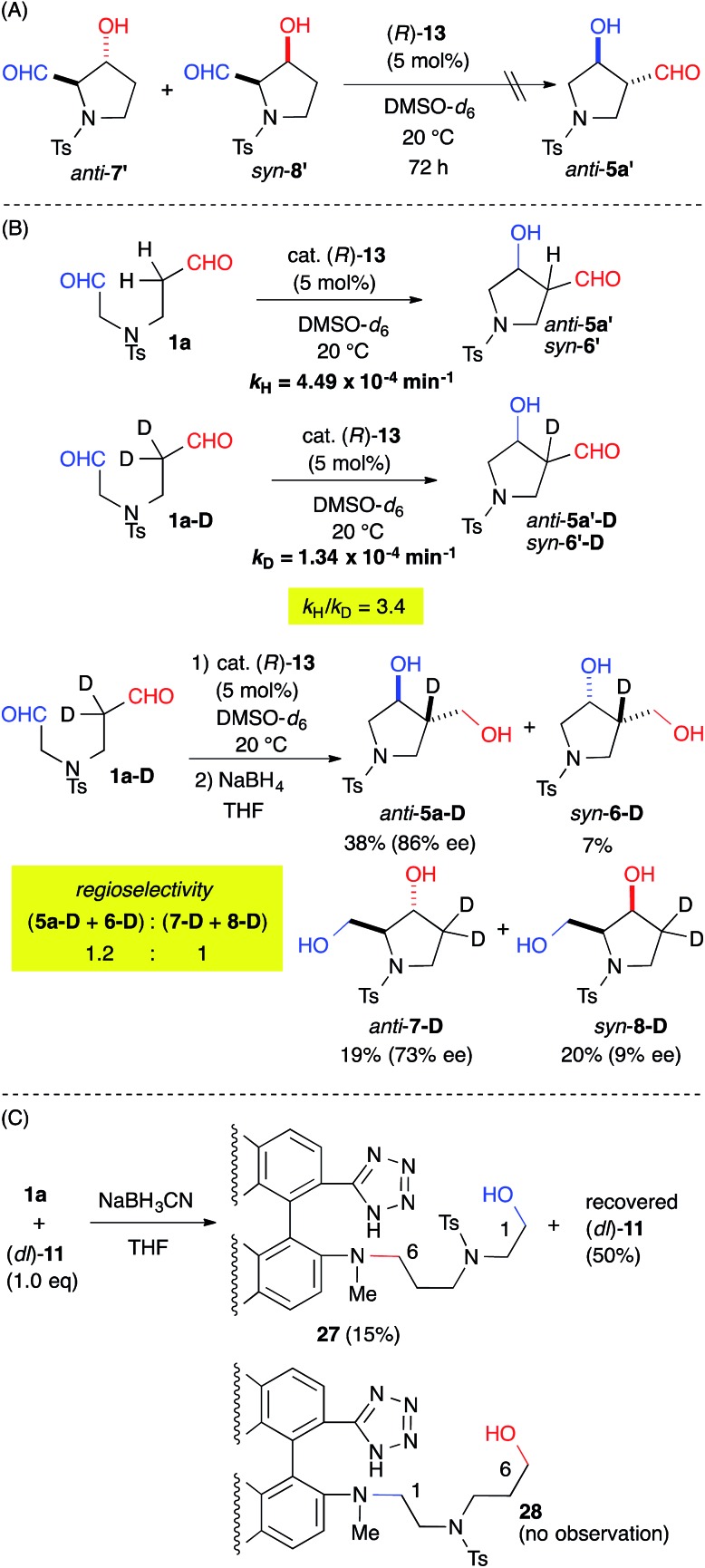
Mechanistic investigation.

The origin of the regioselectivity was explored by treating a mixture of the aldol-adducts *anti*-**7′** and *syn*-**8′**, the minor regioisomers, with cat. **13**. The absence of a conversion to *anti*-**5a′** suggested that the regioselectivity was governed by kinetic factors (Fig. 4A). The rate-determining step of the reaction was revealed by the kinetic isotope effect (KIE) using the α-deuterated substrate **1a-D**. The apparent primary KIE (*k*_H_/*k*_D_ = 3.4) indicated that the rate-determining step was associated with the enamine-forming step from the iminium intermediate, which involved C–H bond cleavage (Fig. 4B).^16^ Therefore, the regioselectivity of the reaction was defined by the enamine-forming steps rather than by the C–C bond-forming step. Furthermore, the regioselectivity of the reaction decreased to 1.2 : 1 for (**5a-D** + **6-D**) : (**7-D** + **8-D**) from 8.0 : 1 for (**5a** + **6** + **9**) : (**7** + **8**) (Table 2, entry 1). This result indicated that the kinetics associated with the iminium-to-enamine intermediate affected the regioselectivity.

A reductive amination of dial **1a** with the catalyst was also carried out to evaluate the regioselective iminium formation prior to the rate-determining step (Fig. 4C). The NaBH_3_CN reduction of an equimolar mixture of **1a** and cat. (*dl*)-**11**, which gave a regioselectivity {(**5a** + **6** + **9**) : (**7** + **8**): = 7.4 : 1} similar to that obtained from cat. (*R*)-**13** (see ESI†), gave **27** in a 15% yield with a 50% recovery of (*dl*)-**11** without any observable appearance of **28**. This result suggested predominant iminium formation at the C(6)-formyl group of **1a**.

**Fig. 5:**
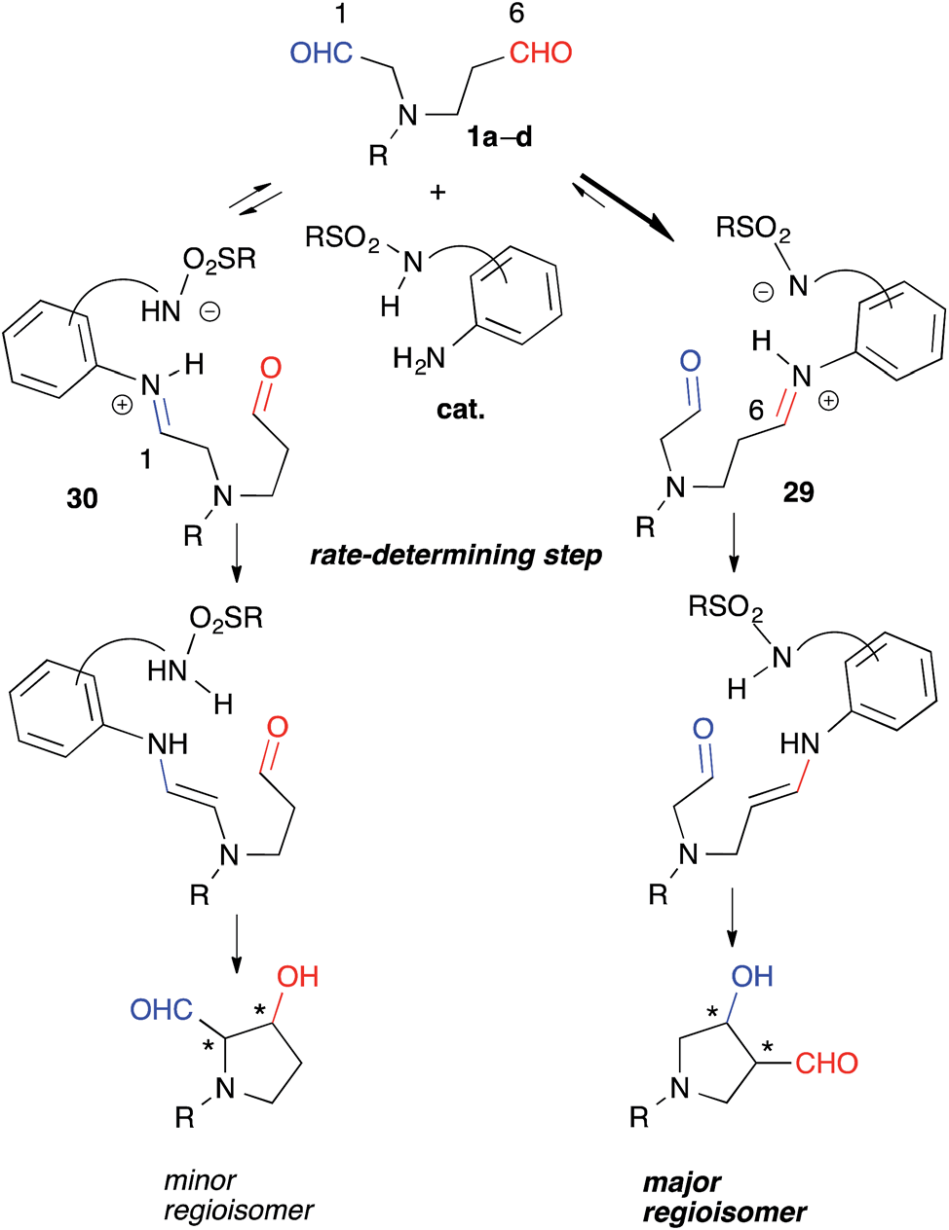
Possible explanation for regioselectivity.

These experiments suggested that the regioselectivity was controlled by the kinetics of the enamine formation. Selective iminium formation at the C(6)-formyl group ([**29**] > [**30**]) of **1a-1d** may have affected the kinetics associated primarily with the production of the major regioisomers (Fig. 5). The thermodynamic stability of **29** due to the iminium cation located two carbons away from the electron-withdrawing NR group may have contributed to the predominant formation of **29** relative to **30** during the equilibration step. The steric factor of the catalyst may also play a role in the preferential formation of the sterically less congested **29** than **30**. The regioselective formation of *anti*-**26** from dial **25** bearing *gem*-diester group instead of the NR group might be mainly governed by the steric factor (Scheme 1). The mild reactivity of the aniline-type catalyst may have facilitated the discrimination between tiny electronic differences and/or steric circumstances of the C(1)- and C(6)-formyl groups.^17^

## Conclusions

In summary, catalytic discrimination among formyl groups was achieved in the highly regio-, diastereo-, and enantioselective intramolecular cross-aldol reactions of enolizable 1,6- and 1,7-dials. The key to realizing formyl group discrimination was the mild reactivity of the aniline-type acid–base catalysts, which led to excellent regioselectivity. Mechanistic investigations including kinetic isotope effect studies and reductive amination experiments revealed that the regioselectivity was controlled under the enamine-forming steps. The high accessibility to the chiral pyrrolidines and piperidines provided a prominent feature of this cross-aldol reaction. Further mechanistic investigations are currently underway.

## Supplementary Material

Supplementary information
