# Supplementary material for: Catalytic discrimination between formyl groups in regio- and stereoselective intramolecular cross-aldol reactions
Source: Chem Sci. 2016 Feb 22;7(6):3791–7. doi: 10.1039/c5sc04594k (PMC6013812; doi:10.1039/c5sc04594k)

Electronic Supplementary Material (ESI) for Chemical Science.
This journal is © The Royal Society of Chemistry 2016

Electronic Supplementary Information

Catalytic Discrimination Between Formyl Groups in Regio- and Stereoselective Intramolecular Cross-Aldol Reactions

Tomonori Baba,[a] Junya Yamamoto,[a] Kazuhiro Hayashi,[a] Makoto Sato,[b] Masahiro Yamanaka,[b] Takeo Kawabata,[a] and Takumi Furuta*[a]

[a]Institute for Chemical Research, Kyoto University, Uji, Kyoto 611-0011, Japan
[b]Department of Chemistry and Research Center for Smart Molecules, Faculty of Science, Rikkyo University, 3-34-1 Nish-Ikebukuro, Toshima-ku, Tokyo 171-8501, Japan

E-mail: furuta@fos.kuicr.kyoto-u.ac.jp

Content:

General Information S-3
List of abbreviation S-3
Preparation of Catalyst (*R*)-**11** S-4
Preparation of catalysts (*R*)-**12a**–**12c** S-6
Preparation of catalyst (*R*)-**13** S-7
Reactivity of (*dl*)-**11** in the self-aldol reaction of dodecanal S-8
Survey of the reaction conditions for *enolexo*-intramolecular aldol reaction of 1,6-hexanedial (**15**). S-9
Typical procedure of the intramolecular aldol reaction and determination of the absolute configuration of the aldol products for Table 1, Entry 1 and Table S1, Entry 1. S-9
HPLC analysis of *anti*-(*E*)-**18** S-12
HPLC analysis of *syn*-(*E*)-**19** S-13
Survey of the reaction conditions for intramolecular cross-aldol reaction of *N*-Ts dial (**1a**). S-14
Typical procedure of the intramolecular cross-aldol reaction of **1a** in the presence of cat. (*R*)-**13** (Table 2, Entry 1) S-14
Procedure of the intramolecular cross-aldol reaction of **1a** in the presence of L-proline (Table 2, Entry 6) S-15
Characterization and determination of the stereochemistries of the aldol products for Table 2 S-15
Characterization of the aldol products for Table 3 S-20
Preparation of *N*-containing dials for Tables 2 and 3 S-22
Typical procedure of the intramolecular cross-aldol reaction of **23** (Table 4, Entry 3) S-25
Characterization and determination of the stereochemistry of *anti*-**24** for Table 4 S-25
Preparation of dial **23** for Table 4 S-27
Procedure of the intramolecular cross-aldol reaction of **25** and determination of the absolute configuration of aldol product **26** (Scheme 1) S-28

Preparation of dial **25** for Scheme 1 S-30
Treatment of the mixture of aldol-adducts *anti*-**7'** and *syn*-**8'** with cat. (*R*)-**13** S-31
Preparation of **1a-D** for Figure 4B S-32
KIE experiment for Figure 4B S-33
Reductive amination for Figure 4C S-35
Computational details S-36
Mechanism analysis S-36
Cartesian coordinates of optimized structures S-40
References S-62
^{1}H and ^{13}C NMR spectra S-63

General

^{1}H NMR spectra were obtained at 400 or 600 MHz with chemical shifts being given in ppm units (tetramethylsilane, solvent resonance of DMSO-d_6 as internal standards, indicating 0 and 2.49, respectively). ^{13}C NMR spectra were measured at 100 or 150 MHz with chemical shifts being given in ppm units (tetramethylsilane, solvent resonance of DMSO-d_6 and $CDCl_3$ as internal standards, indicating 0, 39.7 and 77.0, respectively). IR spectra were recorded on a FT-IR spectrometer. Specific rotation was measured with an automatic digital polarimeter. MS spectra were recorded by EI or FAB mass spectrometer. TLC analysis and preparative TLC were performed on commercial glass plates bearing a 0.25 mm layer or 0.5 mm layer of silica gel. Silica gel chromatography was performed with 150-325 mesh silica gel.

Dry solvents (acetone, DMF, DMSO, THF) obtained from commercial suppliers were used without further purification.

List of abbreviation

AcOEt	ethyl acetate
DIPEA	diisopropylethylamine
DMF	dimethylformamide
DMSO	dimethylsulfoxide
EDCI	1-ethyl-3-(3-dimethylaminopropyl)carbodiimide hydrochloride
HOBt	1-hydroxybenzotriazole
NMO	*N*-methylmorphorine *N*-oxide
TFA	trifluoroacetic acid
THF	tetrahydrofuran

Preparation of Catalyst (*R*)-11

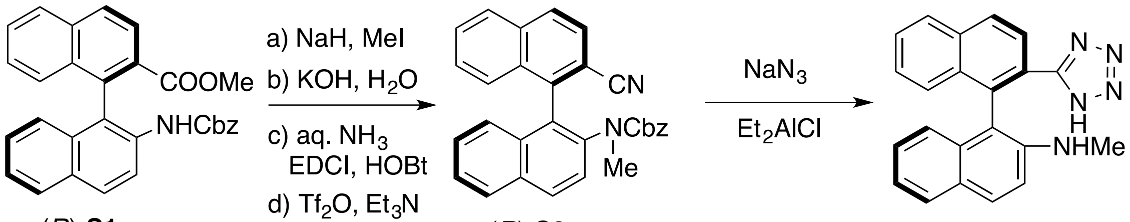

(*R*)-**S1** (*R*)-**S2** (*R*)-**11**

(*R*)-Benzyl (2'-cyano-[1,1'-binaphthalen]-2-yl)(methyl)carbamate ((*R*)-**S2**)

To a solution of (*R*)-**S1**[1] (300 mg, 0.64 mmol) in THF (9 mL) were added NaH (65 mg, 0.98 mmol) and MeI (0.4 mL, 6.4 mmol) at 0 ℃ under Ar atmosphere. After being stirred for 1.5 h at rt, the solvent was evaporated to give the residue.

The residue was diluted with THF/MeOH/H_2O (2 : 1 : 1, 8 mL) and added KOH (359 mg, 6.4 mmol) at rt. After being stirred for 12 h at rt, the reaction was quenched with saturated aq. NH_3Cl and extracted with AcOEt. The extract was washed with brine, dried over anhydrous Na_2SO_4, filtered, and evaporated to give the residue (297 mg).

The residue (297 mg) was diluted with THF (7 mL), and added EDCI (184 mg, 0.96 mmol) and HOBt (129.7mg, 0.96 mmol) at rt. After being stirred for 3 h at rt, the reaction was added aq. NH_3 (30% solution, 30 mL) and further stirred for 30 min. The reaction was quenched with saturated aq. $NaHCO_3$ and extracted with AcOEt. The extract was washed with 2 *N* aq. HCl, brine, dried over anhydrous Na_2SO_4, filtered, and evaporated to give the residue (333 mg).

To a solution of the residue (333 mg) in CH_2Cl_2 (14 mL) were added Tf_2O (0.15 mL, 0.9 mmol) and TEA (0.18 mL, 2.27 mmol) at 0 ℃ under Ar atmosphere. After being stirred for 30 min at rt, the reaction was quenched with saturated aq. $NaHCO_3$ and extracted with AcOEt. The extract was washed with brine, dried over anhydrous Na_2SO_4, filtered, and evaporated to give the residue (483 mg). The residue was purified by column chromatography on silica gel (*n*-hexane/AcOEt, 6:1) to afford (*R*)-**S2** (295 mg, 87%).

Colorless needles (*n*-hexane-AcOEt). M.p. 63 ℃. $[\alpha]_D^{20}$ = –18 (c 1.1, $CHCl_3$). ^{1}H NMR (600 MHz, DMSO-d_6, 80 °C, rotamers) δ 2.89 (br s, 3H), 4.20-5.10 (m, 2H), 6.97 (d, *J* = 8.3 Hz, 1H), 7.05 (br s, 1H), 7.15-7.35 (m, 5H), 7.36-7.40 (m, 1H), 7.54-7.59 (m, 1H), 7.62-7.69 (m, 2H) 7.92 (d, *J* = 8.3 Hz, 1H), 8.09 (d, *J* = 8.2 Hz, 1H), 8.13 (d, *J* = 8.2 Hz, 1H), 8.16-8.26 (m, 3H). ^{13}C NMR (150 MHz, DMSO-d_6, 80 °C) δ 39.4, 66.1, 78.9, 110.9, 118.0, 124.7, 126.35, 126.43, 126.6, 126.9, 127.2, 127.3, 127.4, 128.0, 128.1, 128.2, 128.9, 129.5, 130.4, 130.7, 131.2, 132.0, 132.2, 134.3, 136.4, 139.7, 140.7, 154.3. IR (KBr) 3064, 2940, 2223, 1705 cm^{-1}. MS (FAB) *m/z* 443 $(M+H)^+$, 465 $(M+Na)^+$. HRMS (FAB) *m/z* calcd for $C_{30}H_{23}N_2O_2$ $(M+H)^+$ 443.1760, found 443.1751.

(*R*)-*N*-methyl-2'-(1H-tetrazol-5-yl)-[1,1'-binaphthalen]-2-amine ((*R*)-**11**)

To a suspension of NaN_3 (122 mg, 2.21 mmol) in THF (9 mL) was added Et_2AlCl (0.92mol/L in hexane, 2.4 mL, 2.21 mmol) at 0 ℃ under Ar atmosphere. After being stirred for 12 h at rt, the solvent was concentrated under reduced pressure. To a concentrated solution was added (*R*)-**S2** (144 mg, 0.28 mmol) under Ar atmosphere. After being stirred for 20 h at 136 °C, the reaction was quenched with aq. 2 *N* NaOH and neutralized with aq. 2 *N* HCl, and extracted with AcOEt. The extract was washed with brine, dried over anhydrous Na_2SO_4, filtered, and evaporated to give the residue. The residue was purified by column

chromatography on silica gel ($CHCl_3$/MeOH, 15:1) to afford (*R*)-**11**. (295 mg, 87%).

Light yellow needles (*n*-hexane-toluene). M.p. 235 ℃. $[\alpha]_D^{20}$ = –113 (c 0.5, $CHCl_3$). ^{1}H NMR (400MHz, $CDCl_3$) δ 2.80 (s, 3H), 6.72 (d, *J* = 8.2 Hz, 1H), 7.10-7.45 (m, 5H), 7.55-7.65 (m, 1H), 7.85 (d, *J* = 7.8 Hz, 1H), 8.00-8.10 (m, 2H), 8.18 (d, *J* = 8.7 Hz, 1H), 8.58 (d, *J* = 8.7 Hz, 1H). ^{13}C NMR (150 MHz, $CDCl_3$) δ 30.8, 112.4, 113.3, 122.2, 122.8, 123.0, 126.48, 126.51, 127.4, 127.8, 128.0, 128.4, 128.48, 128.53, 129.8, 131.5, 132.6, 132.9, 133.7, 135.3, 144.5, 153.8. IR (KBr) 3383, 3051, 2909, 2821, 1619 cm^{-1}. MS (FAB) *m/z* 352 $(M+H)^+$, 374 $(M+Na)^+$. HRMS (FAB) *m/z* calcd for $C_{22}H_{18}N_5$ $(M+H)^+$ 352.1562, found 352.1587; Anal calcd. for $C_{22}H_{17}N_5$: C, 75.19; H, 4.88; N,19.93. Found: C, 75.22; H, 4.81; N,19.66.

Preparation of catalysts (*R*)-12a–12c

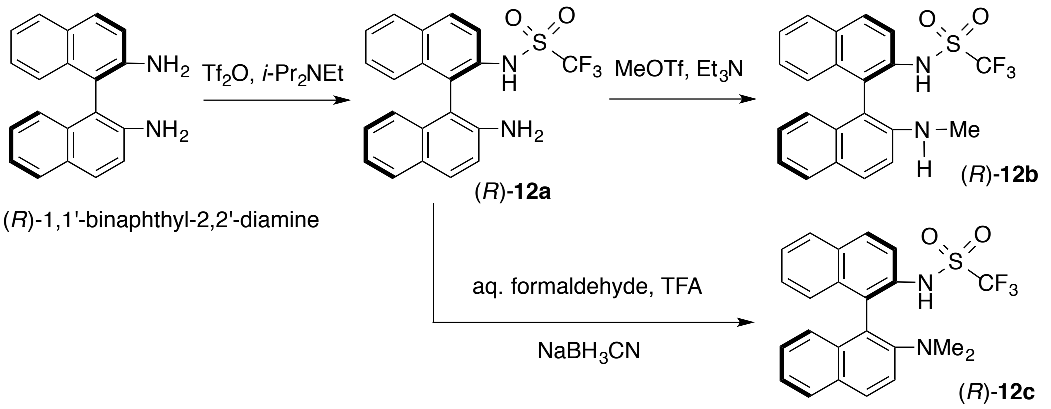

(*R*)-2'-Amino-2-trifluoromethanesulfonylamino-1,1'-binaphthyl (**12a**)

The solution of (*R*)-1,1'-binaphthyl-2,2'-diamine (300 mg, 1.06 mmol), and DIPEA (0.22 ml, 1.27 mmol) in CH_2Cl_2 (5.0 mL) was added trifluoromethanesulfonic anhydride (0.21 ml, 1,27 mmol) at -78 °C for 1 h under Ar atmosphere. The mixture was warmed to rt with stirring for 19 h. The reaction was quenched with H_2O, and extracted with AcOEt. The extract was washed with brine, dried over anhydrous Na_2SO_4, filtered, and evaporated. The crude residue was purified by column chromatography on silica gel (*n*-hexane/AcOEt, 5:1 to *n*-hexane/AcOEt, 4:1) to afford a pale yellow oil. The resulting pale yellow oil was recrystallized from $CHCl_3$ and *n*-hexane to afford **12a** (342 mg, 78%) as colorless plates.

Colorless plates (*n*-hexane-$CHCl_3$). M.p. 159-161 °C. $[\alpha]_D^{22}$ = −43.0 (*c* 1.0, $CHCl_3$). ^1H NMR (400 MHz, $CDCl_3$) δ 3.69 (s, 2H), 6.83 (d, *J* = 8.3 Hz, 1H), 7.15 (d, *J* = 8.7 Hz, 1H), 7.18-7.38 (m, 5H), 7.46-7.55 (m, 1H), 7.83 (d, *J* = 7.3 Hz, 1H), 7.89 (d, *J* = 8.7 Hz, 1H), 7.96 (d, *J* = 9.2 Hz, 2H), 8.04 (d, *J* = 9.2 Hz, 1H). ^{13}C NMR (150 MHz, $CDCl_3$) δ 109.7, 118.1, 119.4 (q, $J_{C\text{-}F}$ = 321.6 Hz), 120.0, 123.1, 123.3, 124.4, 126.1, 126.4, 127.5, 127.6, 128.3, 128.4, 130.1, 131.1, 131.6, 132.1, 132.5, 133.3, 142.4. IR (KBr) 3563, 3408, 3331, 1622, 1422, 1219, 1145, 988, 820, 754, 592 cm^{-1}. MS (FAB) m/z 416 $(M)^+$. HRMS (FAB) m/z calcd for $C_{21}H_{15}F_3N_2O_2S_1$ $(M)^+$ 416.0806, found 416.0803.

(*R*)-2'-Methylamino-2-trifluoromethanesulfonylamino-1,1'-binaphthyl (**12b**)

The solution of (*R*)-**12a** (60 mg, 0.14 mmol) in CH_3CN (2.0 mL) was added MeOTf (16 μL, 0.14 mmol) at rt and the reaction mixture was stirred at 80 °C overnight under Ar atmosphere. The reaction was quenched with $NaHCO_3$, and extracted with CH_2Cl_2. The extract was washed with brine, dried over anhydrous Na_2SO_4, filtered, and evaporated. The crude residue was purified by column chromatography on silica gel (*n*-hexane/AcOEt, 8:1) to afford **12b** (23 mg, 37%) as brown amorphous.

Brown amorphous. $[\alpha]_D^{22}$ = −48.0 (*c* 0.4, $CHCl_3$). ^1H NMR (600 MHz, $CDCl_3$) δ 2.85 (s, 3H), 6.75 (d, *J* = 8.9 Hz, 1H), 7.16-7.27 (m, 3H), 7.29-7.37 (m, 1H), 7.46-7.55 (m, 1H), 7.82 (d, *J* = 7.6 Hz, 1H), 7.92-8.01 (m, 3H), 8.04 (d, *J* = 8.9 Hz, 1H). ^{13}C NMR (150 MHz, $CDCl_3$) δ 30.6, 108.2, 113.1, 119.41 (q, $J_{C\text{-}F}$ = 320.3 Hz), 119.42, 122.4, 122.8, 123.9, 126.0, 126.4, 127.4, 127.5, 127.6, 128.3, 128.5, 130.2, 131.4, 131.9, 132.1, 132.8, 133.3, 145.1. IR ($CHCl_3$) 3302, 1599, 1511, 1325, 869, 816, 681 cm^{-1}. MS (FAB) m/z 430 $(M)^+$, 453 $(M+Na)^+$. HRMS (FAB) m/z calcd for $C_{22}H_{17}F_3N_2O_2S_1$ $(M)^+$ 430.0963, found 430.0963.

(*R*)-2,2'-Dimethylamino-2-trifluoromethanesulfonylamino-1,1'-binaphthyl (**12c**)

The solution of (*R*)-**12a** (15 mg, 0.036 mmol) and HCHO (11 mg, 0.36 mmol) in THF (3.0 mL) was added

$NaBH_4$ (6.8 mg, 0.18 mmol) at 0 °C and the reaction mixture was stirred at rt 34 h under Ar atmosphere. The reaction was quenched with 1 N HCl aq, and extracted with AcOEt. The extract was washed with brine, dried over anhydrous Na_2SO_4, filtered, and evaporated. The crude residue was purified by prep. TLC (SiO_2, *n*-hexane/AcOEt, 4:1) to afford **12c** (6.7 mg, 42%) as colorless amorphous.

Colorless amorphous. $[\alpha]_D^{22} = -309.0$ (*c* 0.6, $CHCl_3$). ^1H NMR (600 MHz, $CDCl_3$) δ 2.63 (s, 6H), 6.82 (d, *J* = 8.7 Hz, 1H), 7.08-7.15 (m, 1H), 7.15-7.19 (m, 1H), 7.24-7.29 (m, 1H), 7.32-7.36 (m, 1H), 7.45-7.49 (m, 1H), 7.52 (d, *J* = 9.0 Hz, 1H), 7.83-7.87 (m, 2H), 7.92-7.96 (m, 1H), 7.98-8.04 (m, 2H). ^{13}C NMR (100 MHz, $CDCl_3$) δ 43.6, 117.7, 119.3 (q, $J_{C\text{-}F}$ = 321.0 Hz), 123.4, 123.6, 124.6, 126.1, 126.2, 126.5, 127.1, 127.2, 127.9, 128.3, 129.2, 129.7, 130.1, 130.7, 130.8, 132.3, 133.5, 133.8, 148.3. IR (neat) 3583, 2348, 1598, 1462, 1375, 1249, 1141, 872, 769 cm^{-1}. MS (FAB) m/z 444 $(M)^+$. HRMS (FAB) m/z calcd for $C_{23}H_{19}F_3N_2O_2S_1$ $(M)^+$ 444.1119, found 444.1115.

Preparation of catalyst (*R*)-13

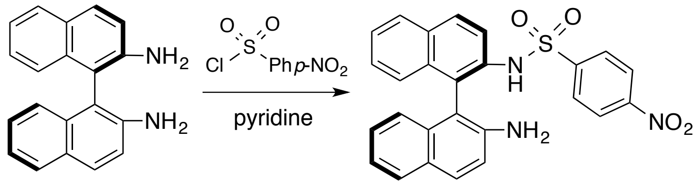

(*R*)-1,1'-binaphthyl-2,2'-diamine (*R*)-**13**

To a solution of (*R*)-1,1'-binaphthyl-2,2'-diamine (200 mg, 0.70 mmol) in CH_2Cl_2 (8 mL) were added pyridine (0.68 mL, 8.4 mmol) and *p*-NsCl (203 mg, 0.91 mmol) at 0 ℃ under Ar atmosphere. After being stirred for 24 h at rt, the reaction was quenched with aq. 5% (v/v) HCl, and extracted with AcOEt. The extract was washed with aq. 1% (v/v) HCl and brine, dried over anhydrous Na_2SO_4, filtered, and evaporated to give the residue. The residue was purified by column chromatography on silica gel (hexane/AcOEt, 5:1 to 4:1) to afford (*R*)-**13** (241 mg, 73%) as yellow plates.

Yellow plates (*n*-hexane-$CHCl_3$). M.p. 118-121 °C. $[\alpha]_D^{22} = -70.3$ (*c* 0.5, $CHCl_3$). ^1H NMR (600 MHz, $CDCl_3$) δ 3.65 (s, 2H), 6.15 (d, *J* = 7.8 Hz, 1H), 6.71-6.81 (m, 1H), 7.01-7.14 (m, 3H), 7.17-7.24 (m, 1H), 7.31-7.40 (m, 3H), 7.41-7.49 (m, 1H), 7.62-7.71 (m, 3H), 7.80 (d, *J* = 8.7 Hz, 1H), 7.91 (d, *J* = 8.7 Hz, 1H), 8.00 (d, *J* = 8.7 Hz, 1H), 8.07 (d, *J* = 8.7 Hz, 1H). ^{13}C NMR (150 MHz, $CDCl_3$) δ 111.2, 117.9, 122.6, 123.1, 123.7, 125.0, 126.1, 126.4, 126.8, 127.1, 127.4, 127.9, 128.0, 128.2, 129.9, 130.6, 132.06, 132.10, 132.7, 133.3, 141.6, 144.7, 149.5. IR ($CHCl_3$) 3451, 3364, 3318, 3031, 1619, 1532, 1510, 1468, 1433, 1350, 1316 cm^{-1}. MS (FAB) m/z 469 $(M)^+$. HRMS (FAB) m/z calcd for $C_{26}H_{19}N_3O_4S_1$ $(M)^+$ 469.1096, found 469.1094.

Reactivity of (*dl*)-11 in the self-aldol reaction of dodecanal

The catalytic activity of aniline catalyst was evaluated by the intermolecular self-aldol reaction of dodecanal. Although 10 mol% of (*dl*)-proline gave the self-aldol adduct in 79% yield, (*dl*)-**11** did not give the product at all under the same conditions. Primary amino acid, (*dl*)-isoleucine, yielded the product in 14% yield. These experiments proved the low reactivity of aniline-type catalyst, and showed the reactivity order of these catalysts in the intermolecular reaction is proline > isoleucine > (*dl*)-**11**.

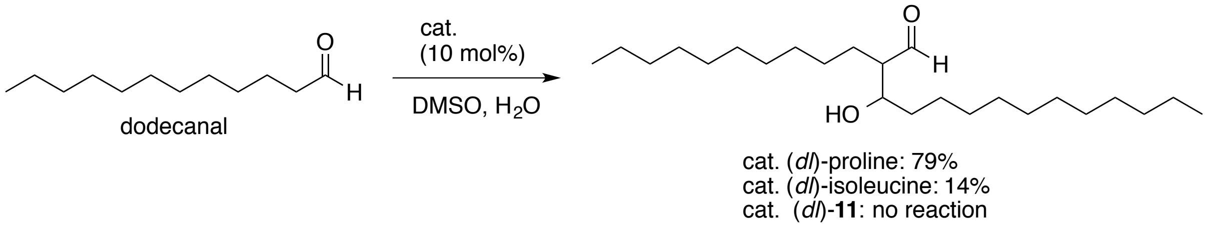

Experimental procedure: To a solution of dodecanal (100mg, 0.54 mmol) in DMSO (1.0 mL) and H_2O (0.05 mL) was added catalyst (0.054 mmol). After being stirred for 4 h at 20 °C, the reaction was quenched with sat. aq. NH_4Cl, and extracted with Et_2O. The extract was washed with brine, dried over anhydrous Na_2SO_4, filtered, and evaporated. The yields of the aldol adduct were determined by the integration of ^{1}H NMR signals in the presence of 1,3-dinitrobenzene as an internal standard.

Self-aldol product[2]: MS (FAB) m/z 369 $(M+H)^+$. HRMS (FAB) m/z calcd for $C_{24}H_{49}O_2$ $(M+H)^+$ 369.3757, found 369.3752.

Survey of the reaction conditions for *enolexo*-intramolecular aldol reaction of 1,6-hexanedial (15).

Table S1, Entries 2–7, and 10 were depicted in Table 1 in the paper. Herein, the results with cat. (*R*)-**12b**, **14**, and **12c**, as well as the yields of **S3** and **S4**, correspond to the dehydrated adduct and the starting material recovery, respectively, were included.

Table S1. *Enolexo*-intramolecular aldol reaction of 1,6-hexanedial (**15**).

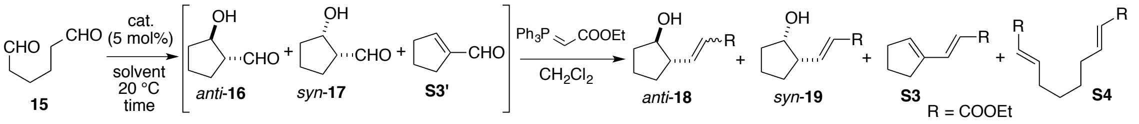

entry	catalyst	solvent	time (h)	yield (%)[a] 18[b], 19, S3, S4	d.r. *anti*-**18** : *syn*-**19**	ee (%)[d] *anti*-**18**/ *syn*-**19**[g]
1	(*R*)-**11**	DMSO	192	40, 52, n.d.	1 : 1.3	50[e] / 93
2	(*R*)-**12b**	DMSO	4	6, 2, 65, 4	3.0 : 1	n.d.
3	(*R*)-**12a**	DMSO	4	58, 9, 31, <1	6.4 : 1	87[f] / 13
4	(*R*)-**12a**	DMF	4	53, 12, 31, 0	4.4 : 1	95[f] / 37
5	(*R*)-**12a**	acetone	24	62, 8, 22, <1	7.8 : 1	95[f] / 34
6	(*R*)-**12a**	THF	36	74, 5, 19, <1	15 : 1	97[f] / 11
7	(*R*)-**13**	DMSO	68	80, 5, 9, 4	16 : 1	87[f] / 29
8	(*R*)-**14**	DMSO	192	29, 4, <1, 47	7.2 : 1	77[f] / 4
9	(*R*)-**12c**	DMSO	24	–[c]	–	–
10	L-proline	DMSO	6	13, 59, 19, <1	1 : 4.5	67[f] / 19

[a]Determined by the integration of the ^{1}H NMR signals in the presence of dibenzyl ether as an internal standard. [b]The combined yield of the *E*/*Z* isomers. [c]The products were not detected. [d]Determined by HPLC analysis with a chiral stationary phase. [e]The absolute configurations of the major enantiomers of *anti*-**18** for entry 1 was determined to be (1*S*,2*R*). [f]The absolute configurations of the major enantiomers of *anti*-**18** for entries 3–8 and 10 were determined to be (1*R*,2*S*). [g]The absolute configuration of the major enantiomer of *syn*-**19** was determined to be (1*S*,2*S*). n.d. = not determined.

Typical procedure of the intramolecular aldol reaction and determination of the absolute configuration of the aldol products for Table 1, Entry 1 and Table S1, Entry 1.

To a solution of dial **15** (43 mg, 0.38 mmol) in DMSO-d_6 (500 μL) was added cat. (*R*)-**11** (6.6 mg, 0.019 mmol). After being stirred for 96 h at 20 °C, the mixture was diluted with CH_2Cl_2 (5 mL). The diluted mixture was added ethyl (triphenylphosphoranylidene)acetate (327 mg, 0.94 mmol), and stirred for 24 h at rt. The mixture was added H_2O (10 mL) and extracted with AcOEt. The extract was washed with brine, dried over anhydrous Na_2SO_4, filtered, and evaporated. The crude residue was purified by column chromatography on silica gel (*n*-hexane/AcOEt, 5:1 to 3:1) to afford the fractions including *anti*-(*E*)-**18**, *anti*-(*Z*)-**18**, and *syn*-(*E*)-**19**. The yields of *anti*-(*E*)-**18** (29%), *anti*-(*Z*)-**18** (11%), and *syn*-(*E*)-**19** (52%) were determined by the integration of ^{1}H NMR signals in the presence of dibenzyl ether as an internal standard. The yield of *anti*-**18** (40%) depicted in Table 1, Entry 1 was combined yield of (*E*) and (*Z*)-isomers.

The enantiomeric excess of *anti*-(*E*)-**18** and *syn*-(*E*)-**19** was determined by HPLC analysis with chiral stationary phase as shown in pages S-13 and S-14, respectively.

For determination of absolute configuration of *anti*-**18** and *syn*-**19**, alternative set of the reaction in the presence of cat. (*R*)-**11** under the same conditions was performed as follows.

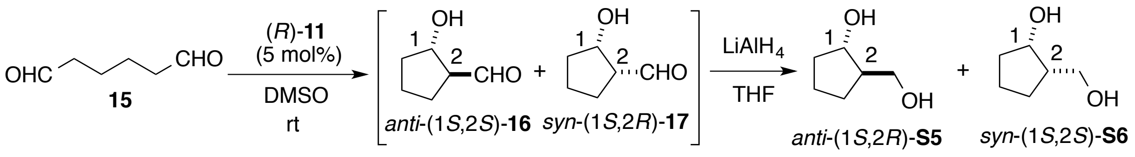

Experimental procedure: To a solution of dial **15** (100 mg, 0.88 mmol) in DMSO (860 μL) was added cat. (*R*)-**11** (15 mg, 44 μmol). After being stirred for 13 h at rt, the mixture was diluted with Et_2O and washed with brine. The organic layer was washed dried over anhydrous Na_2SO_4, filtered, and evaporated to give a residue. The residue in Et_2O was added to the suspension of $LiAlH_4$ (110 mg, 2.9 mmol) in THF at −15 °C, and stirred for 30 min at 0 °C. The reaction was quenched by successive addition of H_2O (110 μL), 15% aq. NaOH (110 μL), and H_2O (330 μL), and filtered. The filtrate was evaporated under reduced pressure to give a residue. The residue was purified by column chromatography on silica gel (*n*-hexane/AcOEt, 1:1) to give *anti*-**S5** (37 mg, 36%) and *syn*-**S6** (20 mg, 20%).

Absolute configurations of *anti*-**S5** and *syn*-**S6** were determined to be (1*S*,2*R*) and (1*S*,2*S*), respectively, by comparing the optical rotation to the literature data[3] as shown below.

anti-**S5**, $[\alpha]_D^{20}$ = +17 (*c* 0.5, MeOH). Lit.[3] $[\alpha]_D^{20}$ = +40 (*c* 3.0, MeOH) for (1*S*,2*R*)
syn-**S6**, $[\alpha]_D^{20}$ = +33 (*c* 0.5, MeOH). Lit.[3] $[\alpha]_D^{20}$ = +40 (*c* 1.0, MeOH) for (1*S*,2*S*)

Therefore, the absolute configurations of aldol adducts, *anti*-**16** and *syn*-**17**, in Table 1, Entry 1 were determined to be (1*S*,2*S*) and (1*S*,2*R*), respectively. According to the assignment, the absolute configurations of the corresponding olefin products *anti*-**18** and *syn*-**19** in Table 1, Entry 1, were determined to be (1*S*,2*R*) and (1*S*,2*S*), respectively.

The absolute configurations of *anti*-**18** and *syn*-**19** from Table 1, Entries 2–3 and Table S1, Entries 3–10 were determined to be (1*R*,2*S*) and (1*S*,2*S*), respectively, by comparison of the HPLC retention time with the corresponding compounds in Scheme 1 as shown in pages S-13 and S-14.

anti-(*E*)-(1*R*,2*S*)-**18**

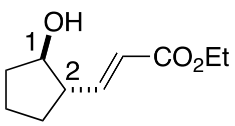

Colorless oil. 97% ee; $[\alpha]_D^{22}$ = −54 (*c* 1.1, $CHCl_3$). ^1H NMR (400 MHz, $CDCl_3$) δ 1.29 (t, *J* = 6.9 Hz, 3H), 1.42-2.10 (m, 6H), 2.44-2.58 (m, 1H), 3.94-4.07 (m, 1H), 4.19 (q, *J* = 6.9 Hz, 2H), 5.89 (d, *J* = 15.1 Hz, 1H), 6.90 (dd, *J* = 7.3 Hz, 15.1 Hz, 1H). ^{13}C NMR (100 MHz, $CDCl_3$) δ 21.5, 48.3, 48.4, 54.4, 62.6, 72.8, 127.6, 129.7, 132.9, 143.8. IR ($CHCl_3$) 3612, 3008, 2966, 2908, 2875, 1766, 1709, 1652 cm^{-1}. MS (EI) m/z 184 $(M)^+$. HRMS (EI) m/z calcd for $C_{10}H_{16}O_3$ $(M)^+$ 184.1099, found 184.1101.

anti-(*Z*)-(1*R*,2*S*)-**18**

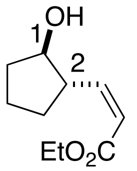

Colorless oil. $[\alpha]_D^{22}$ = +8.0 (*c* 0.6, $CHCl_3$, 98% ee). ^{1}H NMR (400 MHz, $CDCl_3$) δ 1.29 (t, *J* = 6.9 Hz, 3H), 1.33-2.12 (m, 6H), 3.40-3.53 (m, 1H), 3.87-4.01 (m, 1H), 4.10-4.27 (m, 2H), 5.87 (dd, *J* = 0.9 Hz, 11.5 Hz, 1H), 6.15 (dd, *J* = 9.2 Hz, 11.5 Hz, 1H). ^{13}C NMR (150 MHz, $CDCl_3$) δ 14.2, 22.5, 31.7, 34.7, 48.3, 60.4, 80.0, 120.7, 152.8, 167.5. IR ($CHCl_3$) 2962, 1703, 1229, 1199 cm^{-1}. MS (EI) m/z (rel intensity) 184 $(M)^+$. HRMS (EI) m/z calcd for $C_{10}H_{16}O_3$ $(M)^+$ 184.1099, found 184.1105.

syn-(*E*)-(1*S*,2*S*)-**19**

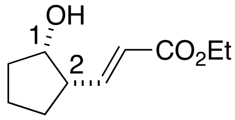

Colorless oil. $[\alpha]_D^{22}$ = −25 (*c* 0.2, $CHCl_3$, 92% ee). ^{1}H NMR (400 MHz, $CDCl_3$) δ 1.29 (t, *J* = 6.9 Hz, 3H), 1.57-2.01 (m, 6H), 2.49-2.65 (m, 1H), 4.18 (q, *J* = 6.9 Hz, 2H), 4.23-4.33 (m, 1H), 5.90 (d, *J* = 15.6 Hz, 1H), 7.11 (dd, *J* = 8.2 Hz, 15.6 Hz, 1H). ^{13}C NMR (100 MHz, $CDCl_3$) δ 14.3, 22.1, 27.9, 34.7, 48.7, 60.3, 75.8, 122.6, 148.4, 166.5. IR ($CHCl_3$) 3612, 3017, 2967, 2875, 1709, 1652 cm^{-1}. MS (EI) m/z 184 $(M)^+$. HRMS (EI) m/z calcd for $C_{10}H_{16}O_3$ $(M)^+$ 184.1099, found 184.1098.

(*E*)-Ethyl 3-cyclopentenyl acrylate (**S3**)

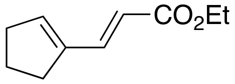

Colorless oil. ^{1}H NMR (400 MHz, $CDCl_3$) δ 1.30 (t, *J* = 6.9 Hz, 3H), 1.90-2.03 (m, 2H), 2.37-2.56 (m, 4H), 4.21 (q, *J* = 6.9 Hz, 2H), 5.74 (d, *J* = 15.6 Hz, 1Hz), 6.10-6.21 (m, 1H), 7.50 (d, *J* = 15.6 Hz, 1H). ^{13}C NMR (100 MHz, $CDCl_3$) δ 14.3, 23.0, 30.7, 33.4, 60.2, 118.2, 140.4, 140.8, 141.4, 167.5. IR ($CHCl_3$) 2982, 2960, 1701, 1630, 1308, 1276, 1232, 1176, 1039 cm^{-1}. MS (FAB) m/z 167 $(M+H)^+$. HRMS (FAB) m/z calcd for $C_{10}H_{15}O_2$ $(M+H)^+$ 167.1072, found 167.1067.

Olefine **S4**

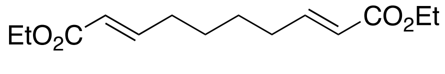

Colorless oil. ^{1}H NMR (400 MHz, $CDCl_3$) δ 1.22 (t, *J* = 6.8 Hz, 6H), 1.39-1.47 (m, 4H), 2.10-2.30 (m, 4H), 4.11 (q, *J* = 6.8 Hz, 4H), 5.74 (dt, *J* = 1.8, 16.0 Hz, 2H), 6.87 (dt, *J* = 6.8, 16.0 Hz, 2H). ^{13}C NMR (100 MHz, $CDCl_3$) δ 14.2, 27.4, 31.8, 60.1, 121.6, 148.6, 166.6. IR ($CHCl_3$) 3023, 2985, 2936, 2862, 1711, 1654 cm^{-1}. MS (FAB) m/z 255 $(M+H)^+$. HRMS (FAB) m/z calcd for $C_{14}H_{23}O_4$ $(M+H)^+$ 255.1596, found 255.1594.

HPLC analysis of *anti*-(*E*)-18

Conditions: Chiralcel OD-H (0.46 x 25 cm), *n*-hexane–*i*-PrOH (95 : 5), 0.5 mL/min, 254 nm, t_R = 23.1 min (1*R*,2*S*), 36.4 min (1*S*,2*R*).

Racemic anti-(*E*)-**18**

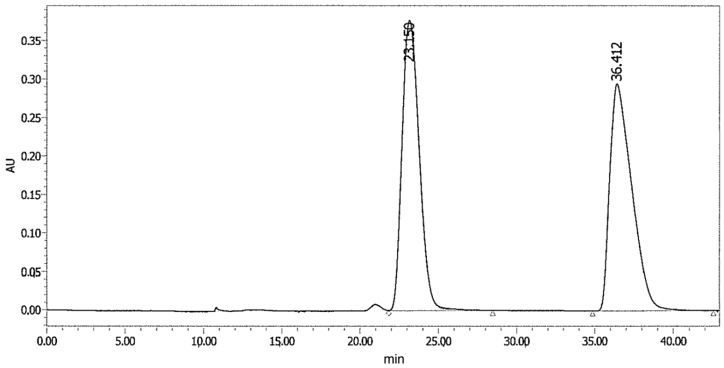

	Retention time (min)	Peak area (%)
1	23.15	50.1
2	36.41	49.9

Chromatogram of optically active anti-(*E*)-**18** (50% ee) for Table 1, Entry 1 and Table S1, Entry 1.

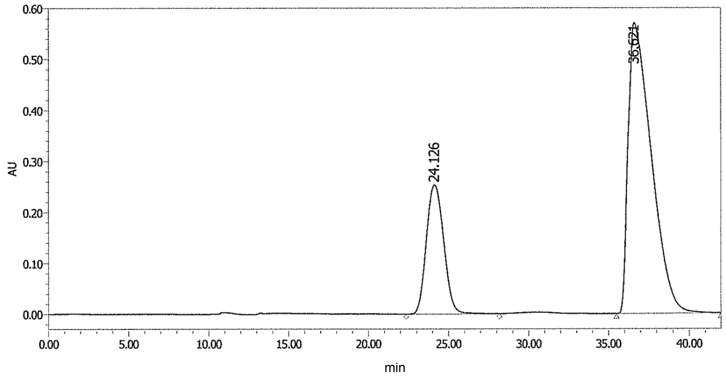

	Retention time (min)	Peak area (%)
1	24.13	25.2
2	36.62	74.8

Typical chromatogram of optically active anti-(*E*)-**18** (95% ee) (Table S1, Entry 4)

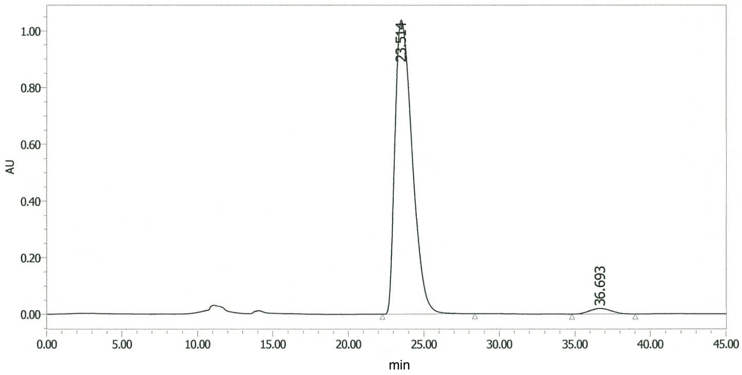

	Retention time (min)	Peak area (%)
1	23.51	97.7
2	36.41	2.3

HPLC analysis of *syn*-(*E*)-19

Conditions: Chiralcel OD-H (0.46 x 25 cm), *n*-hexane–*i*-PrOH (95 : 5), 1.0 mL/min, 254 nm, t_R = 18.2 min (1*R*,2*R*), 26.8 min (1*S*,2*S*).

Racemic syn-(*E*)-**19**

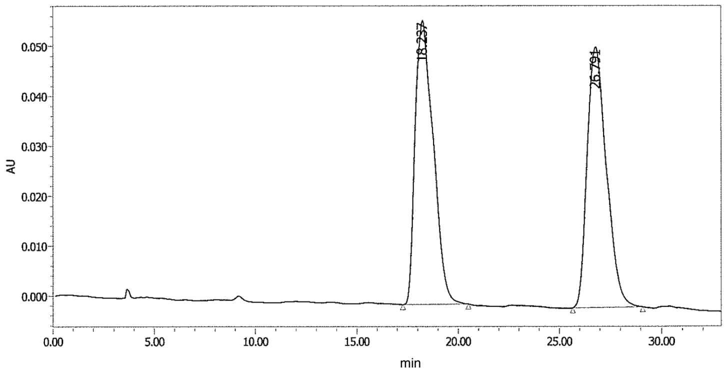

	Retention time (min)	Peak area (%)
1	18.24	50.6
2	26.79	49.4

Chromatogram of optically active syn-(*E*)-**19** (93% ee) for Table 1, Entry 1 and Table S1, Entry 1.

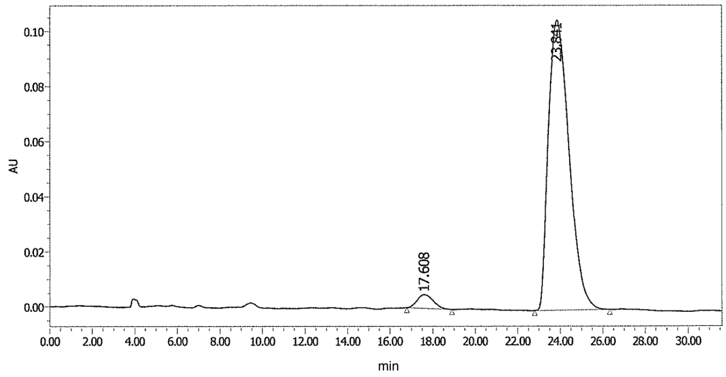

	Retention time (min)	Peak area (%)
1	17.61	3.6
2	23.84	96.4

Typical chromatogram of optically active syn-(*E*)-**19** (37% ee) (Table S1, Entry 4)

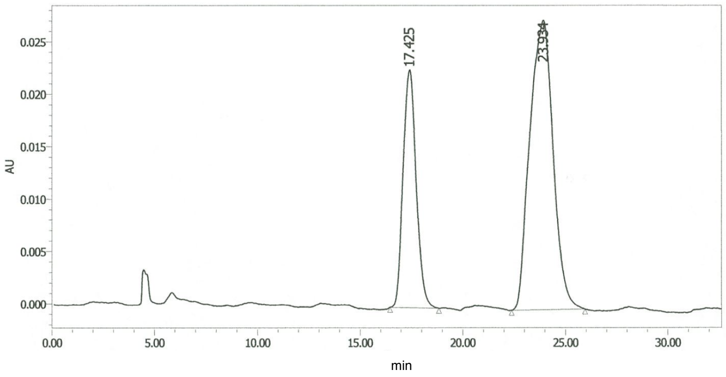

	Retention time (min)	Peak area (%)
1	17.43	31.6
2	23.93	68.4

Survey of the reaction conditions for intramolecular cross-aldol reaction of *N*-Ts dial (1a).

The reaction in the presence of cat. (*R*)-**12b** gave the dehydrated **9** as the major product (Table S2, Entry 1). To clarify the catalyst property, we tested the reaction with (*R*)-BINAM (**S7**), and found the reaction gave *anti*-**7** and *syn*-**8** as major isomers (Entry 2). The negligible ee value of *syn*-**8** may suggest that the products were produced through the background reaction (Table 2, Entry 7). This indicates that the acidic moiety of the catalyst for activation of formyl group is crucial to yield **5a** regioselectively. L-Histidine, which was successfully employed for intermolecular cross-aldol reaction with α-branched substrate[4], was also tested (Entry 3). This catalyst gave *anti*-**7** and *syn*-**8** as major regioisomers. However, no ee value in *syn*-**8** might suggest this catalyst did not overcome the background reaction.

The product ratio by cat. (*dl*)-**11** was also examined for the reductive amination experiment (Figure 4C) as shown in Entry 4. The regioselectivity, (**5a**+**6**+**9**) : (**7**+**8**) = 7.4 : 1, was found to be similar to that by cat. (*R*)-**13** {(**5a**+**6**+**9**) : (**7**+**8**) = 8.0 : 1} (Table 2, Entry 1).

Table S2. Intramolecular cross-aldol reaction of **1a**.

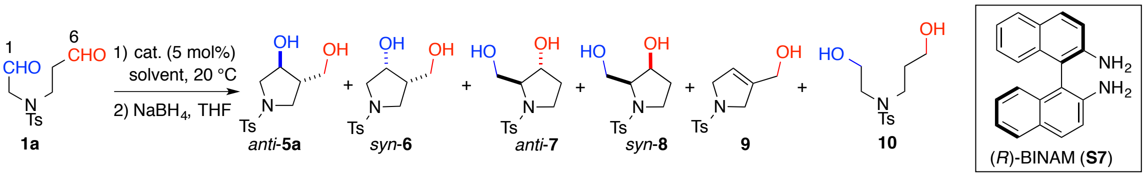

entry	catalyst	*t* (h)	yield (%)[a] 5a[b], 6[c], 7[c], 8[c], 9, 10	regioselectivity (5a+6+9) : (7+8)	d.r. 5a : 6	d.r. 7 : 8	ee (%) 5a[e]	ee (%) 8[e]
1	(*R*)-**12b**	72	19, 2, 11[d], 4, 57, 0	5.2 : 1	8.0 : 1	2.8 : 1	82	34
2	(*R*)-**S7**	72	3, 7, 21, 60, 0, 6	1 : 8.0	1 : 2.3	1 : 2.9	n.d.	10
3	L-histidine	72	7, 12, 17, 40, 2, 14	1 : 3.0	1 : 1.6	1 : 2.4	n.d.	0
4	(*dl*)-**11**	24	42, 18, 5, 5, 14, 0	7.4 : 1	2.3 : 1	1.0 : 1	–	–

[a]Determined by the integration of the ^{1}H NMR signals in the presence of dibenzyl ether as an internal standard. [b]The absolute configuration of the major enantiomer of *anti*-**5a** for entry 1 was determined to be (3*S*,4*S*). [c]The relative stereochemistry of all isomers were determined. The absolute configurations of them are tentative based on the assumption that both products were generated from the same enamine geometry for *anti*-**5a**. [d]18% ee was observed by HPLC analysis with a chiral stationary phase. [e]Determined by HPLC analysis with a chiral stationary phase.

Typical procedure of the intramolecular cross-aldol reaction of 1a in the presence of cat. (*R*)-13 (Table 2, Entry 1)

The reaction was monitored by ^{1}H NMR. To a solution of dial **1a** (28 mg, 0.10 mmol) in DMSO-d_6 (500 μL) was added cat. (*R*)-**13** (2.3 mg, 5.0 μmol). The reaction mixture was stored for 72 h at 20 °C with monitoring ^{1}H NMR from time to time. To the diluted reaction mixture with THF (5 mL) was added $NaBH_4$ (12 mg, 0.31 mmol), and stirred for 24 h at rt. The mixture was added H_2O and extracted with AcOEt. The extract was washed with brine, dried over anhydrous Na_2SO_4, filtered, and evaporated. The crude residues were purified by prep. TLC on silica gel ($CHCl_3$/MeOH, 8:1) to afford *anti*-**5a**, *anti*-**7**, and the fraction including *syn*-**6** and **8**. The yields of these compounds, *anti*-**1a** (59%), *syn*-**6** (5%), *anti*-**7** (8%), and diol **10** (25%), were determined by the integration of ^{1}H NMR signals in the presence of dibenzyl ether as an internal standard.

Procedure of the intramolecular cross-aldol reaction of 1a in the presence of L-proline (Table 2, Entry 6)

The reaction was monitored by ^{1}H NMR. To a solution of dial **1a** (28 mg, 0.10 mmol) in DMSO-d_6 (500 μL) was added L-proline (0.6 mg, 5.0 μmol). The reaction mixture was mixed and stored for 24 h at 20 °C with monitoring ^{1}H NMR. To the diluted reaction mixture with THF (5 mL) was added $NaBH_4$ (12 mg, 0.31 mmol), and stirred for 24 h at rt. The mixture was added H_2O and extracted with AcOEt. The extract was washed with brine, dried over anhydrous Na_2SO_4, filtered, and evaporated. The crude residues were purified by prep. TLC on silica gel ($CHCl_3$/MeOH, 8:1) to afford *anti*-**5a** and *syn*-**8**, and the two parts of the fraction including *anti*-**7** and dehydrated **9**, as well as *syn*-**6** and **8**. The yields of these compounds, *anti*-**5a** (9%), *syn*-**6** (31%), *anti*-**7** (5%), *syn*-**8** (17%), dehydrated **9** (2%), and diol **10** (20%), were determined by the integration of ^{1}H NMR signals in the presence of dibenzyl ether as an internal standard.

Characterization and determination of the stereochemistries of the aldol products for Table 2

anti-(3*S*,4*S*)-**5a**

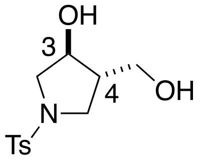

Colorless plates ($CHCl_3$). M.p. 87 °C. $[\alpha]_D^{22}$ = −11 (*c* 1.3, $CHCl_3$, 89% ee). ^{1}H NMR (600 MHz, $CDCl_3$) δ 2.19-2.27 (m, 1H), 2.44 (s, 3H), 3.00-3.08 (m, 1H), 3.08-3.14 (m, 1H), 3.41-3.64 (m, 4H), 4.16 (q, *J* = 5.5 Hz, 1H), 7.33 (d, *J* = 8.2 Hz, 2H), 7.70 (d, *J* = 8.2 Hz, 2H). ^{13}C NMR (150 MHz, $CDCl_3$) δ 21.6, 48.3, 48.4, 54.4, 62.6, 72.9, 127.6, 129.7, 133.0, 143.8. IR ($CHCl_3$) 3672, 3615, 3563, 3026, 1598, 1555, 1539 cm^{-1}. MS (FAB) m/z 272 $(M+H)^+$. HRMS (FAB) m/z calcd for $C_{12}H_{17}N_1O_4S_1$ $(M+H)^+$ 272.0957, found 272.0969.

The enantiomeric excess of *anti*-**5a** was determined by HPLC analysis with chiral stationary phase as shown below.

HPLC conditions: Chiralpak IA (0.46 x 25 cm), hexane–*i*-PrOH (80 : 20), 1.0 mL/min, 254 nm, t_R = 11.8 min (3*R*,4*R*), 16.4 min (3*S*,4*S*).

Racemic anti-**5a**

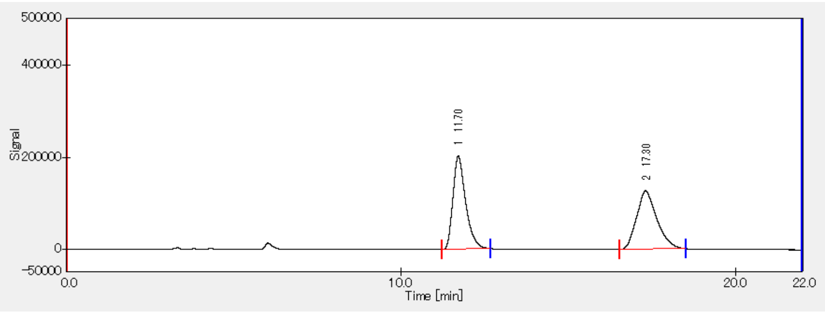

	Retention time (min)	Peak area (%)
1	11.70	50.1
2	17.30	49.9

Typical HPLC chromatogram of optically active anti-**5a** (90% ee) derived from *anti*-**5d** for Table 3.

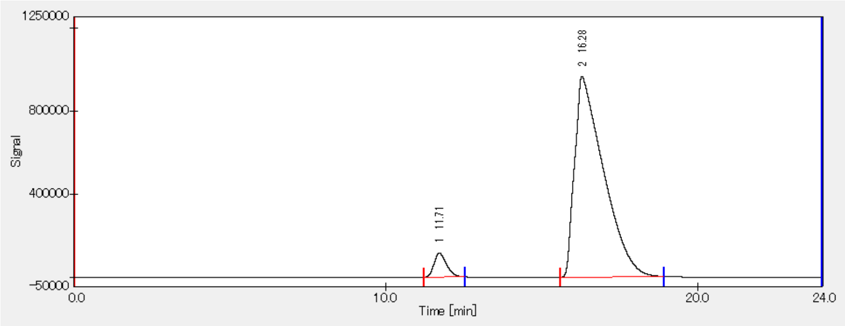

	Retention time (min)	Peak area (%)
1	11.71	5.0
2	16.28	95.0

The absolute configuration of *anti*-**5a** was determined to be (3*S*,4*S*) by transformation to *anti*-**5d** and comparison of its optical rotation to the literature data[5] as shown below.

anti-**5d** derived from *anti*-**5a** (89% ee), $[\alpha]_D^{22}$ = –13 (*c* 0.3, MeOH). Lit.[5] $[\alpha]_D^{21}$ = +15.9 (*c* 1.09, MeOH) for (3*R*,4*R*).

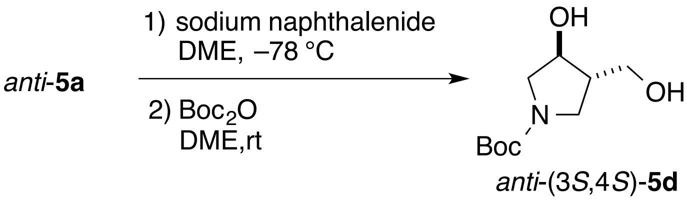

Experimental procedure: To a solution of naphthalene (393 mg, 0.375 mmol) in DME (5.0 mL) was added Na (300 mg, 18.7 μmol) at rt under Ar atmosphere. After being stirred for 40 min at rt, the solution of *anti*-**5a** (35 mg, 0.13 mmol) in DME (5 mL) was added to the sodium naphthalenide solution in DME until green color remained and stirred for 15 min at –78 °C, then stirred for 30 min at rt. The mixture was added Boc_2O (34 mg, 0.155 mmol) solution in DME (2.0 mL) at rt, and stirred for 9 h at the same temperature. The mixture was quenched with H_2O and extracted with AcOEt. The extract was washed with brine, dried over anhydrous Na_2SO_4, filtered, and evaporated. The crude residue was purified by column chromatography on silica gel ($CHCl_3$/MeOH, 8:1), and further purified by prep. TLC (SiO_2, $CHCl_3$/MeOH, 8:1) to afford *anti*-**5d** (8.8 mg, 31%).

syn-**6**

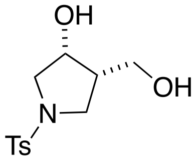

The absolute configuration was not determined. The stereostructure written in Table 2 is tentative based on the assumption that *syn*-**6** was generated from the same enamine geometry for *anti*-(3*S*,4*S*)-**5a** in the presence of cat. (*R*)-**13**.

Colorless needles ($CHCl_3$). M.p. 114-115 °C. ^{1}H NMR (600 MHz, $CDCl_3$) δ 2.21-2.35 (m, 1H), 2.43 (s, 3H), 3.23 (t, *J*=9.7 Hz, 1H), 3.30-3.37 (m, 1H), 3.41-3.52 (m, 2H), 3.76 (q, *J* = 5.5 Hz, 1H), 3.84 (q, *J* = 5.5 Hz, 1H), 4.41-4.48 (m, 1H), 7.33 (d, *J* = 8.2 Hz, 2H), 7.75 (d, *J* = 8.2 Hz, 2H). ^{13}C NMR (150 MHz, $CDCl_3$) δ 21.6, 44.5, 47.4, 56.4, 60.3, 72.3, 127.6, 129.7, 133.5, 143.6. IR ($CHCl_3$) 3621, 3028, 1730, 1343, 1160, 1031 cm^{-1}. MS (FAB) m/z 272 $(M+H)^+$. HRMS (FAB) m/z calcd for $C_{12}H_{17}N_1O_4S_1$ $(M+H)^+$ 272.0957, found 272.0955.

The enantiomeric excess of *syn*-**6** was determined by HPLC analysis with chiral stationary phase as shown below.

HPLC conditions: Chiralpak ID (0.46 x 25 cm), hexane–*i*-PrOH (80 : 20), 1.0 mL/min, 254 nm

Racemic syn-**6**

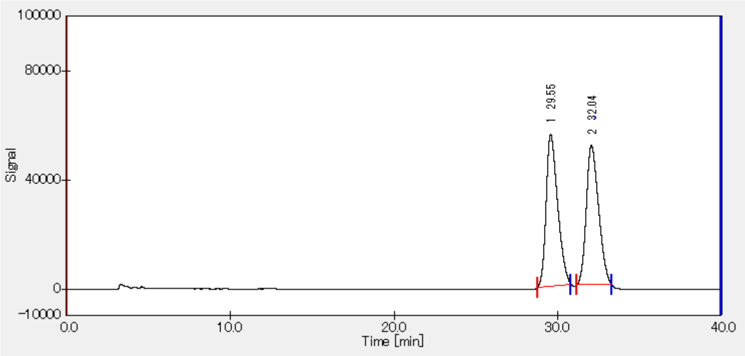

	Retention time (min)	Peak area (%)
1	29.55	50.3
2	32.04	49.7

Typical HPLC chromatogram of optically active syn-**6** (>99% ee) for Table 2, Entry 6.

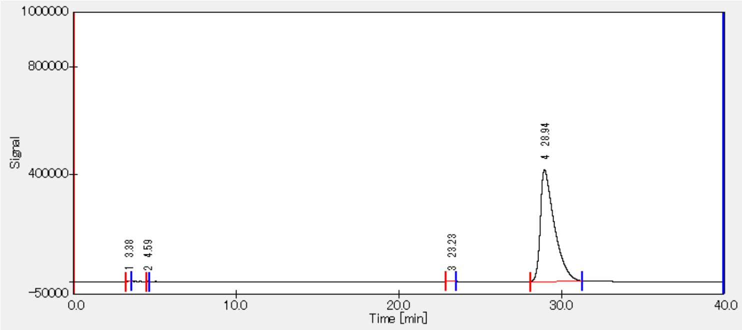

	Retention time (min)	Peak area (%)
1	28.94	> 99

anti-**7**

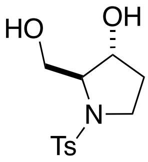

Colorless needles ($CHCl_3$). M.p. 108 °C. The absolute configuration was not determined. The stereostructure written in Table 2 is tentative based on the assumption that *anti*-**7** was generated from the same enamine geometry for *anti*-(3*S*,4*S*)-**5a** in the presence of cat. (*R*)-**13**.

^{1}H NMR (600 MHz, $CDCl_3$) δ 1.65-1.70 (m, 1H), 2.00-2.1 (m, 1H), 2.42 (s, 3H), 3.26-3.34 (m, 1H), 3.38-3.44 (m, 1H) 3.52-3.59 (m, 1H), 3.72-3.79 (m, 2H), 4.22-4.26 (m, 1H), 7.33 (d, J = 8.2 Hz, 2H), 7.75 (d, J = 8.2 Hz, 2H). ^{13}C NMR (150 MHz, $CDCl_3$) δ 21.6, 32.7, 47.3, 64.3, 69.6, 73.9, 127.8, 129.7, 133.1, 143.9. IR ($CHCl_3$) 3490, 3029, 2950, 1598, 1338, 1198 cm^{-1}. MS (FAB) m/z 272 $(M+H)^+$. HRMS (FAB) m/z calcd for $C_{12}H_{17}N_1O_4S_1$ $(M+H)^+$ 272.0957, found 272.0959.

The enantiomeric excess of *anti*-**7** was determined by HPLC analysis with chiral stationary phase as shown below.

HPLC conditions: Chiralpak IA (0.46 x 25 cm), hexane–*i*-PrOH (80 : 20), 1.0 mL/min, 254 nm

Racemic anti-**7**

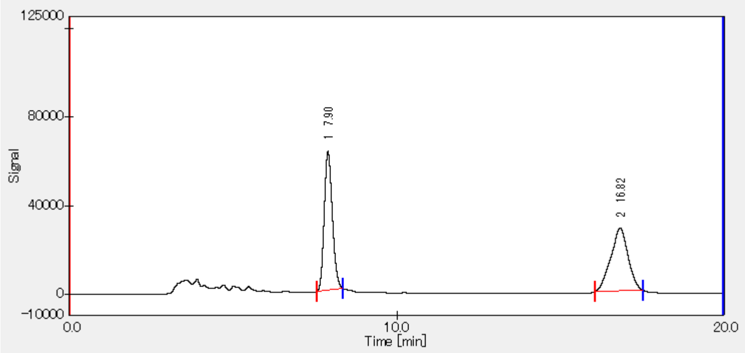

	Retention time (min)	Peak area (%)
1	7.90	50.9
2	16.82	49.1

Typical HPLC chromatogram of optically active anti-**7** (74% ee) for Table 2, Entry 1.

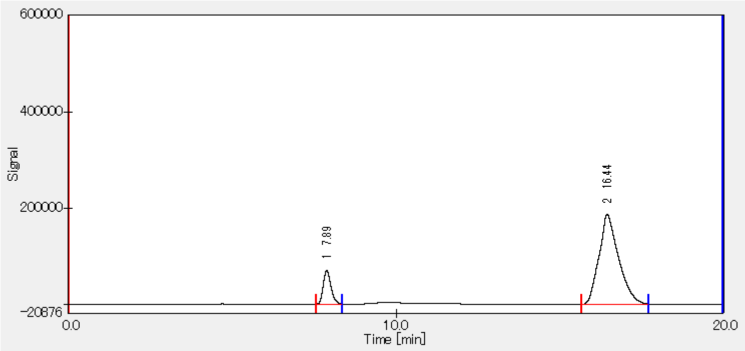

	Retention time (min)	Peak area (%)
1	7.89	13.1
2	16.44	86.9

syn-(2*R*,3*R*)-**8**

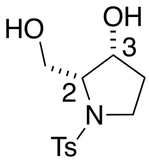

The relative and absolute configuration of the product by L-proline catalyst (Table 2, Entry 6) was determined as written in the structure. The experimental detail was depicted below.

^{1}H NMR (600 MHz, $CDCl_3$) δ 1.56-1.69 (m, 1H), 1.79-1.88 (m, 1H), 2.44 (s, 3H), 3.24-3.34 (m, 1H) Colorless plates ($CHCl_3$). M.p. 96-97 °C. 3.42-3.50 (m, 1H), 3.59-3.68 (m, 1H), 4.02 (q, *J* = 5.5 Hz, 1H), 4.13 (q, *J* = 5.5 Hz, 1H), 4.22-4.29 (m, 1H), 7.34 (d, *J* = 8.2 Hz, 2H), 7.72 (d, *J* = 8.2 Hz, 2H). ^{13}C NMR ($CDCl_3$, 150 MHz) δ 21.6, 33.3, 47.0, 62.6, 62.9, 73.5, 127.6, 129.9, 133.5, 144.0. IR ($CHCl_3$) 3503, 3028, 1597, 1402, 1341, 913 cm^{-1}. MS (FAB) m/z 272 $(M+H)^+$. HRMS (FAB) m/z calcd for $C_{12}H_{17}N_1O_4S_1$ $(M+H)^+$ 272.0957, found 272.0956.

The enantiomeric excess of *syn*-**8** was determined by HPLC analysis with chiral stationary phase as shown below.

HPLC conditions: Chiralpak IA (0.46 x 25 cm), hexane–*i*-PrOH (80 : 20), 1.0 mL/min, 254 nm

*Racemic syn-***8**

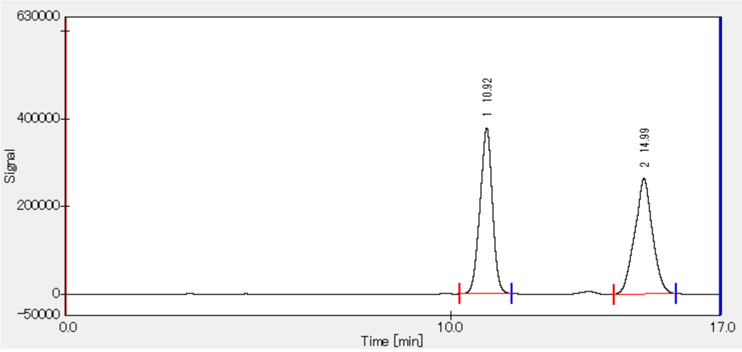

	Retention time (min)	Peak area (%)
1	10.92	50.1
2	14.99	50.0

*Typical HPLC chromatogram of optically active syn-***8** (53% ee) for Table 2, Entry 6.

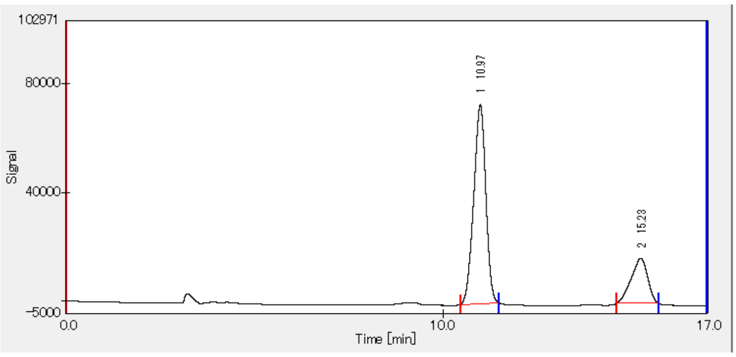

	Retention time (min)	Peak area (%)
1	10.97	76.4
2	15.23	23.6

The relative and absolute stereochemistries of the major enantiomer of *syn*-**8** {$[\alpha]_D^{22}$ = −15 (*c* 0.8, $CHCl_3$)} from Table 2, Entry 6 were determined to be *syn* and (2*R*,3*R*), respectively, by identification of NMR data and comparison of the optical rotation with *syn*-**8** {$[\alpha]_D^{22}$ = −68 (*c* 1.3, $CHCl_3$)} prepared from *cis*-3-hyrdoxy-L-proline[6] as shown below.

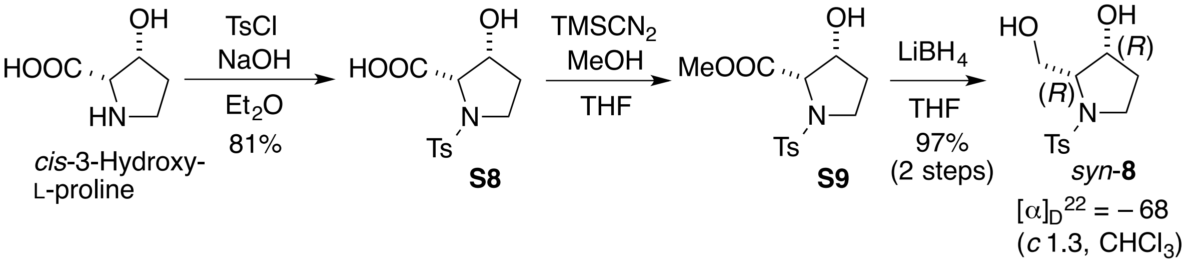

Experimental procedure: To a solution of *cis*-3-hydroxy-L-proline[4] (1.00 g, 7.60 mmol) in 2 N aq. NaOH (10 mL) was added TsCl (1.50 g, 7.80 mmol) at rt. After being stirred for 6 h at rt, the H_2O layer was separated, acidified by 6 N aq. HCl to pH 2.0, and extracted with AcOEt. The extract was washed with brine, dried over anhydrous Na_2SO_4, filtered, and evaporated to afford **S8**[7] (1.83 g, 81%).

To a solution of **S8**[7] (500 mg, 1.75 mmol) in MeOH–THF (8 mL, 3/5) was added trimethylsilyldiazomethane (2.0 M solution in Et_2O, 1.32 mL, 2.63 mmol) at rt. After being stirred for 30 min at rt, AcOH was added until yellow color of the mixture disappeared. The mixture was concentrated in vacuo, and then diluted with AcOEt. The organic layer was washed with sat. aq. $NaHCO_3$, brine, dried over anhydrous Na_2SO_4, filtered, and evaporated to give the crude mixture (608 mg) containing **S9**[7] as a major

product.

To a solution of the part of the mixture (145 mg) containing **S9**[7] in THF (4 mL) was added $LiBH_4$ (3.0 M solution in Et_2O, 0.48 mL, 1.44 mmol) at 0 °C under Ar atmosphere. After being stirred for 13 h at rt, the mixture was poured into H2O, and extracted with AcOEt. The extract was washed with brine, dried over anhydrous Na_2SO_4, filtered, and evaporated. The crude residue was purified by column chromatography on silica gel ($CHCl_3$/MeOH, 8:1) to afford *syn*-(2*R*,3*R*)-**8** (106 mg, 97% for 2 steps).

Dehydrated **9**

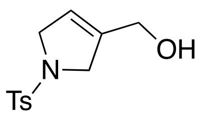

Colorless needles ($CHCl_3$). M.p. 89 °C. ^{1}H NMR (600 MHz, $CDCl_3$) δ 2.42 (s, 3H), 4.06-4.19 (m, 6H), 5.53-5.58 (m, 1H), 7.32 (d, J = 8.2 Hz, 2H), 7.72 (d, J = 8.2 Hz, 2H). ^{13}C NMR (150 MHz, $CDCl_3$) δ 21.5, 54.5, 54.9, 59.6, 120.0, 127.4, 129.8, 134.0, 139.2, 143.6. IR ($CHCl_3$) 2982, 2960, 1701, 1630, 1308, 1276, 1232, 1176, 1039 cm^{-1}. MS (FAB) m/z 254 $(M+H)^+$. HRMS (FAB) m/z calcd for $C_{12}H_{16}N_1O_3S_1$ $(M+H)^+$ 254.0851, found 254.0850.

Diol **10**

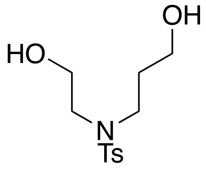

Colorless needles ($CHCl_3$). M.p. 55-57 °C. ^{1}H NMR (400 MHz, $CDCl_3$) δ 1.76-1.89 (m, 2H), 2.41 (s, 3H), 3.16-3.37 (m, 4H), 3.71-3.92 (m, 4H), 7.30 (d, J = 8.2 Hz, 2H), 7.68 (d, J = 8.2 Hz, 2H). ^{13}C NMR (100 MHz, $CDCl_3$) δ 21.4, 31.3, 47.4, 52.0, 59.3, 61.5, 127.2, 129.8, 135.3, 143.6. IR ($CHCl_3$) 3671, 3623, 3029, 3010, 2954, 1599, 1543 cm^{-1}. MS (FAB) m/z 274 $(M+H)^+$. HRMS (FAB) m/z calcd for $C_{12}H_{19}N_1O_4S_1$ $(M+H)^+$ 274.1000, found 274.1022.

Characterization of the aldol products for Table 3

anti-**5b**

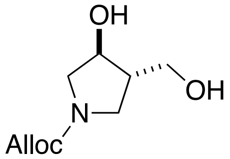

Pale yellow oil. $[\alpha]_D^{22}$ = −21 (*c* 0.5, $CHCl_3$, 90% ee). ^{1}H NMR (600 MHz, $CDCl_3$) δ 2.30-2.40 (m, 1H), 3.15-3.25 (m, 1H), 3.26-3.38 (m, 1H), 3.60-3.80 (m, 4H), 4.28-4.35 (m, 1H), 4.58 (d, J = 5.5 Hz, 2H), 5.17-5.24 (m, 1H), 5.27-5.34 (m, 1H), 5.88-5.98 (m, 1H). ^{13}C NMR (150 MHz, $CDCl_3$, rotamers) δ 46.2, 46.5, 47.4, 48.1, 52.4, 52.8, 63.1, 63.3, 65.9, 72.6, 73.6, 117.37, 117.42, 133.0, 154.9. IR ($CHCl_3$) 1689, 1216, 767, 721 cm^{-1}. MS (FAB) m/z 202 $(M+H)^+$. MS (FAB) m/z 200 $(M-H)^-$. HRMS (FAB) m/z calcd for $C_9H_{14}N_1O_4$ $(M-H)^-$ 200.0923, found 200.0923.

By transformation of *anti*-**5b** to *anti*-**5a** and its HPLC analysis with chiral stationary phase, the absolute configuration and the enantiomeric excess of *anti*-**5b** were determined to be (3*S*,4*S*) and 90% ee, respectively.

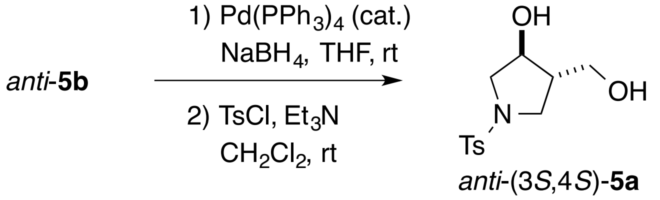

Experimental procedure: To a solution of *anti*-**5b** (15 mg, 0.075 mmol) and $Pd(PPh_3)_4$ (8.7 mg, 0.0075 mmol) in THF (5.0 mL) was added $NaBH_4$ (5.7 mg, 0.15 mmol) at rt under Ar atmosphere. After being stirred for 4 h at rt, the solution was diluted with CH_2Cl_2 (5.0 mL) and Et_3N (0.02 mL, 0.15 mmol) and cooled to 0 °C. To a mixture was added TsCl (14.3 mg, 0.075 mmol) solution in CH_2Cl_2 (2.0 mL), and warmed to rt, then stirred for 6 h at the same temperature. The mixture was quenched with H_2O and extracted with AcOEt. The extract was washed with brine, dried over anhydrous Na_2SO_4, filtered, and evaporated. The crude residue was purified by prep. TLC (SiO_2, $CHCl_3$/MeOH, 8:1) to afford *anti*-**5a** (13.4 mg, 67%) as yellow amorphous.

anti-**5c**[8]

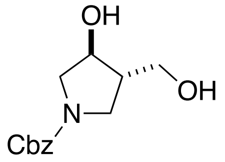

Spectroscopic and physical data was reported in the literature.[8]

By transformation of *anti*-**5c** to *anti*-**5a** and its HPLC analysis with chiral stationary phase, the absolute configuration and the enantiomeric excess of *anti*-**5c** were determined to be (3*S*,4*S*) and 90% ee, respectively.

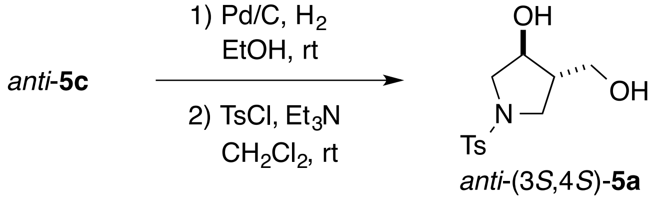

Experimental procedure: To a suspension of Pd/C (10 wt%, 15 mg) in EtOH (10 mL) was added *anti*-**5c** (17mg, 0.068 mmol) solution in EtOH (2.0 mL). After being stirred at rt for 5 h under H_2 atomosphere, the mixture was filtered and the filtrate was evaporated to give a residue.

To a solution of the residue in CH_2Cl_2 (3.0 mL) were added Et_3N (0.50 mL, 0.204 mmol) and TsCl (15 mg, 0.081 mmol) at 0 ℃ under Ar atmosphere. After being stirred for 24 h at rt, the reaction was quenched with H_2O, and extracted with AcOEt. The extract was washed with brine, dried over anhydrous Na_2SO_4, filtered, and evaporated to give the residue. The residue was purified by prep. TLC (SiO_2, $CHCl_3$/MeOH, 8:1) to afford *anti*-**5a** (7.4 mg, 40%) as yellow amorphous.

anti-**5d**[5]

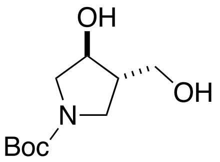

Spectroscopic and physical data was reported in the literature.[5]

By transformation of *anti*-**5d** to *anti*-**5a** and its HPLC analysis with chiral stationary phase, the absolute configuration and the enantiomeric excess of *anti*-**5d** were determined to be (3*S*,4*S*) and 90% ee, respectively.

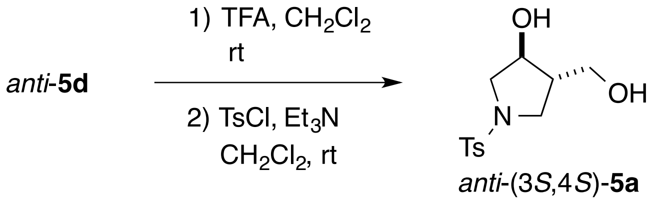

Experimental procedure: To a solution of *anti*-**5d** (11 mg, 0.051 mmol) in CH_2Cl_2 (1.0 mL) was added TFA (200 μL) at rt. After being stirred for 2 h at rt, the mixture was evaporated *in vacuo* to give a residue.
To a solution of the residue in CH_2Cl_2 (2.0 mL) were added Et_3N (0.70 mL, 5.0 mmol) and TsCl (11 mg, 0.056 mmol) at 0 ℃ under Ar atmosphere. After being stirred for 4 h at rt, the reaction was quenched with aq. 1% (v/v) HCl, and extracted with AcOEt. The extract was washed with aq. 1% (v/v) HCl and brine, dried over anhydrous Na_2SO_4, filtered, and evaporated to give the residue. The residue was purified by column chromatography on silica gel ($CHCl_3$/MeOH, 8:1) to afford *anti*-**5a** (40 mg, 31%) as yellow amorphous.

Preparation of *N*-containing dials for Tables 2 and 3

Dial **1a**

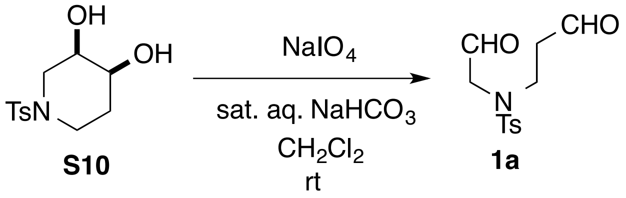

To a solution of diol **S10**[9] (20 mg, 74 μmol) in CH_2Cl_2 (1.0 mL) were added sat. aq. $NaHCO_3$ (15 μL) and $NaIO_4$ (40 mg, 0.19 mmol). After being stirred for 3 h at rt, $NaIO_4$ (40 mg, 0.19 mmol) was further added, and the mixture was stirred for 1 h at rt to complete the reaction. The mixture was diluted with Et_2O (20 mL) and washed with H_2O and brine. The organic layer was dried over anhydrous Na_2SO_4, filtered, and evaporated to give a residue. The residue was employed for asymmetric cross-aldol reaction without further purification.
Pale yellow oil. ^1H NMR (600 MHz, $CDCl_3$) δ 2.44 (s, 3H), 2.92 (t, J = 6.2 Hz, 2H), 3.45 (t, J = 6.2 Hz, 2H), 4.01 (s, 2H), 7.34 (d, J = 8.3 Hz, 2H), 7.69 (d, J = 8.3 Hz, 2H), 9.54 (s, 1H), 9.77 (s, 1H). ^{13}C NMR (150 MHz, $CDCl_3$) δ 21.5, 43.3, 44.2, 58.7, 127.4, 129.9, 135.2, 144.2, 197.4, 200.1. IR ($CHCl_3$) 2982, 2960, 1701, 1630, 1308, 1276, 1232, 1176, 1039 cm^{-1}. MS (FAB) m/z 270 $(M+H)^+$. HRMS (FAB) m/z calcd for $C_{12}H_{16}N_1O_4S_1$ $(M+H)^+$ 270.0800, found 270.0800.

Dial **1b**

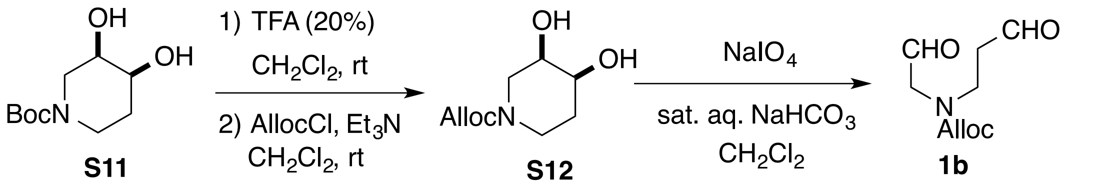

To a solution of diol **S11**[10] (300 mg, 1.38 mmol) in CH_2Cl_2 (5.0 mL) was added TFA (1.0 mL) at rt. After being stirred for 4 h at rt, the mixture was evaporated *in vacuo* to give a residue. To a solution of the residue and Et_3N (1.5 mL, 10.8 mmol) in CH_2Cl_2 (5.0 mL) was added AllocCl (183 mg, 1.52 mmol) at 0 °C. After being stirred for 24 h at rt, the reaction was quenched with H_2O and extracted with AcOEt. The extract was washed with brine, dried over anhydrous Na_2SO_4, filtered, and evaporated. The crude residue was purified by column chromatography on silica gel ($CHCl_3$/MeOH, 10:1) to afford **S12** (191 mg, 69%).

Dial **1b** was prepared from diol **S12** according to the procedure for dial **1a**.

Diol **S12**

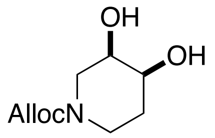

Colorless needles ($CHCl_3$). M.p. 59 °C. ^{1}H NMR (400 MHz, $CDCl_3$) δ 1.63-1.91 (m, 2H), 3.26-.3.95 (m, 6H), 4.53-4.66 (m, 2H), 5.14-5.42 (m, 2H), 5.83-6.05 (m, 1H). ^{13}C NMR (150 MHz, $CDCl_3$) δ 29.5, 40.1, 46.2, 66.2, 67.9, 68.5, 117.5, 132.8, 155.7. IR ($CHCl_3$) 3427, 3017, 1686, 1471, 1133 cm^{-1}. MS (FAB) m/z 200 $(M-H)^-$. HRMS (FAB) m/z calcd for $C_9H_{14}N_1O_4$ $(M-H)^-$ 200.0923, found 200.0922.

Dial **1b**

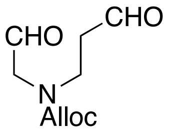

Pale yellow oil. ^{1}H NMR (400 MHz, $CDCl_3$, rotamers) δ 2.75-2.95 (m, 2H), 3.61 (t, J = 6.0 Hz, 2H), 4.15-4.25 (m, 2H), 4.50-4.68 (m, 2H), 5.14-5.40 (m, 2H), 5.75-6.10 (m, 1H), {9.55 (s), 9.57 (s), 1H}, 9.79 (s, 1H). ^{13}C NMR (150 MHz, $CDCl_3$, rotamers) δ 42.1, 43.1, 43.4, 43.8, 58.9, 59.0, 66.5, 66.7, 117.9, 118.1, 132.29, 132.35, 155.5, 155.9, 197.5, 197.6, 200.4, 201.0. IR ($CHCl_3$) 2982, 2960, 1701, 1630, 1308, 1276, 1232, 1176, 1039 cm^{-1}. MS (EI) m/z 199 $(M)^+$. HRMS (EI) m/z calcd for $C_9H_{13}N_1O_4$ $(M)^+$ 199.0845, found 199.0849.

Dial **1c**

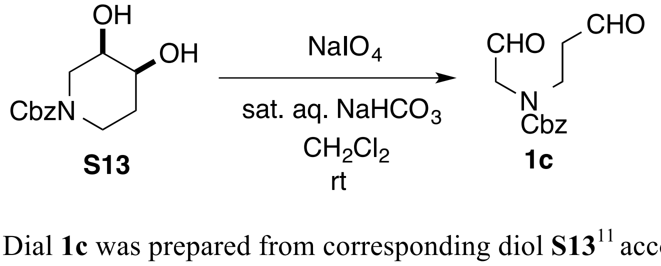

Dial **1c** was prepared from corresponding diol **S13**[11] according to the procedure for dial **1a**.

Pale yellow oil. ^{1}H NMR (600MHz, $CDCl_3$, rotamers) δ {2.77 (t, J = 6.2 Hz), 2.86 (t, J = 6.2 Hz), 2H},

3.54-3.64 (m, 2H), {4.16 (s), 4.19 (s), 2H}, {5.07 (s), 5.15 (s), 2H}, 7.20-7.40 (m, 5H), {9.50 (s), 9.55 (s), 1H}, {9.72 (s), 9.77 (s), 1H}. ^{13}C NMR (150 MHz, $CDCl_3$, rotamers) δ 42.1, 43.1, 43.4, 43.8, 58.9, 59.0, 67.6, 67.8, 127.9, 128.0, 128.2, 128.3, 128.5, 128.6, 136.95, 136.04, 155.7, 156.1, 197.49, 197.54, 200.4, 200.9. IR ($CHCl_3$) 3029, 2926, 1700, 1468, 1196 cm^{-1}. MS (EI) m/z 249 $(M)^+$. HRMS (EI) m/z calcd for $C_{13}H_{15}N_1O_4$ $(M)^+$ 249.1001, found 249.1004.

Dial **1d**

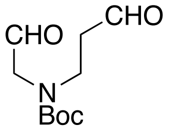

Dial **1d** was prepared from corresponding diol **S11**[10] according to the procedure for dial **1a**.
Pale yellow oil. ^{1}H NMR (600MHz, $CDCl_3$, rotamers) δ {1.43 (s), 1.48 (s), 9H}, {2.78 (t, *J* = 5.5 Hz), 2.83 (t, *J* = 5.5 Hz), 2H}, 3.50-3.60 (m, 2H), {4.04 (s), 4.11 (s), 2H}, {9.52 (s), 9.56 (s), 1H}, 9.80 (s, 1H). ^{13}C NMR (150 MHz, $CDCl_3$, rotamers) δ 28.1, 28.3, 42.5, 42.7, 43.6, 43.9, 58.5, 59.2, 81.0, 155.0, 155.3, 198.1, 198.2, 200.5, 201.1. IR ($CHCl_3$) 3027, 2979, 1732, 1693, 1605, 1197 cm^{-1}. MS (FAB) m/z 214 $(M–H)^-$. HRMS (FAB) m/z calcd for $C_{10}H_{16}N_1O_4$ $(M–H)^-$ 214.1079, found 214.1083.

Typical procedure of the intramolecular cross-aldol reaction of 23 (Table 4, Entry 3)

To a solution of dial **23** (23 mg, 0.10 mmol) in THF (500 μL) was added (*R*)-**12a** (2.1 mg, 5.0 μmol). After being stirred at 0 °C for 36 h, the mixture was diluted with THF (5 mL). The diluted mixture was added $NaBH_4$ (11.0 mg, 0.30 mmol), and stirred for 24 h at rt. The mixture was added H_2O and extracted with AcOEt. The extract was washed with brine, dried over anhydrous Na_2SO_4, filtered, and evaporated. The crude residues were purified by column chromatography on silica gel ($CHCl_3$/MeOH, 8:1) to afford the fraction including *anti*-**24** The yield of *anti*-**24** (96%) was determined by the integration of ^1H NMR signals in the presence of 1,3-dinitrobenzene as an internal standard.

Characterization and determination of the stereochemistry of *anti*-24 for Table 4

anti-(3*R*,4*R*)-**24**[12b]

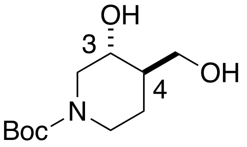

Spectroscopic data was reported in the literature.[12b]

The absolute configuration of *anti*-**24** was determined to be (3*R*,4*R*) by transformation to *N*-Bn diol **S14**[12] and comparison of its optical rotation to the literature data[12b,c] as shown below.

N-Bn diol **S14** prepared from *anti*-**25**, $[\alpha]_D^{20}$ = +4.9 (*c* 0.2, $CHCl_3$). Lit.[12c] $[\alpha]_D^{21}$ = +6.3 (*c* 1.0, $CHCl_3$) for (3*R*,4*R*)

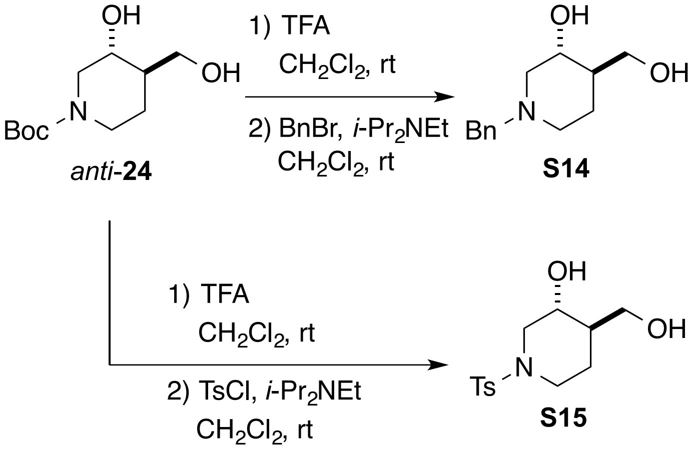

Experimental procedure to **S14** from *anti*-**24**: To a solution of *anti*-**24** (15 mg, 0.065 mmol) in CH_2Cl_2 (1.0 mL) was added TFA (0.20 mL) at rt. After being stirred for 4 h at rt, the mixture was evaporated *in vacuo* to give a residue. To a solution of the residue in CH_2Cl_2 (5.0 mL) were added DIPEA (0.23 mL, 1.30 mmol) and BnBr (12 mg, 0.072 mmol) at 0 ℃ under Ar atmosphere. After being stirred for 24 h at rt, the reaction was quenched with H_2O, and extracted with AcOEt. The extract was washed with brine, dried over anhydrous Na_2SO_4, filtered, and evaporated to give the residue. The residue was purified by prep. TLC (SiO_2, $CHCl_3$/MeOH, 8:1) to afford **S14**[12b,c] (7.6 mg, 53%).

Experimental procedure to **S15** from *anti*-**24**: To a solution of *anti*-**24** (13 mg, 0.054 mmol) in CH_2Cl_2 (1.0 mL) was added TFA (200 μL) at rt. After being stirred for 9 h at rt, the mixture was evaporated *in vacuo* to give a residue. To a solution of the residue in CH_2Cl_2 (2.0 mL) were added DIPEA (0.028 mL, 0.16 mmol)

and TsCl (12 mg, 0.07 mmol) at 0 ℃ under Ar atmosphere. After being stirred for 12 h at rt, the reaction was quenched with aq. 1% (v/v) HCl, and extracted with AcOEt. The extract was washed with aq. 1% (v/v) HCl and brine, dried over anhydrous Na_2SO_4, filtered, and evaporated to give the residue. The residue was purified by column chromatography on silica gel ($CHCl_3$/MeOH, 8:1) to afford **S15** (8.2 mg, 53%) as white solid.

N-Ts derivative **S15**

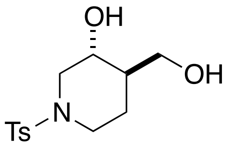

Colorless plates ($CHCl_3$). M.p. 103-105 °C. $[\alpha]_D^{22}$ = −34 (*c* 0.8, $CHCl_3$, 86% ee). ^{1}H NMR (600 MHz, $CDCl_3$) δ 1.31-1.41 (m, 1H), 1.43-1.52 (m, 1H), 1.60-1.67 (m, 1H), 2.05 (t, *J* = 9.6 Hz, 1H), 2.16-2.24 (m, 1H), 2.43 (s, 3H), 3.63-3.80 (m, 4H), 3.87-3.92 (m, 1H), 7.33 (d, *J* = 8.3 Hz, 2H), 7.64 (d, *J* = 8.3 Hz, 2H). ^{13}C NMR (150 MHz, $CDCl_3$) δ 21.5, 25.7, 43.8, 45.6, 51.6, 67.2, 71.7, 127.6, 129.7, 133.0, 143.8. IR ($CHCl_3$) 3617, 3504, 3029, 2945, 1599, 1417, 1201, 815 cm^{-1}. MS (FAB) m/z 286 $(M+H)^+$. HRMS (FAB) m/z calcd for $C_{13}H_{19}N_1O_4S_1$ $(M+H)^+$ 286.1113, found 286.1110.

The enantiomeric excess of *anti*-**24** was determined by HPLC analysis of *N*-Ts derivative **S15** with chiral stationary phase as shown below.

HPLC conditions: Chiralpak IA (0.46 x 25 cm), hexane–*i*-PrOH (80 : 20), 1.0 mL/min, 254 nm, t_R = 13.8 min (3*S*,4*S*), 21.5 min (3*R*,4*R*).

Racemic **S15**

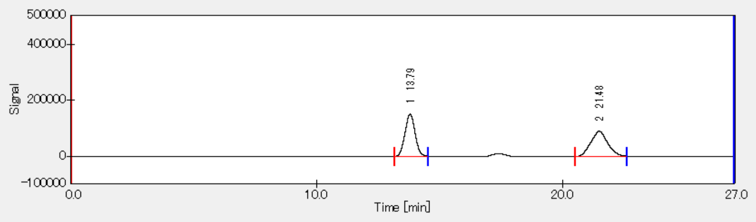

	Retention time (min)	Peak area (%)
1	13.79	50.2
2	21.48	49.8

Typical HPLC chromatogram of optically active **S15** (86% ee) derived from *anti*-**24** (Scheme 1A)

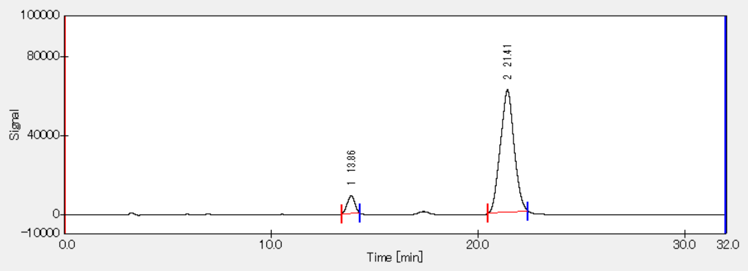

	Retention time (min)	Peak area (%)
1	13.86	7.2
2	21.41	92.8

Preparation of dial 23 for Table 4

Dial **23**

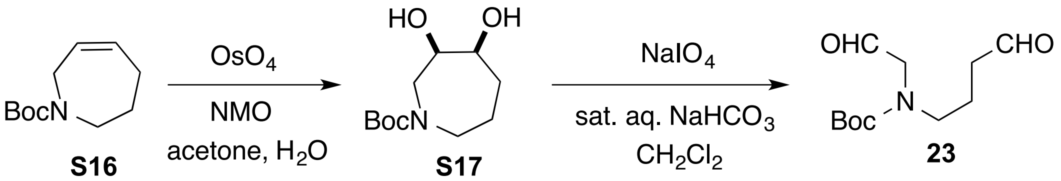

To a solution of olefin **S16**[13] (658 mg, 3.33 mmol) in acetone–H_2O (20 mL, 3/1) was added 0.1 M solution of OsO_4 in H_2O (230 μL) and NMO (702 mg, 5.99 mmol) at rt. After being stirred for 36 h at rt, the reaction was quenched with sat. aq. $Na_2S_2O_3$ and extracted with AcOEt. The extract was washed with brine, dried over anhydrous Na_2SO_4, filtered, and evaporated. The crude residue was purified by column chromatography on silica gel (*n*-hexane/AcOEt, 1:3) to afford diol **S17** (512 mg, 67%).

Dial **23** was prepared from diol **S17** according to the procedure for dial **1a**.

Diol **S17**

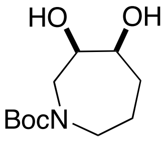

Relative stereochemistry of **S17** was deduced based on the mechanism of *syn*-addition of OsO_4.

Pale yellow oil. ^1H NMR (400 MHz, $CDCl_3$) δ 1.41 (s, 9H), 1.44-1.60 (m, 2H), 1.81-2.12 (m, 2H), 2.73 (br s, 1H), 2.96-3.73 (m, 4H), 3.81 (s, 2H), 4.18 (br s, 1H). ^{13}C NMR ($CDCl_3$, 150 MHz, rotamers) δ 20.9, 21.0, 28.2, 28.3, 28.4, 46.7, 47.2, 71.5, 71.9, 72.0, 79.7, 80.0, 155.5, 156.9. IR ($CHCl_3$) 3020, 1686, 1422, 1166 cm^{-1}. MS (FAB) m/z 232 $(M+H)^+$. HRMS (FAB) m/z calcd for $C_{11}H_{22}N_1O_4$ $(M+H)^+$ 232.1549, found 232.1550.

Dial **23**

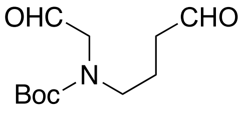

Pale yellow oil. ^1H NMR (600 MHz, $CDCl_3$) δ 1.34-1.53 (m, 9H), 1.75-1.89 (m, 2H), 2.40-2.62 (m, 2H), 3.21-3.43 (m, 2H), 3.80-4.05 (m, 2H), 9.56 (s, 1H), 9.77 (s, 1H). ^{13}C NMR (150 MHz, $CDCl_3$, rotamers) δ 20.7, 21.0, 28.1, 28.3, 40.6, 40.9, 47.7, 57.3, 57.6, 80.9, 155.1, 155.7, 198.4, 201.2, 201.5. IR ($CHCl_3$) 3020, 1686, 1422, 1166 cm^{-1}. MS (FAB) m/z 228 $(M–H)^-$. HRMS (FAB) m/z calcd for $C_{11}H_{18}N_1O_4$ $(M–H)^-$ 228.1236, found 228.1236.

Procedure of the intramolecular cross-aldol reaction of 25 and determination of the absolute configuration of aldol product 26 (Scheme 1)

The reaction was monitored by ^{1}H NMR. To a solution of dial **25** (30 mg, 0.12 mmol) in DMSO-d_6 (500 μL) was added cat. (*R*)-**13** (2.8 mg, 6.0 μmol). The reaction mixture was stored at 20 °C with monitoring ^{1}H NMR from time to time. After disappearing the signal of starting material **25**, the mixture was diluted with THF (5 mL). The diluted mixture was added $NaBH_4$ (14 mg, 0.36 mmol), and stirred for 24 h at rt. The mixture was added H_2O and extracted with AcOEt. The extract was washed with brine, dried over anhydrous Na_2SO_4, filtered, and evaporated. The crude residues were purified by column chromatography on silica gel ($CHCl_3$/MeOH, 8:1) to afford the fraction including *regio-,* and *diastereo-isomer*. The yields of these compounds, *anti*-**26** (74%) were determined by the integration of ^{1}H NMR signals in the presence of 1,3-dinitrobenzene as an internal standard.

anti-**26**

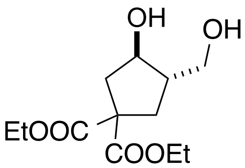

Yellow oil. $[\alpha]_D^{22}$ = +7.2 (*c* 0.8, $CHCl_3$). ^{1}H NMR (400 MHz, $CDCl_3$) δ 1.21-1.29 (m, 6H), 1.88 (dd, *J* = 9.6, 13.8 Hz, 1H), 2.11-2.63 (m, 4H), 3.63 (dd, *J* = 8.1, 11.0 Hz, 1H), 3.69-3.82 (m, 1H), 4.09-4.27 (m, 5H). ^{13}C NMR ($CDCl_3$, 100 MHz) δ 14.0, 34.1, 41.9, 49.0, 57.5, 61.7, 61.8, 65.0, 76.0, 172.0, 172.6. IR ($CHCl_3$) 3612, 2461, 2369, 821, 707, 432 cm^{-1}. MS (FAB) m/z 261 $(M+H)^+$. HRMS (FAB) m/z calcd for $C_{12}H_{21}O_6$ $(M+H)^+$ 261.1338, found 261.1338.

The absolute configuration of *anti*-**26** was determined to be (3*R*,4*S*) by transformation to *anti*-**S18** and comparison of its optical rotation to the literature data[14] as shown below.

anti-**S18**, $[\alpha]_D^{20}$ = +8.5 (*c* 2.0, $CHCl_3$). Lit.[14] $[\alpha]_D^{21}$ = −12.5 (*c* 2.23, $CHCl_3$) for (3*S*,4*R*)

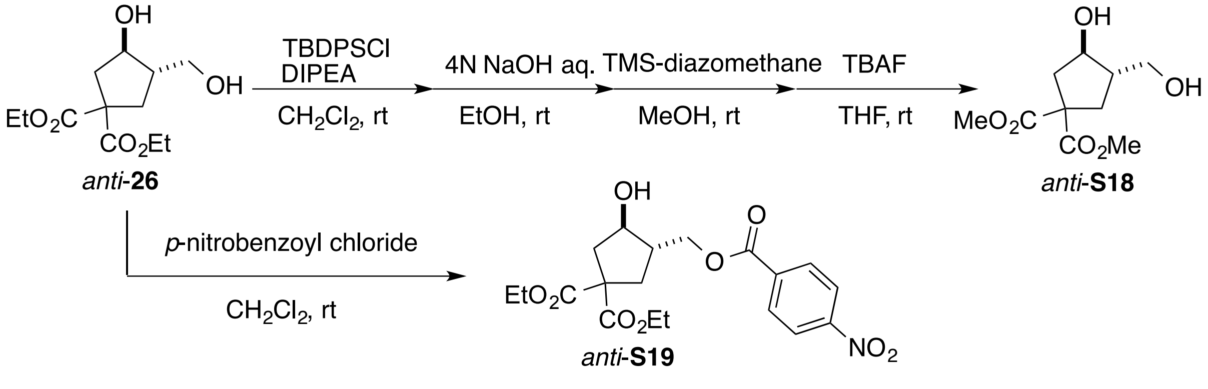

Experimental procedure for *anti*-**S18**: To a solution of *anti*-**26** (82% ee) (58 mg, 0.22 mmol), *N*-methylimidazole (18 mg, 0.22 mmol), and *i*-Pr_2EtN (52 mg, 0.40 mmol) in CH_2Cl_2 (5.0 mL) was added TBDPSCl (86 mg, 0.32 mmol) at 0 °C. After being stirred for 10 h at rt, the reaction was quenched with H_2O and extracted with AcOEt. The extract was washed with brine, dried over anhydrous Na_2SO_4, filtered, and evaporated.

To a solution of the residue in THF (5.0 mL) was added 4 N aq. NaOH (2.0 mL) at rt. After being stirred for 8 h at rt, the H_2O layer was acidified by 5 N aq. HCl to pH 2.0, and extracted with AcOEt. The extract was washed with brine, dried over anhydrous Na_2SO_4, filtered, and evaporated to afford the residue.

To a solution of the residue in MeOH–THF (5.0 mL, 3/5) was added trimethylsilyldiazomethane (48 mg, 0.42 mmol) at 0 °C. After being stirred for 12 h at rt, AcOH was added until yellow color of the mixture disappeared. The mixture was concentrated in vacuo, and then diluted with AcOEt. The organic layer was washed with sat. aq. $NaHCO_3$, brine, dried over anhydrous Na_2SO_4, filtered, and evaporated to give the residue.

To a solution of the residue in THF (5.0 mL) was added TBAF (76 mg, 0.29 mmol) at rt. the reaction was quenched with H_2O and extracted with AcOEt. The extract was washed with brine, dried over anhydrous Na_2SO_4, filtered, and evaporated to give the residue. The residue was purified by column chromatography on silica gel ($CHCl_3$/MeOH, 8:1) to afford *anti*-**S18** (20 mg, 86 μmol, 39% for 4 steps).

Experimental procedure for *anti*-**S19**: To a solution of *anti*-**26** (8.0 mg, 0.03 mmol) in CH_2Cl_2 (1.0 mL) was added *p*-nitrobenzoyl chloride (8.7 mg, 0.047 mmol) at 0 ℃ under Ar atmosphere. After being stirred for 12 h at rt, the reaction was quenched with H_2O, and extracted with AcOEt. The extract was washed with brine, dried over anhydrous Na_2SO_4, filtered, and evaporated to give the residue. The residue was purified by prep. TLC (SiO_2, hexane/AcOEt, 2:1) to afford *anti*-**S19** (5.7 mg, 44%).

anti-**S19**

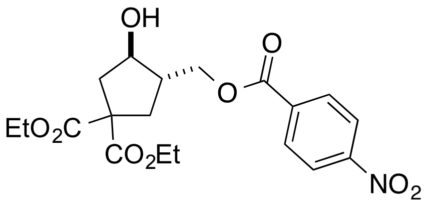

Yellow oil. $[\alpha]_D^{22} = -9.39$ (*c* 0.5, $CHCl_3$). ^{1}H NMR (600 MHz, $CDCl_3$) δ 1.20-1.32 (m, 6H), 2.08 (dd, *J* = 9.2, 13.7 Hz, 1H), 2.27-2.50 (m, 3H), 2.52-2.72 (m, 2H), 4.12-4.29 (m, 5H), 4.32-4.49 (m, 2H), 8.21 (d, *J* = 8.7 Hz, 2H), 8.30 (d, *J* = 8.7 Hz, 2H). ^{13}C NMR ($CDCl_3$, 100 MHz) δ 14.0, 34.7, 42.0, 46.8, 57.6, 61.8, 62.0, 66.2, 74.5, 123.6, 130.8, 135.3, 150.6, 164.7, 171.6, 172.6, 187.4. IR ($CHCl_3$) 3614, 3029, 2984, 2963, 2905, 1725, 1530, 1349, 1273, 1216, 810 cm^{-1}. MS (FAB) m/z 410 $(M+H)^+$. HRMS (FAB) m/z calcd for $C_{19}H_{24}N_1O_9S_1Na_1$ $(M+Na)^+$ 432.1271, found 432.1270.

The enantiomeric excess of *anti*-**26** was determined by HPLC analysis of *anti*-**S19** with chiral stationary phase as shown below.

HPLC conditions: Chiralpak IA (0.46 x 25 cm), hexane–*i*-PrOH (80 : 20), 1.0 mL/min, 254 nm, t_R = 11.4 min (3*S*,4*R*), 17.3 min (3*R*,4*S*).

Racemic **S19**

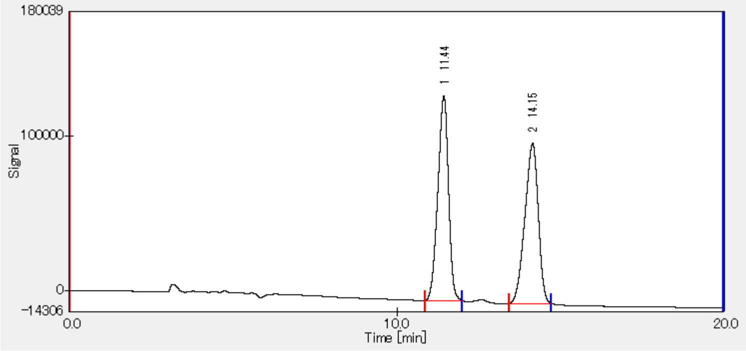

	Retention time (min)	Peak area (%)
1	11.84	8.9
2	13.32	91.1

HPLC chromatogram of optically active **S19** (82% ee) derived from *anti*-**26** in Scheme 1B.

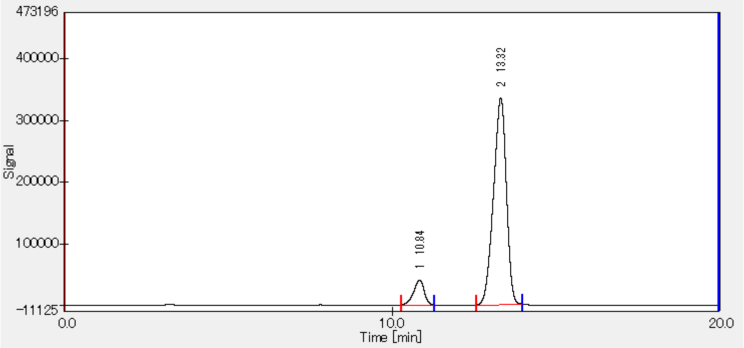

	Retention time (min)	Peak area (%)
1	11.44	50.4
2	17.30	49.6

Preparation of dial 25 for Scheme 1

Dial **25** was prepared from diol **S20**[9] according to the procedure for dial **1a**.

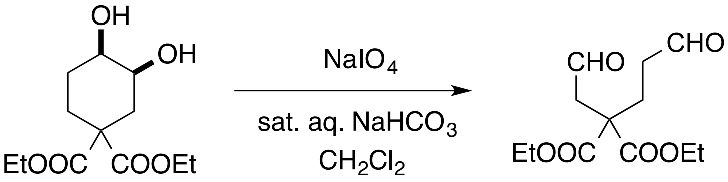

S20 **28**

Dial **25**

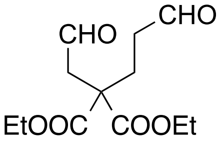

Pale yellow oil. ^{1}H NMR (400 MHz, $CDCl_3$) δ 1.27 (t, *J* = 7.4 Hz, 6H), 2.31 (t, *J* = 7.4 Hz, 2H), 2.57 (t, *J* = 7.4 Hz, 2H), 2.96 (s, 2H), 4.22 (q, *J* = 7.4 Hz, 4H), 9.72 (s, 1H), 9.75 (s, 1H). ^{13}C NMR (100 MHz, $CDCl_3$) δ 13.9, 26.1, 39.4, 46.9, 53.9, 62.1, 169.9, 198.3, 200.3. IR ($CHCl_3$) 3026, 1724, 1225, 1098, 1020 cm^{-1}. MS (FAB) m/z 259 $(M+H)^+$, 281 $(M+Na)^+$. HRMS (FAB) m/z calcd for $C_{12}H_{19}O_6$ $(M+H)^+$ 259.1182, found 259.1180.

Treatment of the mixture of aldol-adducts *anti*-7' and *syn*-8' with cat. (*R*)-13

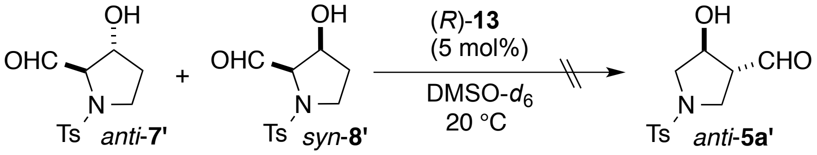

The mixture of aldol-adducts, *anti*-**7'** and *syn*-**8'**, was obtained from the background reaction. The DMSO-d_6 solution (500 μL) of dial **1a** (55 mg, 0.2 mmol) was kept for 72 h at 20 °C. Then, the reaction mixture was added H_2O, and extracted with AcOEt. The extract was washed with brine, dried over anhydrous Na_2SO_4, filtered, and evaporated to give the residue. The residue was purified by prep. TLC (SiO_2, hexane/AcOEt, 3:1) to afford the mixture of *anti*-**7'** and *syn*-**8'** (13 mg, 24% as the mixture).

To a solution of the mixture of *anti*-**7'** and *syn*-**8'** (13 mg, 0.10 mmol) in DMSO-d_6 (500 μL) was added cat. (*R*)-**13** (1.1 mg, 2.4 μmol) at rt. After being kept at 20 °C for 72 h, the aldehyde protons of *anti*-**7'** and *syn*-**8'** were monitored at 20 °C by ^{1}H NMR. There was no shift and consumption for the signals for *anti*-**7'** and *syn*-**8'** as shown in Figure S1.

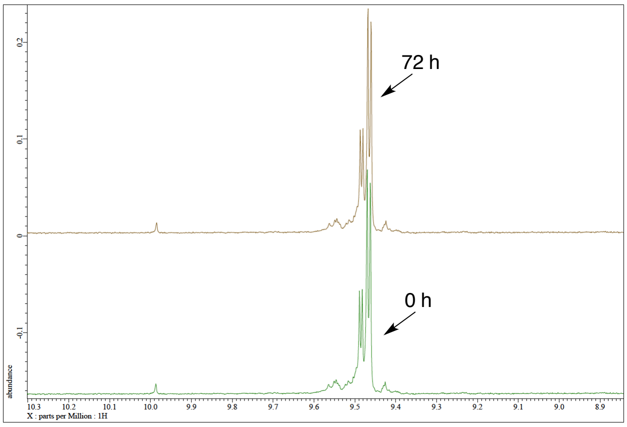

Figure S1. The aldehyde signals of the mixture of *anti*-**7'** and *syn*-**8'** in ^{1}H NMR spectra (DMSO-d_6).

To confirm the products after 72 h, the reaction mixture was diluted with THF (5.0 mL), and added $NaBH_4$ (5.3 mg, 0.14 mmol), and stirred 24 h at rt. The mixture was quenched with 1 N HCl, and extracted with AcOEt. The extract was washed with brine, dried over anhydrous Na_2SO_4, filtered, and evaporated. The crude residues were purified by column chromatography on silica gel ($CHCl_3$/MeOH, 8:1) to afford *anti*-**7** (5.0 mg, 38%) and *syn*-**8** (7.0 mg, 54%). No regioisomers, *anti*-**5a** and *syn*-**6**, were detected.

Preparation of 1a-D for Figure 4B

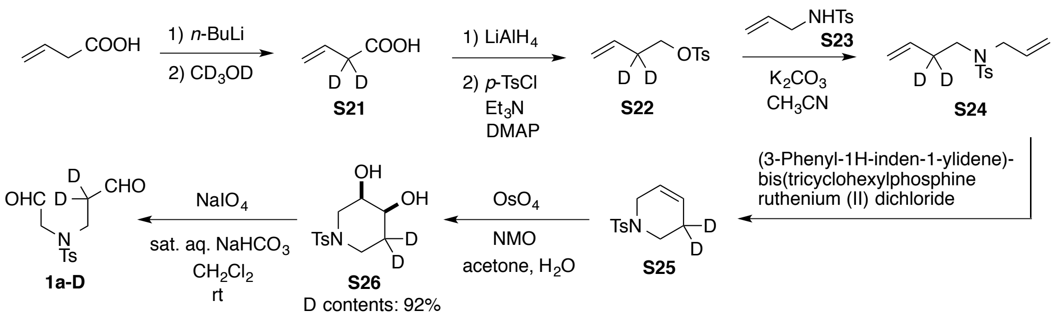

To a solution of 3-butenoic acid (2.0 g, 0.023 mol) in THF (60 mL) was added *n*-BuLi in hexane (0.051 mol) at –78 ℃ under Ar atmosphere. After being stirred for 1.5 h at –78 ℃, the reaction was quenched with CD_3OD (2.25 mL) and stirred for 30 min at rt. The reaction mixture was acidified by 2 N HCl (50 mL), and extracted with AcOEt. The extract was washed with brine, dried over anhydrous Na_2SO_4, filtered, and evaporated to give the residue (2.11 g) including mono-deuterated 3-butenoic acid.

This deprotonation–deuteration procedure was repeated twice towards the residue including mono-deuterated 3-butenoic acid to give the mixture containing di-deuterated 3-butenoic acid **S21**.

To a suspension of $LiAlH_4$ (5.90 g, 0.156 mol) in Et_2O (75 mL) was added the mixture containing **S21** (4.54 g) in Et_2O (15 mL) at 0 ℃ under Ar atmosphere. After being stirred for 4.5 h at rt, the reaction was quenched with H_2O (4.54 mL), 15% aq. NaOH (4.54 mL), and H_2O (13.5 mL) at 0 ℃, and the solid was filtered under the reduced pressure to give the mixture of extracted with AcOEt. The extract was washed with brine, dried over anhydrous Na_2SO_4, filtered, and evaporated to give the residue (4.15 g).

To a solution of the residue (4.15 g) in CH_2Cl_2 (75 mL) was added TsCl (10.7 g, 0.056 mol), DMAP (684 mg, 5.6 mmol), and Et_3N (14.3 mL, 0.112 mol) at 0 °C. After being stirred for 24 h at rt, the reaction was quenched with 2 N HCl (30 mL), and extracted with AcOEt. The extract was washed with brine, dried over anhydrous Na_2SO_4, filtered, and evaporated to give the residue (5.07 g). The crude residue was purified by column chromatography on silica gel (hexane/AcOEt, 50:1 to 20:1) to afford **S22**[15] (458 mg).

To a solution of **S22**[15] (380 mg, 1.67 mmol), **S23** (353 mg, 1.67 mmol) in CH_3CN (10 mL) was added K_3CO_3 (692 mg, 5.0 mmol). After being refluxed for 24 h, the mixture was quenched with sat. aq. $NaHCO_3$, and extracted with AcOEt. The extract was washed with brine, dried over anhydrous Na_2SO_4, filtered, and evaporated to give the residue (575 mg). The residue was purified by column chromatography on silica gel (hexane/AcOEt, 50:1 to 20:1) to afford **S24**[16] (357 mg, 80%).

To a solution of **S24**[16] (342 mg, 1.28 mmol) in CH_2Cl_2 (10 mL) was added (3-phenyl-1H-inden-1-ylidene)-bis(tricyclohexylphosphine ruthenium (II) dichloride THF adduct (2.3 mg, 0.0025 mmol). After being stirred for 24 h at rt, the catalyst (5.0 mg, 0.0054 mmol) was further added to the mixture and stirred for 3 h at rt. The mixture was evaporated to give the residue. The residue was purified by column chromatography on silica gel (hexane/AcOEt, 5:1) to afford **S25**[16] (215 mg, 70%).

To a solution of **S25**[16] (204 mg, 0.85 mmol) in acetone (3.5 mL)–H_2O (2.6 mL) was added 0.1 M solution of OsO_4 in H_2O (84 μL, 8.5 μmol) and NMO (199 mg, 1.7 mmol) at rt. After being stirred for 24 h at rt, the reaction was quenched with sat. aq. $Na_2S_2O_3$ and extracted with AcOEt. The extract was washed with brine, dried over anhydrous Na_2SO_4, filtered, and evaporated. The crude residue was purified by column chromatography on silica gel (*n*-hexane/AcOEt, 1:3 to $CHCl_3$/MeOH, 10:1) to afford **S10-D**[17] {198 mg, 85%, D contents: 92%, MS (FAB) m/z 296 $(M+Na)^+$. HRMS (FAB) m/z calcd for $C_{12}H_{15}D_2NO_4SNa$ $(M+Na)^+$ 296.0866, found 296.0896}.

Dial **1a-D** was prepared from diol **S10-D**[17] according to the procedure for dial **1a**.

KIE experiment for Figure 4B

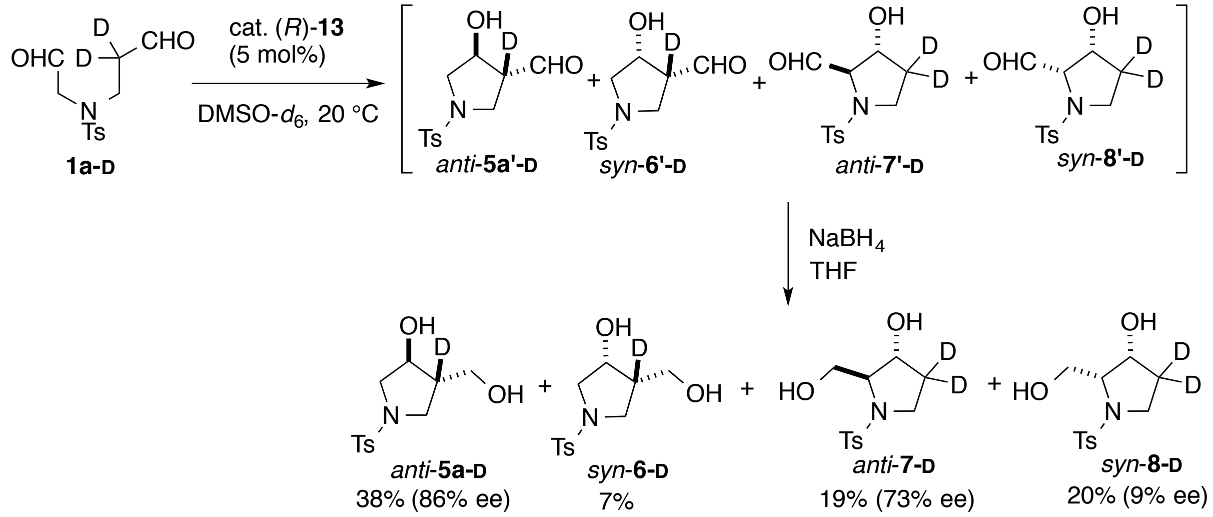

regioselectivity (**5a-D** + **6-D**) : (**7-D** + **8-D**) = 1.2 : 1

To a solution of dial **1a-D** (28 mg, 0.10 mmol) in DMSO-d_6 (343 μL) was added dibenzyl ether (4.0 mg, 20 μmol) in DMSO-d_6 (80 μL) as an internal standard, and cat. (*R*)-**13** (2.3 mg, 5.0 μmol) in DMSO-d_6 (77 μL) at rt. The reaction was monitored by the aldehyde protons of starting dial **1a-D** and the aldol adducts, *anti*-**5a'-D**, *syn*-**6'-D**, *anti*-**7'-D**, and *syn*-**8'-D**, by ^{1}H NMR at 20 °C (Figure S2).

The kinetic constant of the reaction ($k_{\text{D-6}}$) was calculated according to the kinetics of competitive reaction toward *anti*-**5a'-D**+*syn*-**6'-D** through the enamine formation at C(6)-formyl group and *anti*-**7'-D**+*syn*-**8'-D** through the enamine formation at C(1)-formyl group. Firstly, the kinetic constant of the total conversion ($k_{\text{total-D}}$ = 3.89 x 10^{-4} min^{-1} = $k_{\text{6-D}}$ + $k_{\text{1-D}}$) was determined by t he consumption of starting dial **1a-D** by monitoring the integration of the aldehyde proton as shown in Figures S2 and S3. The kinetic constant ($k_{\text{6-D}}$ = 1.34 x 10^{-4} min^{-1}) was calculated by the following equation with the concentrations of each isomer, which was determined by the ^{1}H NMR integration (I) of the aldehyde protons of aldol adducts, *anti*-**5a'-D**, *syn*-**6'-D**, *anti*-**7'-D**, and *syn*-**8'-D** (Table S4).

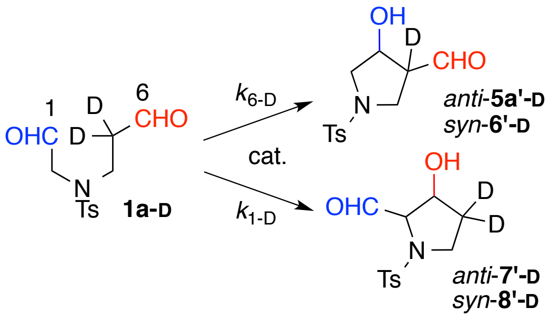

$k_{\text{6-D}}$ = $k_{\text{total-D}}$ • ([*anti*-**5a'-D**] + [*syn*-**6'-D**])/{ ([*anti*-**5a'-D**] + [*syn*-**6'-D**]) + ([*anti*-**7'-D**]+[*syn*-**8'-D**])}

The kinetic constant ($k_{\text{6-H}}$ = 4.49 x 10^{-4} min^{-1}) was also determined with dial **1a** in the same manner by the use of the same stock solutions of cat. (*R*)-**13** and dibenzyl ether used for the experiment with dial **1a-D** (Table S5, Figure S3). Thus, the KIE value ($k_{\text{6-H}}$ / $k_{\text{6-D}}$ = 3.4) was determined.

In the case of the reaction with dial **1a-D**, the yields of the aldol products were determined as depicted in Figure 4(B). After the reaction mixture was stored for 13 days at 20 °C in DMSO-d_6, the mixture was treated with $NaBH_4$, and determined the yields of each isomer, *anti*-**5a-D** (38%), *syn*-**6-D** (7%), *anti*-**7-D** (19%), and *syn*-**8-D** (20%), according to the procedure for Table 2, Entry 6.

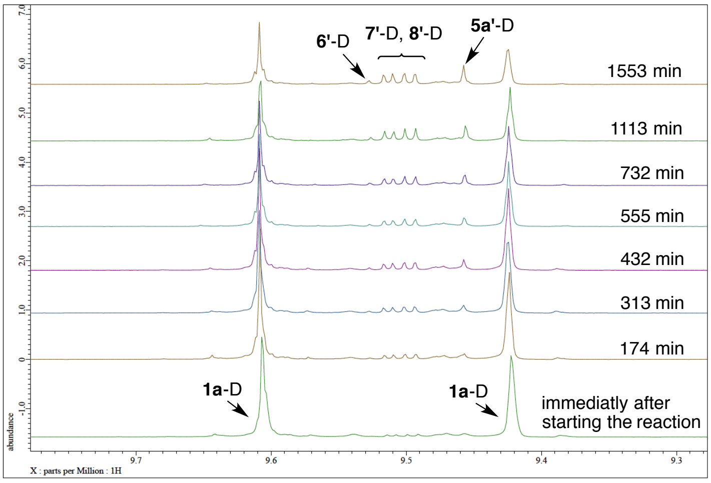

Figure S2. The signals of the aldehyde protons observed in the reaction mixture with **1a-D** (^{1}H NMR, DMSO-d_6).

Table S4. Kinetic data for **1a-D**.

Dial **1a-D**

Time (min)	174	313	432	555	732	1113	1553
Total conversion	0.060	0.116	0.178	0.235	0.283	0.376	0.441
−ln(1-conv.)	0.0619	0.123	0.196	0.268	0.333	0.472	0.582
^{1}H NMR integration of the isomers (I)							*anti*-**5a'-D** + *syn*-**6'-D** (0.79) *anti*-**7'-D** + *syn*-**8'-D** (1.51)
$k_{total\text{-}D}$ (min^{-1})	3.89 x 10^{-4}						
$k_{6\text{-}D}$ (min^{-1})	1.34 x 10^{-4}						

Table S5. Kinetic data for **1a**.

Dial **1a**

Time (min)	130	256	369	491	621	800	1170	1619
Total conversion	0.145	0.156	0.260	0.302	0.328	0.386	0.533	0.628
−ln(1-conv.)	0.157	0.170	0.301	0.360	0.397	0.488	0.761	0.989
^{1}H NMR integration of the isomers (I)								*anti*-**5a'** + *syn*-**6'** (2.88) *anti*-**7'** + *syn*-**8'** (0.92)
$k_{total\text{-}H}$ (min^{-1})	5.92 x 10^{-4}							
$k_{6\text{-}H}$ (min^{-1})	4.49 x 10^{-4}							

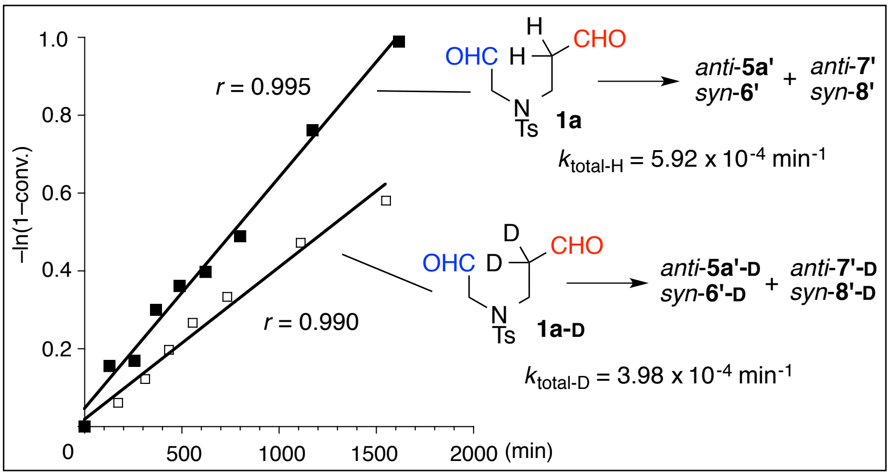

Figure S3. The kinetics of the total conversion with **1a** and **1a-D**.

Reductive amination for Figure 4C

The solution of (*dl*)-**11** (10 mg, 0.030 mmol) and **1a** (8 mg, 0.030 mmol) in DMSO (2.0 mL) was added $NaCNBH_3$ (5.5 mg, 0.089 mmol) at 20 °C and the reaction mixture was stirred at rt for 12 h under Ar atmosphere. The reaction was quenched with 1 N HCl aq, and extracted with AcOEt. The extract was washed with brine, dried over anhydrous Na_2SO_4, filtered, and evaporated. The crude residue was purified by prep. TLC (SiO_2, $CHCl_3$/MeOH, 12:1) to afford (*dl*)-**27** (2.8 mg, 15%) as yellow amorphous.

(*dl*)-**27**

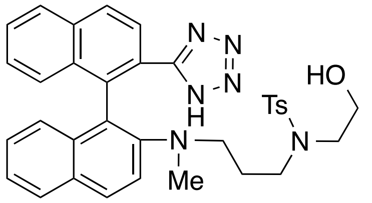

Yellow amorphous. ^{1}H NMR (600MHz, $CDCl_3$) δ 1.08-1.32 (m, 2H), 2.18-2.29 (m, 1H), 2.32 (s, 3H), 2.34-2.43 (m, 1H), 2.45 (s, 3H), 2.57 (t, 2H), 2.90-3.05 (m, 2H), 3.53 (t, 2H), 6.92 (d, J = 7.8 Hz, 1H), 7.21-7.33 (m, 4H), 7.31-7.44 (m, 2H), 7.48 (d, J = 7.8 Hz, 1H), 7.53-7.62 (m, 3H), 7.93 (d, J = 8.3 Hz, 1H), 7.98 (d, J = 8.2 Hz, 1H), 8.02-8.11 (m, 2H), 8.33 (d, J = 8.9 Hz, 1H); ^{13}C NMR (100 MHz, $CDCl_3$) δ 21.5, 26.8, 40.3, 46.8, 50.9, 53.2, 61.0, 120.8, 124.8, 125.1, 125.4, 126.5, 127.2, 127.5, 127.7, 127.9, 128.5, 129.3, 129.7, 130.2, 131.3, 133.1, 133.2, 133.4, 134.7, 135.6, 135.8, 140.9, 143.4, 149.6, 154.7; IR ($CHCl_3$) 3666, 3385, 3059, 2857, 1619 cm^{-1}. MS (FAB) m/z 607 (M+H)$^+$. HRMS (FAB) m/z calcd for $C_{34}H_{35}N_6O_3S_1Na_1$ (M+Na)$^+$ 629.2311, found 629.2310.

Computational details

DFT calculation of the model system consisting of 1,6-dial (**15**) and a simplified catalyst model (**20**) was conducted. Local minima and transition state (TS) structures along the reaction pathway were explored at B3LYP/6-31G* with the polarized continuum model (PCM) of DMSO using the "int=ultrafine" option. For TS structures of the enamine forming and the intramolecular C-C bond forming steps, in particular, conformational analysis focusing on the hydrogen bonding networks and hydrocarbon skeletons were conducted systematically. Based on the primary study, typical structures were re-optimized with PCM(DMSO)-B3LYP/6-31+G** and PCM(DMSO)-ωB97XD/6-31+G**. Frequency analyses were also carried out to identify the stationary points (local minima: no imaginary frequencies, TS: one imaginary frequency) and to estimate thermodynamic properties at 298.15 K and 1 atm. For all the calculations, Gaussian 09 Revision D.01. was used.[18] The molecular structures were depicted by using the CYLview v1.0.561 β.[19]

Mechanism analysis

According to Houk's computational study for the L-proline catalyzed aldol reaction[20], the nucleophilic catalysis of **20** was explored (Figure S4). The reaction mechanism includes five steps as follows; 1) nucleophilic addition of the aniline amino group to the formyl group (**TS1**), 2) iminium formation (**TS2**), 3) enamine formation (**TS3**), 4) C-C bond formation (**TS4**, intramolecular aldol reaction), 5) catalyst regeneration (**TS5**, **TS6**).

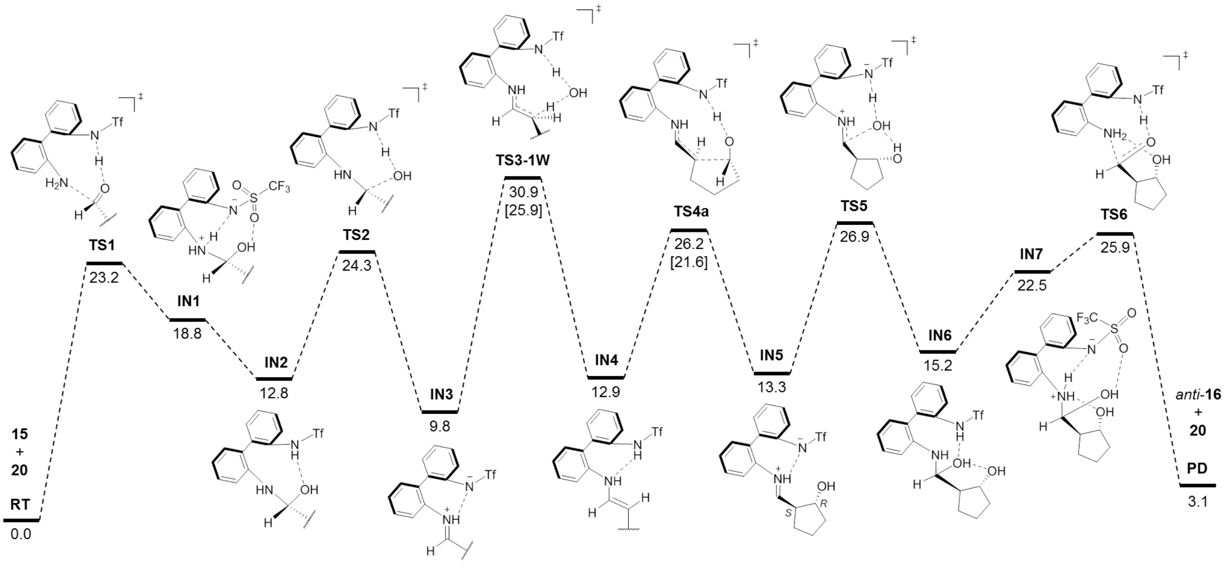

Figure S4. Gibbs free energy profile of the **20** catalyzed intramolecular cross-aldol reaction of **15** at PCM(DMSO)-B3LYP/6-31+G** (in kcal/mol). The relative Gibbs free energy calculated at PCM(DMSO)-ωB97XD/6-31+G** are also shown in brackets.

The free energy profile of the **20** catalyzed intramolecular cross-aldol reaction of **15** was shown in Figure S4. The computational result indicates that the enamine forming step (**TS3**) from the iminium intermediate (**IN3**) and the C-C bond forming step (**TS4**) from the enamine intermediate (**IN4**) is the rate-determining and the stereo-determining steps of this reaction, respectively. The energy profile would be qualitatively unchanged even at the PCM(DMSO)-ωB97XD/6-31+G** level including dispersion effects. The enamine forming step remains rate-determining regardless of computational method. The iminium formation (**TS1**,

TS2) through the nucleophilic addition of **20** and dehydration is reversible process. In the step of intramolecular proton transfer, TS was found to be located nearby zwitter ionic and less stable intermediates (e.g., **IN1**, **IN7**).

Both **TS3** and **TS4** are key steps for controlling catalytic activity and stereoselectivity. Therefore, TS structures in those steps were investigated in details. In contrast to the previous work on the L-proline catalyzed aldol reaction[20], in which only a direct isomerization through a proton transfer from CH_2 at the β-position to a carboxylate anion was reported, enamine formation is accelerated by proton-relay through a molecule of H_2O (Figure S5). The H_2O-mediated TS structures (**TS3-1W** = **TS3**, **TS3-2W**) are more stable than that without involving H_2O (**TS3-0W**).

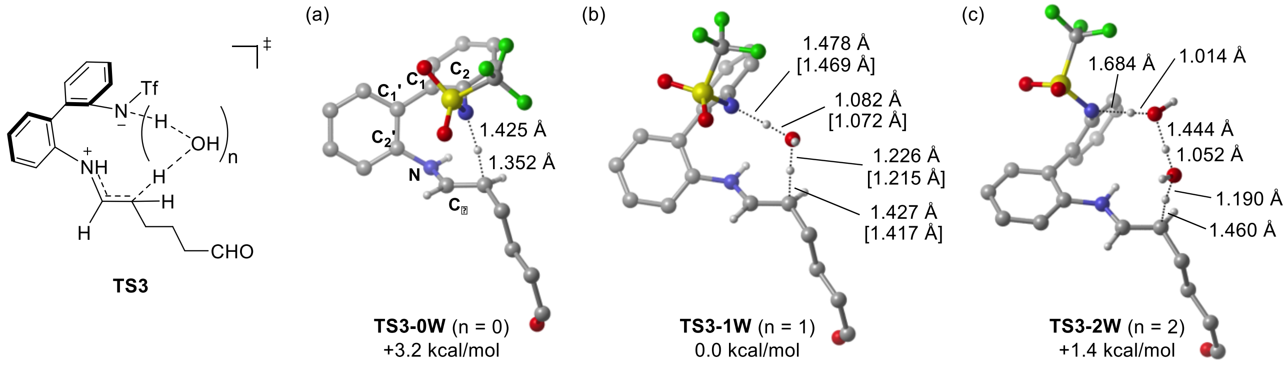

Figure S5. Schematic structures and relative Gibbs free energies for **TS3**. (a) **TS3-0W**: without H_2O. (b) **TS3-1W**: with a molecule of H_2O. (c) **TS3-2W**: with two molecules of H_2O. The values in brackets are calculated at PCM(DMSO)-ωB97XD/6-31+G**. Unimportant hydrogen atoms were omitted from the 3D structures.

The structural properties and thermodynamic parameters for **TS3** were shown in Table S6. Without involvement of a H_2O (**TS3-0W**), the dihedral angle between biaryl axis (C_2-C_1-C_1'-C_2') has to be narrowed to 62.7 ° from usual dihedral angle (~ 90 °) for binaphthyl compounds. Furthermore, the aryl and the enamine planes are also twisted (the dihedral angle of C_1'-C_2'-N-Cα: 105.3 °) in larger extent than that observed in **TS3-1W** (the dihedral angle of C_1'-C_2'-N-Cα: 141.8 °).

Table S6. Structural properties and thermodynamic parameters for TS3

TS3	dihedral angle C_2-C_1-C_1'-C_2' (°)	dihedral angle C_1'- C_2'-N-Cα (°)	$\Delta\Delta H^a$	$T\Delta\Delta S^a$
**TS3-0W**	62.7	105.3	12.8	9.6
**TS3-1W**	66.8	141.8	0.0	0.0
**TS3-2W**	77.9	178.8	– 7.9	– 9.2

[a] kcal/mol at 298.15 K under 1 atm.

These structural distortions would cause a large enthalpic disadvantage to destabilize **TS3-0W** rather than **TS3-1W**. In the case of **TS3-2W** involving two molecules of H_2O, large entropic cost ($T\Delta\Delta S$ = – 9.2 kcal/mol) has to be paid, even though the enthalpic factor is favorable, destabilizing in 1.3 kcal/mol from **TS3-1W**. These computational results suggested that a molecule of H_2O is involved in the enamine forming step.

The transition states for stereo-determining C-C bond forming step (**TS4**) were also investigated (Figure S6). Two proton sources, NHTf and iminium-NH, are available for interaction with carbonyl oxygen during the cause of the C-C bond formation. Based on the structural demand that NHTf or iminium-NH should be oriented to the carbonyl oxygen, TS structures on each diastereomer (1S2R, 1R2S, 1S2S, 1R2R) were explored systematically: 1) relative bond orientations of C1'-C2' and N-C1 (anti, syn), C2'-N and C1-C2 (anti, syn), and N-C1 and C2-C3 (anti, syn), 2) five-membered ring conformer (half chair 1-2, envelope 1-2), and 3) N-S bond rotation (out, in), respectively (Scheme S1).

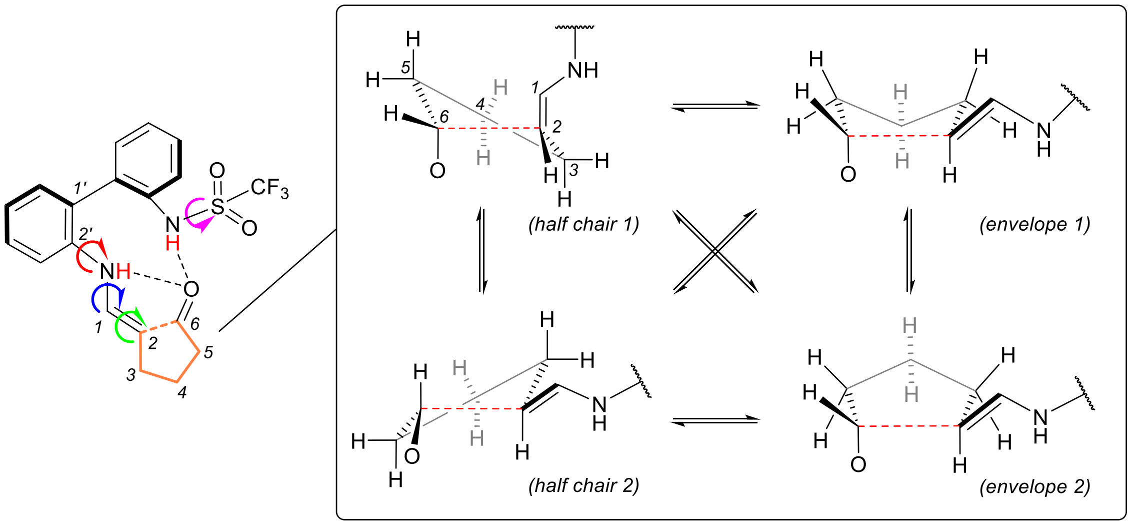

Scheme S1. Possible conformations in the intramolecular C-C bond forming step.

It was found that the order of the stabilities of TSs (**TS4a** > **TS4b** > **TS4c** > **TS4d**) explained well the experimental yields of the aldol isomers, *anti*-**16** (55%), *syn*-**17** (5%), *ent*-*syn*-**17** (4%), and *ent*-*anti*-**16** (3%) presented in Table 1, Entry 2 (Figure S6).

The structural and electronic factors in **TS4** significantly play crucial roles for controlling the stereoselectivity. In the most stable **TS4a** leading to the major *anti*-isomer, (1*R*,2*R*)-**16**, the diastereoface of the enamine is found to be controlled to *Si* face due to all *anti* geometries in C1'-C2'-N-C1, C2'-N-C1-C2 as well as N-C1-C2-C3.

Furthermore, **TS4a** has a stable gauche orientation around the C-C bond forming moiety. These conformational restrictions require no structural distortion and cause the most strong N-H•••O hydrogen bond between the sulfonylamide NH and the formyl carbonyl group, stabilizing **TS4a** (Figure S6a). In the next stable **TS4b** leading to the minor *syn*-isomer, (1*S*,2*R*)-**17**, the facial selectivity of the formyl group is reversed, resulting in the less stable eclipse orientation (Figure S6b). In contrast, the energetically disfavored **TS4c** and **TS4d** for *ent*-*syn*-**17** and *ent*-*anti*-**16** are caused by the less stable *syn* geometry in C2'-N-C1-C2 as well as the eclipse orientation around the C-C bond forming moiety (Figures S6c and S6d). The PCM(DMSO)-ωB97XD/6-31+G** calculations showed similar tendencies in the relative energies and

the gross structures of **TS4**. Whereas **TS4a** is the most stable TS, the energy difference between **TS4b** and **TS4c** is negligibly small. There exists a relatively large structural change for TSs affording *anti*-isomers (**TS4a** and **TS4d**), in which the C-C bond forming distances are shorten (i.e. late TS).

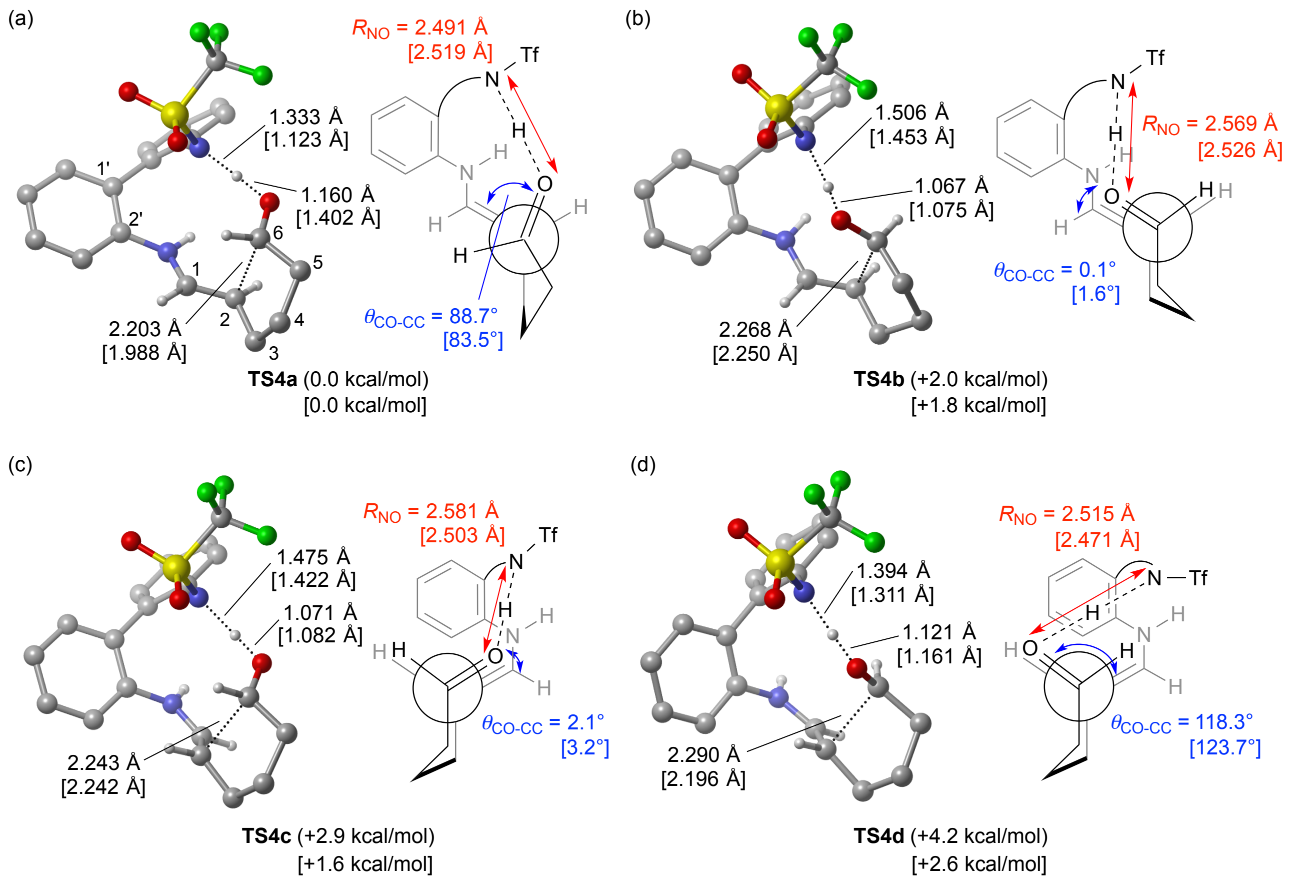

Figure S6. 3D structures and the relative Gibbs free energies of (a) **TS4a**, (b) **TS4b**, (c) **TS4c**, and (d) **TS4d** leading to (1*R*,2*R*), (1*S*,2*R*), (1*R*,2*S*), and (1*S*,2*S*) products at PCM(DMSO)-B3LYP/6-31+G**. The values in brackets are calculated at PCM(DMSO)-ωB97XD/6-31+G**. Unimportant hydrogen atoms were omitted from the 3D structures.

Cartesian coordinates of optimized structures

(PCM(solv=dimethylsulfoxyde)-B3LYP/6-31+G**)

1,6-dial (C_i) (= **15**)

E = -385.129719 au, *G* = -385.015292 au

C	-0.532083	-0.259506	1.872909
H	-1.316993	0.497846	1.965269
H	-1.015739	-1.238836	1.736275
C	0.384168	0.016293	0.663750
H	0.861791	0.996754	0.784716
H	1.190295	-0.728096	0.637551
C	-0.384168	-0.016293	-0.663750
H	-1.190295	0.728096	-0.637551
H	-0.861791	-0.996754	-0.784716
C	0.532083	0.259506	-1.872909
H	1.316993	-0.497846	-1.965269
H	1.015739	1.238836	-1.736275
C	0.238861	-0.333851	3.161144
O	-0.006371	0.323952	4.160040
H	1.086765	-1.049632	3.170353
C	-0.238861	0.333851	-3.161144
O	0.006371	-0.323952	-4.160040
H	-1.086765	1.049632	-3.170353

20

E = -1459.684582 au, *G* = -1459.503937 au

C	2.335967	-0.924302	1.000004
C	0.063334	1.280931	0.343649
C	2.260878	0.031108	-0.041423
C	3.105147	-2.085072	0.824884
C	1.448152	1.279182	0.060053
C	-0.665675	2.473346	0.376112
C	3.006943	-0.197998	-1.211651
C	3.815592	-2.300676	-0.354818
C	2.058559	2.522794	-0.187085
C	-0.039061	3.690075	0.107192
C	3.775504	-1.349642	-1.379264
H	4.405581	-3.205404	-0.467530
C	1.329856	3.712513	-0.172306
H	-0.613264	4.610802	0.129088
H	-1.723278	2.438399	0.612915
H	3.151897	-2.813968	1.629661
N	-0.614158	0.046912	0.649084
H	-0.108520	-0.554158	1.329144
S	-1.447527	-0.814526	-0.478675
O	-1.348448	-0.205426	-1.806061
O	-1.223589	-2.246218	-0.258780
N	1.583231	-0.744857	2.185766
H	1.664942	0.185748	2.587534
H	1.790021	-1.436263	2.898881
C	-3.242495	-0.553422	0.038964
F	-4.036366	-1.260524	-0.776891
F	-3.428637	-0.969451	1.297403
F	-3.576090	0.741361	-0.046915
H	4.327533	-1.506020	-2.300648
H	2.954657	0.533887	-2.012354
H	3.124370	2.548427	-0.391972
H	1.832904	4.654239	-0.369372

TS1

E = -1844.798021 au, G = -1844.482222 au

$\nu^{\ddagger}$ = 921.4749i cm^{-1}

C	2.214324	-2.384628	-0.425598
C	0.965238	0.361120	1.264653
C	2.677305	-1.268154	0.292996
C	2.975958	-2.951669	-1.451590
C	1.905227	-0.681408	1.436711
C	0.304482	0.888250	2.383999
C	3.936390	-0.747643	-0.042838
C	4.223470	-2.416334	-1.769076
C	2.149032	-1.163720	2.734128
C	0.574747	0.408659	3.665698
C	4.704896	-1.309327	-1.063881
H	4.814563	-2.863136	-2.562093
C	1.496840	-0.625178	3.844546
H	0.055571	0.835209	4.518585
H	-0.428293	1.674133	2.243771
H	2.592618	-3.809809	-1.996270
N	0.648642	0.797945	-0.059543
H	0.324596	-0.074408	-0.909876
S	1.009154	2.261463	-0.597826
O	1.926976	3.008983	0.279280
O	1.257522	2.210118	-2.048406
C	-1.594230	-2.017359	-0.283826
C	-0.418664	-2.071700	-1.242924
N	0.902078	-2.909946	-0.162475
H	-1.378449	-1.319383	0.533508
H	-0.477787	-2.884374	-1.976643
O	0.105953	-0.981582	-1.704148
H	0.845300	-3.909672	-0.354615
H	-1.751865	-3.010942	0.152500
H	5.675775	-0.887397	-1.303897
H	4.311600	0.107473	0.510980
H	2.872148	-1.963403	2.866654
H	1.707973	-1.010100	4.837307
H	0.627919	-2.762188	0.809508
C	-4.098236	-1.568843	-0.093114
H	-4.248830	-2.570517	0.327384
H	-3.913710	-0.900427	0.756625
C	-5.370216	-1.127632	-0.817410
H	-5.259075	-0.122823	-1.253932
H	-5.591906	-1.778382	-1.677767
C	-6.617225	-1.092172	0.021195
O	-6.679659	-1.384789	1.204691
H	-7.532920	-0.769587	-0.514821
C	-0.591890	3.266404	-0.526667
F	-1.003748	3.447820	0.741198
F	-0.395461	4.475476	-1.080623
F	-1.570853	2.641852	-1.202934
C	-2.874382	-1.575732	-1.018959
H	-2.721882	-0.574972	-1.440248
H	-3.059024	-2.250443	-1.865697

IN1

E = -1844.811805 au, G = -1844.489312 au

C	-0.242376	1.599007	0.585556
C	2.427564	0.617989	-1.071396

C	1.044411	2.170022	0.458612
C	-1.091407	1.929427	1.641992
C	2.018897	1.915139	-0.652712
C	3.433254	0.490674	-2.047859
C	1.421142	3.094358	1.453110
C	-0.682616	2.848809	2.608903
C	2.626902	3.032425	-1.257178
C	4.030728	1.617508	-2.608148
C	0.580255	3.432416	2.512075
H	-1.349890	3.098848	3.427020
C	3.620857	2.897741	-2.223992
H	4.805662	1.491912	-3.358804
H	3.736165	-0.499806	-2.363888
H	-2.075034	1.485395	1.723696
N	1.709100	-0.463682	-0.520463
S	2.144695	-1.967409	-0.371868
O	0.919694	-2.772035	-0.117301
O	3.131490	-2.512789	-1.325915
C	3.055634	-2.078791	1.283167
F	4.148974	-1.293585	1.260808
F	2.266226	-1.675195	2.292372
F	3.444289	-3.342506	1.528813
C	-1.775439	-0.400477	-0.122938
N	-0.658164	0.611763	-0.428524
H	0.239991	0.004965	-0.568519
H	0.916795	4.141870	3.261463
H	2.410014	3.536971	1.397784
H	2.303461	4.025469	-0.960668
H	4.065220	3.780043	-2.673700
C	-4.294834	-0.737146	-0.013857
C	-3.143546	0.154751	-0.499030
H	-4.163597	-1.748001	-0.416565
H	-4.250215	-0.823625	1.080333
H	-3.258305	1.163371	-0.088795
C	-5.666889	-0.189791	-0.428974
H	-5.797888	0.824104	-0.031392
H	-5.713089	-0.101869	-1.521268
C	-6.815411	-1.074910	0.054888
H	-6.812521	-1.178704	1.151252
H	-6.715608	-2.105909	-0.319006
H	-1.685914	-0.607571	0.948193
H	-3.175534	0.247890	-1.592479
C	-8.195201	-0.616494	-0.325802
O	-8.451148	0.385067	-0.975148
H	-9.019880	-1.266960	0.030159
O	-1.520977	-1.535527	-0.892020
H	-0.748405	-2.030141	-0.543653
H	-0.862506	1.071622	-1.321732

IN2

E = -1844.819293 au, *G* = -1844.498896 au

C	0.358868	-2.459082	-0.506932
C	1.555056	-0.013799	1.368862
C	1.608856	-2.234447	0.117651
C	0.313003	-3.221040	-1.688048
C	1.727495	-1.415961	1.363933
C	1.699235	0.723505	2.547967
C	2.761929	-2.816725	-0.432108
C	1.475502	-3.760299	-2.236713

C	2.052905	-2.037877	2.580717
C	2.033076	0.083714	3.742523
C	2.708857	-3.569975	-1.605531
H	1.412121	-4.345813	-3.149314
C	2.211504	-1.301357	3.756795
H	2.146527	0.663705	4.652844
H	1.549669	1.797210	2.520338
H	-0.642560	-3.414821	-2.164923
N	1.206308	0.685614	0.157349
S	2.321801	1.329988	-0.856100
O	3.680797	0.990763	-0.432645
O	1.869582	1.169114	-2.241166
C	2.173620	3.186532	-0.552791
F	0.921256	3.596466	-0.791065
F	2.496803	3.485652	0.712671
F	3.008791	3.832789	-1.378041
C	-1.799195	-1.243511	-0.710090
N	-0.832819	-1.976491	0.075829
H	3.615531	-3.997988	-2.021319
H	3.715144	-2.649634	0.061334
H	2.183769	-3.115644	2.596102
H	2.467670	-1.810511	4.680846
C	-4.154412	-0.238258	-0.649018
C	-3.017354	-0.895410	0.141305
H	-3.786673	0.681424	-1.119060
H	-4.462393	-0.907239	-1.464801
H	-3.372716	-1.821691	0.607182
C	-5.369509	0.083039	0.231248
H	-5.739547	-0.835968	0.702432
H	-5.066307	0.748454	1.049150
C	-6.503388	0.737900	-0.557659
H	-6.837704	0.098986	-1.390211
H	-6.171897	1.669210	-1.043244
H	-2.098834	-1.847850	-1.569942
H	-2.694250	-0.229624	0.953833
C	-7.734465	1.084844	0.231224
O	-7.887481	0.883925	1.425730
H	-8.547685	1.560018	-0.354565
O	-1.234856	0.001714	-1.222307
H	-0.939901	-0.128064	-2.134960
H	-0.688372	-1.562203	0.990604
H	0.319688	0.455325	-0.325992

TS2

E = -1844.798616 au, *G* = -1844.480537 au

$\nu^{\ddagger}$ = 286.2710*i* cm^{-1}

C	0.304925	1.710403	0.250973
C	-1.406589	-0.946882	1.218296
C	-0.831615	1.531047	1.063478
C	0.487965	2.895220	-0.472027
C	-1.060522	0.276579	1.844240
C	-1.623868	-2.086118	2.012781
C	-1.751862	2.587347	1.152786
C	-0.460602	3.915289	-0.397587
C	-0.969322	0.319169	3.245944
C	-1.535005	-2.019685	3.403227
C	-1.579124	3.767119	0.427100
H	-0.310190	4.830187	-0.962177
C	-1.207334	-0.812793	4.026875

H	-1.712253	-2.912808	3.995280
H	-1.870077	-3.022800	1.526301
H	1.384305	3.030778	-1.069054
N	-1.446052	-1.015639	-0.205033
S	-2.723113	-1.506573	-1.007810
O	-2.309820	-1.845766	-2.385569
O	-3.642039	-2.420634	-0.297642
C	3.114195	-0.615456	-0.791538
H	3.268296	-1.161738	-1.725814
C	1.847353	0.190750	-0.919882
H	1.789399	0.857989	-1.775031
N	1.308155	0.696583	0.210013
H	1.414896	0.145160	1.055210
H	-2.313022	4.563287	0.504642
H	-2.626350	2.463635	1.784252
H	-0.705005	1.258892	3.722566
H	-1.129496	-0.753487	5.108139
O	0.722002	-1.056196	-1.547341
H	0.518438	-0.919906	-2.487674
H	-0.211029	-1.015577	-1.003376
H	2.990839	-1.358954	0.004852
C	5.635764	-0.526487	-0.450506
H	5.556972	-1.303823	0.319103
H	5.785023	-1.047645	-1.403689
C	6.850897	0.353918	-0.158666
H	6.963957	1.149775	-0.911345
H	6.741277	0.891084	0.796295
C	8.170719	-0.362855	-0.095407
O	8.329349	-1.562151	-0.257786
H	9.045449	0.284520	0.117748
C	4.332730	0.282602	-0.502377
H	4.184538	0.807690	0.448850
H	4.411332	1.053751	-1.279951
C	-3.813412	0.015984	-1.274950
F	-4.901406	-0.304709	-2.002355
F	-3.143302	0.979905	-1.930928
F	-4.229727	0.516538	-0.096146

IN3

E = -1768.366625 au, *G* = -1768.073249 au

C	-0.599301	2.068741	-0.550788
C	-1.756072	-0.284157	1.407465
C	-1.895986	1.854469	-0.029691
C	-0.367932	2.905660	-1.648103
C	-2.215319	1.042715	1.185801
C	-2.168051	-0.970714	2.565745
C	-2.945281	2.539627	-0.672666
C	-1.430335	3.574972	-2.250324
C	-3.068007	1.622664	2.146810
C	-3.029664	-0.379350	3.486253
C	-2.724834	3.384737	-1.759753
H	-1.249500	4.219073	-3.104453
C	-3.478657	0.929305	3.282934
H	-3.333578	-0.933627	4.369553
H	-1.801604	-1.977021	2.729161
H	0.638094	3.008271	-2.041876
N	-0.807958	-0.825523	0.507334
S	-0.887511	-2.260682	-0.140720
O	0.420239	-2.598859	-0.738340

O	-1.588195	-3.307640	0.636361
C	2.785922	1.040762	0.824459
H	2.447397	0.024343	1.044871
C	1.668853	1.853947	0.282573
H	1.835911	2.910488	0.074104
N	0.502105	1.370218	0.035340
H	0.242561	0.337720	0.231459
H	-3.566375	3.882937	-2.230783
H	-3.958039	2.391702	-0.313061
H	-3.403096	2.644576	1.998572
H	-4.131428	1.409283	4.005421
H	3.095863	1.509990	1.768537
C	5.184156	0.260674	0.466620
H	5.481648	0.735180	1.409219
H	4.879805	-0.762905	0.714715
C	6.382502	0.217499	-0.481417
H	6.124401	-0.261320	-1.438610
H	6.716643	1.229305	-0.758687
C	7.593634	-0.502554	0.043554
O	7.676892	-1.032550	1.139704
H	8.458045	-0.531685	-0.650183
C	3.997227	1.020371	-0.139660
H	4.295574	2.049792	-0.372989
H	3.696517	0.551534	-1.084009
C	-2.003386	-2.086328	-1.661031
F	-2.124430	-3.262046	-2.308219
F	-1.503178	-1.184307	-2.525988
F	-3.236393	-1.678696	-1.298310

TS3-0W
E = -1768.325595 au, G = -1768.034217 au,
$\nu^{\ddagger}$ = 1323.1781i cm^{-1}

C	-0.818015	2.626657	-0.317094
C	-1.562986	-0.239839	1.156459
C	-2.048418	1.979403	-0.073570
C	-0.684027	3.600785	-1.305758
C	-2.248558	0.997513	1.040658
C	-1.806605	-1.062683	2.266696
C	-3.142360	2.363929	-0.866364
C	-1.783867	3.946333	-2.094284
C	-3.182125	1.334472	2.037472
C	-2.732753	-0.703894	3.246382
C	-3.015074	3.326815	-1.870385
H	-1.678343	4.701247	-2.866844
C	-3.431196	0.499082	3.127525
H	-2.904992	-1.360749	4.093648
H	-1.259000	-1.995581	2.348326
H	0.275555	4.088335	-1.446230
N	-0.563712	-0.636617	0.211317
S	-0.918769	-1.455189	-1.104448
O	-2.319984	-1.339567	-1.554368
O	0.170750	-1.286987	-2.085413
C	1.932164	0.447216	0.639153
H	1.863214	0.475079	1.730792
C	1.301582	1.493321	-0.047937
H	1.429387	1.575939	-1.125791
N	0.325650	2.242674	0.466522
H	0.165097	2.157676	1.467004
H	-3.877886	3.591063	-2.473903

H	-4.101179	1.882121	-0.701898
H	-3.709683	2.280248	1.956795
H	-4.155499	0.793922	3.880552
C	-0.772388	-3.294162	-0.677991
H	0.791488	-0.224020	0.361894
C	3.173862	-0.220877	0.076504
C	4.479152	0.482293	0.488769
H	3.197378	-1.261205	0.423228
H	4.468659	1.515739	0.122223
H	4.535814	0.542014	1.582343
C	5.717390	-0.236962	-0.045974
H	5.767439	-1.277021	0.312596
H	5.691455	-0.325271	-1.143403
C	7.035823	0.397275	0.299010
O	7.176742	1.415196	0.957790
H	7.927484	-0.131493	-0.094727
H	3.103788	-0.259232	-1.017672
F	-0.943663	-4.045084	-1.782036
F	-1.702925	-3.657895	0.224659
F	0.440688	-3.570064	-0.165104

TS3-1W

E = -1844.780727 au, G = -1844.469922 au,
$\nu^{\ddagger}$ = 1314.8980i cm^{-1}

C	0.460928	2.068406	-0.538335
C	-1.666860	0.291679	1.232428
C	-0.866668	2.308188	-0.128094
C	0.985546	2.688888	-1.678226
C	-1.441034	1.684805	1.104207
C	-2.173800	-0.214640	2.439687
C	-1.633339	3.207528	-0.884179
C	0.189332	3.551155	-2.431996
C	-1.777377	2.521435	2.183408
C	-2.488578	0.631339	3.503493
C	-1.121679	3.819681	-2.029521
H	0.603373	4.028435	-3.314647
C	-2.298586	2.009110	3.372534
H	-2.883126	0.215177	4.425642
H	-2.324014	-1.285704	2.529589
H	2.021627	2.525162	-1.956648
N	-1.306623	-0.626797	0.196876
S	-2.264402	-0.906487	-1.036269
O	-3.191622	0.187543	-1.387362
O	-1.491229	-1.564228	-2.109493
C	-3.458651	-2.288464	-0.527224
F	-2.783230	-3.376585	-0.108742
F	-4.264342	-1.886439	0.475409
F	-4.235377	-2.652607	-1.565926
C	5.326367	-0.714354	0.289168
C	3.985695	-1.416599	0.007810
C	2.783220	-0.661805	0.557349
H	5.322541	0.278755	-0.175849
H	4.009413	-2.421337	0.448683
H	3.865314	-1.558298	-1.074349
H	2.779089	-0.509470	1.641067
C	2.169117	0.331123	-0.206384
H	2.307147	0.335447	-1.285916
N	1.278641	1.214714	0.251818
H	1.068540	1.198317	1.245381

H	-1.742493	4.501469	-2.602310
H	-2.655944	3.403845	-0.577669
H	-1.609392	3.590097	2.085609
H	-2.542531	2.679805	4.190678
H	5.433981	-0.550668	1.368276
H	1.653349	-1.530625	0.496201
C	6.521944	-1.516301	-0.224828
H	6.556572	-2.522113	0.222651
H	6.450476	-1.699777	-1.308423
C	7.871099	-0.898051	0.013861
O	8.068709	0.167796	0.575181
H	8.730472	-1.487865	-0.364978
O	0.647779	-2.222434	0.613900
H	0.665444	-2.939391	-0.039871
H	-0.206161	-1.590371	0.410914

TS3-2W

E = -1921.230443 au, G = -1920.898183 au,
$\nu^{\ddagger}$ = 1154.3186i cm^{-1}

C	0.662549	1.722243	-0.784120
C	-1.515373	0.203285	1.266863
C	-0.472155	2.130209	-0.047759
C	1.013463	2.384989	-1.968617
C	-0.857395	1.458234	1.234650
C	-1.809877	-0.380405	2.511245
C	-1.205783	3.229338	-0.513187
C	0.254971	3.466271	-2.416080
C	-0.553839	2.096344	2.450222
C	-1.494065	0.264811	3.706919
C	-0.854283	3.899705	-1.685880
H	0.540471	3.970563	-3.334106
C	-0.869149	1.514602	3.679227
H	-1.737552	-0.206645	4.654587
H	-2.297493	-1.349900	2.526017
H	1.878490	2.078174	-2.544889
N	-1.787932	-0.522446	0.069543
S	-3.108518	-0.258614	-0.754122
O	-3.750353	1.061409	-0.559759
O	-2.949721	-0.763295	-2.135569
C	-4.447834	-1.428499	-0.090326
F	-4.045367	-2.714108	-0.143253
F	-4.749103	-1.142822	1.193853
F	-5.581364	-1.319479	-0.813498
C	5.654901	-0.698719	0.139603
C	4.578436	-1.388652	-0.718504
C	3.157943	-1.048672	-0.287085
H	5.540209	0.389057	0.062787
H	4.721625	-2.475274	-0.663966
H	4.716692	-1.112137	-1.772192
H	2.893116	-1.335579	0.735546
C	2.527432	0.085412	-0.789265
H	2.885631	0.513168	-1.721513
N	1.435763	0.654699	-0.265084
H	1.105605	0.285448	0.621527
H	-1.442842	4.745332	-2.027363
H	-2.075241	3.546766	0.052808
H	-0.053574	3.060283	2.421750
H	-0.620848	2.028181	4.602988
H	5.499487	-0.953200	1.195004

H	2.321325	-2.034576	-0.964612
C	7.071270	-1.096109	-0.276159
H	7.221508	-2.185070	-0.209507
H	7.264807	-0.856051	-1.333432
C	8.183451	-0.464328	0.513418
O	8.035464	0.319911	1.437091
H	9.205832	-0.759646	0.201106
O	1.679058	-2.954268	-1.360864
H	1.608087	-2.919220	-2.328652
H	-1.046831	-2.012171	-0.186707
O	-0.602694	-2.908478	-0.349905
H	-0.701609	-3.434117	0.455421
H	0.715068	-2.926243	-0.940741

IN4

E = -1768.361458 au, *G* = -1768.068313 au

C	0.432464	1.701736	-0.739156
C	-1.911840	0.523813	1.197112
C	-0.804527	2.257809	-0.321799
C	1.022621	2.147332	-1.933549
C	-1.491467	1.847919	0.939902
C	-2.599973	0.198046	2.368921
C	-1.388875	3.261800	-1.109552
C	0.402620	3.125014	-2.712637
C	-1.792255	2.821637	1.909362
C	-2.904501	1.187866	3.303673
C	-0.805578	3.692732	-2.302532
H	0.879017	3.452726	-3.631932
C	-2.495393	2.503457	3.072313
H	-3.444395	0.928812	4.208778
H	-2.893528	-0.831643	2.540150
H	1.982408	1.749723	-2.242228
N	-1.602321	-0.527704	0.262518
S	-2.670798	-1.054813	-0.881036
O	-3.919857	-0.295188	-0.831283
O	-1.954249	-1.306366	-2.133702
C	-3.119565	-2.777645	-0.257138
F	-2.019358	-3.531113	-0.147137
F	-3.722199	-2.705162	0.936530
F	-3.956458	-3.349705	-1.132637
C	5.652215	-0.682424	0.429082
C	4.286160	-1.316380	0.103894
C	3.125835	-0.450342	0.513396
H	5.748911	0.269696	-0.105616
H	4.220749	-2.286255	0.619787
H	4.224744	-1.532329	-0.970547
H	3.056800	-0.186085	1.569965
C	2.182978	-0.011742	-0.334387
H	2.226336	-0.256214	-1.392318
N	1.028431	0.694114	0.050224
H	0.947198	0.846823	1.050730
H	-1.289585	4.458369	-2.900415
H	-2.333489	3.689638	-0.786836
H	-1.466825	3.843333	1.739634
H	-2.714752	3.280469	3.798087
H	5.700363	-0.443656	1.498542
C	6.819844	-1.597860	0.061533
H	6.750607	-2.568020	0.578503
H	6.810607	-1.856181	-1.009076

C	8.190609	-1.056805	0.356359
O	8.426971	0.029778	0.860191
H	9.028967	-1.728273	0.079836
H	-0.633155	-0.526568	-0.078890

TS4a
E = -1768.343821 au, G = -1768.047007 au,
$v^{\ddagger}$ = 750.1757i cm^{-1}

C	0.969302	2.292420	-0.441037
C	-1.160615	0.354137	1.184044
C	-0.405456	2.419288	-0.138779
C	1.517771	2.975047	-1.537330
C	-1.038876	1.757946	1.044164
C	-1.762848	-0.179909	2.333582
C	-1.186145	3.255620	-0.951612
C	0.711258	3.775647	-2.344306
C	-1.566621	2.575768	2.060361
C	-2.269429	0.649734	3.333725
C	-0.646832	3.924158	-2.050751
H	1.152645	4.296863	-3.188160
C	-2.177562	2.036605	3.192956
H	-2.733743	0.214399	4.213368
H	-1.831454	-1.258540	2.430674
H	2.579545	2.910262	-1.747370
N	-0.591746	-0.546087	0.225671
H	0.461400	-1.268664	0.607495
S	-1.353512	-0.965437	-1.110046
O	-2.347632	0.012970	-1.585589
O	-0.377358	-1.534633	-2.057660
C	-2.425150	-2.471867	-0.697749
F	-1.678337	-3.457624	-0.167483
F	-3.386573	-2.149659	0.187929
F	-3.019012	-2.943708	-1.809487
C	4.860578	-1.960149	-0.101692
C	4.978896	-0.557648	0.548715
C	3.598634	-0.017182	0.870943
C	2.413548	-1.762814	0.236768
C	3.610100	-2.645158	0.455198
H	4.754832	-1.853042	-1.188182
H	5.754749	-2.565312	0.074041
H	5.530531	0.120880	-0.110359
H	5.549905	-0.628399	1.480274
H	3.258904	-0.114013	1.900012
H	2.227051	-1.447754	-0.793828
H	3.704101	-2.876199	1.521528
H	3.412960	-3.588095	-0.075300
C	2.940583	0.894118	0.071255
H	3.308035	1.092994	-0.931298
O	1.390987	-1.827831	1.019739
N	1.785073	1.503988	0.402405
H	1.403615	1.305051	1.321137
H	-1.279074	4.554063	-2.668429
H	-2.242342	3.358660	-0.722839
H	-1.479434	3.653416	1.958060
H	-2.568812	2.695421	3.962101

TS4b
E = -1768.342778 au, G = -1768.043815 au,
$v^{\ddagger}$ = 230.8948i cm^{-1}

C	1.437626	2.072644	-0.503138
C	-0.894790	0.457542	1.200490
C	0.126539	2.404392	-0.093491
C	1.997343	2.656422	-1.646689
C	-0.463821	1.805827	1.145189
C	-1.380713	-0.058492	2.411545
C	-0.585316	3.345810	-0.847260
C	1.254768	3.570256	-2.395045
C	-0.574697	2.599475	2.299838
C	-1.477907	0.743419	3.549791
C	-0.036663	3.923651	-1.994909
H	1.697190	4.017864	-3.279620
C	-1.082655	2.082122	3.493216
H	-1.862519	0.322797	4.474182
H	-1.688925	-1.097950	2.448512
H	3.014677	2.423361	-1.941839
N	-0.780288	-0.392298	0.054232
H	0.439497	-1.183993	-0.336723
S	-1.991841	-0.507698	-0.965211
O	-2.974186	0.595491	-0.908374
O	-1.499596	-0.956431	-2.283312
C	-3.034868	-2.000095	-0.439054
F	-2.276993	-3.110914	-0.357622
F	-3.607932	-1.798493	0.764209
F	-4.017147	-2.228986	-1.332653
C	4.902967	-1.454521	0.360452
C	3.706421	-0.623347	0.744958
C	2.146681	-2.147774	0.121993
C	3.192820	-3.108527	-0.352907
H	5.227063	-1.199763	-0.655720
H	5.749451	-1.259408	1.029314
H	3.423778	-0.623182	1.797062
H	1.839114	-2.183680	1.168964
H	2.782693	-4.119046	-0.210135
H	3.339359	-2.960930	-1.428137
C	3.190627	0.371801	-0.058151
H	3.531679	0.471076	-1.084618
O	1.273321	-1.717876	-0.734913
N	2.163867	1.162260	0.298325
H	1.801576	1.043758	1.239456
C	4.504635	-2.942720	0.421329
H	4.360198	-3.245439	1.465843
H	5.283973	-3.587084	0.003610
H	-0.611819	4.643535	-2.568560
H	-1.590708	3.609757	-0.534471
H	-0.246333	3.634011	2.255684
H	-1.157179	2.716502	4.371119

TS4c

E = -1768.342518 au, G = -1768.042469 au,
$\nu^{\ddagger}$ = 238.3942i cm^{-1}

C	1.558279	1.948355	-0.292655
C	-1.282744	0.785198	1.012852
C	0.197482	2.305166	-0.397941
C	2.450336	2.232165	-1.336147
C	-0.770275	2.070307	0.718482
C	-2.172931	0.628105	2.085739
C	-0.229501	2.949707	-1.568172
C	1.995722	2.852333	-2.498934

C	-1.196603	3.162533	1.492686
C	-2.579815	1.723301	2.849677
C	0.651422	3.215384	-2.617547
H	2.696891	3.068350	-3.299062
C	-2.095229	2.998521	2.549307
H	-3.272368	1.578564	3.673539
H	-2.544623	-0.365491	2.313612
H	3.502773	1.998379	-1.224345
N	-0.829355	-0.361576	0.291161
H	0.249499	-1.195710	0.853282
S	-1.543672	-0.853527	-1.033190
O	-0.611487	-1.703368	-1.804035
O	-2.339304	0.166540	-1.747259
C	-2.867097	-2.113608	-0.532445
F	-3.836248	-1.532949	0.202699
F	-2.330054	-3.109326	0.198630
F	-3.442157	-2.658171	-1.622471
C	4.728284	-1.279439	0.533233
C	3.537390	-0.414732	0.191895
C	2.001293	-2.025774	0.466999
C	3.027887	-3.040526	0.868860
H	4.971246	-1.179987	1.598202
H	5.614258	-0.963276	-0.030100
H	3.344135	-0.254066	-0.863590
H	1.762634	-1.913536	-0.591923
C	2.978821	0.447649	1.115476
H	3.231994	0.312255	2.164484
O	1.042812	-1.756872	1.303816
N	2.028915	1.383061	0.926275
H	1.587087	1.739349	1.765697
C	4.381717	-2.746529	0.218504
H	0.291860	3.706044	-3.516651
H	-1.274922	3.227885	-1.654850
H	-0.807927	4.150562	1.262693
H	-2.406360	3.858320	3.134737
H	3.102500	-3.053145	1.961600
H	2.643097	-4.020855	0.551999
H	4.313415	-2.890211	-0.867115
H	5.149986	-3.432493	0.587673

TS4d

E = -1768.337839 au, G = -1768.040249 au,
$\nu^{‡}$ = 423.2698i cm^{-1}

C	-2.151182	-1.686961	-0.397806
C	0.854538	-0.784256	1.086128
C	-0.801901	-2.084645	-0.333339
C	-2.895504	-1.871605	-1.570632
C	-0.002538	-1.895054	0.917449
C	1.549142	-0.626583	2.294730
C	-0.231264	-2.681828	-1.467880
C	-2.303101	-2.447658	-2.693198
C	-0.114828	-2.828558	1.960332
C	1.425448	-1.563601	3.321836
C	-0.967841	-2.860054	-2.639896
H	-2.886723	-2.588732	-3.597691
C	0.593712	-2.673236	3.153868
H	1.975332	-1.423941	4.247747
H	2.185741	0.241736	2.424939
H	-3.938294	-1.571514	-1.590524

N	0.940516	0.215984	0.062087
H	0.020880	1.256989	-0.057604
S	2.127949	0.207106	-0.996859
O	1.712150	0.930784	-2.213666
O	2.827382	-1.086788	-1.127548
C	3.492484	1.346904	-0.341306
F	4.047258	0.852923	0.782398
F	3.000815	2.568604	-0.062537
F	4.466770	1.484670	-1.260303
C	-3.386840	1.087467	-0.090035
H	-2.897905	0.910634	-1.043956
C	-3.416860	0.063826	0.832395
H	-3.939101	0.215760	1.774629
N	-2.789137	-1.130766	0.754620
H	-2.721460	-1.662774	1.612791
H	-0.501986	-3.319538	-3.506298
H	0.808061	-2.990637	-1.427490
H	-0.763762	-3.690095	1.826951
H	0.492713	-3.410609	3.944363
C	-3.471332	3.567025	-0.057730
C	-4.309015	2.278051	-0.015481
C	-1.568795	2.157705	0.801778
C	-2.368532	3.416073	1.002206
H	-1.407351	1.495926	1.654761
O	-0.692572	2.117490	-0.143094
H	-2.815380	3.384712	2.001316
H	-1.666321	4.258117	0.967424
H	-3.017126	3.692988	-1.047616
H	-4.080517	4.454935	0.139170
H	-4.891506	2.247215	0.913687
H	-5.026148	2.262871	-0.845879

IN5

E = -17 68.369187 au, *G* = -1768.067658 au

C	0.012305	2.072272	-0.748413
C	-1.538806	0.029336	1.319362
C	-1.320819	2.062906	-0.270144
C	0.385678	2.795959	-1.887733
C	-1.803421	1.384876	0.976535
C	-2.114037	-0.515397	2.483030
C	-2.247908	2.830918	-1.002650
C	-0.557128	3.555131	-2.574221
C	-2.627719	2.136298	1.838853
C	-2.945807	0.247343	3.298827
C	-1.881878	3.566212	-2.128919
H	-0.264361	4.110210	-3.459226
C	-3.198041	1.585977	2.984195
H	-3.378403	-0.199136	4.189467
H	-1.896453	-1.545818	2.736622
H	1.406957	2.740425	-2.249884
N	-0.610763	-0.676372	0.521305
H	2.444250	-1.988351	-0.252704
S	-0.682624	-2.183822	0.082613
O	0.632970	-2.577192	-0.479767
O	-1.347381	-3.136479	0.996799
C	-1.801692	-2.236247	-1.443719
F	-3.035542	-1.795306	-1.130222
F	-1.313422	-1.456073	-2.423916
F	-1.908925	-3.490930	-1.918438

C	5.506676	0.122282	1.200966
C	4.375070	1.143803	1.400732
C	3.156589	0.510940	0.684189
C	3.763064	-0.506130	-0.394881
C	5.284917	-0.355555	-0.241561
H	2.584285	-0.111123	1.379111
H	3.428076	-0.257527	-1.408843
C	2.242214	1.502625	0.067183
H	2.638968	2.445426	-0.309097
O	3.394829	-1.842869	-0.082688
N	0.980713	1.273839	-0.073102
H	0.544607	0.335123	0.241312
H	-2.634736	4.134055	-2.666483
H	-3.284186	2.835982	-0.682353
H	-2.812960	3.179875	1.604612
H	-3.823232	2.197949	3.626818
H	5.638725	0.410072	-0.942104
H	5.788135	-1.294930	-0.486708
H	6.496781	0.559528	1.359635
H	5.394928	-0.713137	1.902095
H	4.643618	2.091153	0.916584
H	4.159449	1.360909	2.450579

TS5
E = -1844.799304 au, G = -1844.476354 au,
$\nu^{\ddagger} = 717.0011i$ cm^{-1}

C	0.847798	2.103100	-0.622660
C	-1.201632	0.364769	1.261921
C	-0.405045	2.397743	-0.049048
C	1.263029	2.732087	-1.801478
C	-0.864626	1.738673	1.211330
C	-1.605488	-0.205060	2.477667
C	-1.208812	3.360643	-0.677944
C	0.430907	3.660324	-2.427499
C	-0.980292	2.503688	2.384387
C	-1.709717	0.570112	3.633445
C	-0.804888	3.983491	-1.860214
H	0.759511	4.143846	-3.342071
C	-1.402422	1.932140	3.586150
H	-2.028459	0.110575	4.564148
H	-1.840793	-1.263528	2.505740
H	2.244159	2.517962	-2.213148
N	-1.071009	-0.458144	0.097771
H	2.194591	-2.770654	-0.179189
S	-2.286700	-0.687505	-0.906006
O	-3.368605	0.310354	-0.816157
O	-1.747887	-1.065525	-2.227795
C	4.736264	0.301784	0.337508
C	3.433637	-0.530009	0.245193
C	3.906832	-1.884258	-0.340111
C	5.365125	-2.059370	0.148463
H	5.017239	0.669047	-0.657889
H	4.632627	1.169663	0.994271
H	3.055951	-0.714549	1.260058
H	3.883871	-1.823353	-1.438231
C	2.370160	0.172321	-0.551016
H	2.564738	0.306500	-1.610900
O	3.106578	-2.988132	0.082604
N	1.716025	1.185674	0.040332

H	1.609566	1.132828	1.048429
H	-1.450976	4.715677	-2.334332
H	-2.173343	3.600281	-0.241071
H	-0.725139	3.558796	2.348056
H	-1.480594	2.544953	4.478909
O	1.135253	-1.202211	-0.815655
H	0.976111	-1.301714	-1.769324
H	0.115824	-0.866892	-0.377567
C	-3.148101	-2.285550	-0.372358
C	5.760605	-0.731727	0.842085
F	-4.112466	-2.610231	-1.253037
F	-2.270247	-3.302769	-0.313603
F	-3.716073	-2.148741	0.840295
H	5.418904	-2.911652	0.832106
H	6.023677	-2.278305	-0.697248
H	6.790059	-0.431731	0.626952
H	5.670919	-0.829984	1.929872

IN6

E = -1844.822258 au, *G* = -1844.494952 au,

C	0.877932	1.994769	-0.716656
C	-1.280862	0.483115	1.292468
C	-0.378556	2.357679	-0.176756
C	1.261140	2.518625	-1.964261
C	-0.852045	1.819719	1.136294
C	-1.740285	0.016797	2.527891
C	-1.190828	3.260776	-0.880731
C	0.423736	3.385885	-2.663477
C	-0.909902	2.661020	2.259606
C	-1.797649	0.874497	3.627477
C	-0.806320	3.771744	-2.120840
H	0.744000	3.775459	-3.625484
C	-1.382070	2.200874	3.491000
H	-2.158998	0.505554	4.582131
H	-2.052391	-1.017698	2.618822
H	2.231944	2.263907	-2.376908
N	-1.230324	-0.432096	0.179904
H	3.115301	-3.323584	0.523475
S	-2.500896	-0.692103	-0.817277
O	-3.607381	0.214240	-0.505357
O	-2.023250	-0.896167	-2.187454
C	4.689985	0.358732	0.524456
C	3.475994	-0.564786	0.290917
C	4.121193	-1.833127	-0.305198
C	5.383296	-2.031779	0.537923
H	4.867305	0.970110	-0.367834
H	4.516825	1.049584	1.352641
H	3.028627	-0.842676	1.258048
H	4.392374	-1.644054	-1.351079
H	5.102485	-2.506445	1.487755
H	6.114612	-2.681058	0.048636
C	2.374023	0.022044	-0.590654
H	2.796207	0.316029	-1.557469
O	3.233726	-2.964705	-0.369218
N	1.766739	1.176331	0.016685
H	1.436943	0.978768	0.956059
H	-1.456552	4.457485	-2.654856
H	-2.148709	3.541921	-0.452285
H	-0.579894	3.690414	2.157233

H	-1.419328	2.876031	4.340420
O	1.344510	-0.976217	-0.850472
H	1.747301	-1.866649	-0.789001
H	-0.310256	-0.659089	-0.245367
C	-3.124815	-2.399828	-0.310881
C	5.902654	-0.592593	0.781793
H	6.292853	-0.482356	1.797257
H	6.723513	-0.355747	0.098373
F	-4.146468	-2.747517	-1.106100
F	-2.146838	-3.306788	-0.436822
F	-3.549505	-2.392816	0.960422

IN7

E = -1844.813524 au, *G* = -1844.483411 au,

C	-0.804198	1.328425	0.975424
C	1.729055	0.806859	-1.065549
C	0.345819	2.098768	0.688392
C	-1.534880	1.513138	2.149409
C	1.175742	2.016063	-0.558466
C	2.589190	0.854680	-2.179128
C	0.714159	3.060142	1.650043
C	-1.139792	2.476153	3.079235
C	1.495238	3.221694	-1.211738
C	2.901575	2.067753	-2.788921
C	-0.008396	3.251158	2.826904
H	-1.714825	2.610776	3.989398
C	2.345597	3.259705	-2.314955
H	3.568745	2.076661	-3.646091
H	3.004667	-0.069288	-2.561900
H	-2.417114	0.919697	2.352760
N	1.291423	-0.378311	-0.440876
S	1.969993	-1.793746	-0.424698
O	0.950768	-2.783093	0.020952
O	2.836691	-2.181240	-1.557636
C	3.176117	-1.773300	1.034080
F	4.115571	-0.827569	0.842148
F	2.535172	-1.503493	2.183806
F	3.791191	-2.962734	1.162479
C	-2.091428	-0.886262	0.425894
N	-1.211257	0.307715	-0.005901
H	-0.281938	-0.136972	-0.313058
H	0.318349	3.997215	3.544461
H	1.603950	3.654291	1.470448
H	1.059016	4.145157	-0.843220
H	2.567024	4.205655	-2.799052
H	-1.860143	-1.060226	1.481164
O	-1.729606	-1.970986	-0.369372
H	-0.844798	-2.315178	-0.119889
H	-1.672924	0.742361	-0.824855
H	-3.852813	1.663635	-1.491385
C	-4.551409	-1.658259	0.650946
C	-3.566144	-0.554811	0.202301
C	-3.940037	-0.305710	-1.274411
C	-5.468568	-0.359164	-1.263019
H	-4.089020	-2.643027	0.542620
H	-4.821550	-1.538432	1.703596
H	-3.790645	0.368197	0.751252
H	-3.535924	-1.110536	-1.895450
H	-5.855570	0.588175	-0.863226

H	-5.891471	-0.495807	-2.262102
O	-3.357017	0.895580	-1.814348
C	-5.787083	-1.525462	-0.297405
H	-6.710196	-1.341695	0.258528
H	-5.931294	-2.453873	-0.858268

TS6

E = -1844.801224 au, G = -1844.477923 au,
$v^{\ddagger}$ = 685.8934i cm^{-1}

C	1.017330	2.051794	-0.618950
C	-1.462044	0.588007	1.147201
C	-0.343503	2.327765	-0.376882
C	1.657470	2.543387	-1.762405
C	-1.051746	1.913203	0.877543
C	-2.127450	0.289981	2.344455
C	-1.026054	3.119576	-1.314614
C	0.954794	3.321413	-2.681757
C	-1.351659	2.907777	1.825981
C	-2.413936	1.291275	3.272017
C	-0.394121	3.609046	-2.458765
H	1.461339	3.701331	-3.563283
C	-2.026668	2.607676	3.009758
H	-2.932673	1.042746	4.192722
H	-2.416339	-0.737061	2.539461
H	2.708897	2.324062	-1.923249
N	-1.126035	-0.478455	0.247365
H	4.793281	0.938058	2.022764
S	-2.084127	-0.946336	-0.948918
O	-3.104203	0.052940	-1.302748
O	-1.275191	-1.589640	-1.996135
C	-3.102461	-2.392698	-0.281477
F	-2.296996	-3.359281	0.189454
F	-3.911801	-1.991327	0.714326
F	-3.862157	-2.909963	-1.262177
C	4.791175	-2.805712	0.222158
C	3.746588	-2.281016	-0.800726
C	3.403138	-0.823927	-0.345688
C	4.157770	-0.625599	0.982676
C	5.380624	-1.539667	0.864068
H	4.294410	-3.412931	0.987682
H	5.552415	-3.435576	-0.247060
H	2.858467	-2.917417	-0.827696
H	4.162451	-2.259954	-1.811933
H	3.824404	-0.114301	-1.067345
H	3.528020	-0.949384	1.821768
H	6.110945	-1.066637	0.195094
H	5.868780	-1.723043	1.826704
C	1.902350	-0.589133	-0.283843
H	1.444481	-0.396646	-1.264056
O	4.459322	0.775725	1.129422
N	1.769834	1.212898	0.271293
H	2.730562	1.532766	0.440112
H	-0.951134	4.214229	-3.167049
H	-2.071873	3.348850	-1.135855
H	-1.042310	3.929238	1.625826
H	-2.242082	3.396744	3.723587
O	1.201177	-1.275676	0.561593
H	-0.040423	-0.975341	0.366471
H	1.317987	1.120011	1.181422

PD (= anti-**16**)
E = -385.130079 au, *G* = -385.010285 au

C	1.669223	-0.943754	0.107594
O	2.502429	-0.244348	-0.451283
H	1.409574	1.958428	0.088876
C	-0.851916	-1.495373	0.166074
C	0.286568	-0.474566	0.462143
C	-0.144066	0.828004	-0.237108
C	-1.658664	0.839900	-0.045131
H	-0.557440	-2.204319	-0.614695
H	-1.087707	-2.082689	1.057009
H	0.315282	-0.279463	1.546605
H	0.092053	0.753535	-1.308887
H	-1.878396	1.132475	0.989570
H	-2.163494	1.547330	-0.709738
O	0.464967	2.003790	0.298790
C	-2.062584	-0.627285	-0.300265
H	-2.983874	-0.895951	0.223684
H	-2.243562	-0.784185	-1.368813
H	1.927028	-1.982255	0.396483

(PCM(solv=dimethylsulfoxyde)-ωB97XD/6-31+G**)

TS3-1W
E = -1844.324193 au, *G* = -1844.006899 au,
$\nu^{\ddagger}$ = 1342.6302*i* cm^{-1}

C	0.405522	2.044708	-0.490072
C	-1.701226	0.282744	1.228193
C	-0.932206	2.277442	-0.140821
C	0.960077	2.621221	-1.632274
C	-1.537092	1.674509	1.080641
C	-2.236199	-0.225072	2.415853
C	-1.686554	3.125993	-0.954843
C	0.178448	3.432617	-2.448182
C	-1.957246	2.516238	2.117009
C	-2.635989	0.625191	3.441883
C	-1.145755	3.695370	-2.103443
H	0.613809	3.877010	-3.336925
C	-2.505124	2.003752	3.289556
H	-3.052052	0.210688	4.354521
H	-2.339994	-1.300463	2.518885
H	2.007815	2.461590	-1.866255
N	-1.249564	-0.629052	0.235314
S	-2.085875	-0.918339	-1.063174
O	-2.990302	0.151186	-1.486863
O	-1.223728	-1.556552	-2.060392
C	-3.270667	-2.297225	-0.623564
F	-2.606281	-3.352957	-0.137677
F	-4.143599	-1.890671	0.306589
F	-3.960679	-2.695128	-1.698461
C	5.216661	-0.635335	0.341799
C	3.892284	-1.396220	0.216133
C	2.708765	-0.614134	0.755425
H	5.150960	0.302465	-0.221960
H	3.968215	-2.347679	0.755638
H	3.713558	-1.651487	-0.836246
H	2.730951	-0.391714	1.825591

C	2.073147	0.316909	-0.056090
H	2.181054	0.242960	-1.137250
N	1.209430	1.239289	0.358464
H	1.018783	1.299878	1.352506
H	-1.756227	4.339688	-2.727227
H	-2.723342	3.311326	-0.692850
H	-1.834709	3.589145	2.000545
H	-2.816977	2.676784	4.081421
H	5.381048	-0.358439	1.389466
H	1.602100	-1.499191	0.745617
C	6.399424	-1.452669	-0.160699
H	6.492835	-2.403193	0.384452
H	6.269676	-1.744278	-1.213102
C	7.732258	-0.771996	-0.065373
O	7.913043	0.347776	0.373086
H	8.592455	-1.365076	-0.431155
O	0.627419	-2.213295	0.870412
H	0.688341	-2.957908	0.258135
H	-0.200952	-1.598694	0.579754

TS4a

E = -1767.913009 au, *G* = -1767.608615 au,
$\nu^{\ddagger}$ = 441.7267*i* cm^{-1}

C	0.590698	2.342187	-0.422141
C	-1.226144	0.177333	1.206709
C	-0.776472	2.308312	-0.102742
C	1.039995	3.087485	-1.515205
C	-1.313383	1.573181	1.077297
C	-1.743563	-0.462964	2.333180
C	-1.663599	3.032927	-0.904292
C	0.134602	3.777315	-2.312686
C	-1.960903	2.292827	2.088807
C	-2.372042	0.270303	3.334056
C	-1.224261	3.754700	-2.007850
H	0.499008	4.348945	-3.159608
C	-2.487026	1.652897	3.206915
H	-2.771525	-0.236803	4.205881
H	-1.647805	-1.540654	2.413257
H	2.099622	3.161209	-1.732466
N	-0.522767	-0.598680	0.236750
H	0.442216	-1.063239	0.575146
S	-1.152441	-1.081989	-1.145825
O	-2.242071	-0.225283	-1.589464
O	-0.071032	-1.450780	-2.049560
C	-1.985274	-2.712447	-0.778397
F	-1.111965	-3.564312	-0.238201
F	-2.993313	-2.534524	0.078805
F	-2.468044	-3.245943	-1.901625
C	5.078474	-1.365802	-0.058388
C	4.963670	0.108080	0.390459
C	3.522785	0.382043	0.789384
C	2.657450	-1.358531	0.378523
C	3.947528	-2.119630	0.627831
H	4.949572	-1.433571	-1.144842
H	6.057522	-1.790248	0.176751
H	5.297502	0.786417	-0.400320
H	5.605387	0.291399	1.256987
H	3.300586	0.348716	1.854262
H	2.445902	-1.230537	-0.695607

H	4.109535	-2.204810	1.707845
H	3.822849	-3.130569	0.222032
C	2.703247	1.184498	0.012806
H	2.971265	1.371061	-1.023721
O	1.640067	-1.513815	1.148438
N	1.514254	1.649090	0.398239
H	1.217883	1.447821	1.347002
H	-1.935397	4.297824	-2.620831
H	-2.721140	3.003105	-0.661841
H	-2.037146	3.371910	1.996535
H	-2.977683	2.234734	3.980102

TS4b
E = -1767.909919 au, *G* = -1767.605810 au,
$v^{\ddagger}$ = 334.6392*i* cm^{-1}

C	1.334301	2.056469	-0.512738
C	-0.868251	0.395761	1.236762
C	0.022476	2.345591	-0.097187
C	1.845576	2.614478	-1.684307
C	-0.506742	1.754939	1.166744
C	-1.277872	-0.146786	2.456556
C	-0.750908	3.207415	-0.873467
C	1.046243	3.453545	-2.455530
C	-0.603431	2.543481	2.317659
C	-1.363662	0.649192	3.595694
C	-0.251815	3.757312	-2.052174
H	1.450712	3.881765	-3.366791
C	-1.035931	2.001246	3.525730
H	-1.688099	0.212326	4.534712
H	-1.533485	-1.200121	2.500158
H	2.867305	2.418685	-1.990765
N	-0.759420	-0.434161	0.084127
H	0.459457	-1.073115	-0.381823
S	-1.967506	-0.518682	-0.923738
O	-2.952636	0.558252	-0.802674
O	-1.488811	-0.895557	-2.254594
C	-2.946112	-2.030882	-0.425754
F	-2.160348	-3.113292	-0.398030
F	-3.482324	-1.873913	0.790786
F	-3.940214	-2.256591	-1.292569
C	4.916327	-1.310618	0.384840
C	3.701058	-0.512136	0.765622
C	2.183014	-2.000333	0.029846
C	3.251453	-2.918669	-0.469845
H	5.270172	-1.007823	-0.607222
H	5.735072	-1.139651	1.090584
H	3.397432	-0.538021	1.811429
H	1.858353	-2.092191	1.069041
H	2.838337	-3.934152	-0.410816
H	3.436702	-2.694246	-1.524943
C	3.159517	0.455547	-0.039509
H	3.501979	0.557695	-1.065511
O	1.326539	-1.541251	-0.812259
N	2.105340	1.208563	0.309690
H	1.754139	1.091297	1.253347
C	4.524721	-2.795929	0.360544
H	4.338660	-3.148169	1.381884
H	5.320513	-3.416794	-0.058280
H	-0.871476	4.418033	-2.648964

H	-1.764415	3.427086	-0.553848
H	-0.323473	3.591309	2.260446
H	-1.103746	2.629064	4.408077

TS4c
E = -1767.911807 au, *G* = -1767.605999 au,
$\nu^{\ddagger}$ = 344.7211*i* cm^{-1}

C	1.297774	2.037467	-0.278448
C	-1.364874	0.646758	1.041032
C	-0.078925	2.290216	-0.385333
C	2.161084	2.392484	-1.317873
C	-1.020593	1.973333	0.725933
C	-2.231011	0.390546	2.106161
C	-0.558545	2.894875	-1.549692
C	1.658741	2.970544	-2.478132
C	-1.584696	3.013997	1.471033
C	-2.777038	1.436173	2.845466
C	0.294187	3.225039	-2.597849
H	2.340046	3.239214	-3.278712
C	-2.459157	2.753642	2.523546
H	-3.450390	1.220224	3.668747
H	-2.472701	-0.639764	2.345648
H	3.229225	2.244726	-1.207774
N	-0.767037	-0.437461	0.342451
H	0.314911	-1.128732	0.953257
S	-1.333128	-0.947974	-1.030897
O	-0.281149	-1.661293	-1.760540
O	-2.178481	0.004602	-1.751656
C	-2.524055	-2.327529	-0.619491
F	-3.568373	-1.858049	0.073933
F	-1.921059	-3.266119	0.118722
F	-2.987657	-2.903532	-1.734384
C	4.751854	-0.852688	0.477258
C	3.481843	-0.104026	0.167767
C	2.103466	-1.825461	0.572447
C	3.232331	-2.720998	0.967687
H	5.033359	-0.698255	1.525401
H	5.578813	-0.485045	-0.138601
H	3.230971	0.015003	-0.881699
H	1.826702	-1.760888	-0.482377
C	2.856797	0.681430	1.103281
H	3.145812	0.569637	2.145511
O	1.163253	-1.607275	1.424865
N	1.807632	1.506496	0.934247
H	1.340628	1.814591	1.776657
C	4.511452	-2.347577	0.228520
H	-0.102857	3.682091	-3.498097
H	-1.623414	3.086201	-1.632880
H	-1.322405	4.038495	1.223382
H	-2.882271	3.575277	3.092127
H	3.364382	-2.665029	2.052847
H	2.914087	-3.742508	0.721317
H	4.389933	-2.533732	-0.845053
H	5.352061	-2.955488	0.572125

TS4d
E = -1767.906753 au, *G* = -1767.604526 au,
$\nu^{\ddagger}$ = 870.9685*i* cm^{-1}

C	-2.102241	-1.691880	-0.405617

C	0.811124	-0.701222	1.130108
C	-0.757304	-2.064259	-0.290421
C	-2.798351	-1.891289	-1.598931
C	-0.016197	-1.826685	0.981283
C	1.441695	-0.460792	2.352602
C	-0.132576	-2.655417	-1.392318
C	-2.154620	-2.457954	-2.692475
C	-0.167587	-2.703892	2.058943
C	1.279991	-1.341044	3.418586
C	-0.819633	-2.847361	-2.586648
H	-2.698498	-2.608318	-3.619122
C	0.477683	-2.470253	3.270716
H	1.778610	-1.142381	4.361696
H	2.055300	0.426582	2.462367
H	-3.842485	-1.601510	-1.659839
N	0.930213	0.233773	0.058042
H	0.057751	1.197656	-0.111371
S	2.120546	0.149536	-0.977499
O	1.724958	0.777076	-2.236807
O	2.804042	-1.143113	-0.995865
C	3.440644	1.318529	-0.358480
F	3.960225	0.885928	0.795474
F	2.928891	2.536703	-0.157415
F	4.429862	1.416598	-1.251889
C	-3.310955	1.064207	-0.203348
H	-2.794660	0.852697	-1.135483
C	-3.399642	0.064988	0.737480
H	-3.958714	0.248724	1.652336
N	-2.782260	-1.128839	0.713900
H	-2.779091	-1.662419	1.571513
H	-0.314152	-3.299762	-3.433494
H	0.910134	-2.942645	-1.309126
H	-0.800249	-3.579326	1.941023
H	0.348862	-3.163605	4.095332
C	-3.393227	3.532800	-0.108800
C	-4.236007	2.254145	-0.191903
C	-1.582346	2.044405	0.731961
C	-2.378862	3.290525	1.007613
H	-1.399497	1.352902	1.560841
O	-0.703873	2.069198	-0.201927
H	-2.895766	3.171888	1.965145
H	-1.666172	4.117189	1.100992
H	-2.868386	3.707380	-1.054409
H	-4.008132	4.412947	0.097421
H	-4.903498	2.202346	0.676274
H	-4.867121	2.258881	-1.086544

References

(1) T. Furuta, M. Nikaido, J. Yamamoto, T. Kuribayashi, T. Kawabata, *Synthesis,* 2013, **45**, 1312.

(2) T. Takahashi, H. H. O. Schmid, *Chem. Phys. Lipids,* 1969, 185.

(3) D. Buisson, R. Azerad, *Tetrahedron: Asymmetry* 1996, **7**, 9.

(4) (a) M. Markert, U. Scheffler, R. Mahrwald, *J. Am. Chem. Soc.,* 2009, **131**, 16642; (b) U. Scheffler, R. Mahrwald, *J. Org. Chem.,* 2012, **77**, 2310.

(5) K. Clinch, G. B. Evans, G. W. J. Fleet, R. H. Furneaux, S. W. Johnson, D. H. Lenz, S. P. H. Mee, P. R. Rands, V. L. Schramm, E. A. Taylor Ringia, P. C. Tyler, *Org. Biomol. Chem.,* 2006, **4**, 1131.

(6) *cis*-3-Hydroxy-L-proline was obtained from KYOWA HAKKO BIO CO., LTD. The authors thank KYOWA HAKKO BIO CO., LTD. for generously providing this compound.

(7) R. P. Philp, A. V. Robertson, *Aust. J. Chem.* 1977, **30**, 131.

(8) Y. Asahina, M. Takei, T. Kimura, Y. Fukuda, *J. Med. Chem.* 2008, **51**, 3238.

(9) S. Beligny, S. Eibauer, S. Maechling, S. Blechet, *Angew. Chem. Int. Ed.* 2006, **45**, 1900.

(10) D. Wang, W. A. Nugent, *J. Org. Chem.* 2007, **72**, 7307.

(11) L. F. Solares, I. Lavandera, V. Gotor-Fernández, R. Brieva, V. Gotor, *Tetrahedron* 2006, **62**, 3284.

(12) (a) S. Furegati, W. Ganci, F. Gorla, U. Ringeisen, P. Rüedi, *Helv. Chem. Acta* 2004, **57**, 2629; (b) H. J. M. Gijsen, M. J. A. De Cleyn, C. J. Love, M. Surkyn, S. F. A. Van Brandt, M. G. C. Verdonck, L. moens, J. Cuypers, J.-P. R. M. A. Bosmans, *Tetrahedron,* 2008, **64**, 2456; (c) P. Lorenzetto, M. Wächter, P. Rüedi, *Helv. Chem. Acta* 2011, **94**, 746.

(13) M. Matsugi, D. P. Curran, *J. Org. Chem.* 2005, **70**, 1636.

(14) A. Renard, J. Lhomme, M. Kotera, *Bull. Chem. Soc. Jpn.* 2002, **75**, 1771.

(15) Physical data for **S22** as H-form was reported, see, N. A. Heaps, C. D. Poulter, *J. Org. Chem.* 2011, **76**, 1838.

(16) Physical data for **S24** and **S25** as H-form was reported, see, C. Che, W. Li, S. Lin, J. Chen, J. Zheng, J-c. Wu, Q. Zheng, G. Zhang, Z. Yang, B. Jiang, *Chem. Commun.* 2009, 5990.

(17) Physical data for **S10-D**, see, Ref. 9.

(18) Gaussian 09, Revision D.01, M. J. Frisch, G. W. Trucks, H. B. Schlegel, G. E. Scuseria, M. A. Robb, J. R. Cheeseman, G. Scalmani, V. Barone, B. Mennucci, G. A. Petersson, H. Nakatsuji, M. Caricato, X. Li, H. P. Hratchian, A. F. Izmaylov, J. Bloino, G. Zheng, J. L. Sonnenberg, M. Hada, M. Ehara, K. Toyota, R. Fukuda, J. Hasegawa, M. Ishida, T. Nakajima, Y. Honda, O. Kitao, H. Nakai, T. Vreven, J. A. Montgomery Jr., J. E. Peralta, F. Ogliaro, M. Bearpark, J. J. Heyd, E. Brothers, K. N. Kudin, V. N. Staroverov, T. Keith, R. Kobayashi, J. Normand, K. Raghavachari, A. Rendell, J. C. Burant, S. S. Iyengar, J. Tomasi, M. Cossi, N. Rega, J. M. Millam, M. Klene, J. E. Knox, J. B. Cross, V. Bakken, C. Adamo, J. Jaramillo, R. Gomperts, R. E. Stratmann, O. Yazyev, A. J. Austin, R. Cammi, C. Pomelli, J. W. Ochterski, R. L. Martin, K. Morokuma, V. G. Zakrzewski, G. A. Voth, P. Salvador, J. J. Dannenberg, S. Dapprich, A. D. Daniels, O. Farkas, J. B. Foresman, J. V. Ortiz, J. Cioslowski, D. J. Fox, Gaussian, Inc., Wallingford CT, 2013.

(19) CYLview, 1.0b, Legault, C. Y. Université de Sherbrooke, 2009, http://www.cylview.org.

(20) F. R. Clemente, K. N. Houk, *Angew. Chem. Int. Ed.* 2004, **43**, 5766.

NMR spectra of (*R*)-S**2**

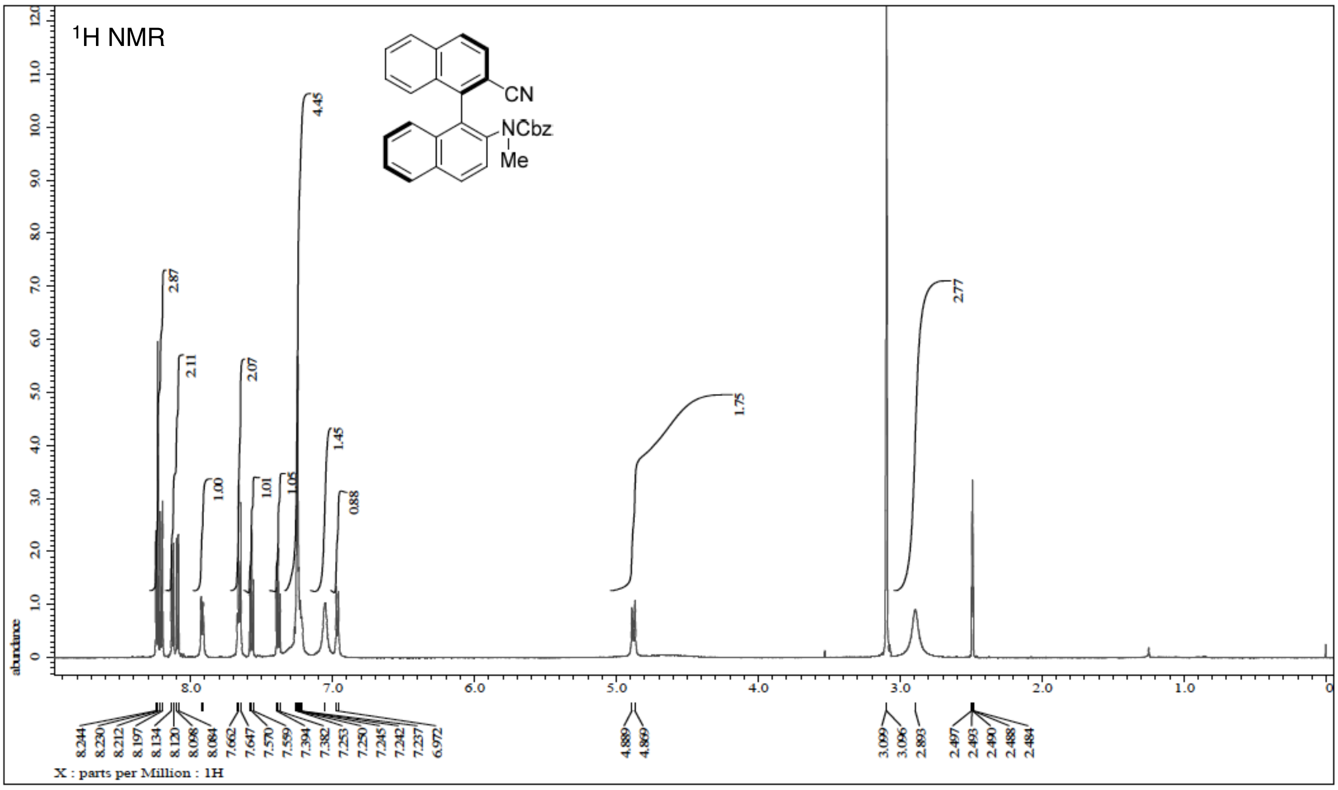

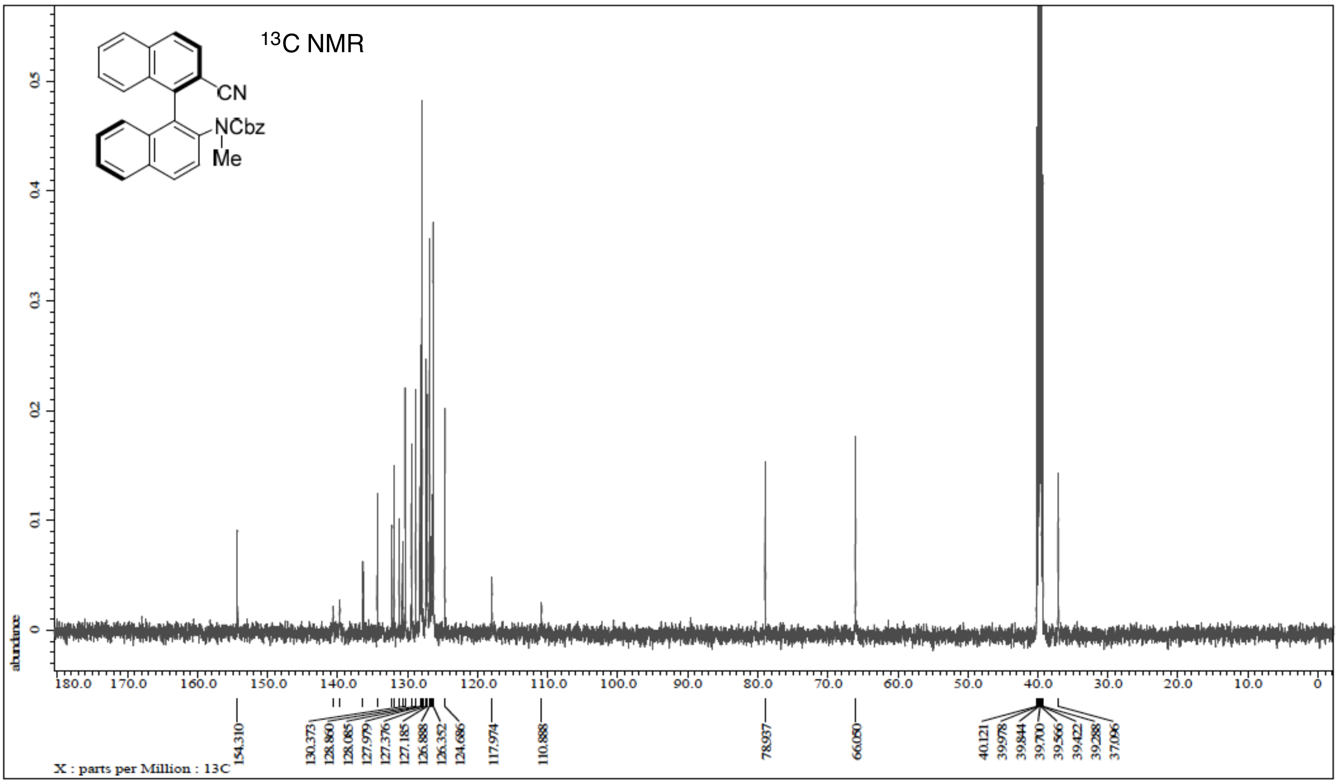

NMR spectra of (*R*)-**11**

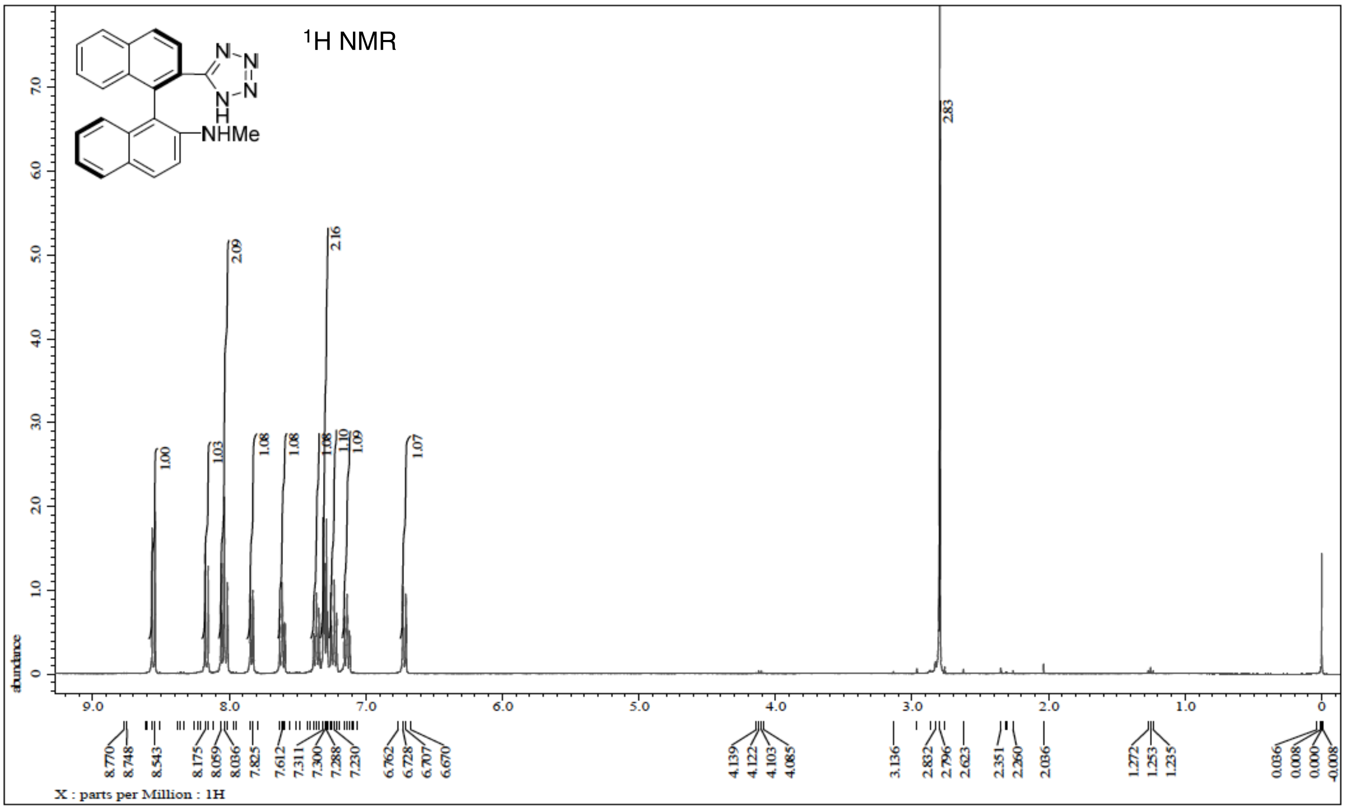

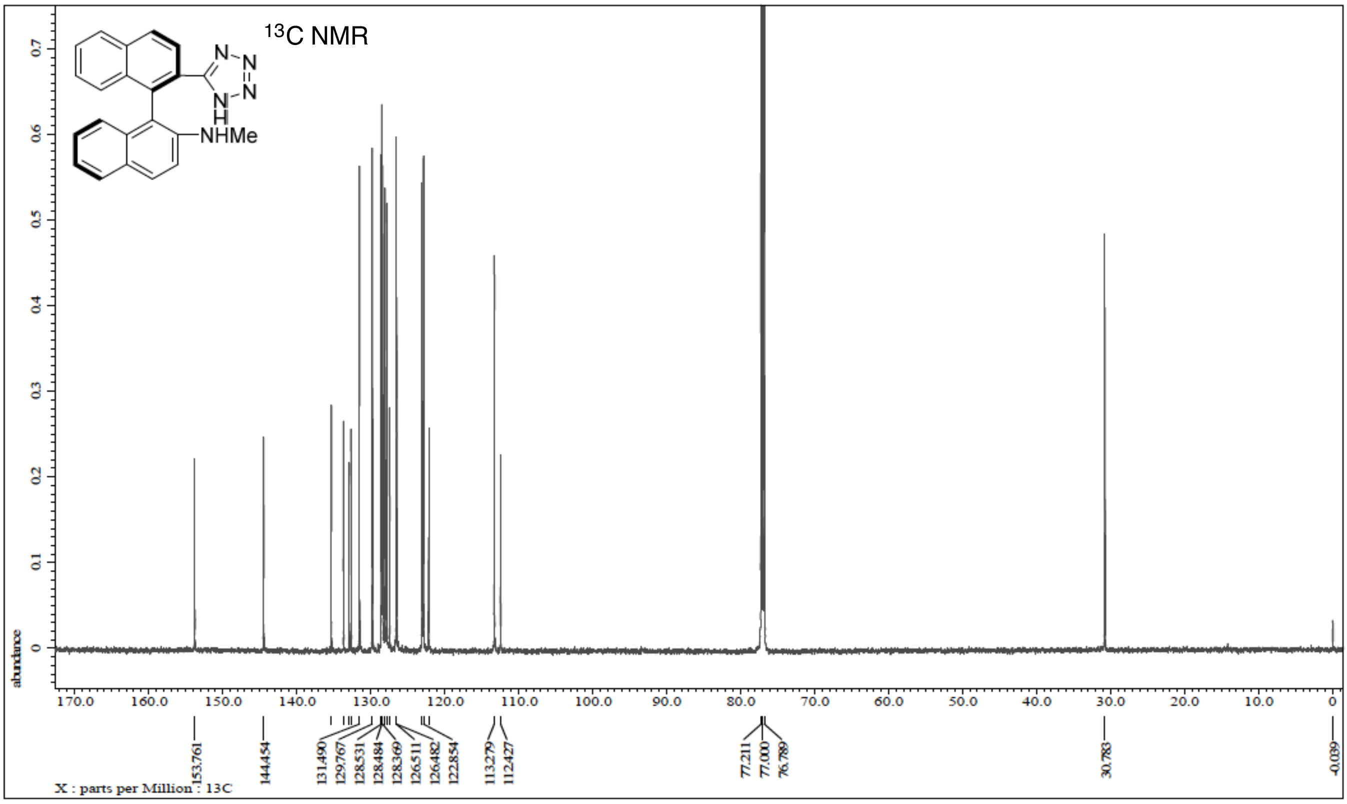

NMR spectra of (*R*)-**12a**

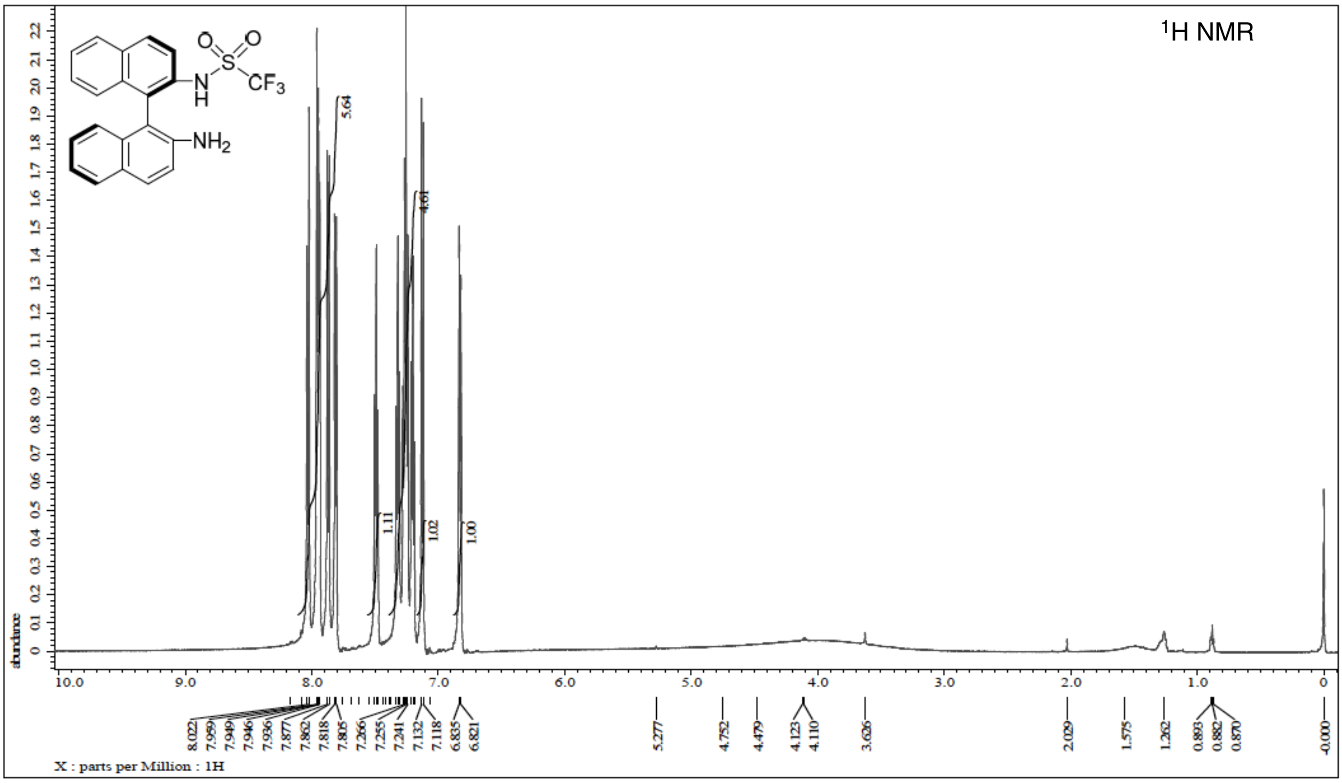

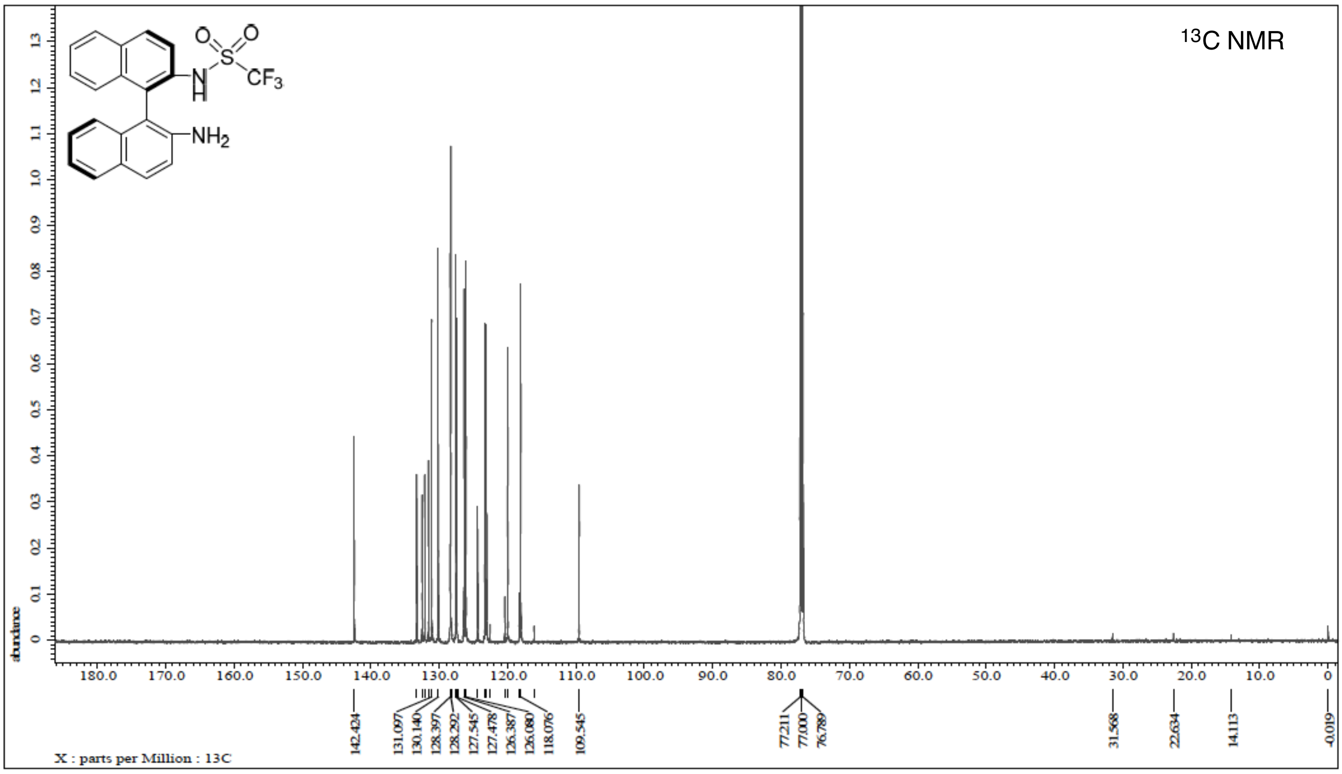

NMR spectra of (*R*)-**12b**

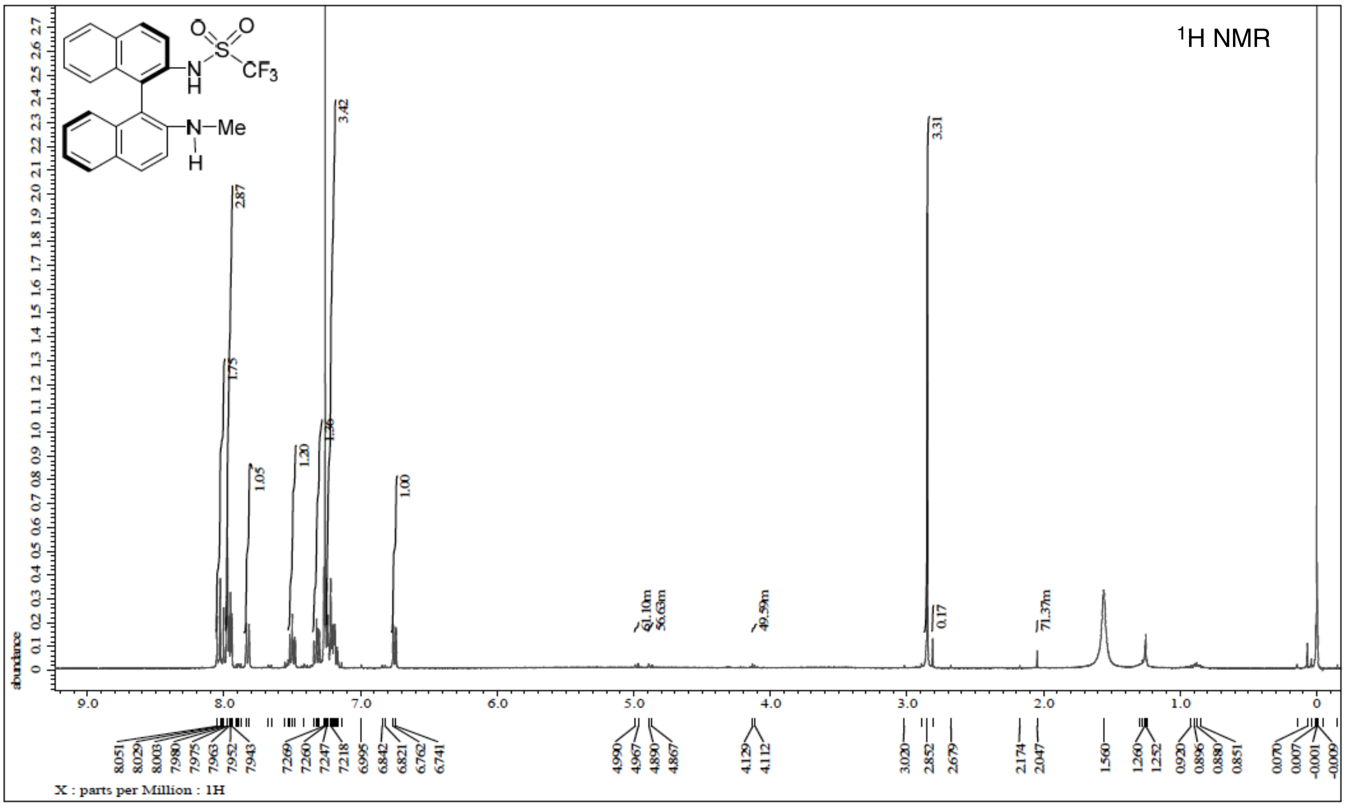

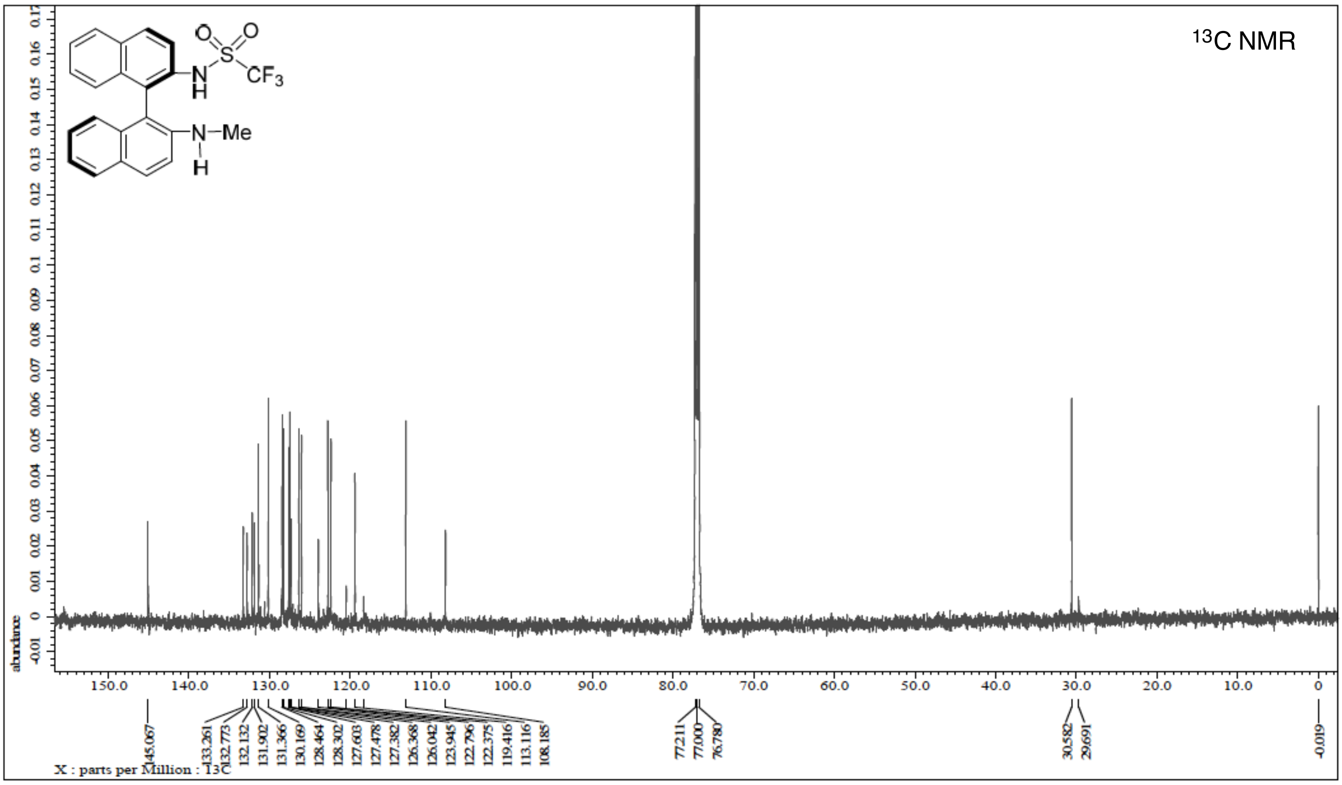

NMR spectra of (*R*)-**12c**

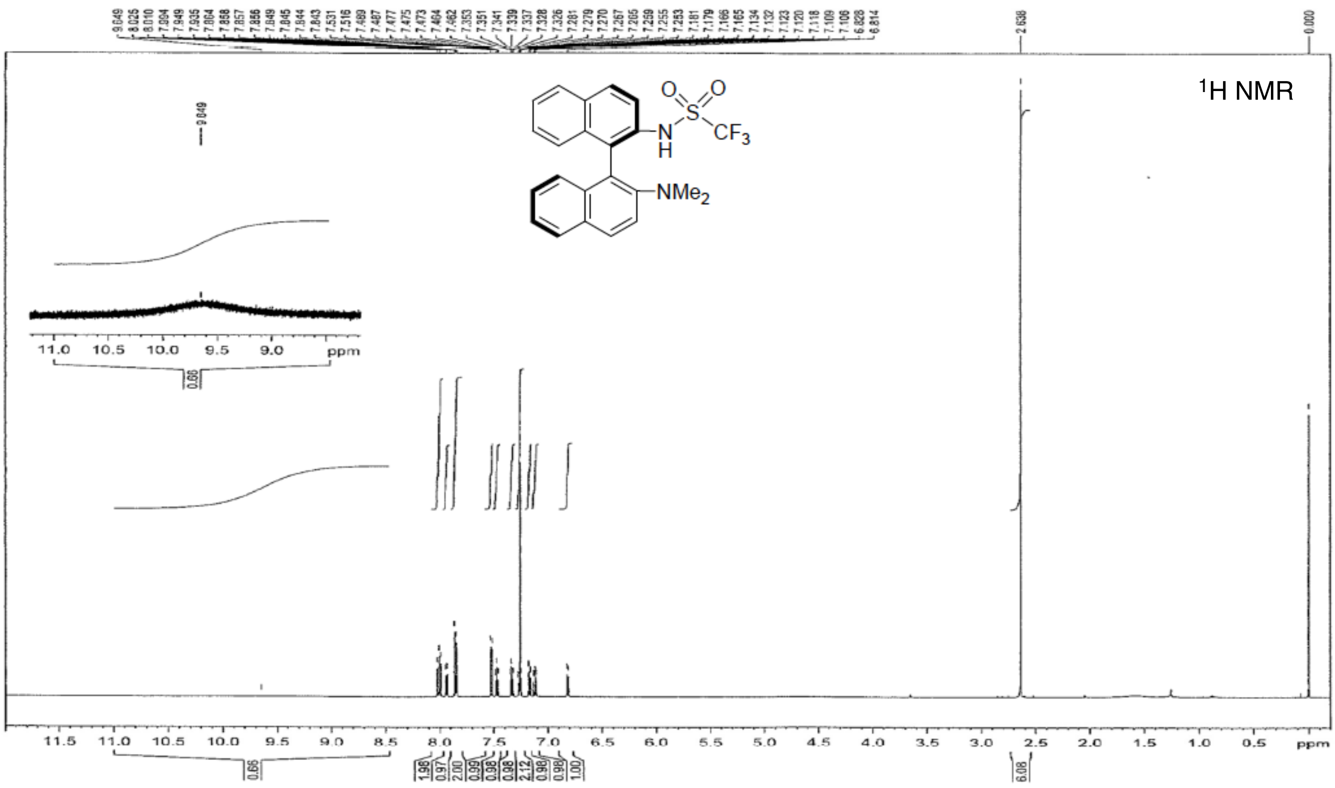

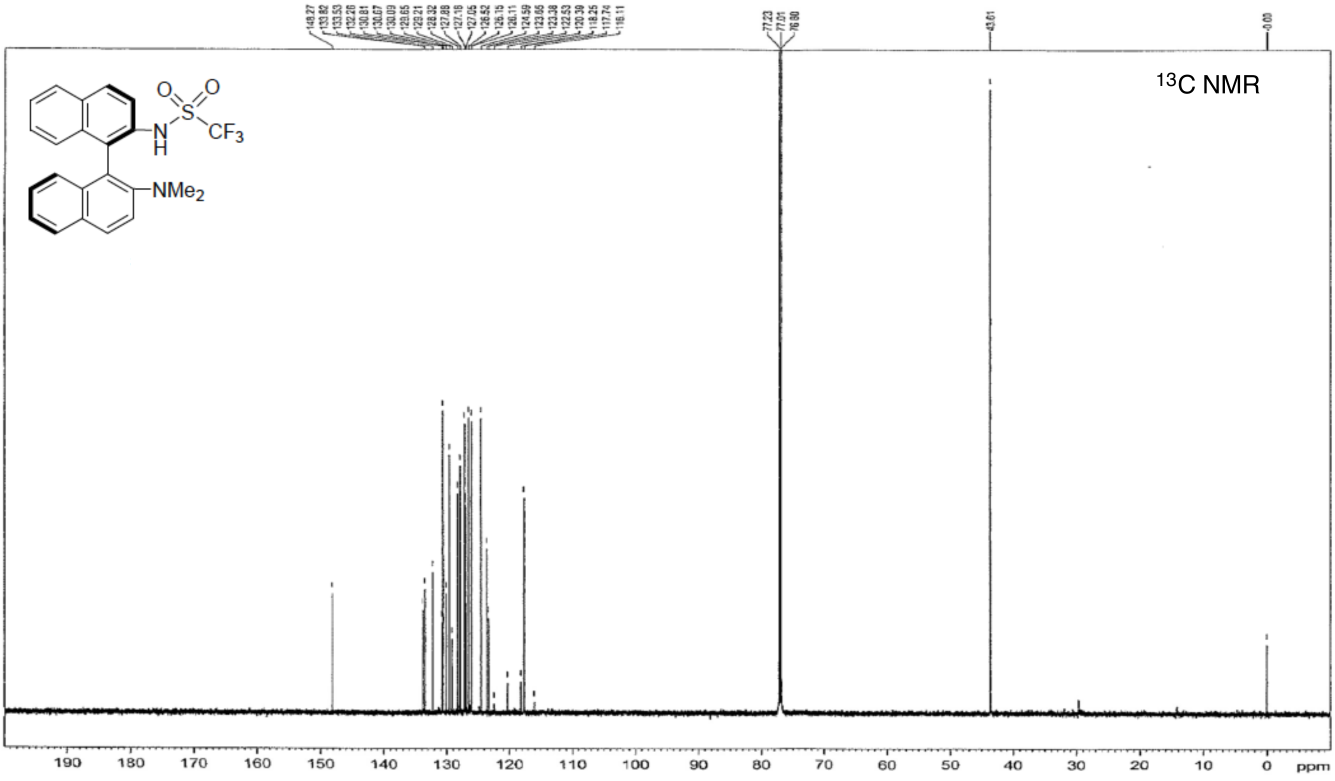

NMR spectra of (*R*)-**13**

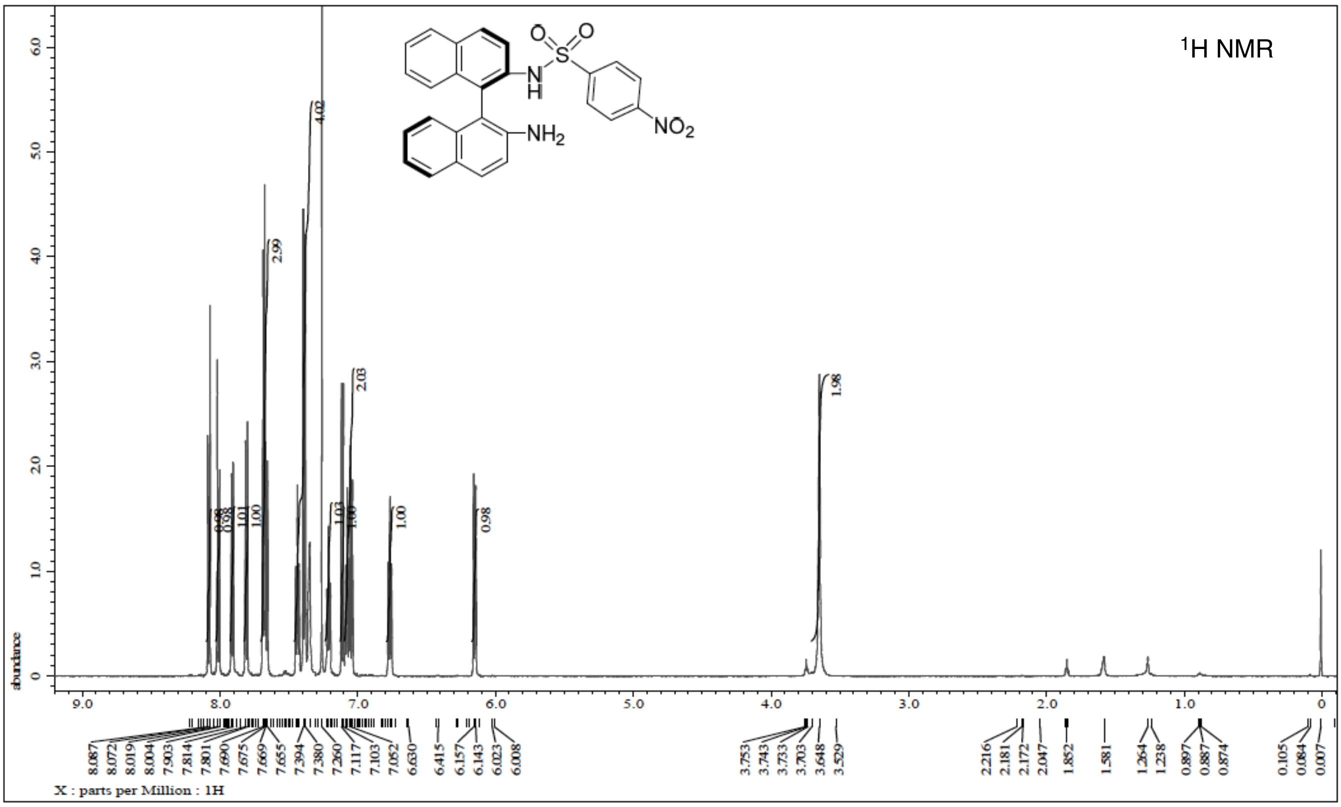

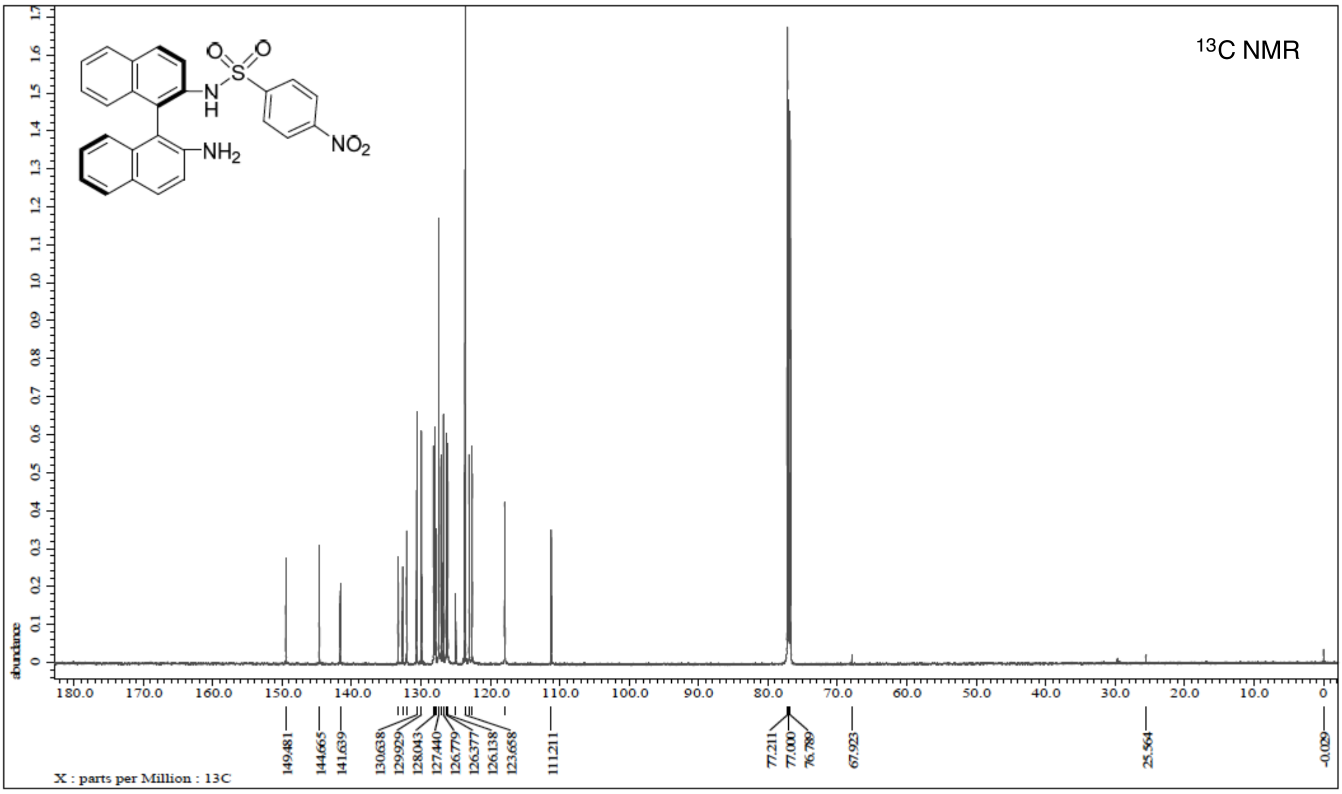

NMR spectra of *anti*-(*E*)-**18**

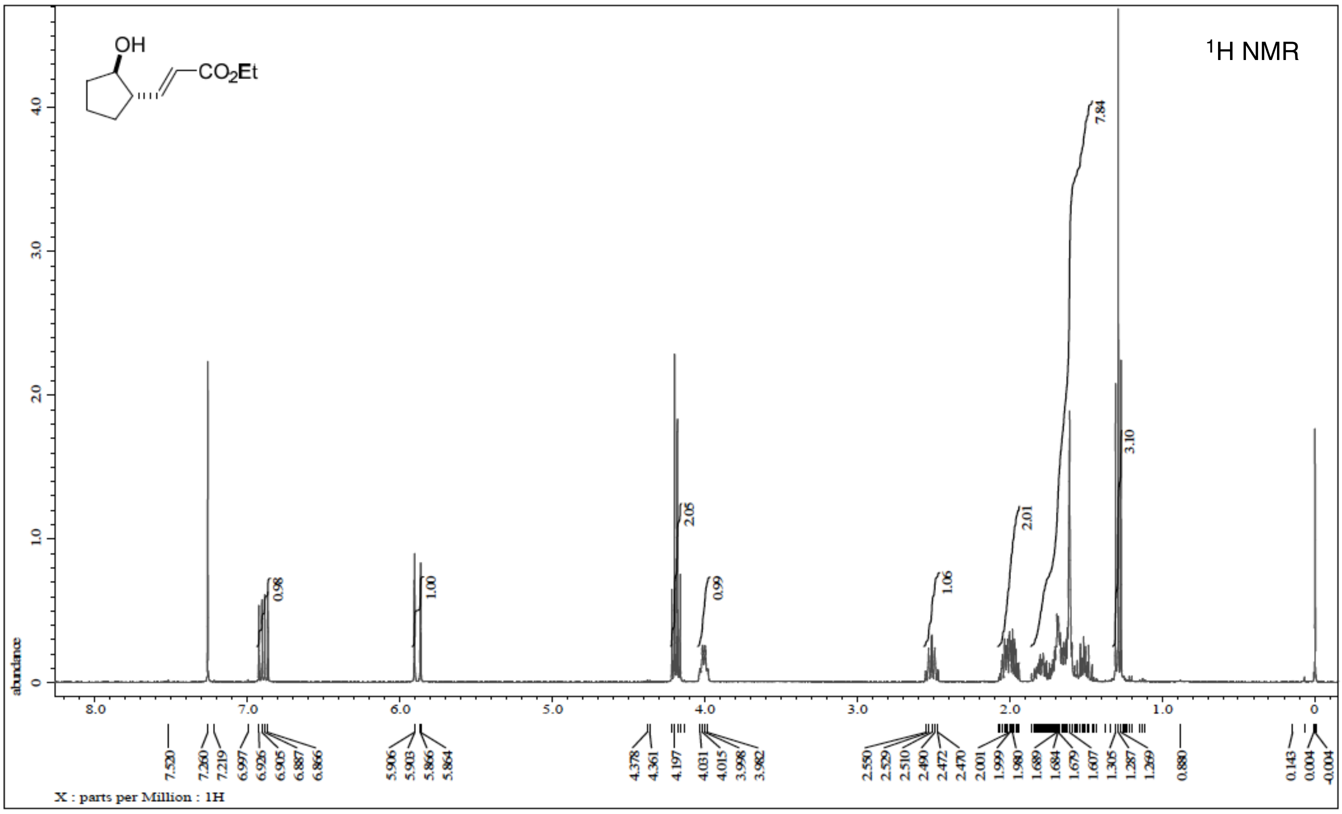

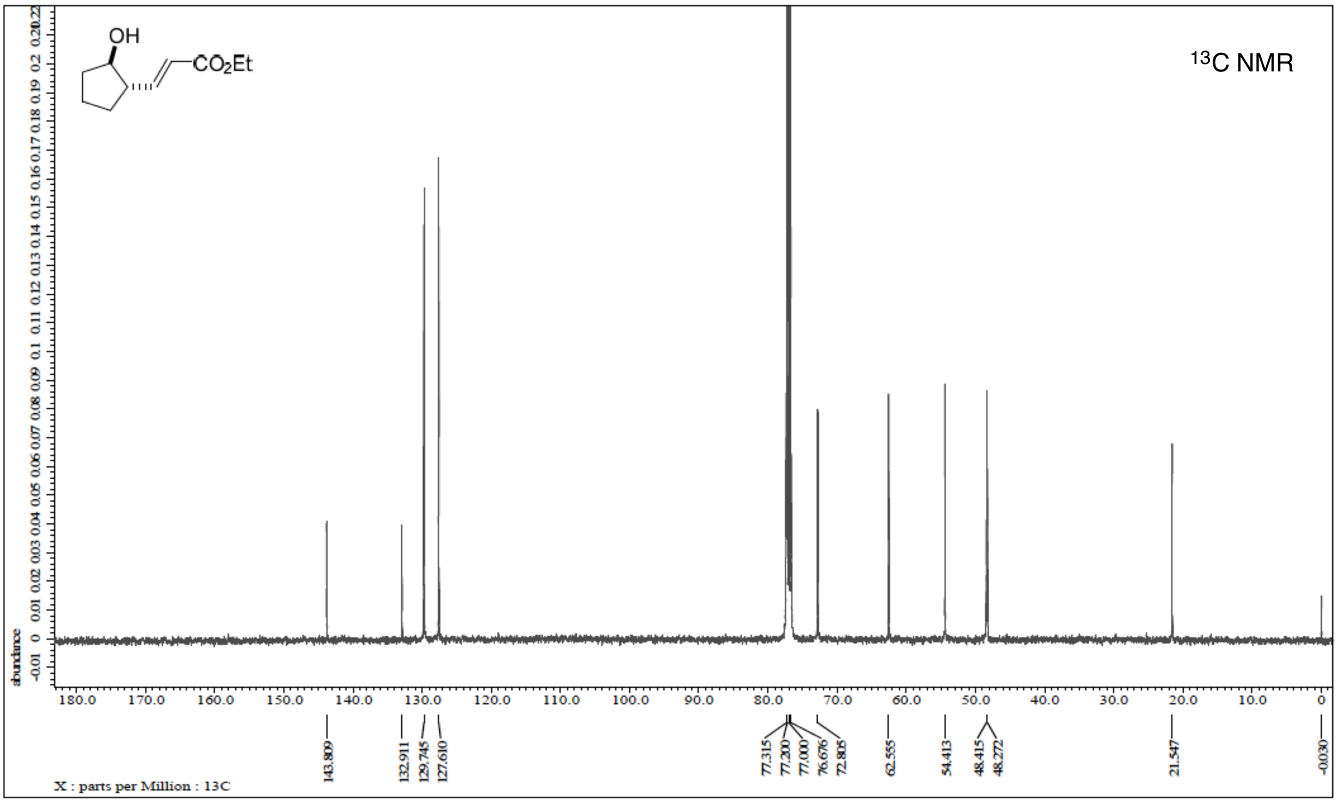

NMR spectra of *anti*-(*Z*)-**18**

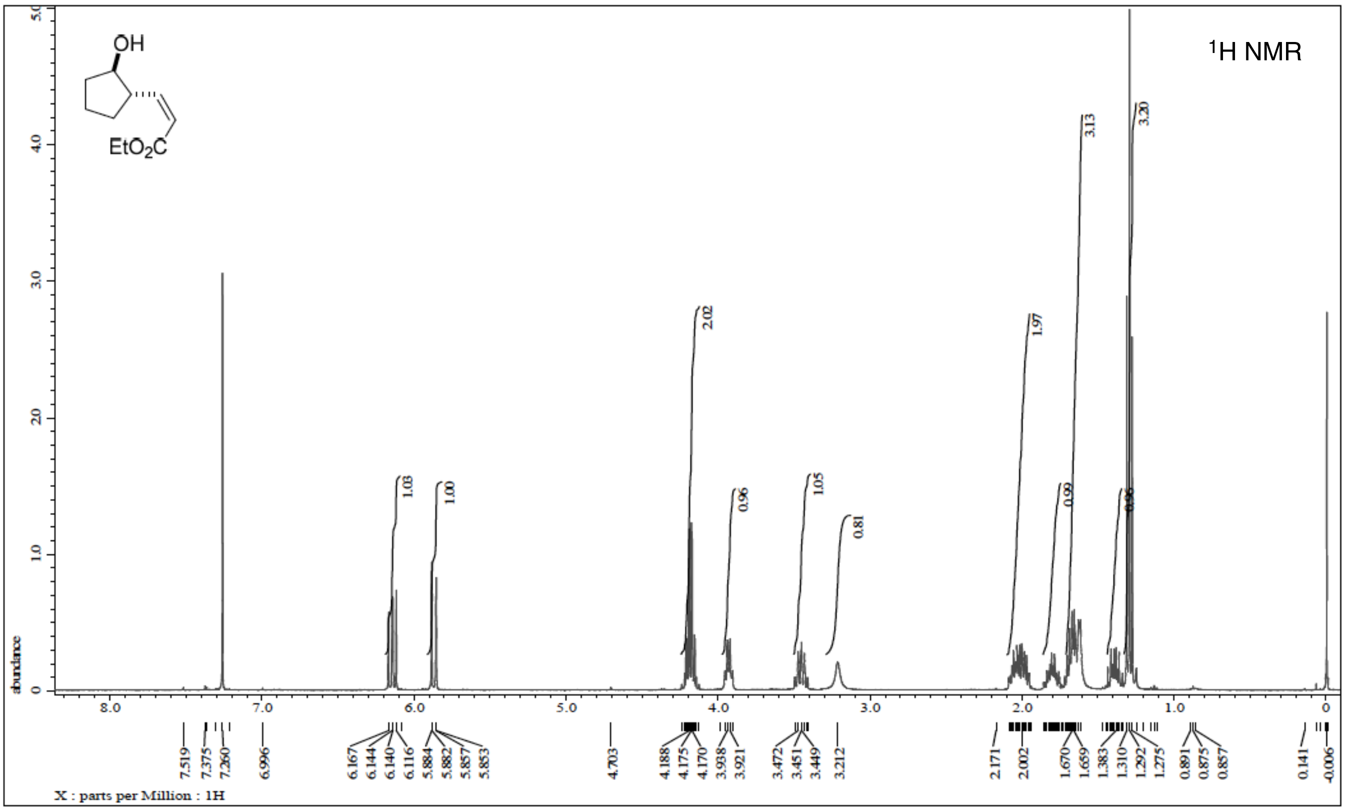

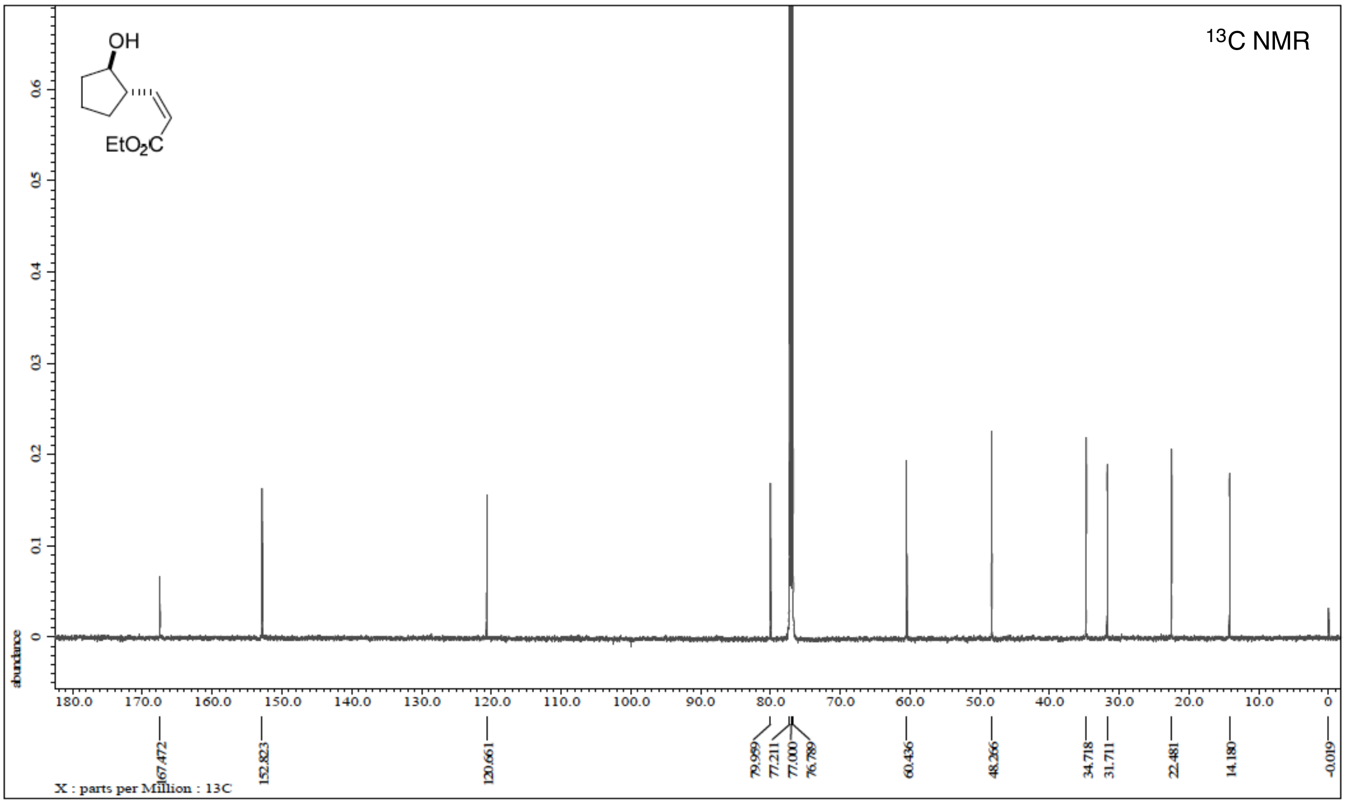

NMR spectra of *anti*-(*E*)-**19**

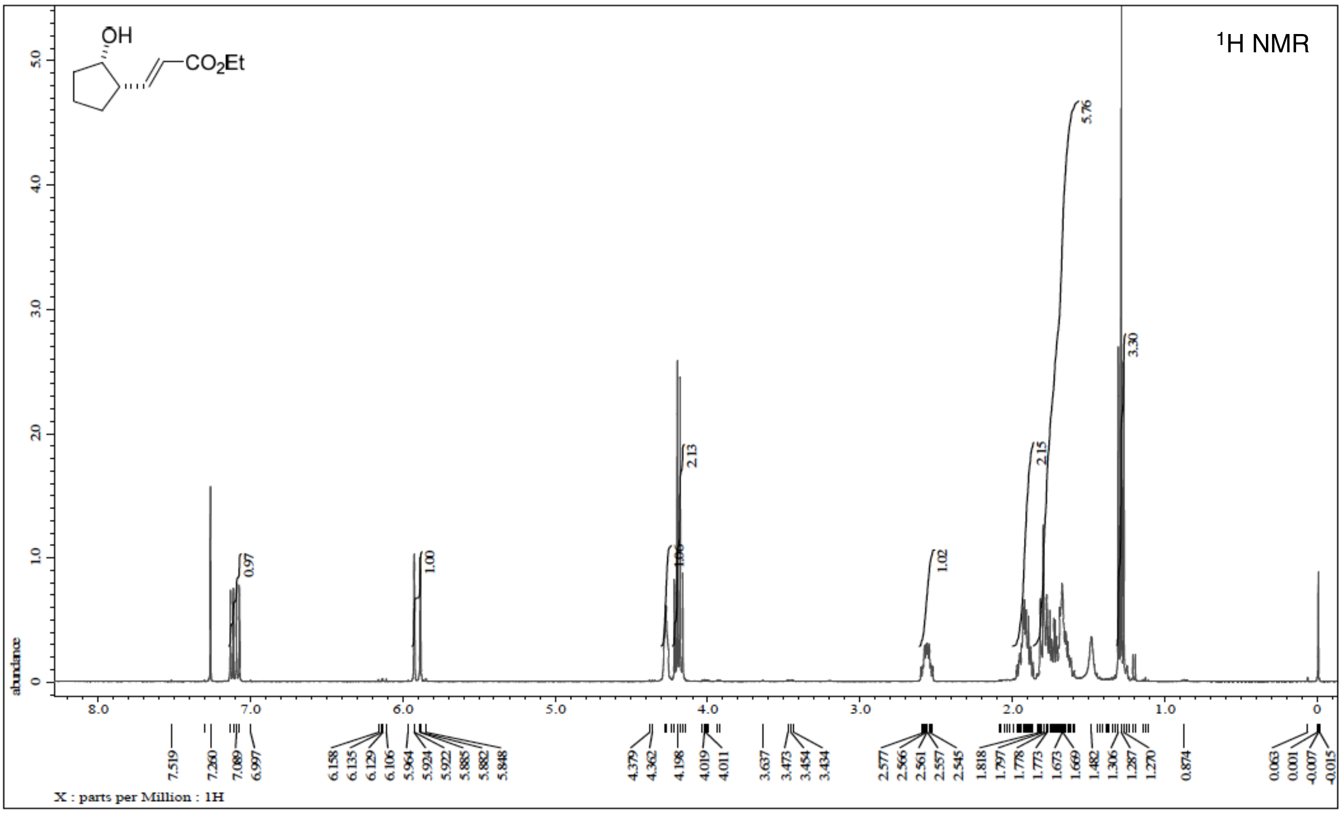

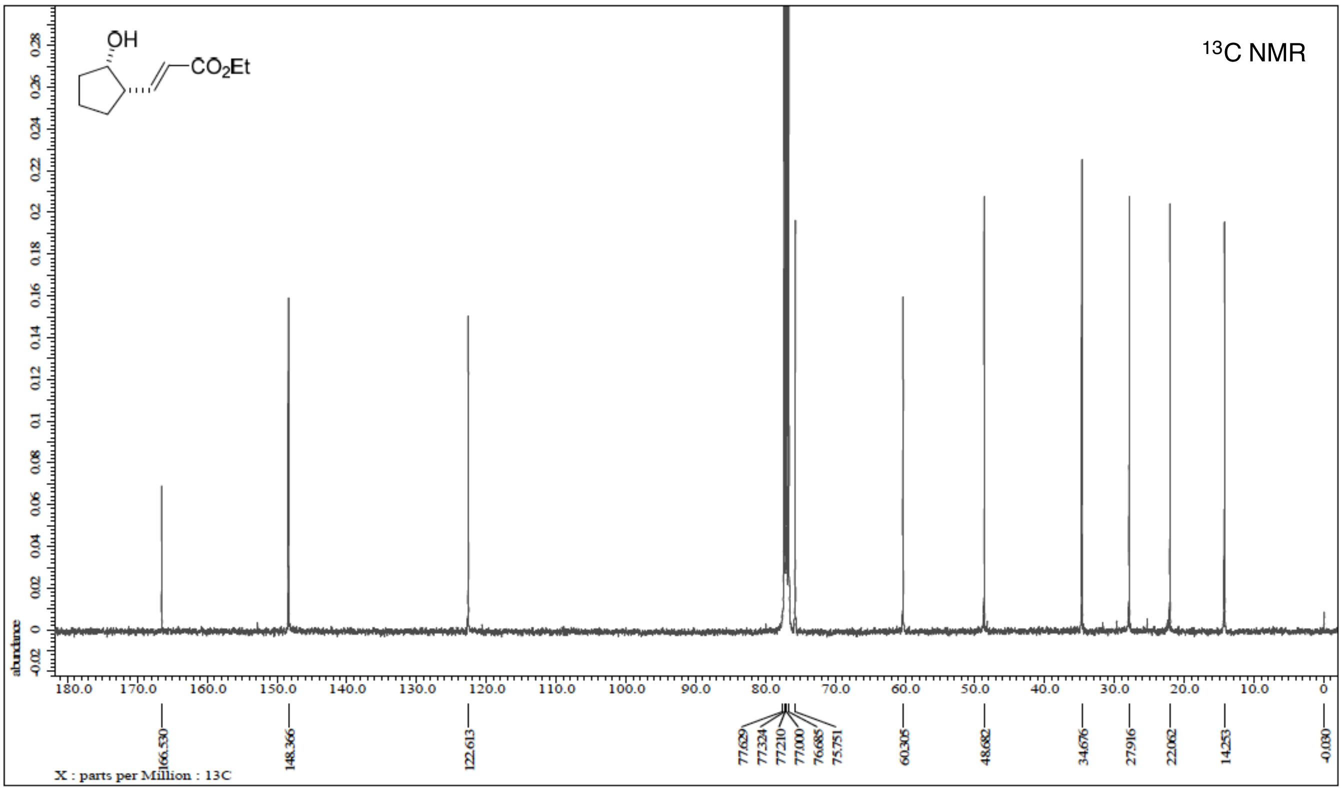

NMR spectra of **S3**

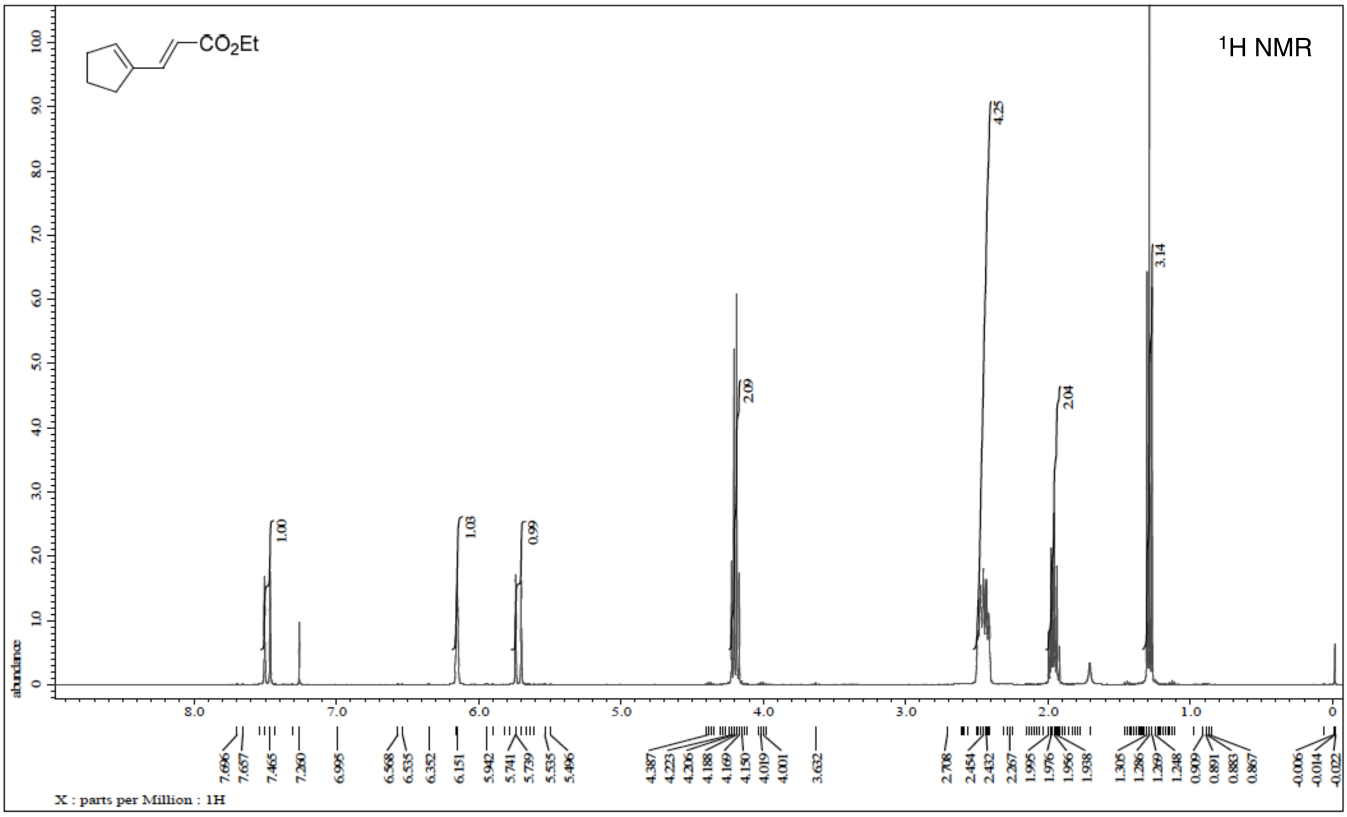

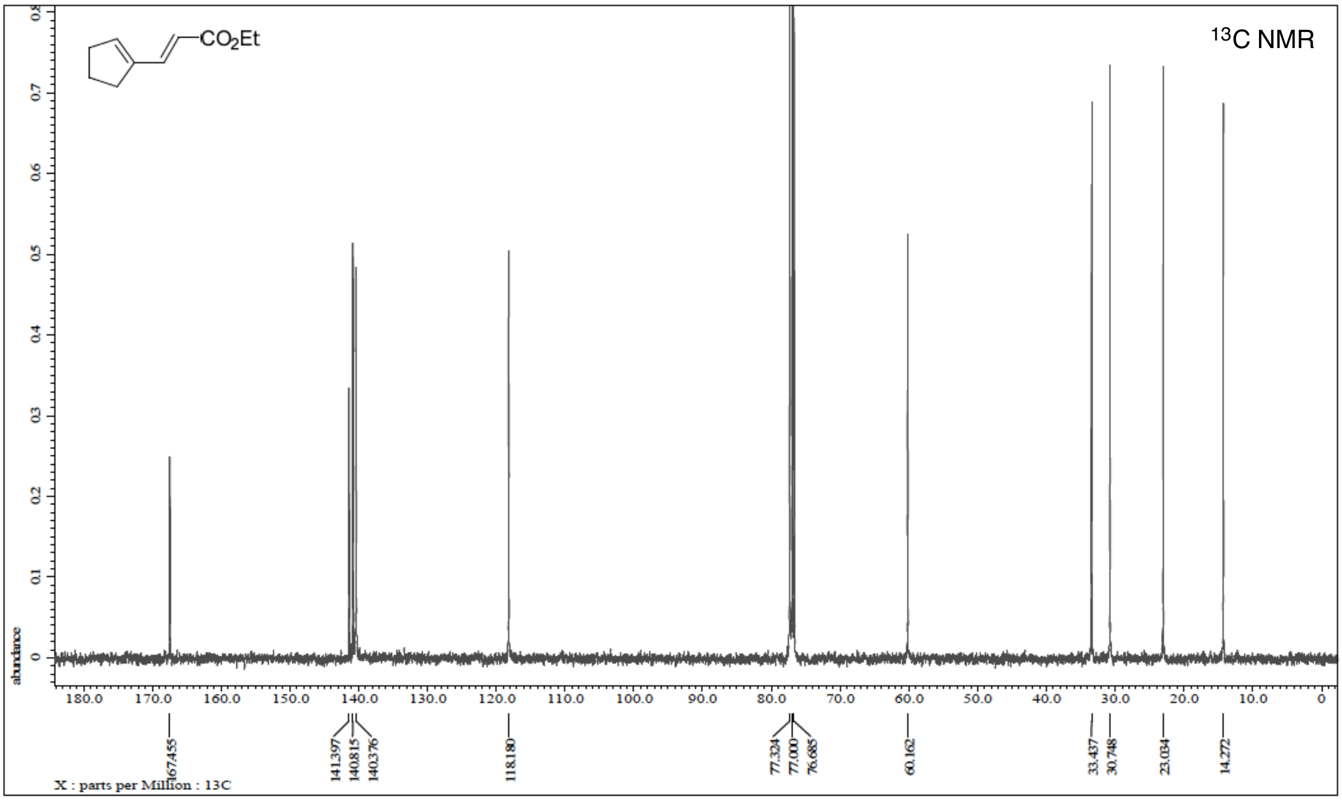

NMR spectra of **S4**

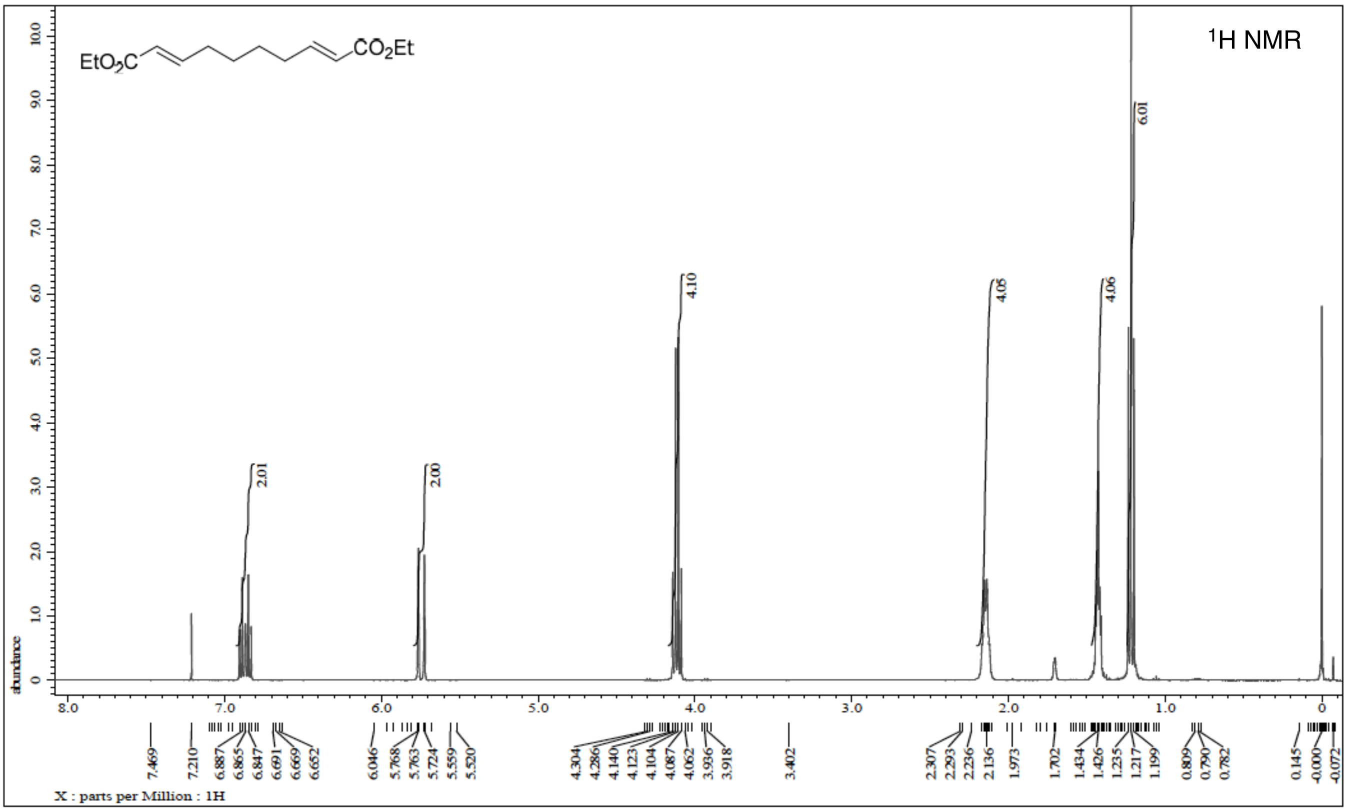

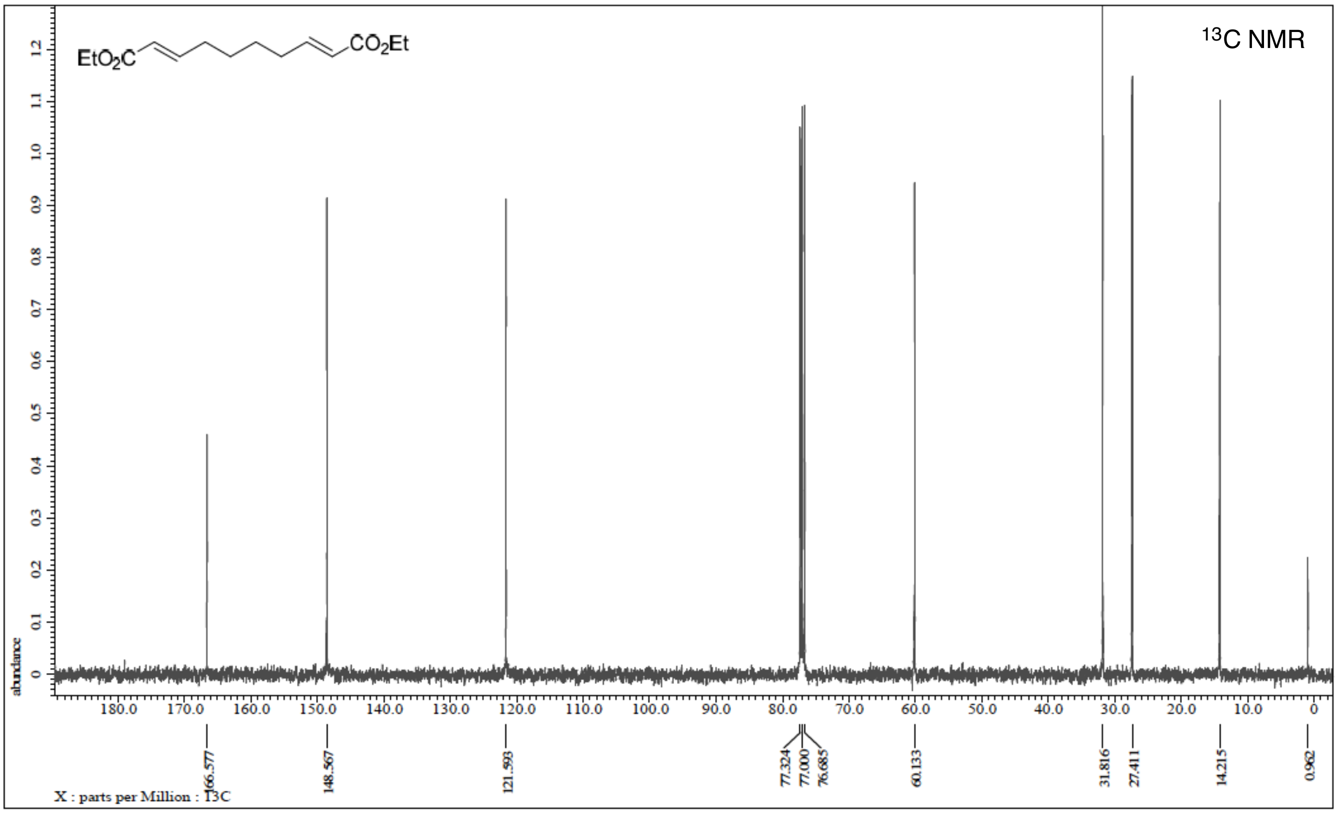

NMR spectra of *anti*-**5a**

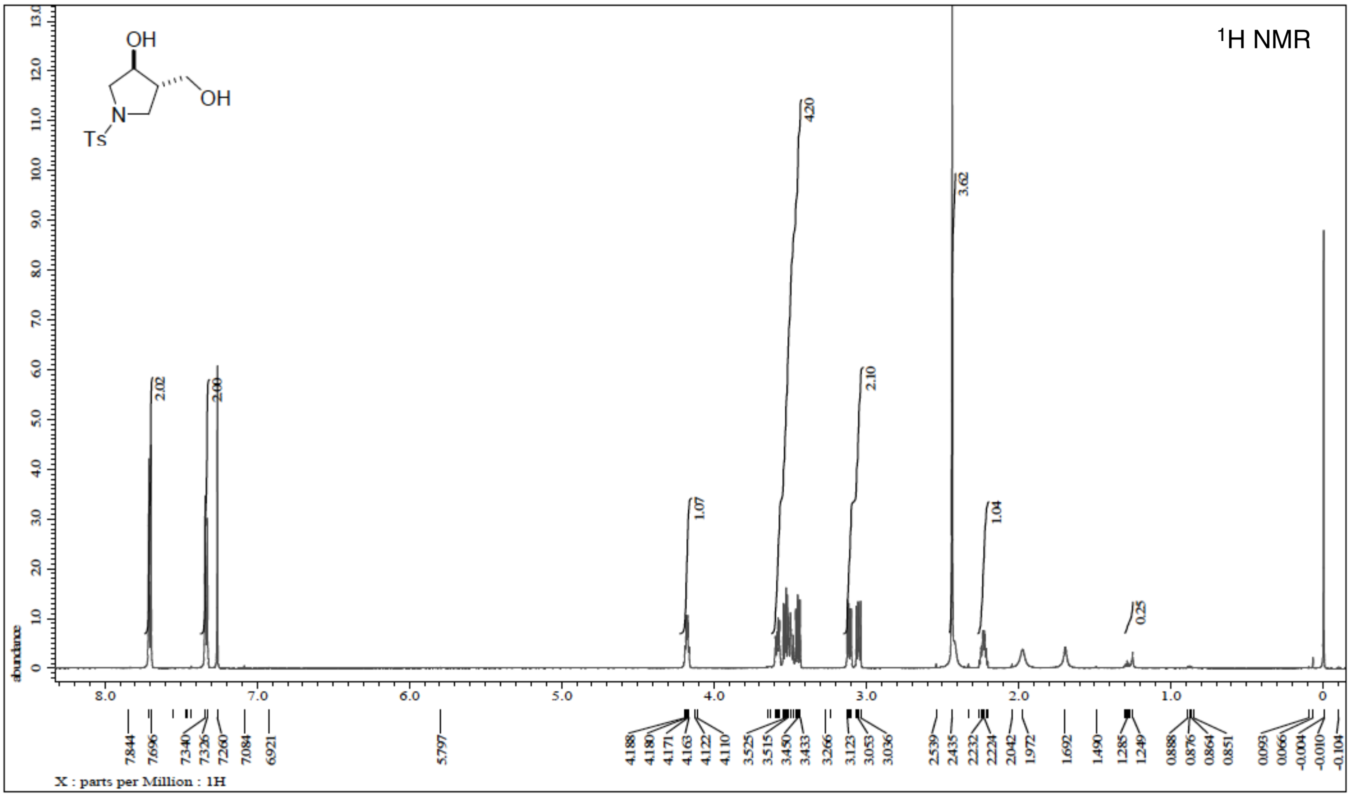

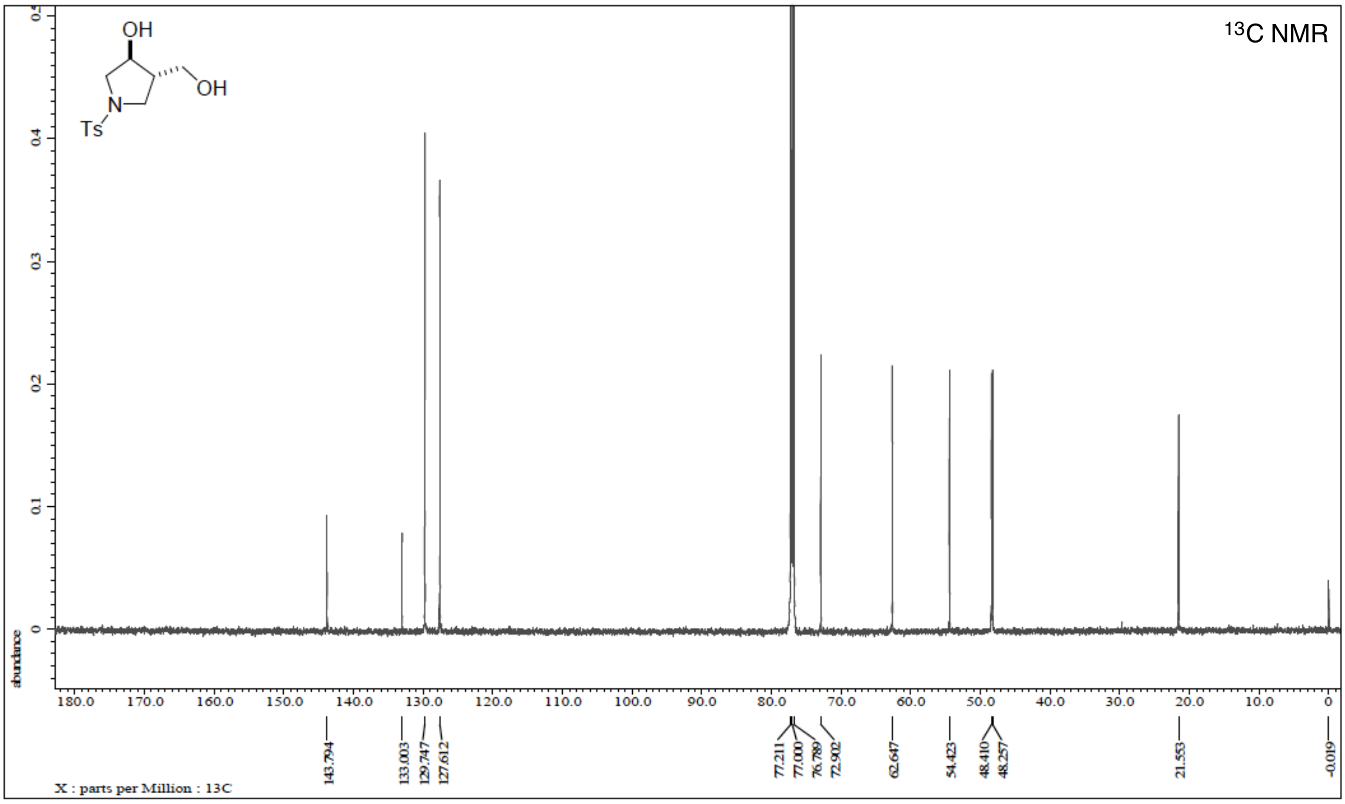

NMR spectra of *syn*-**6**

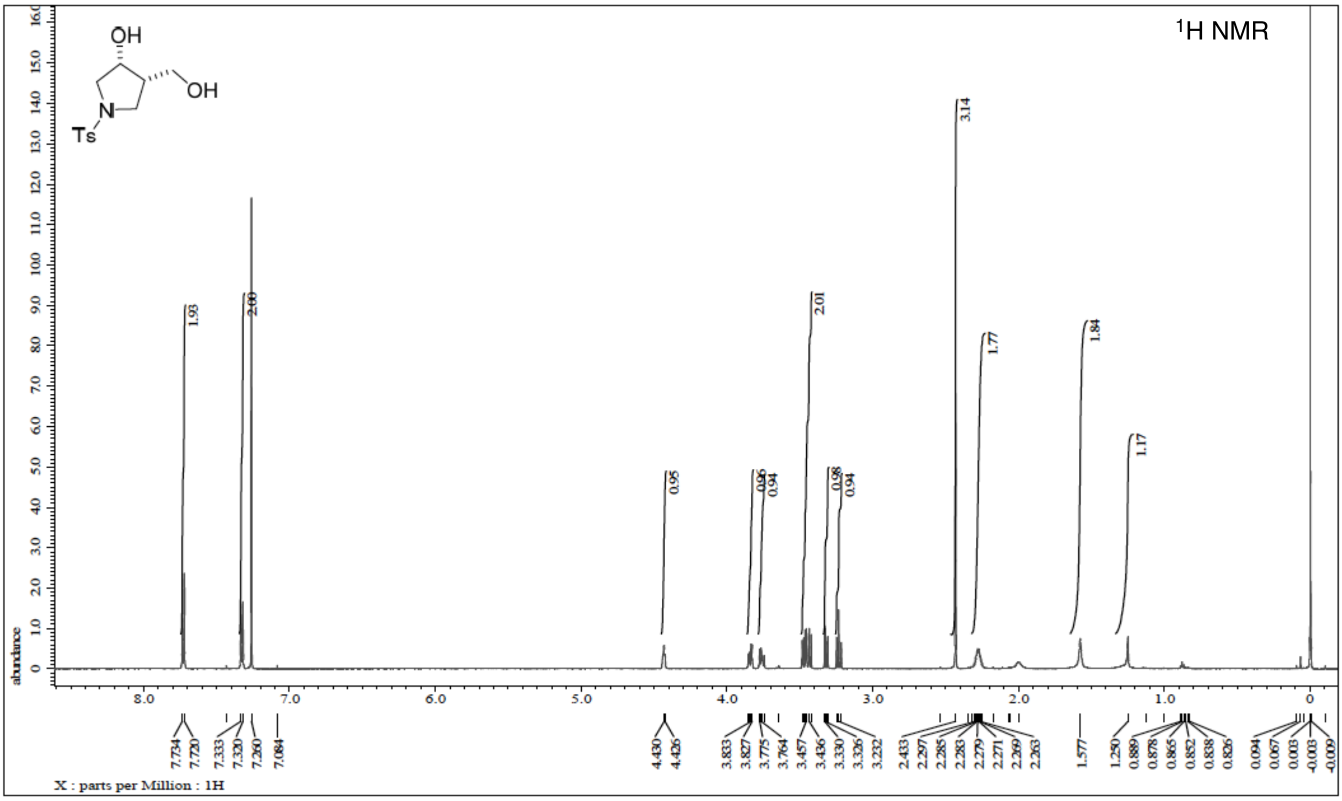

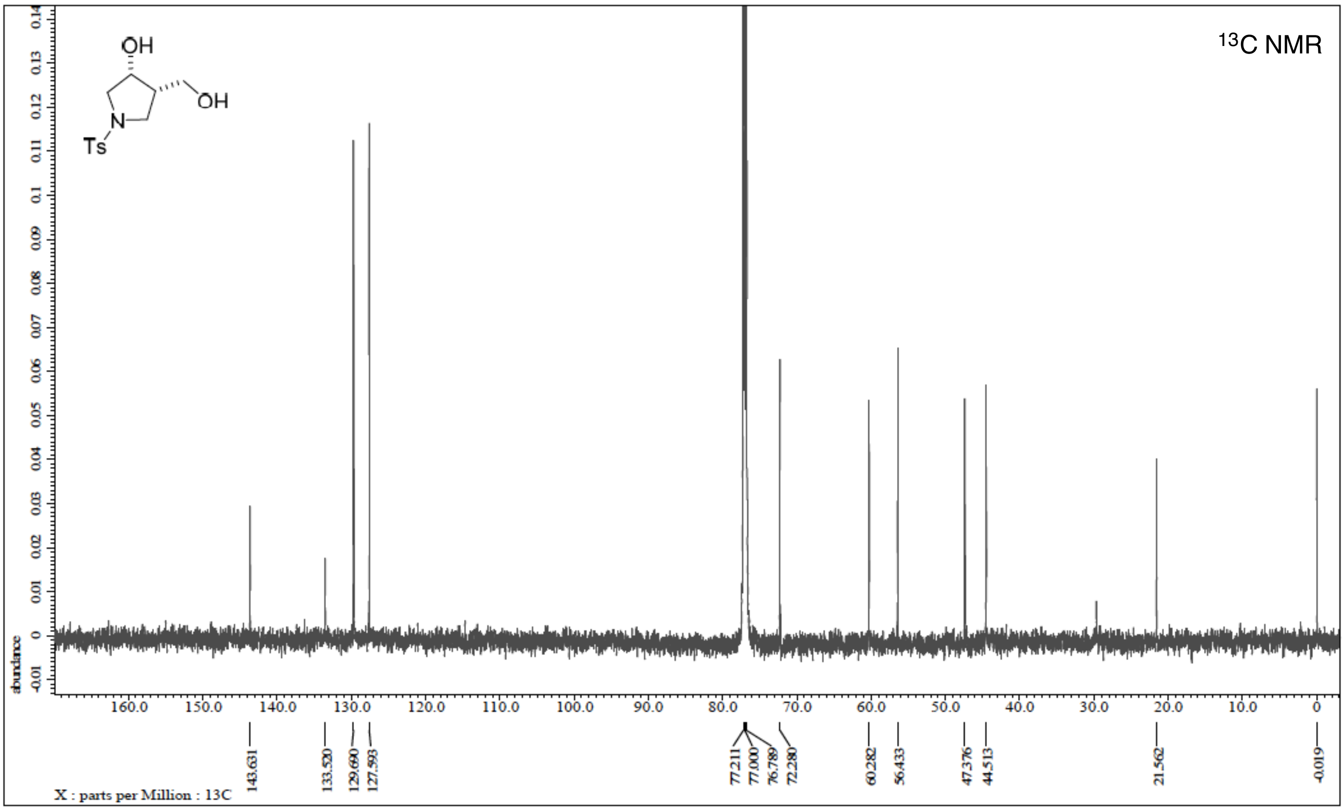

NMR spectra of *anti*-**7**

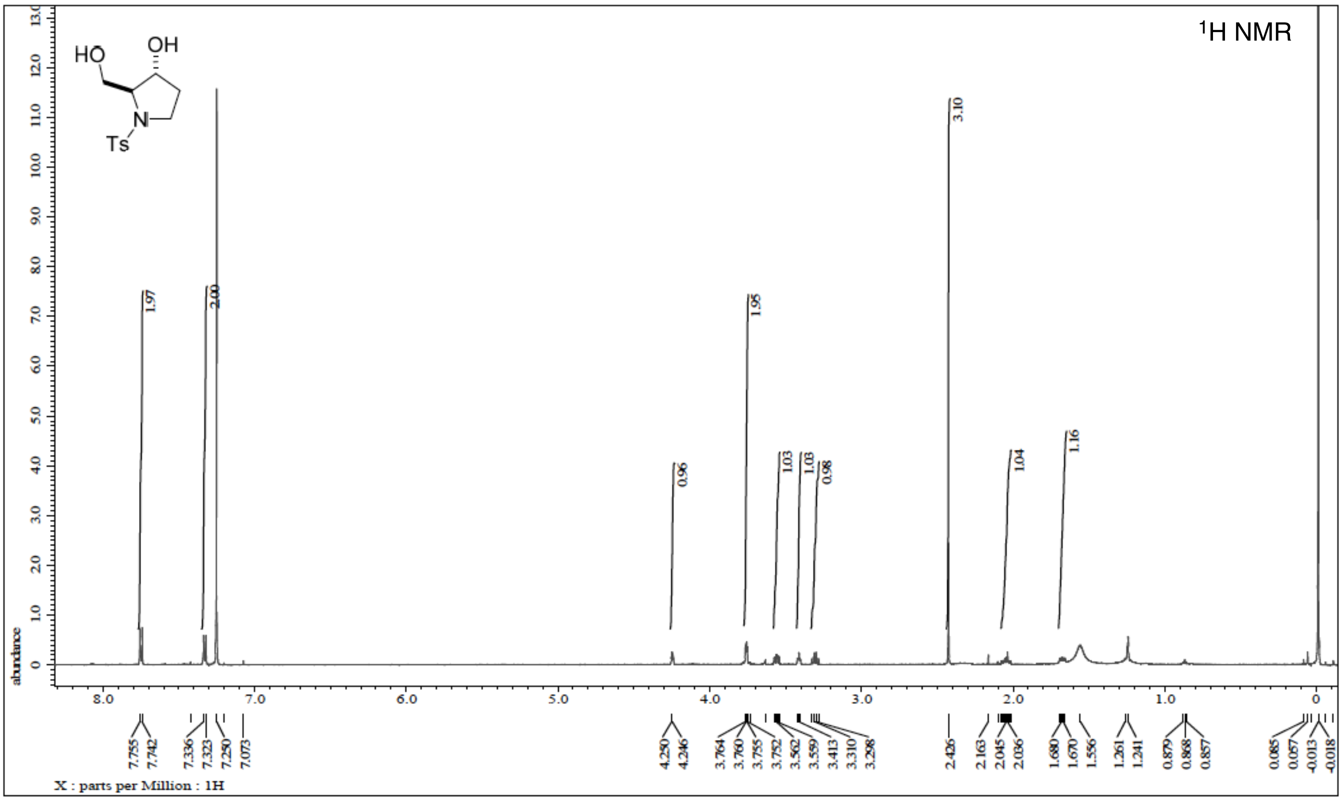

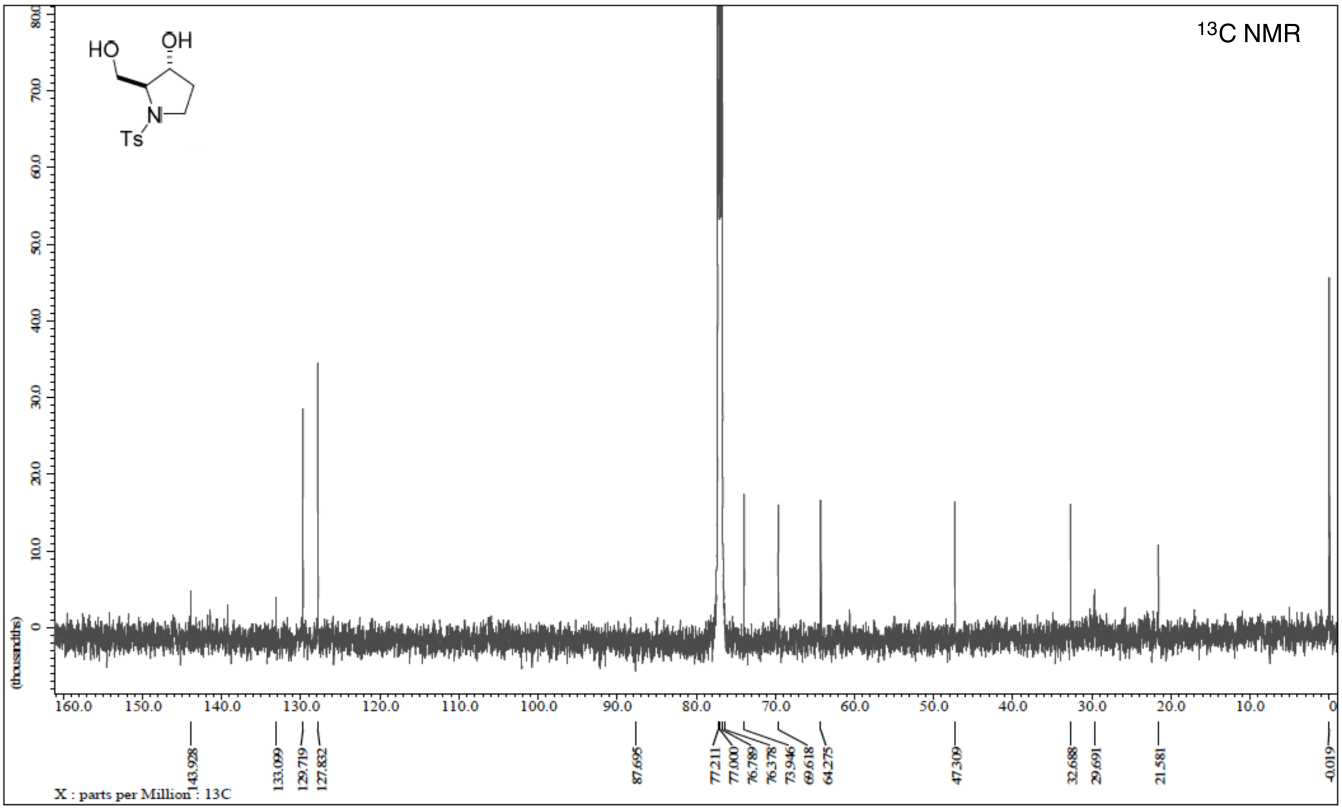

NMR spectra of *syn*-**8**

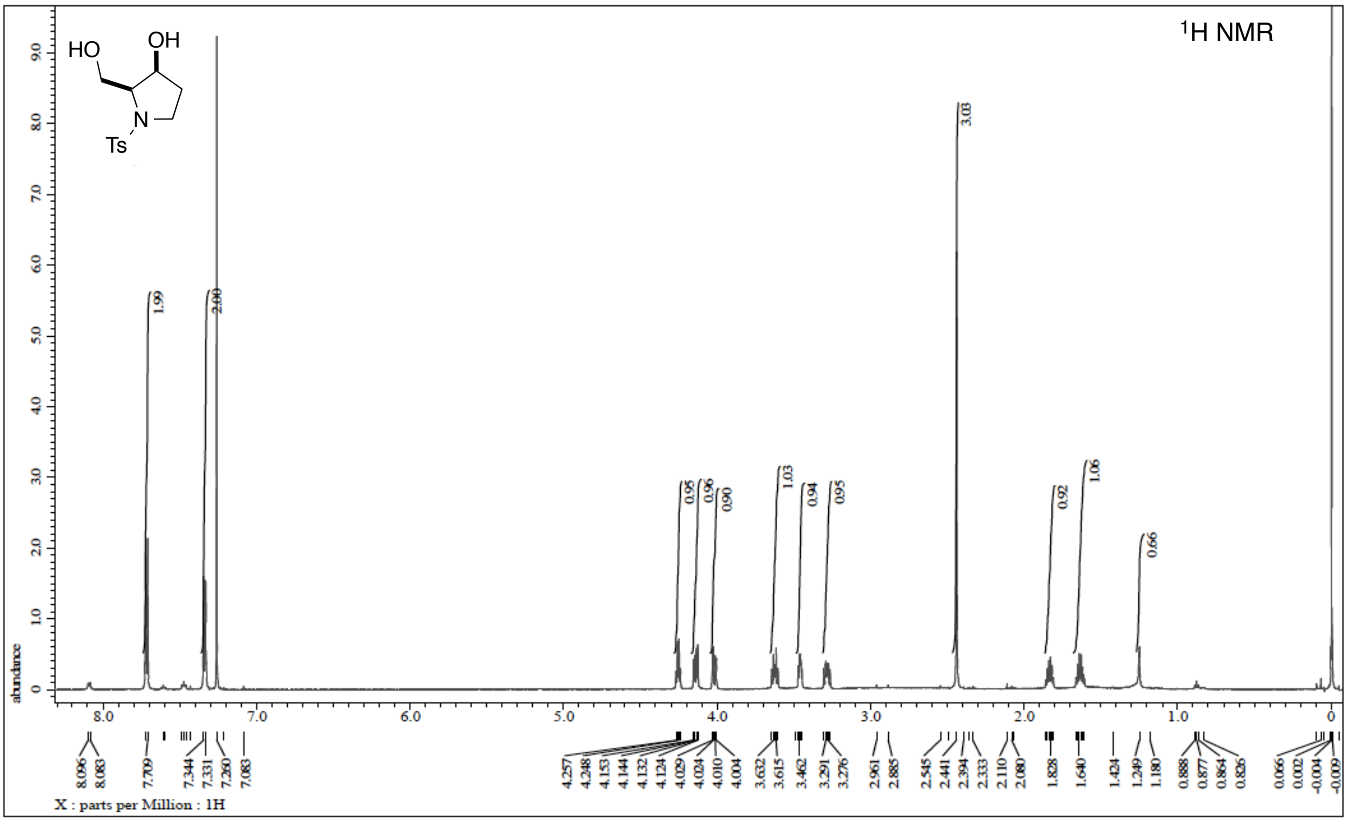

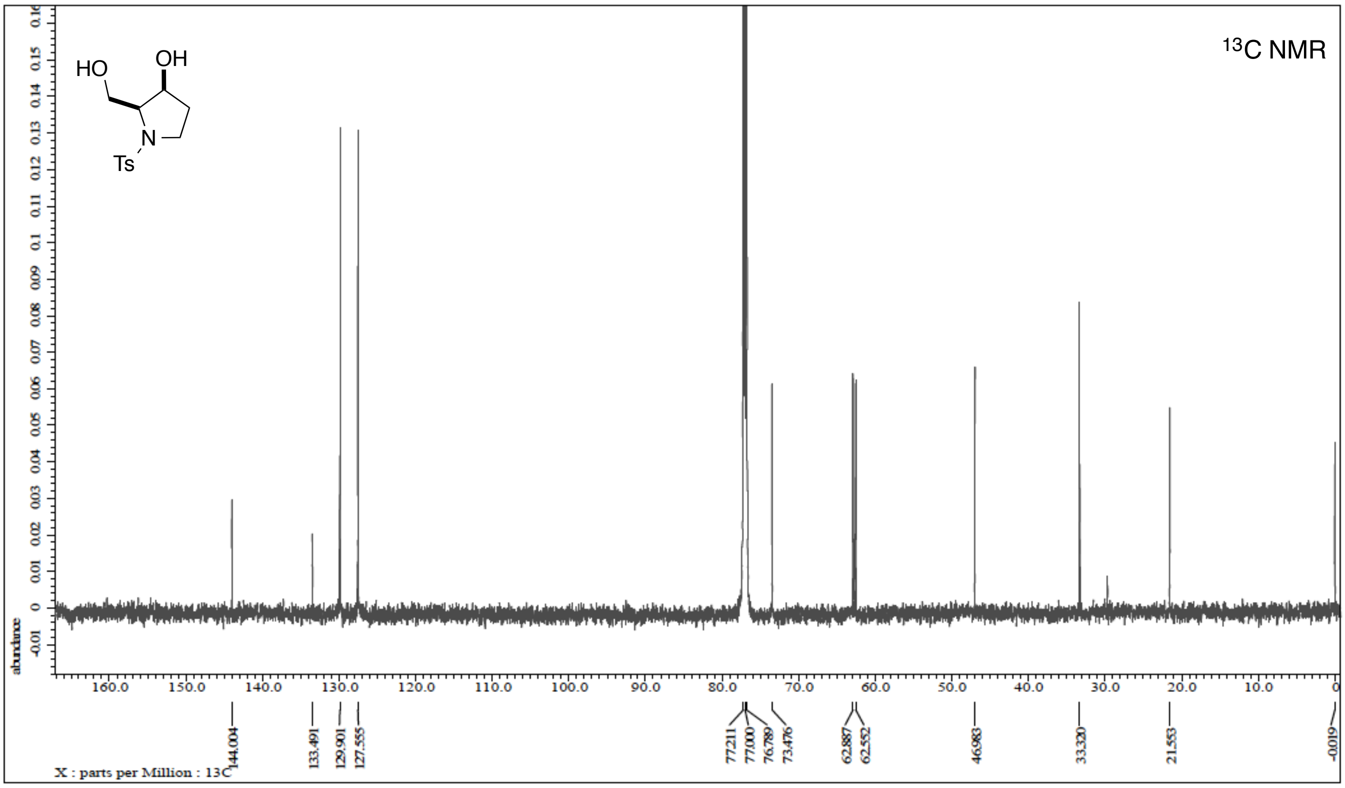

NMR spectra of **9**

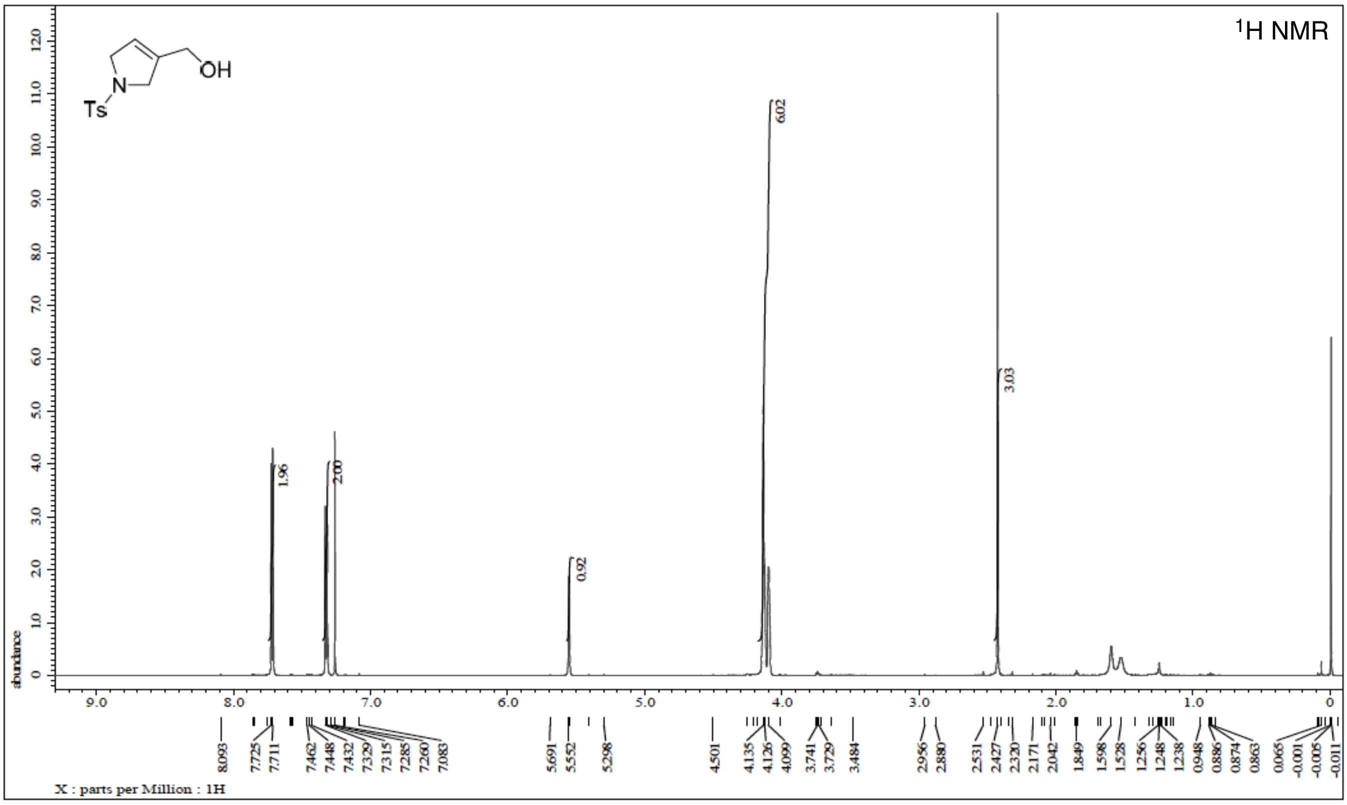

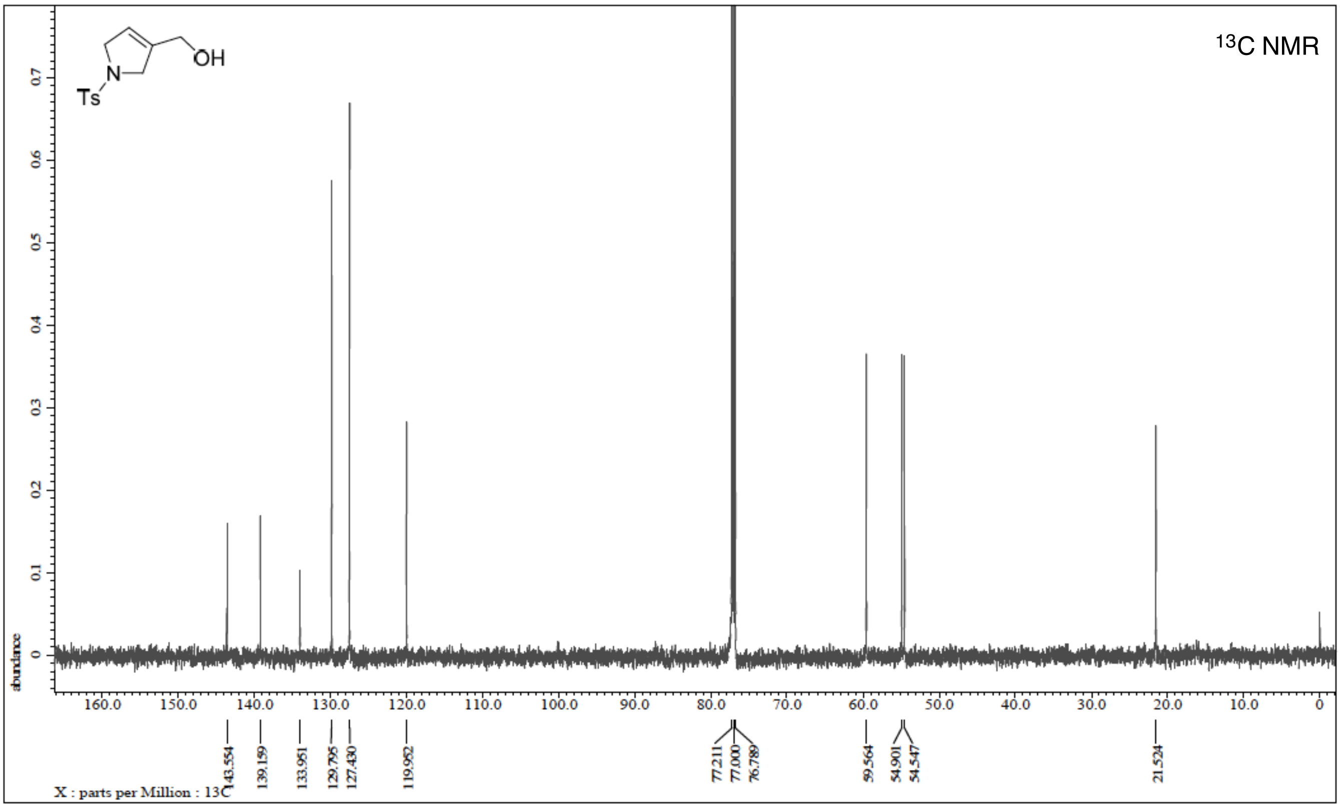

NMR spectra of **10**

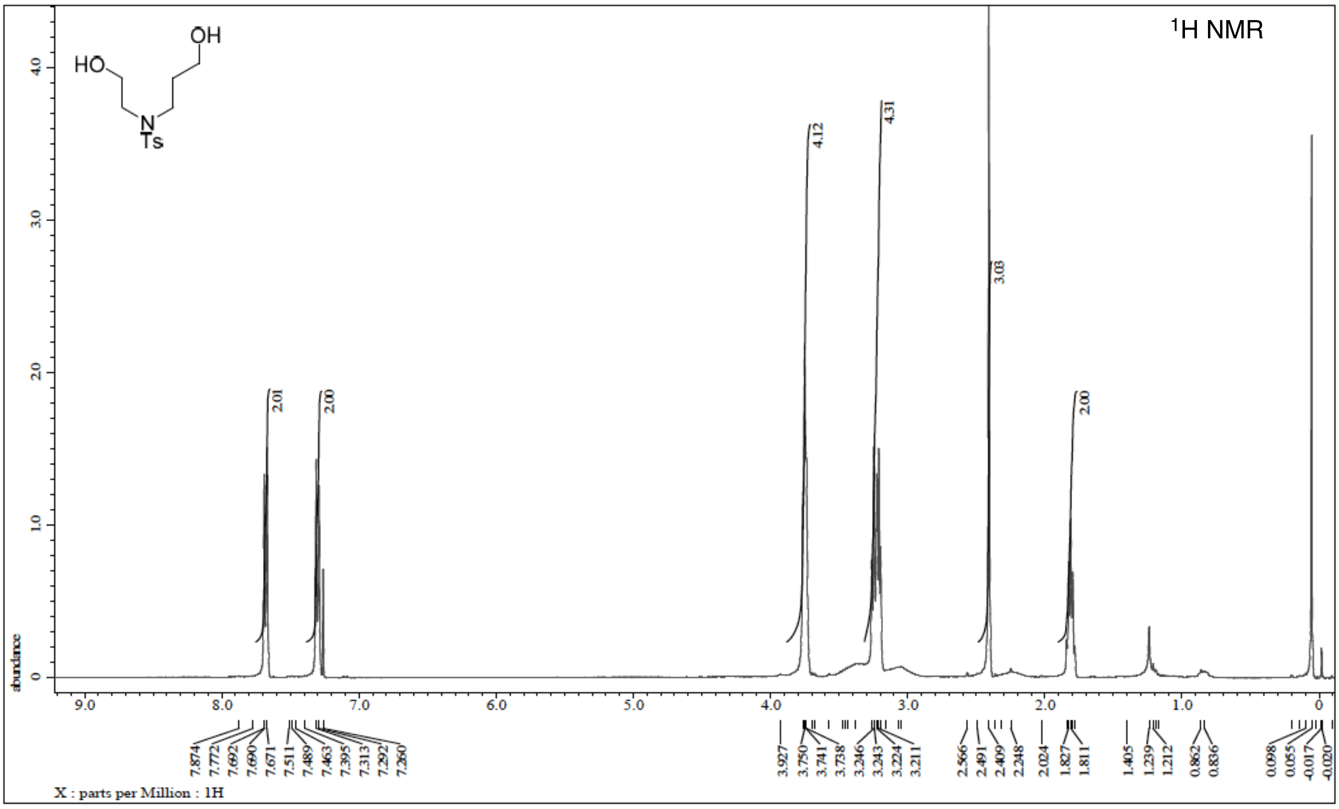

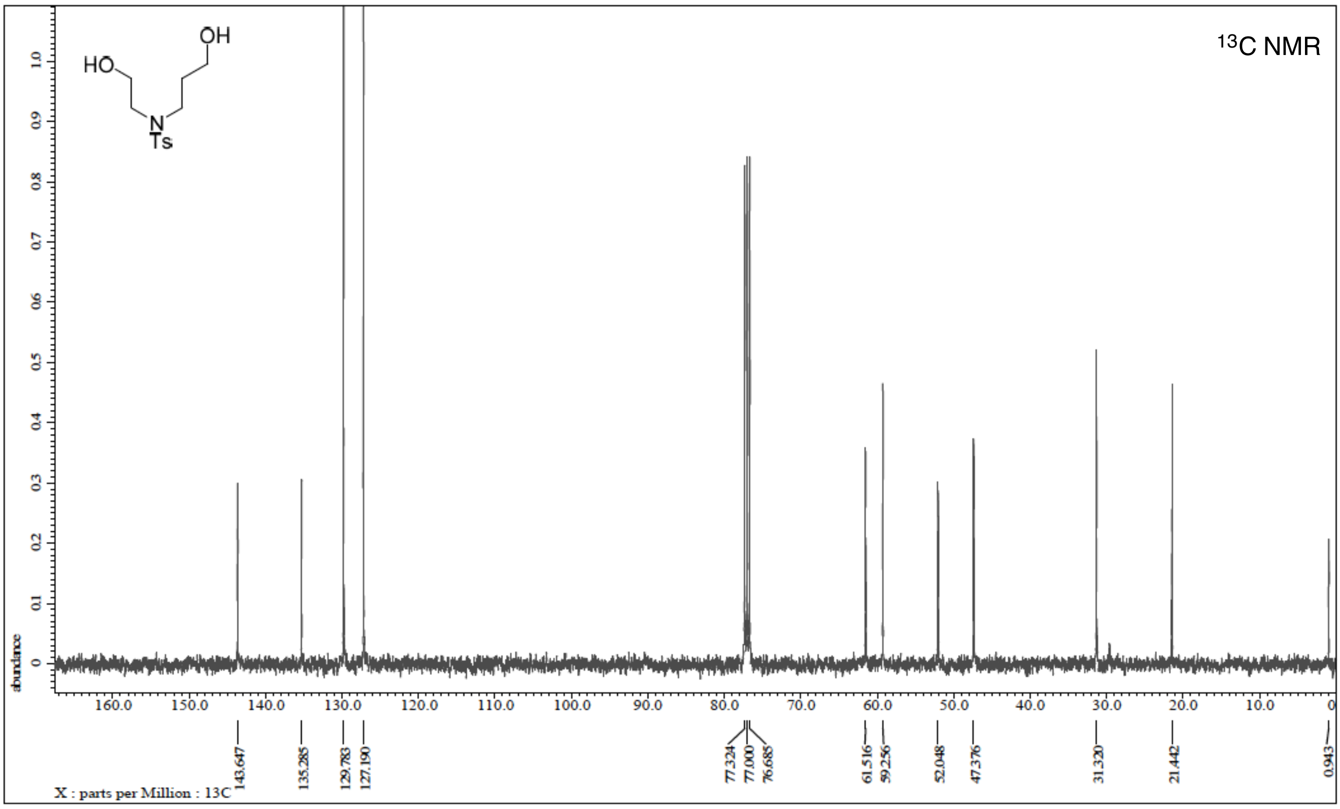

NMR spectra of *anti*-**5b**

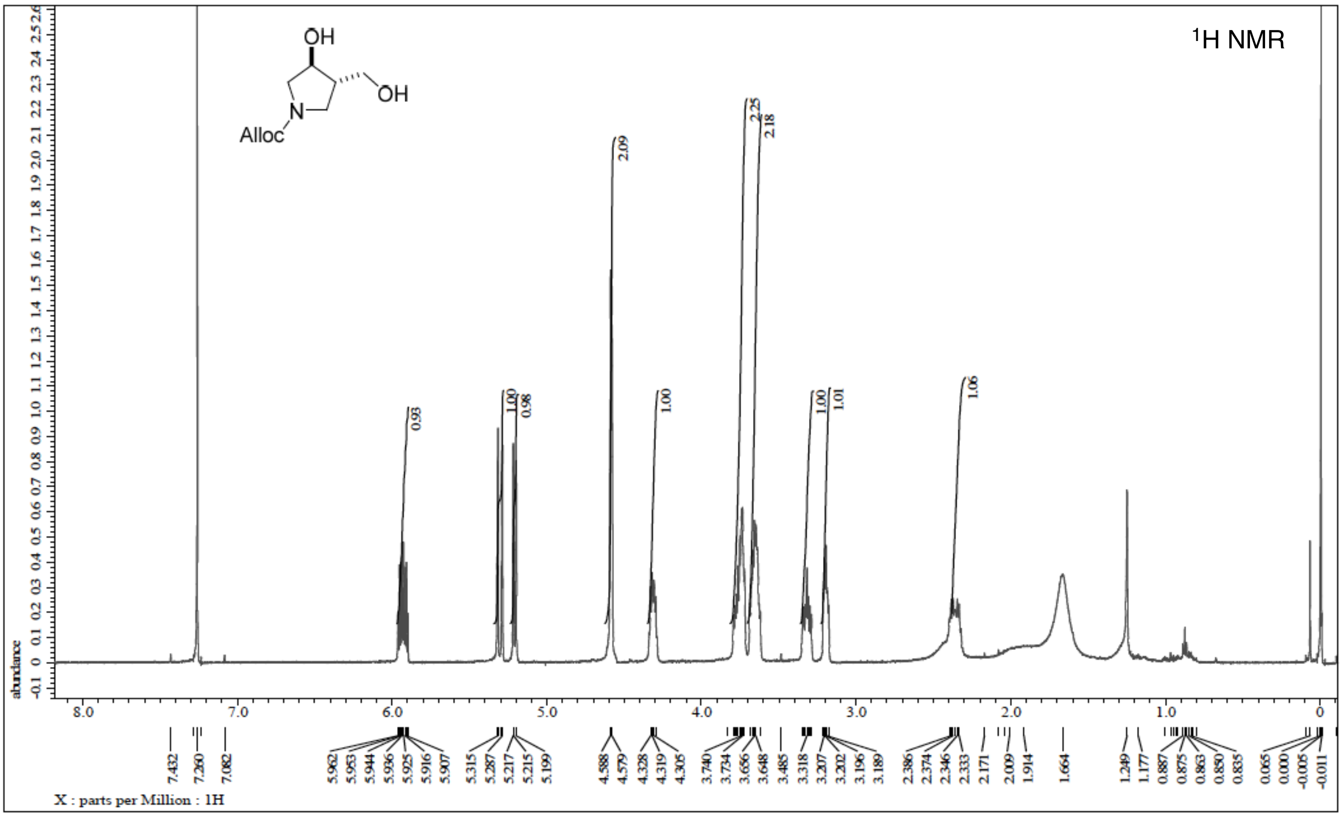

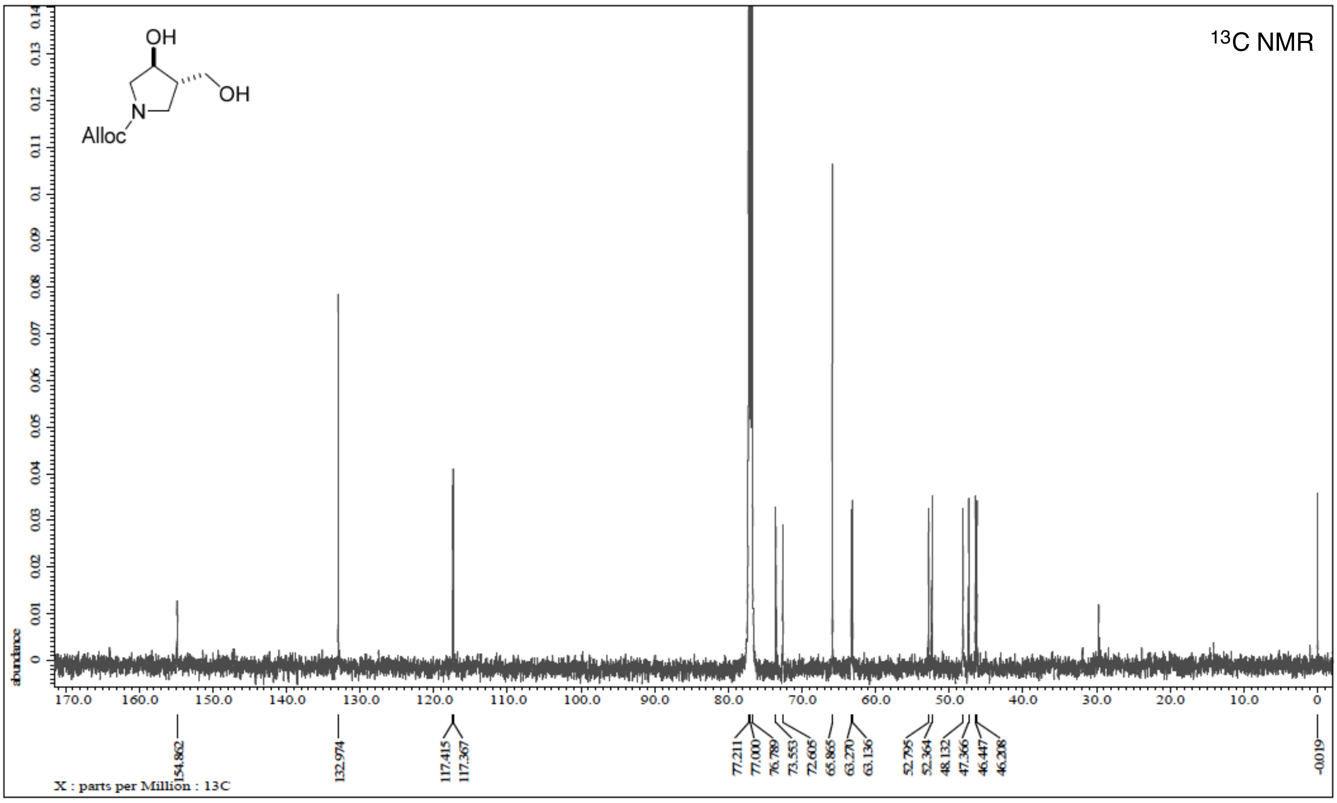

NMR spectra of **1a**

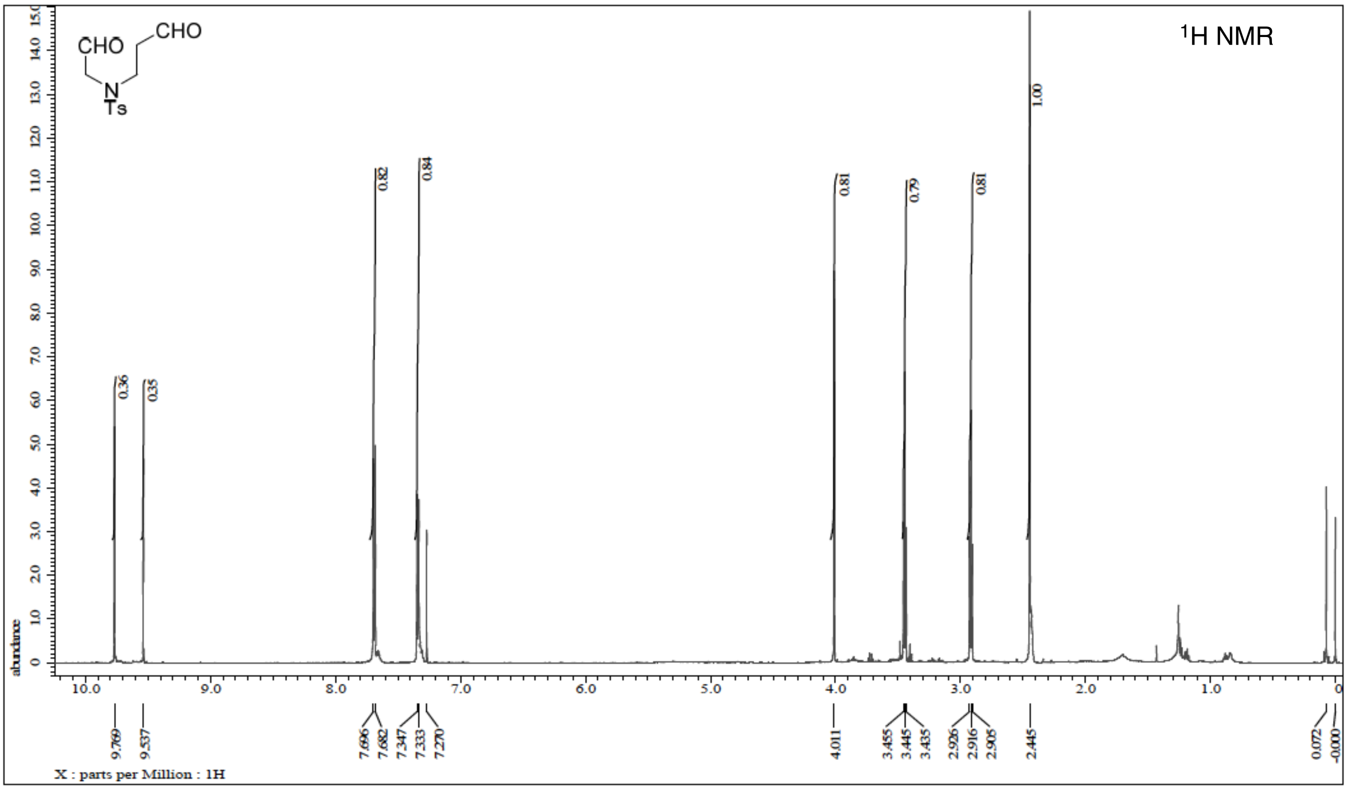

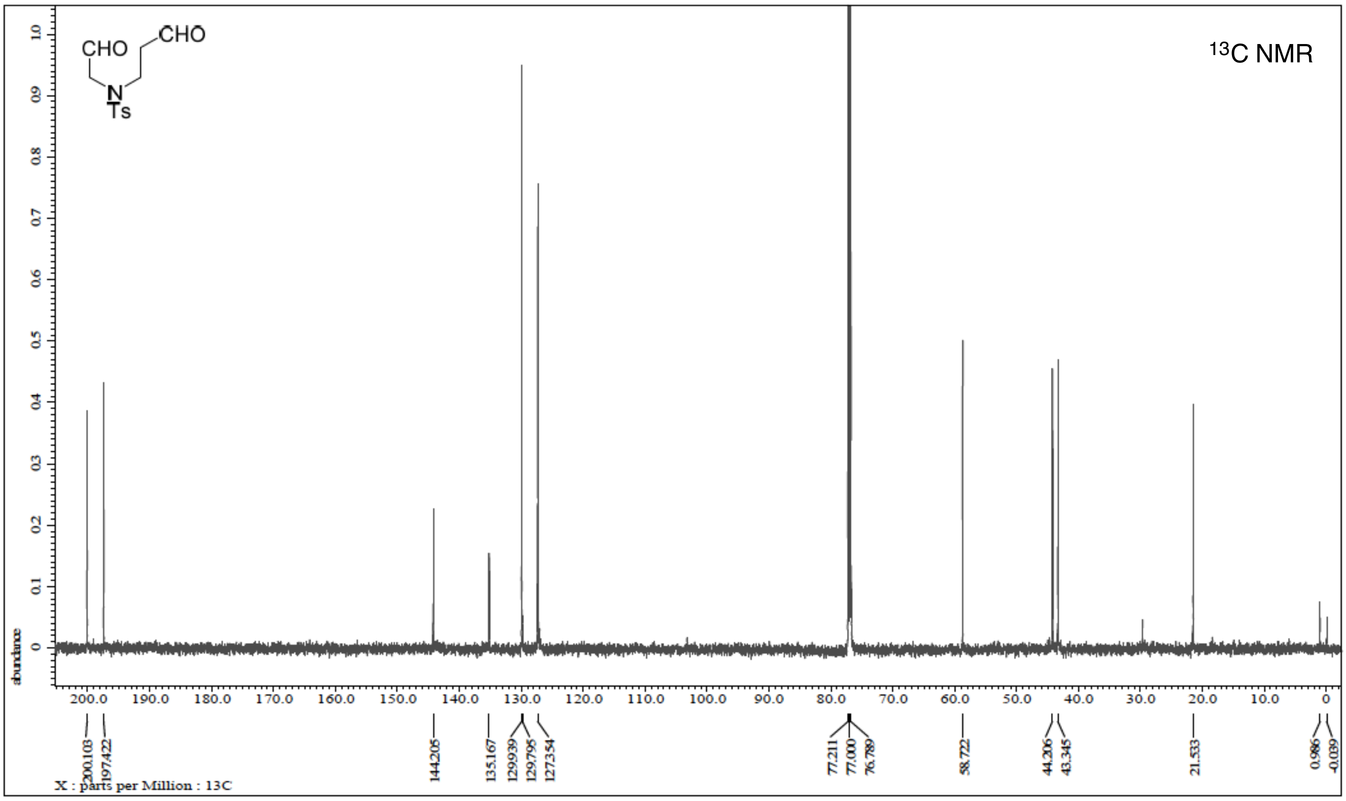

NMR spectra of **S12**

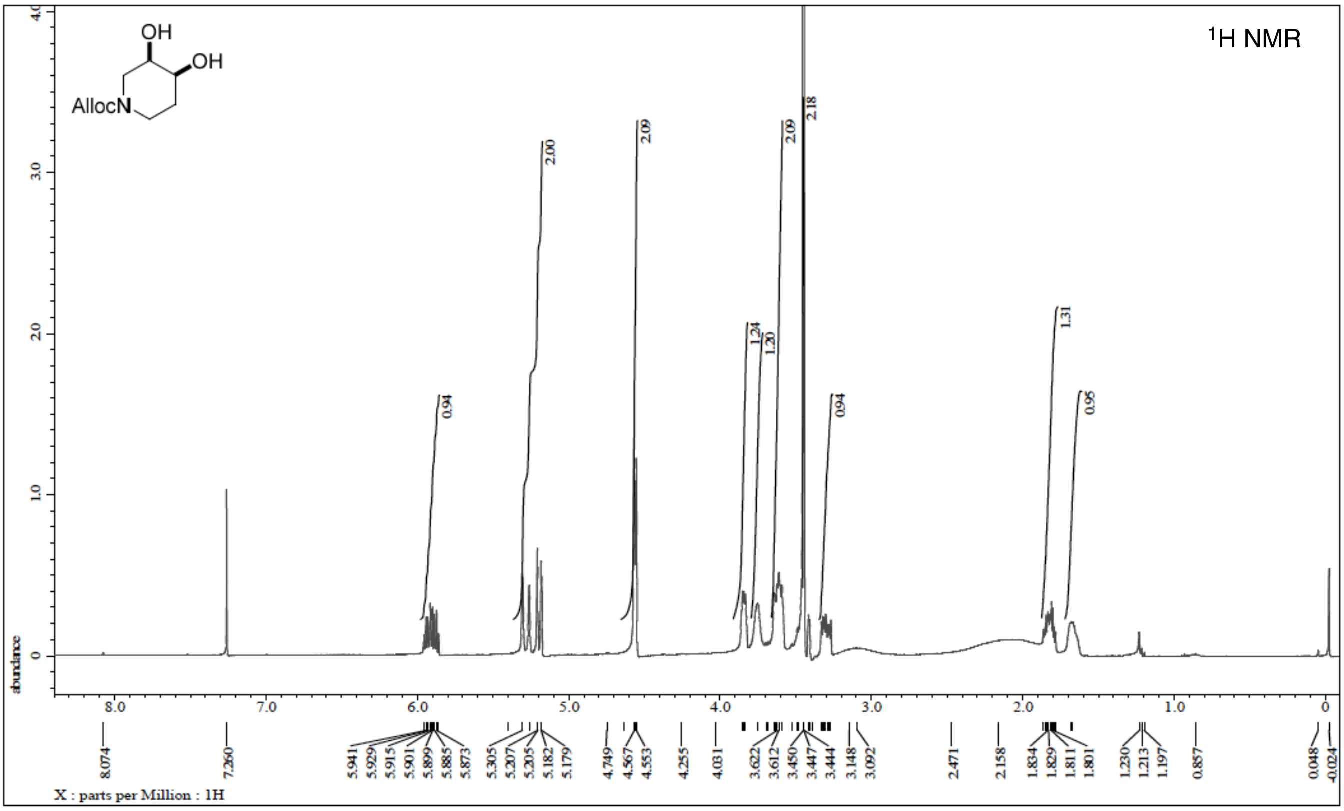

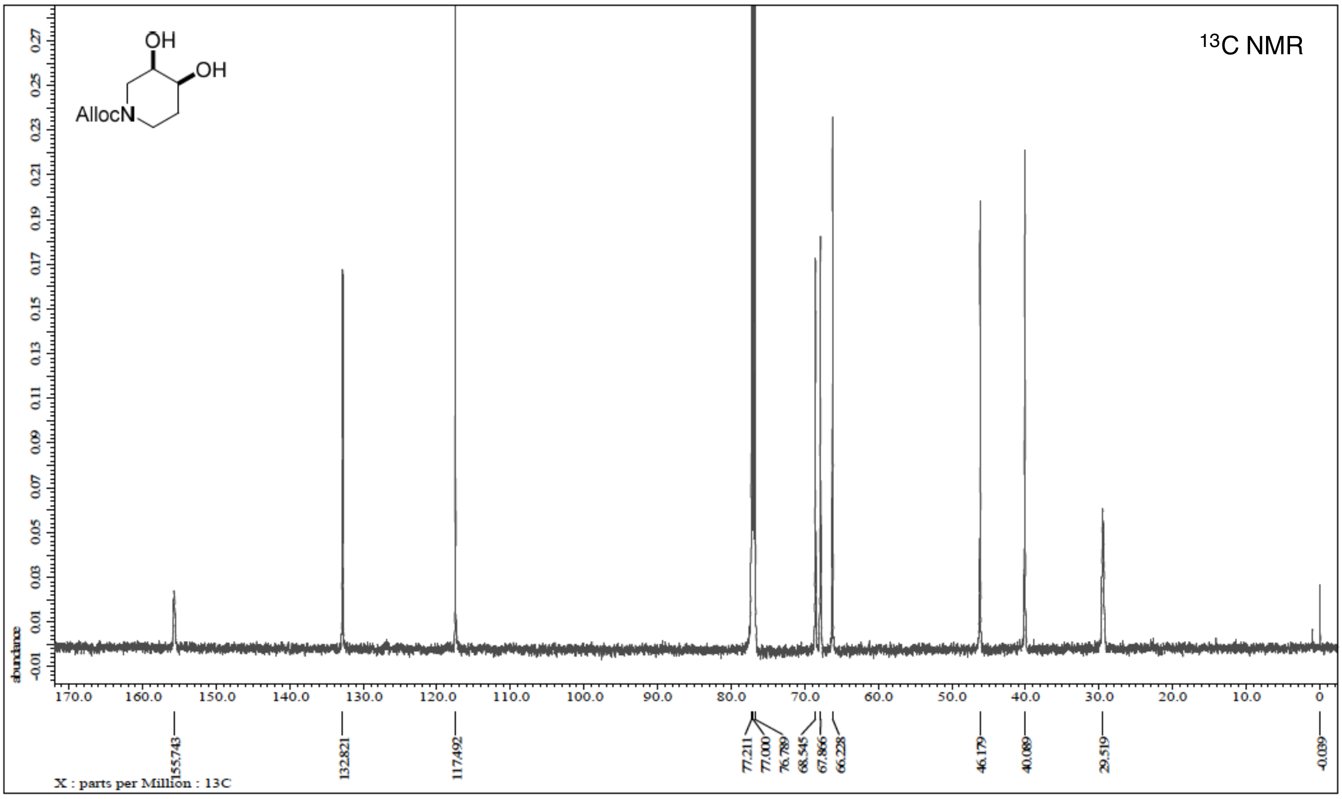

NMR spectra of **1b**

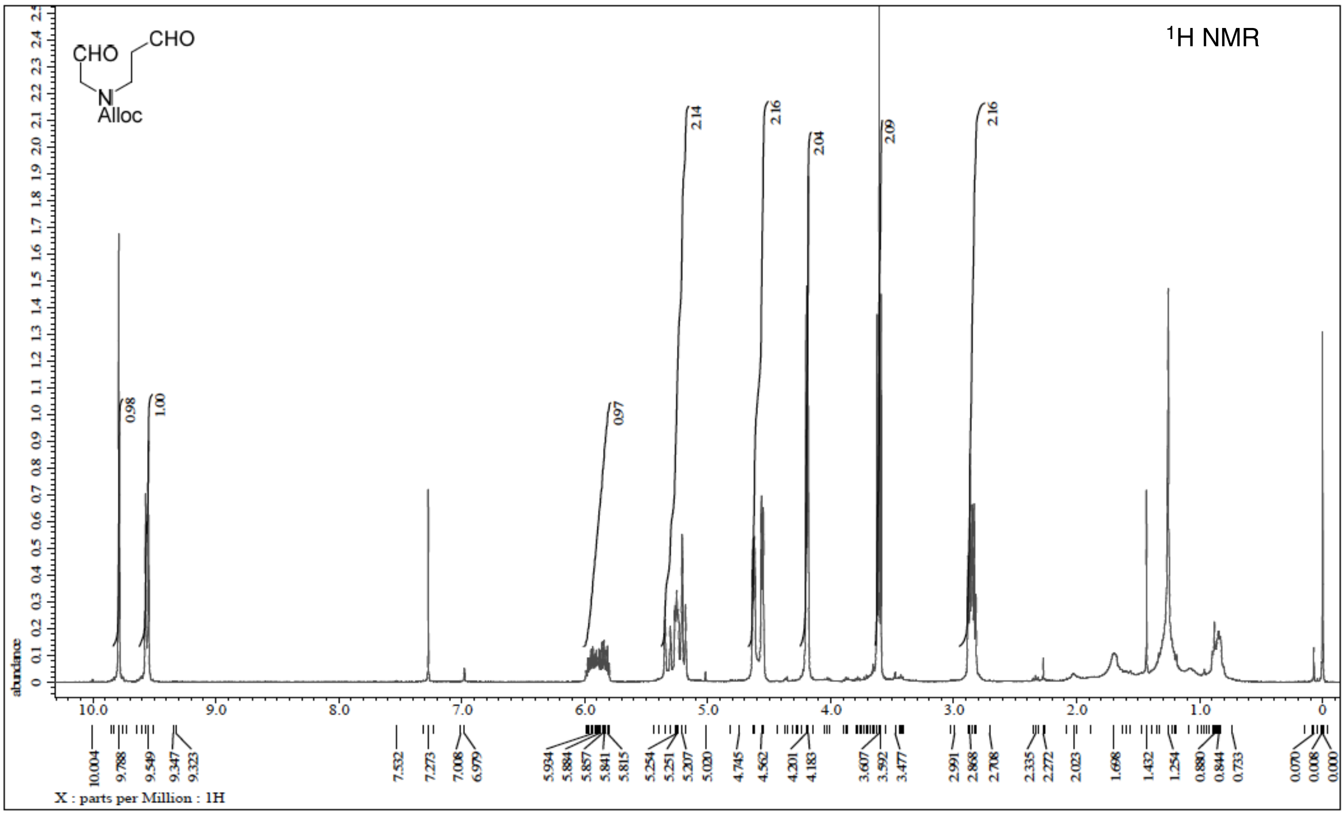

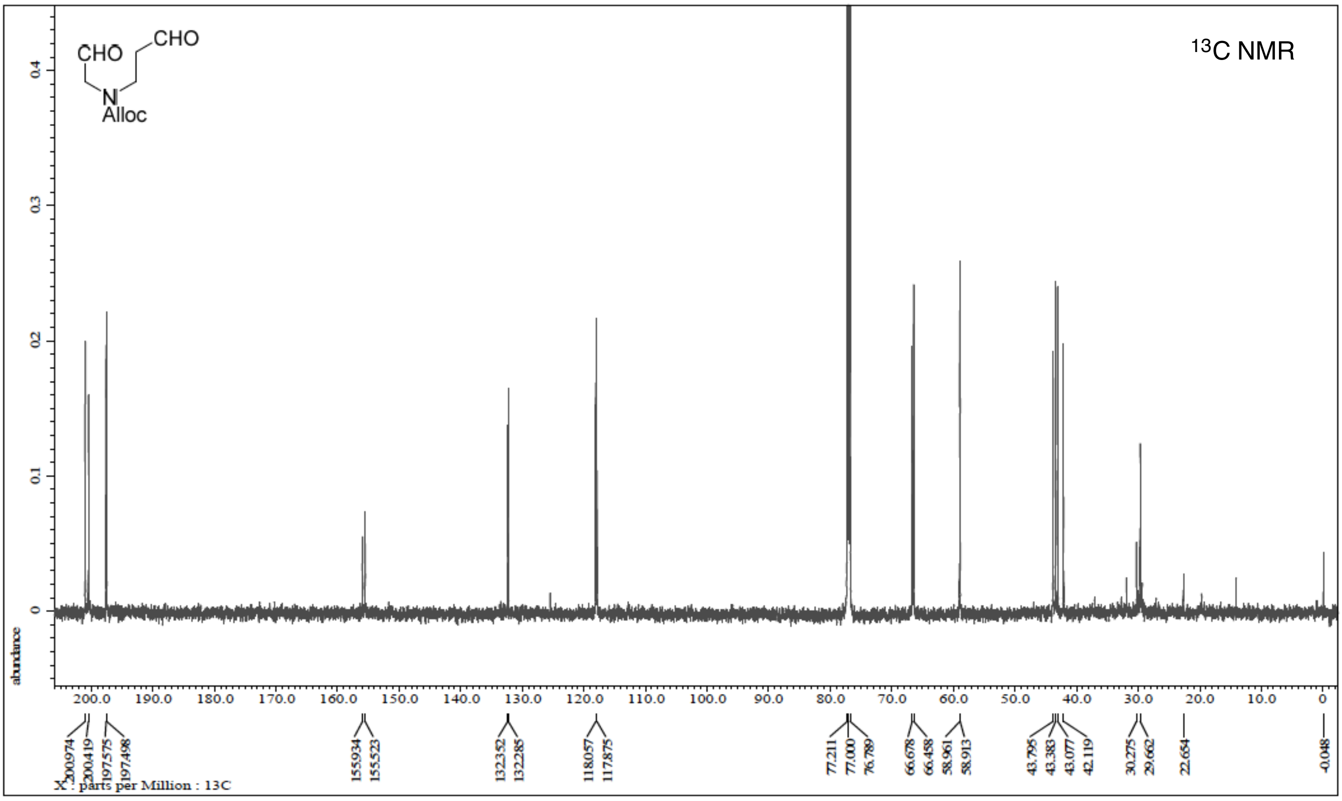

NMR spectra of **1c**

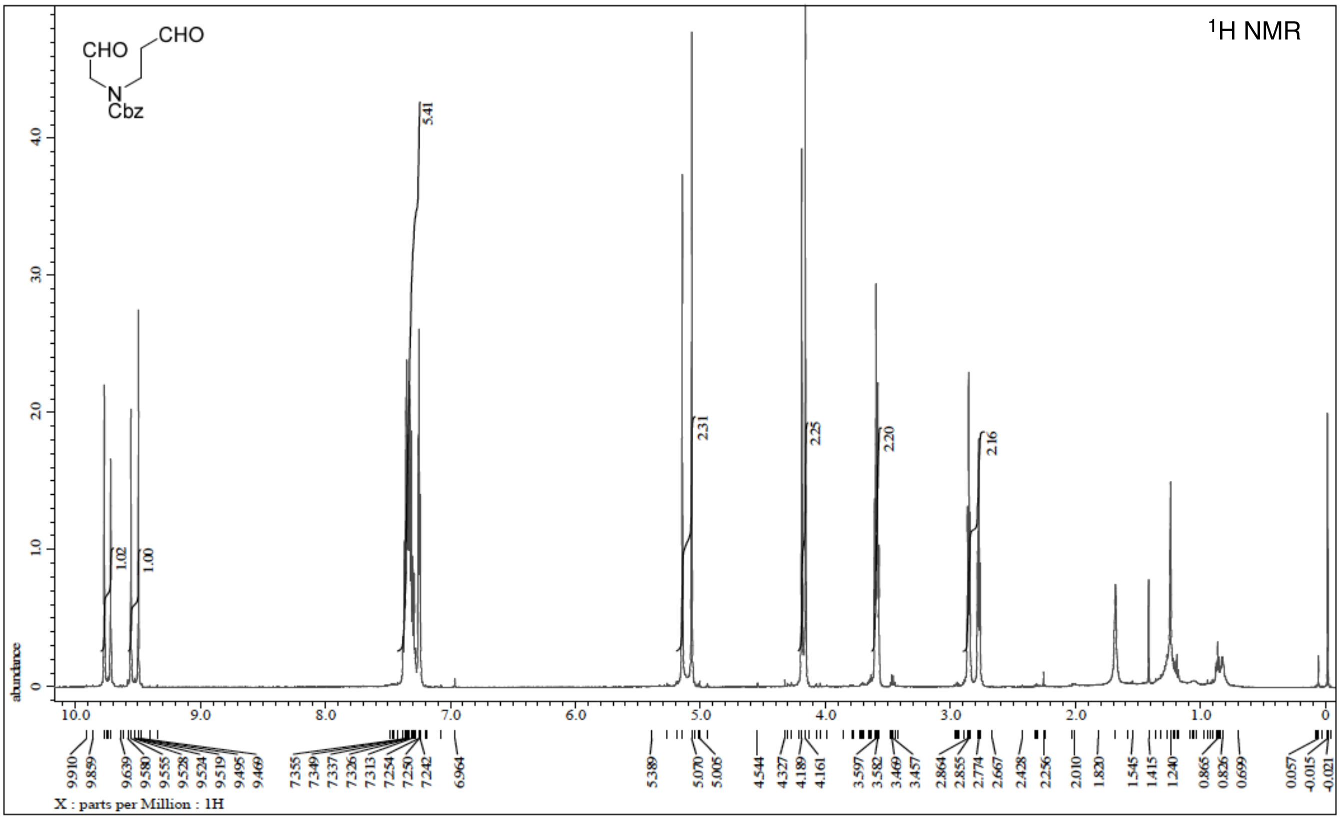

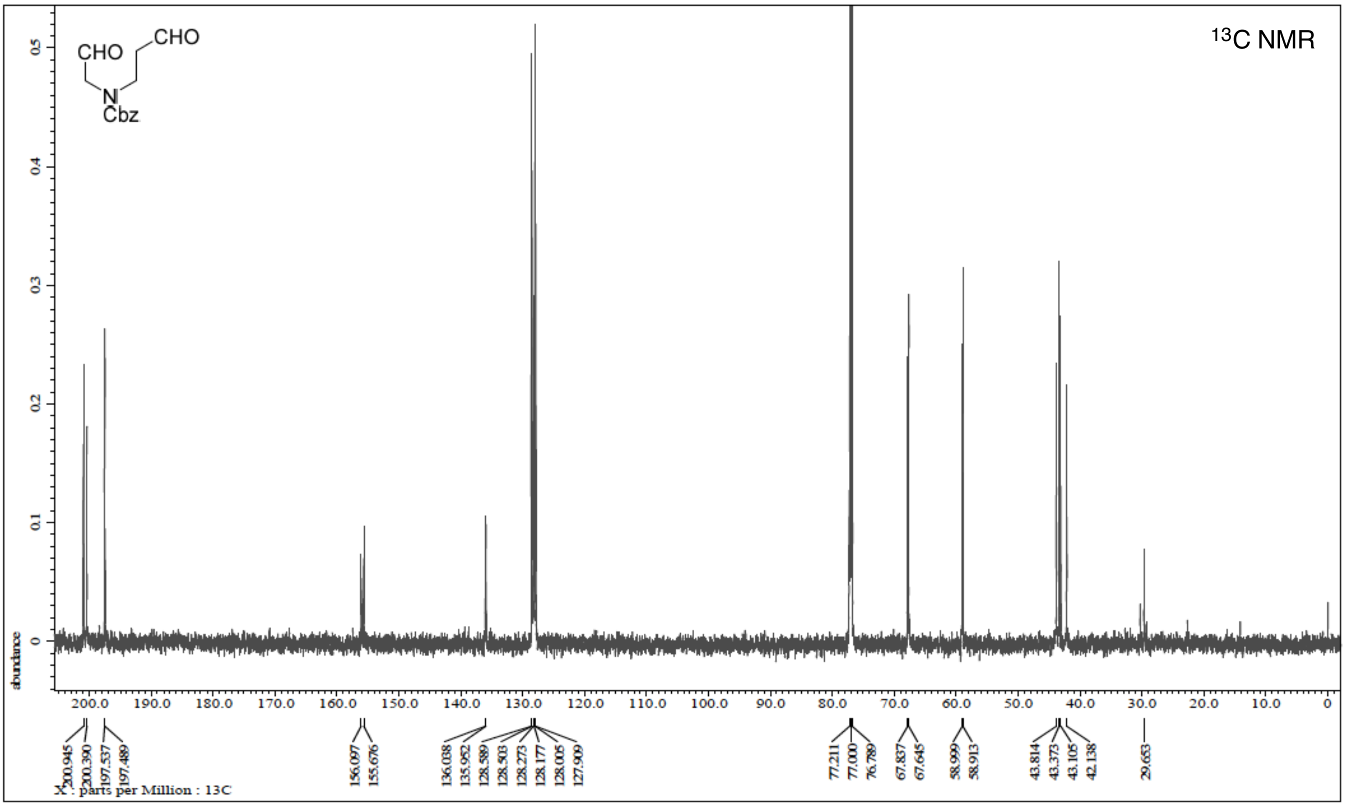

NMR spectra of **1d**

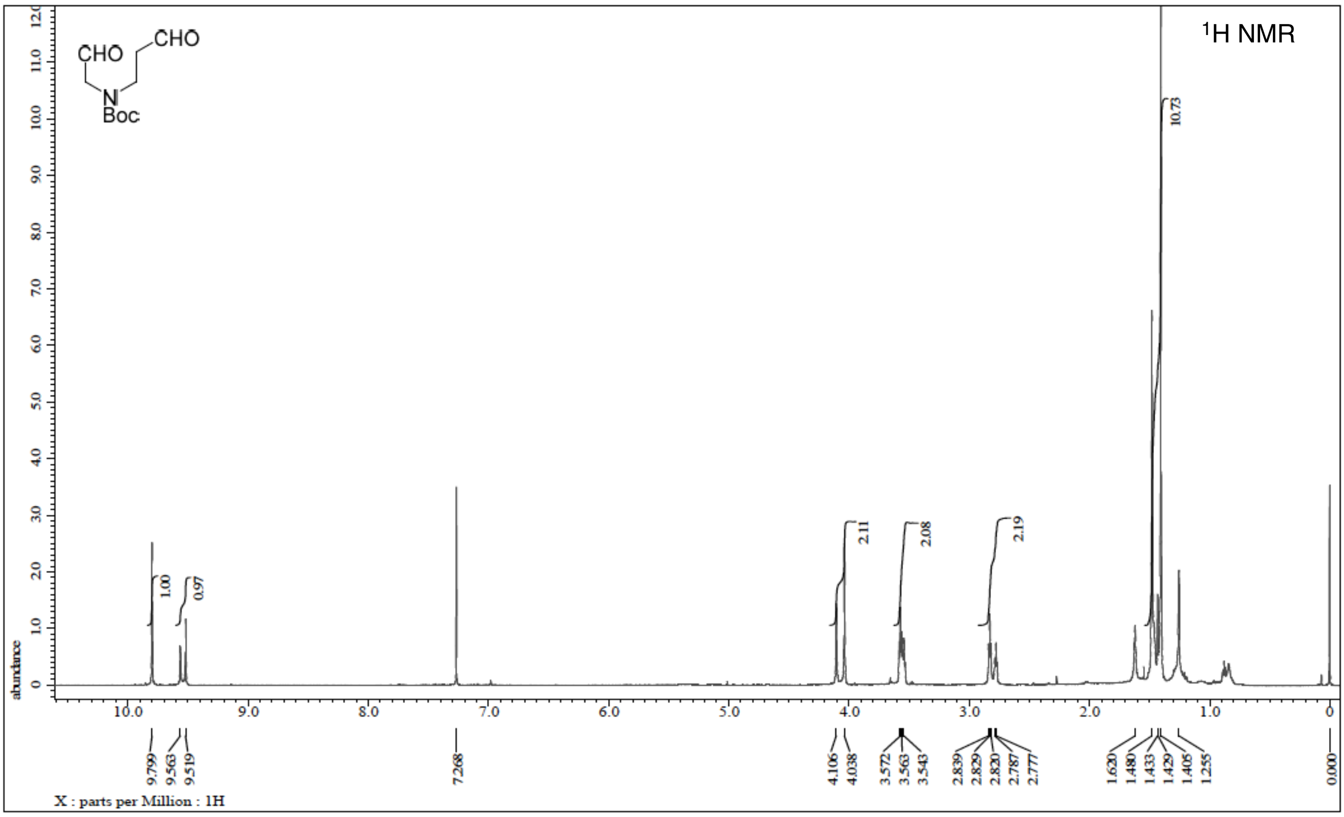

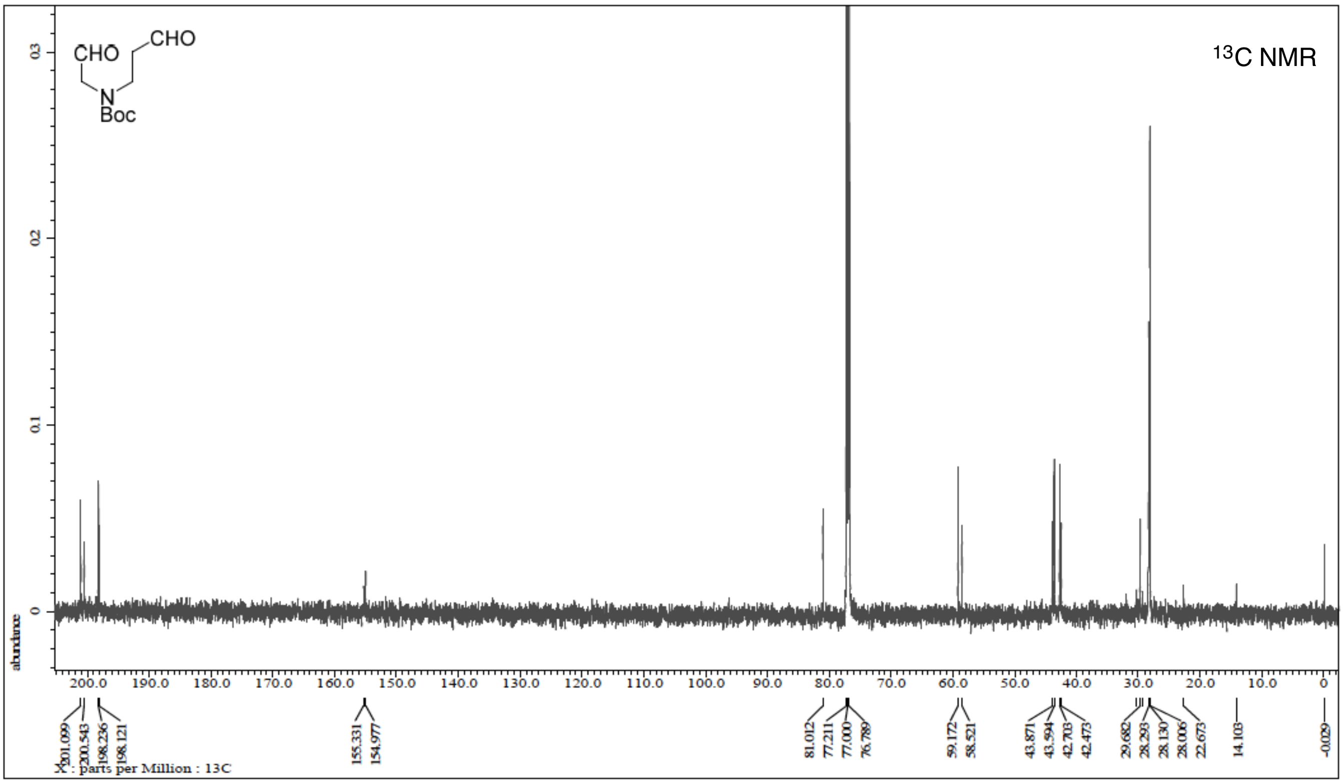

NMR spectra of **S15**

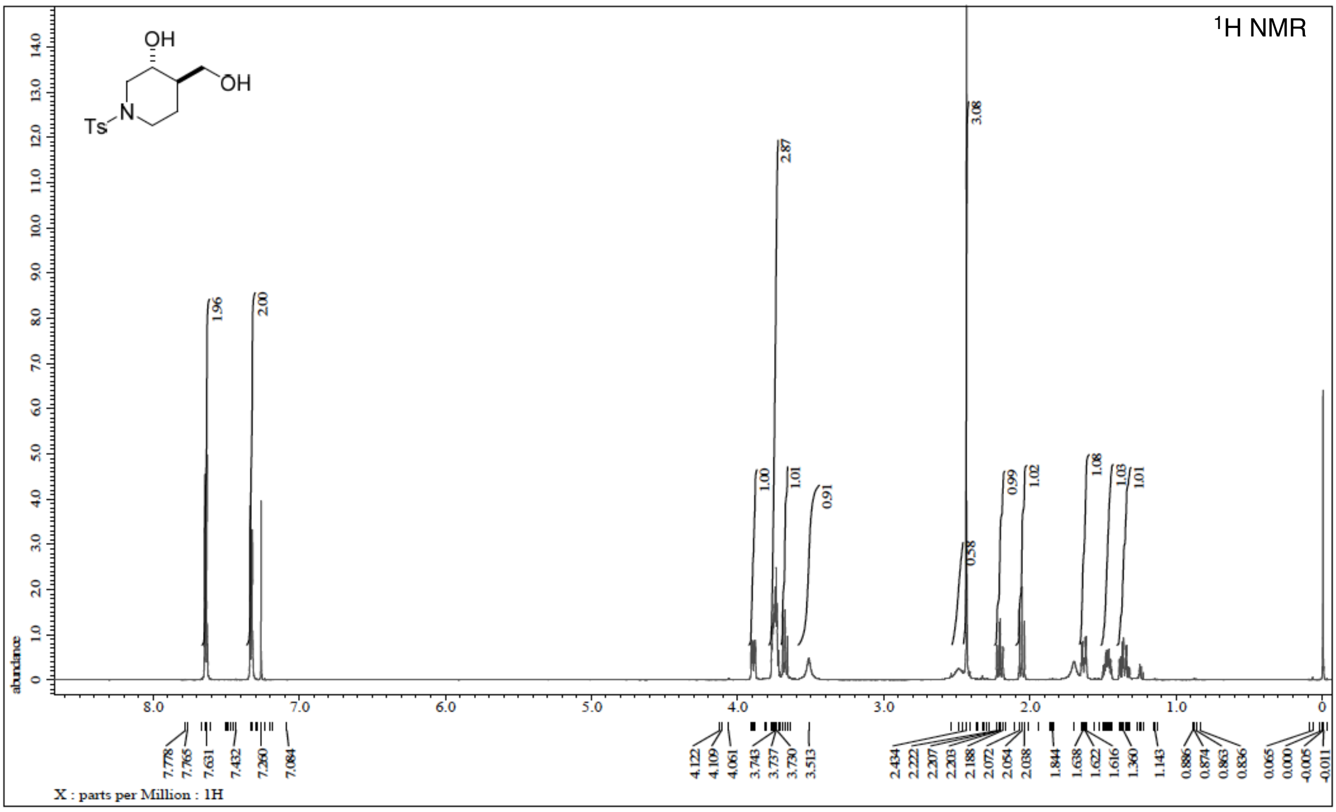

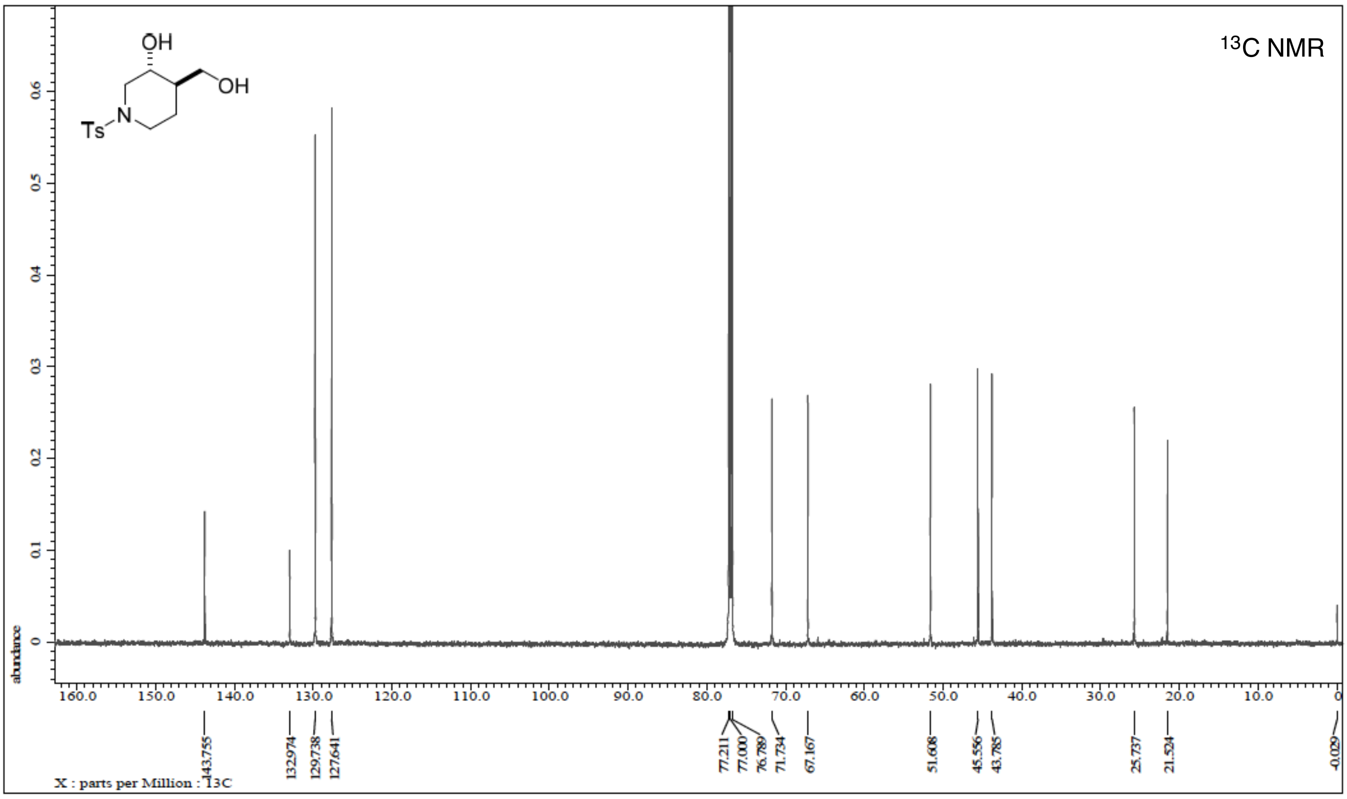

NMR spectra of **S17**

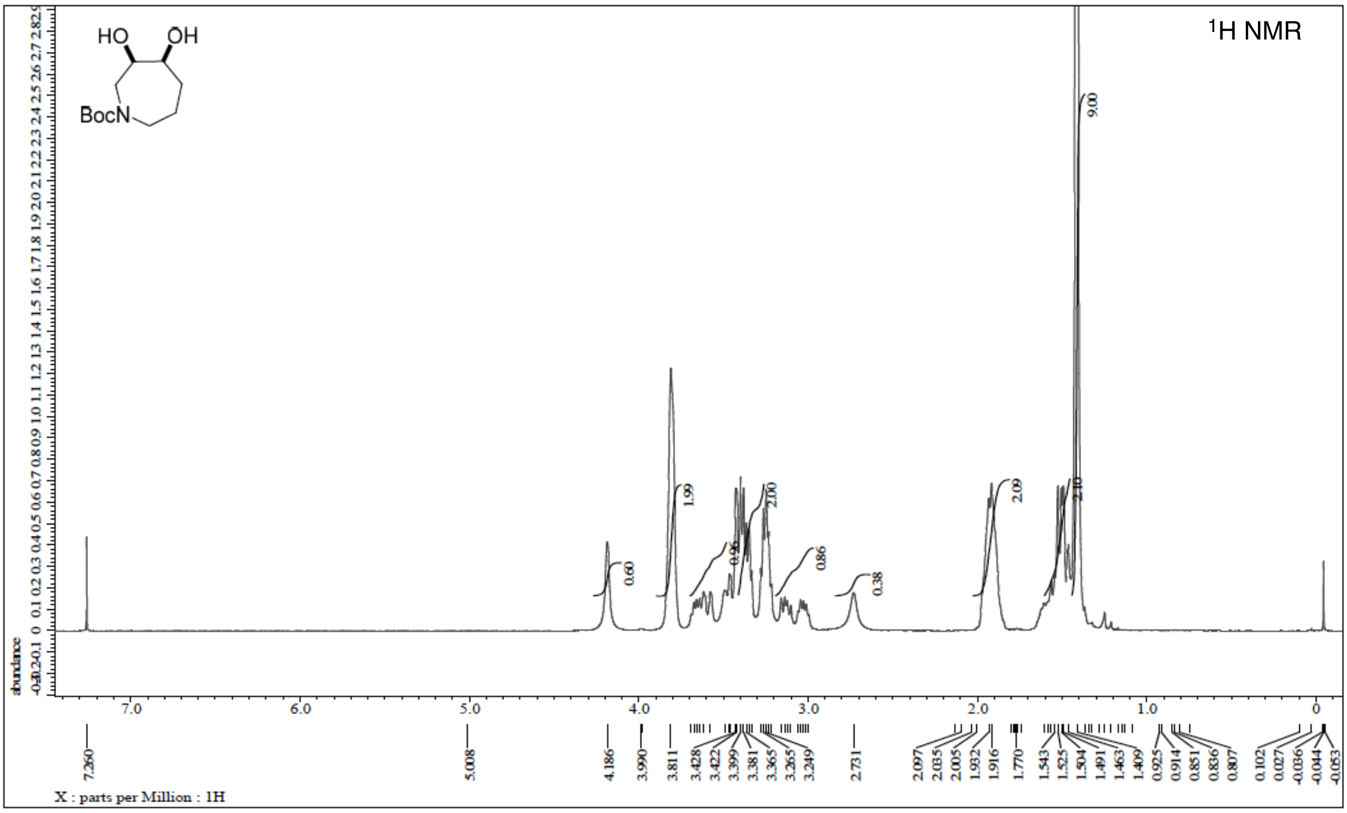

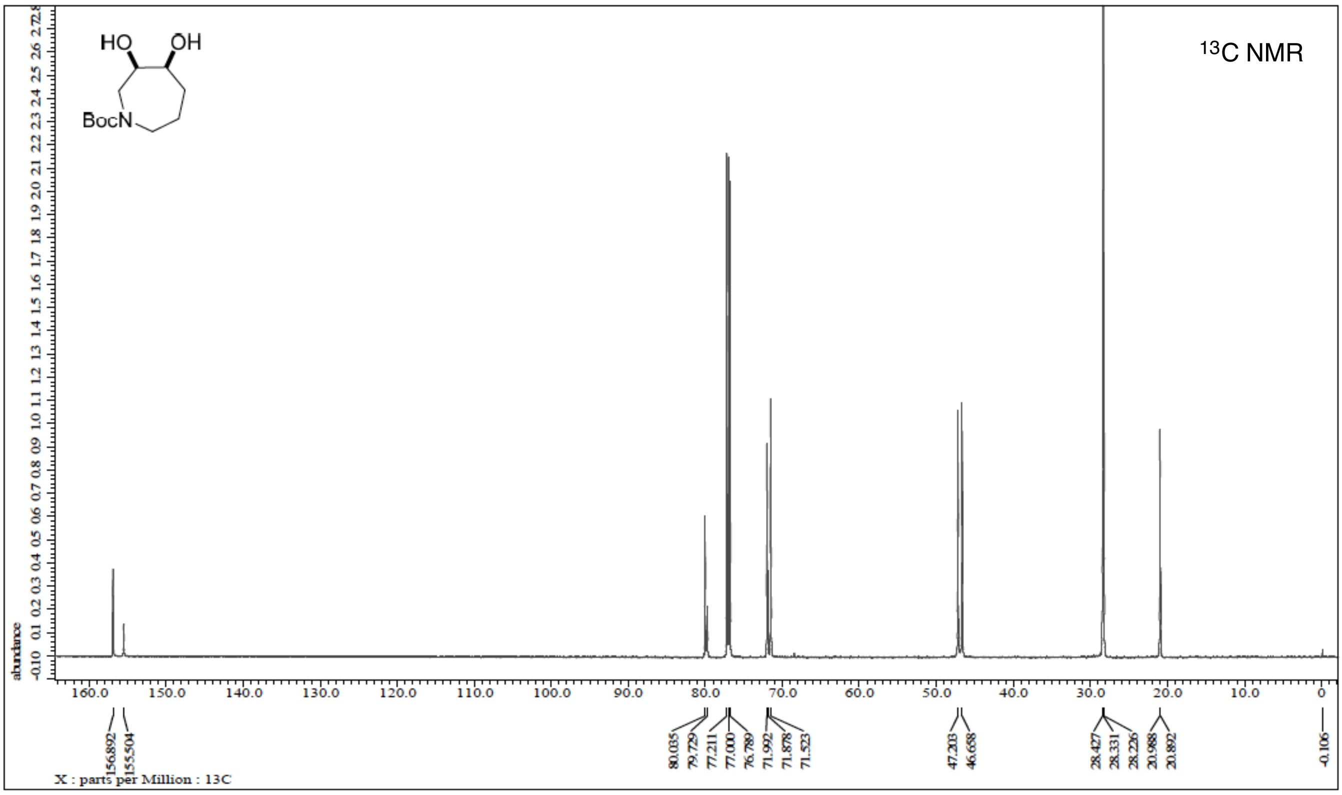

NMR spectra of **23**

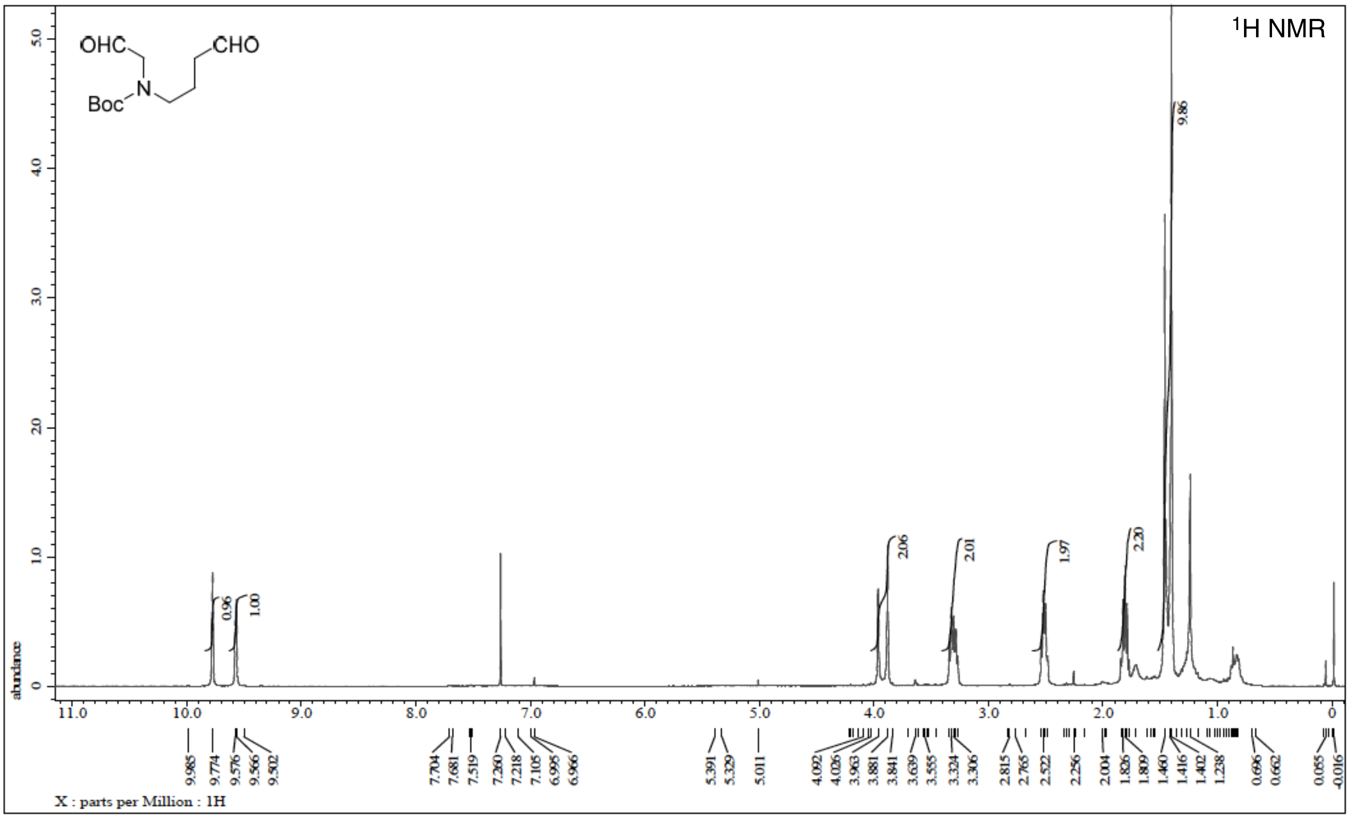

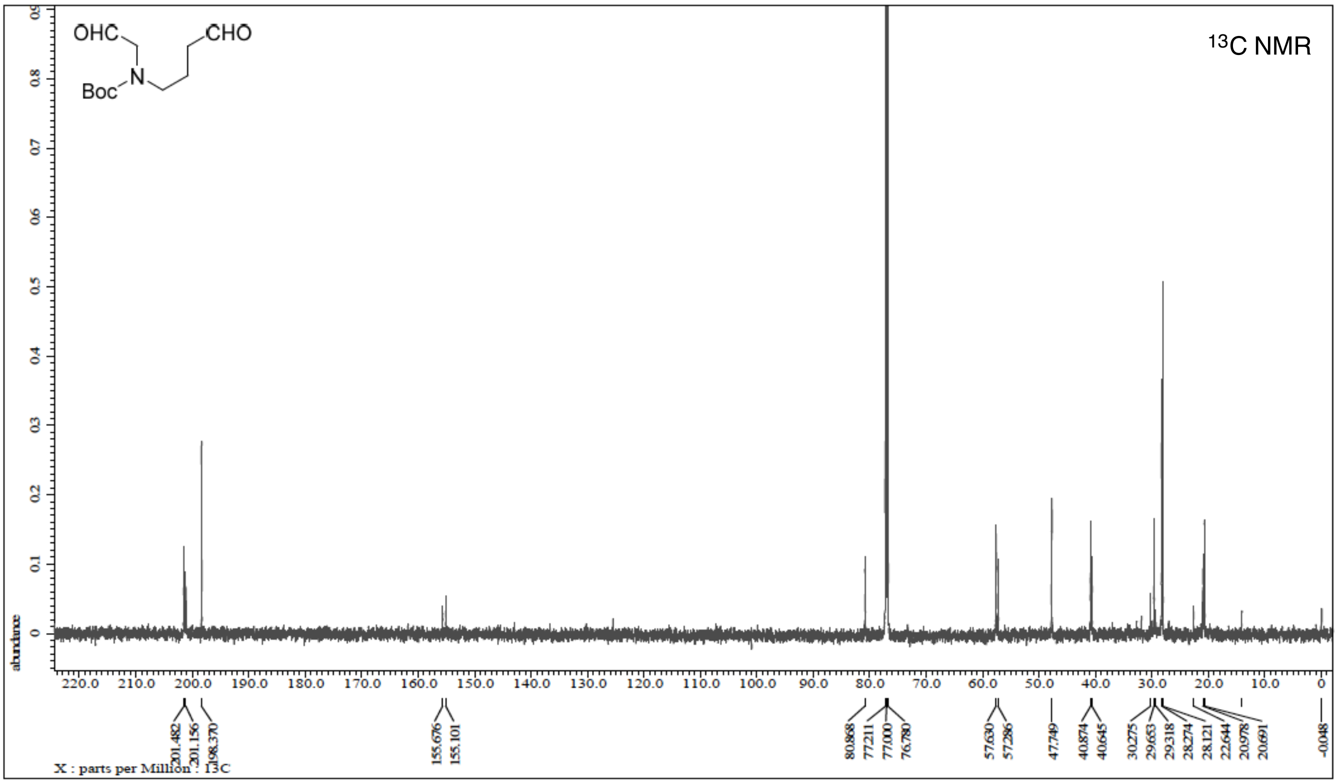

NMR spectra of *anti*-**26**

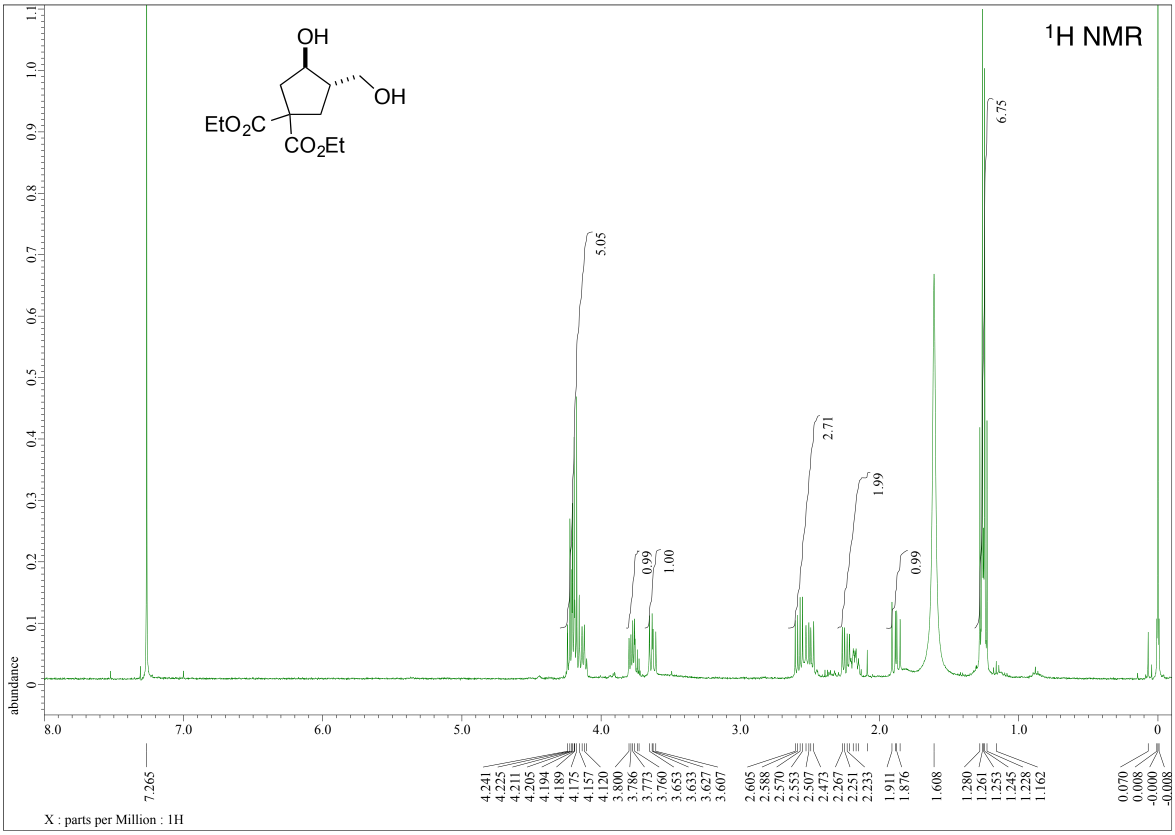

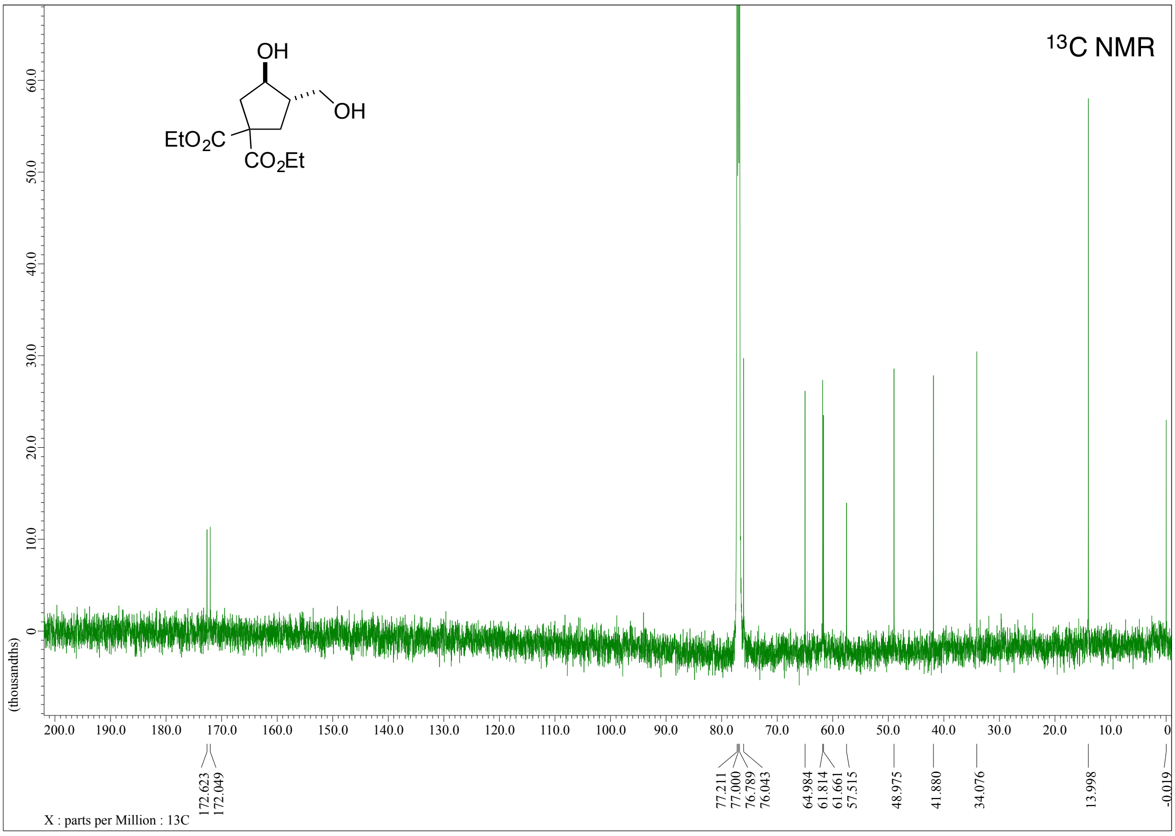

NMR spectra of *anti*-S**19**

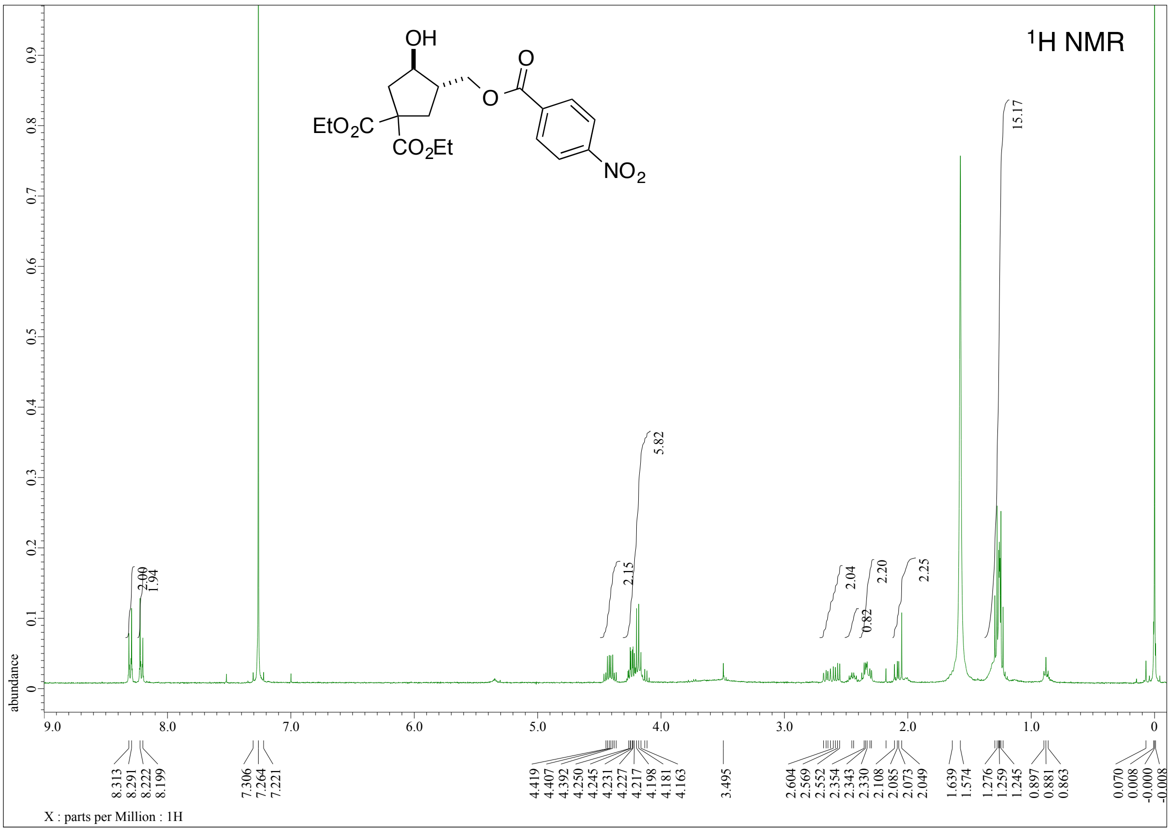

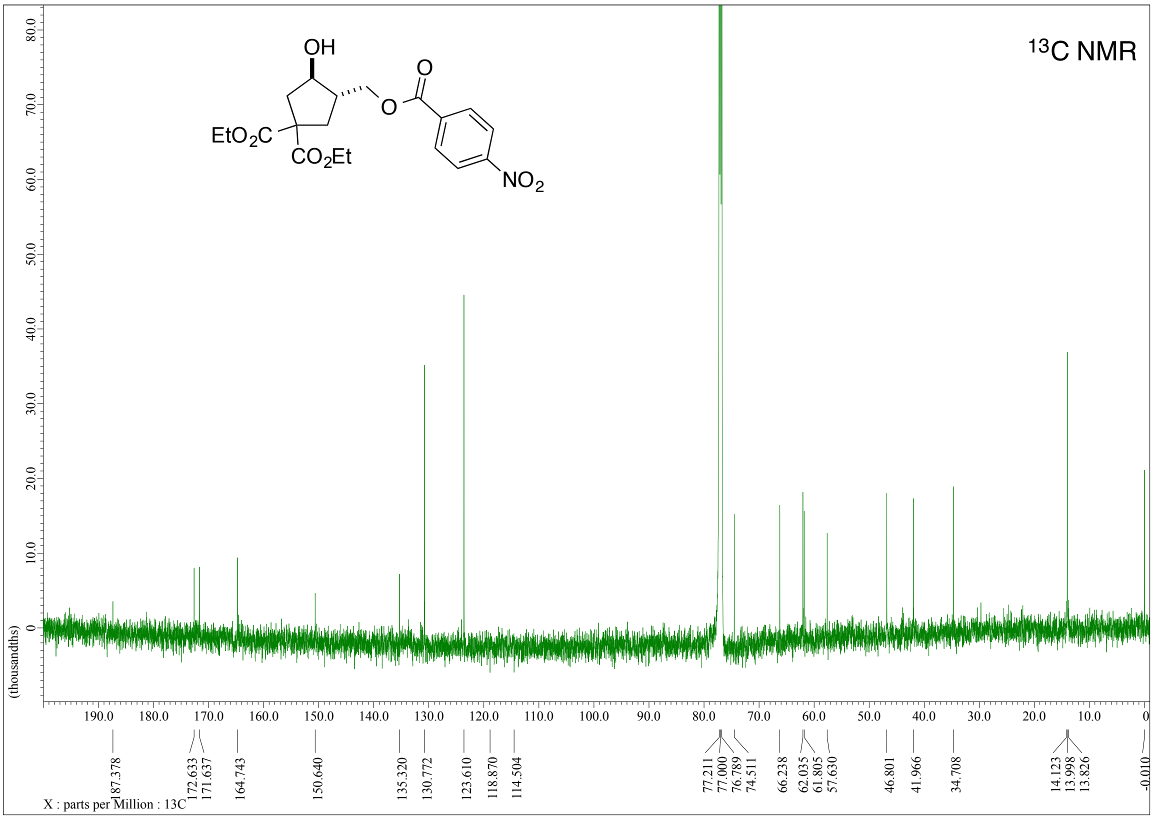

NMR spectra of **25**

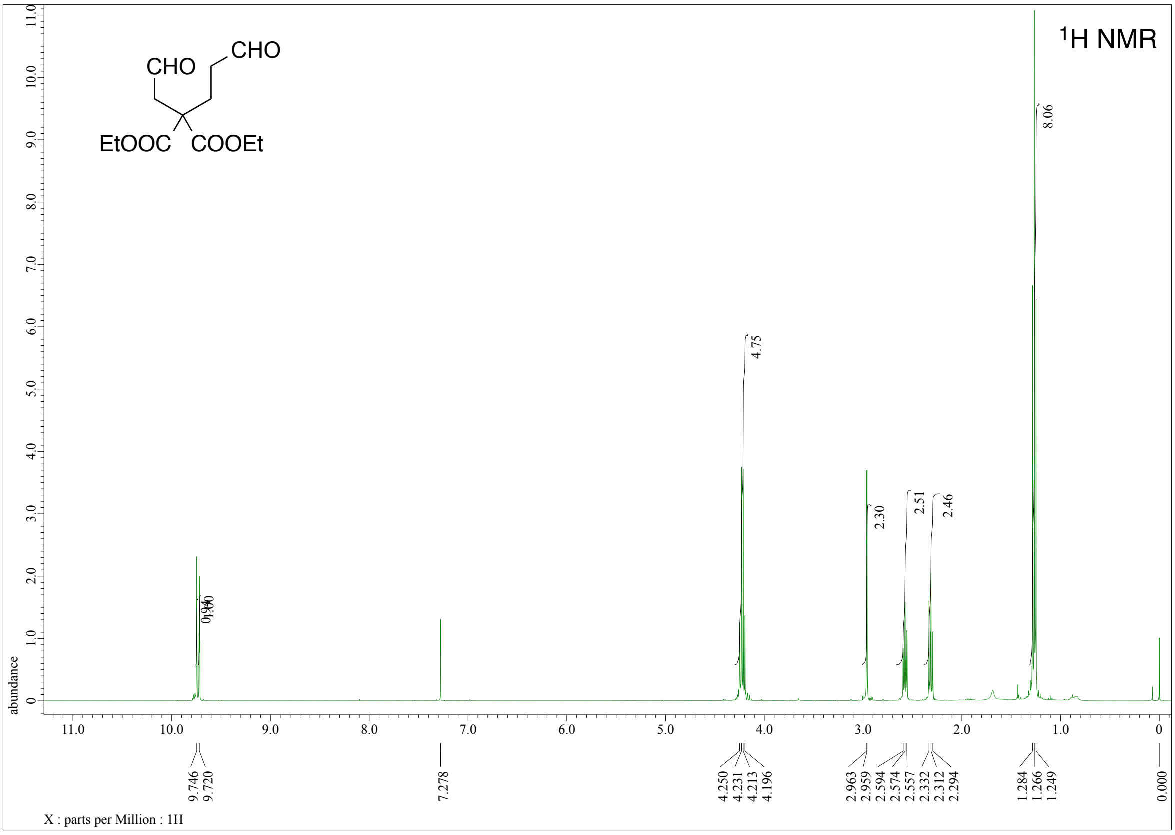

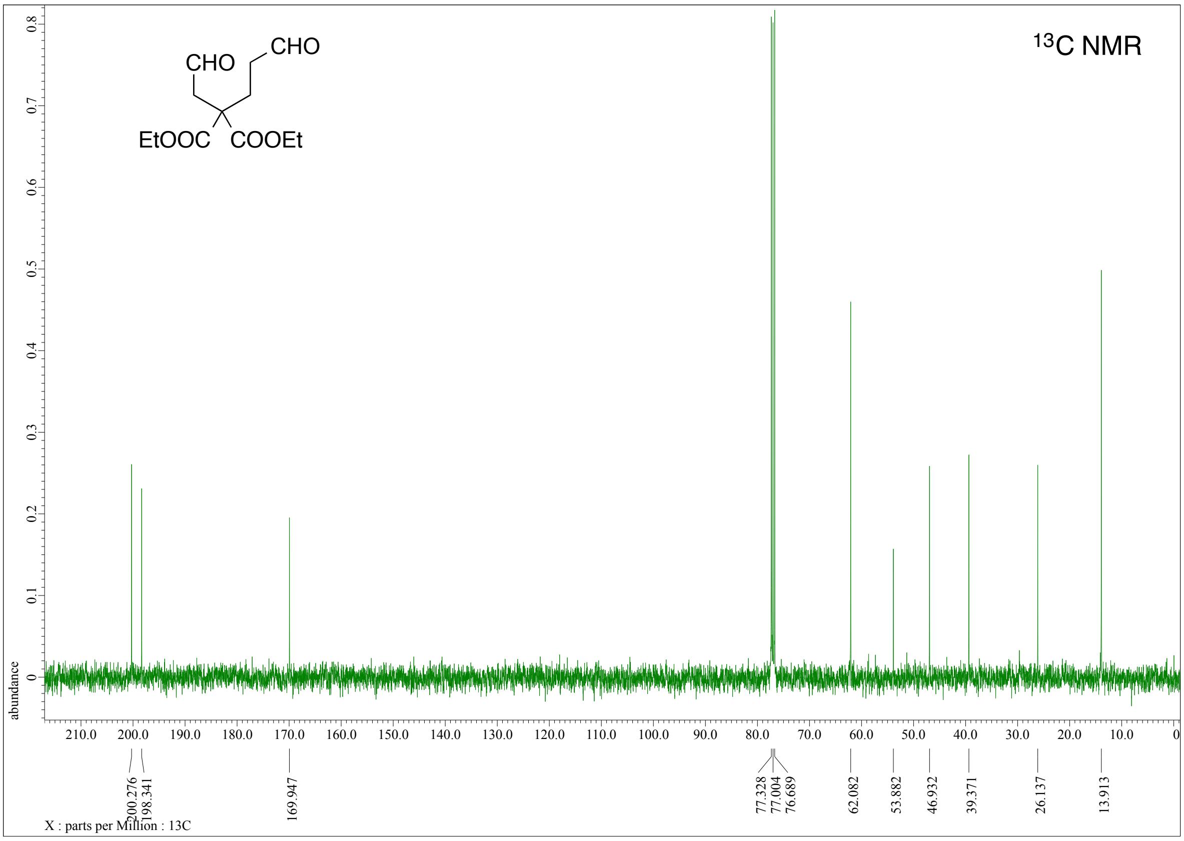

NMR spectra of (*dl*)-**27**

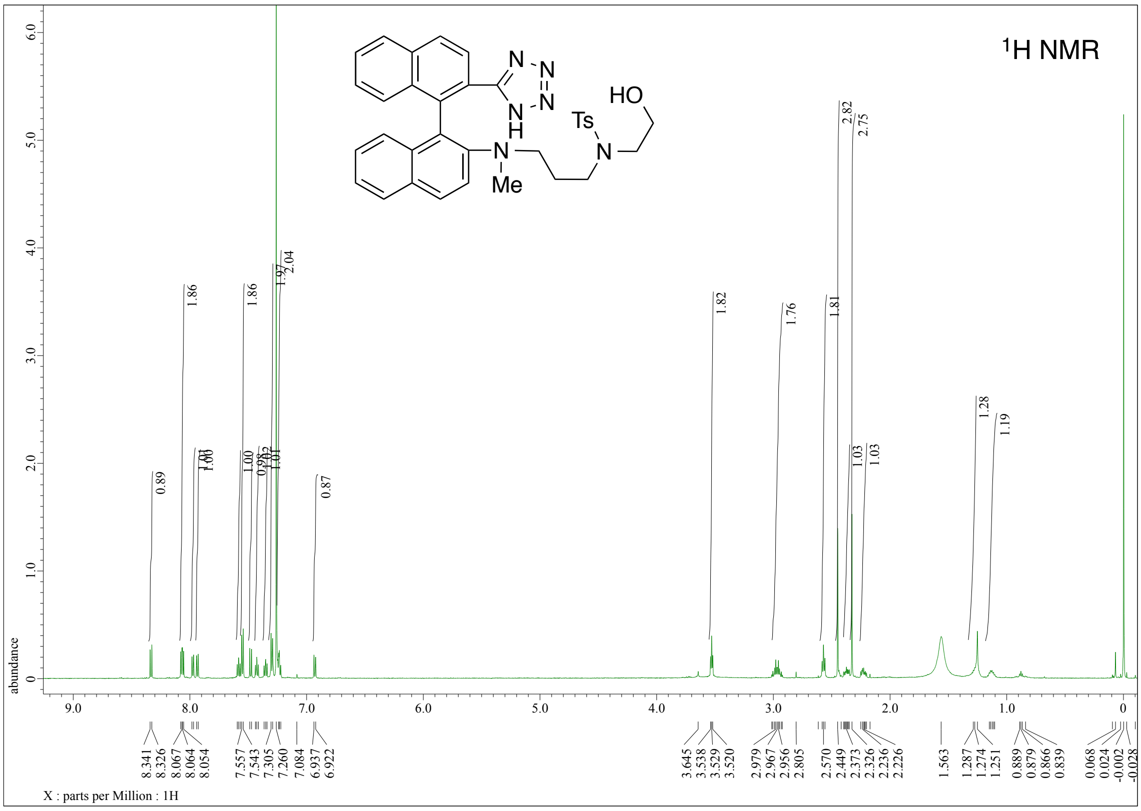

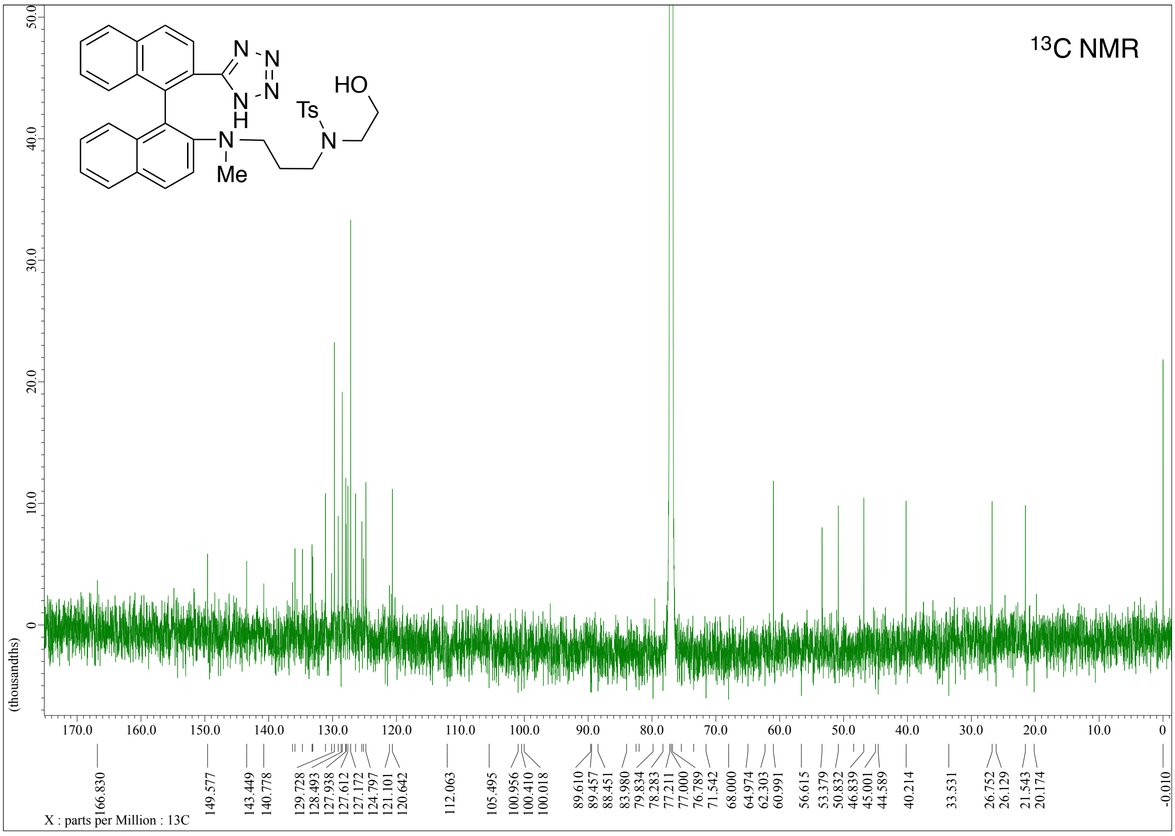

Key HMQC correlations for (*dl*)-**27**

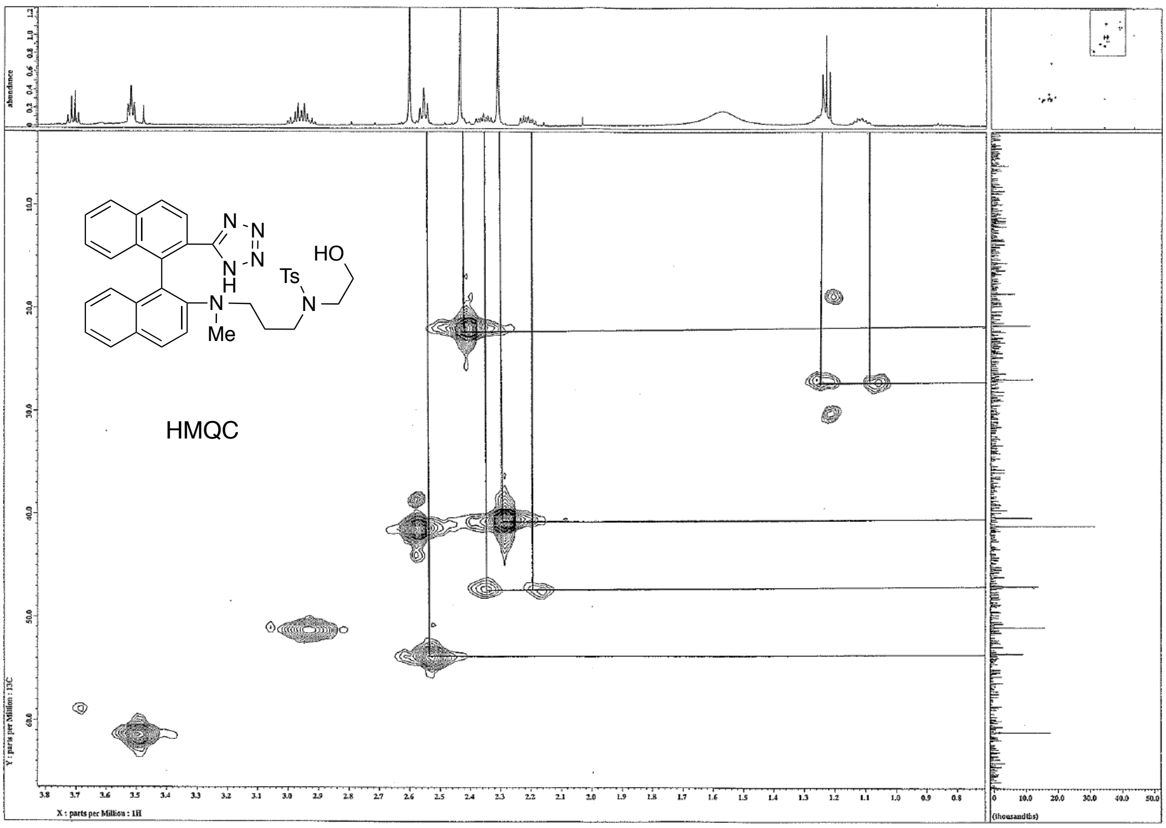

Key HMBC correlations for (*dl*)-**27**

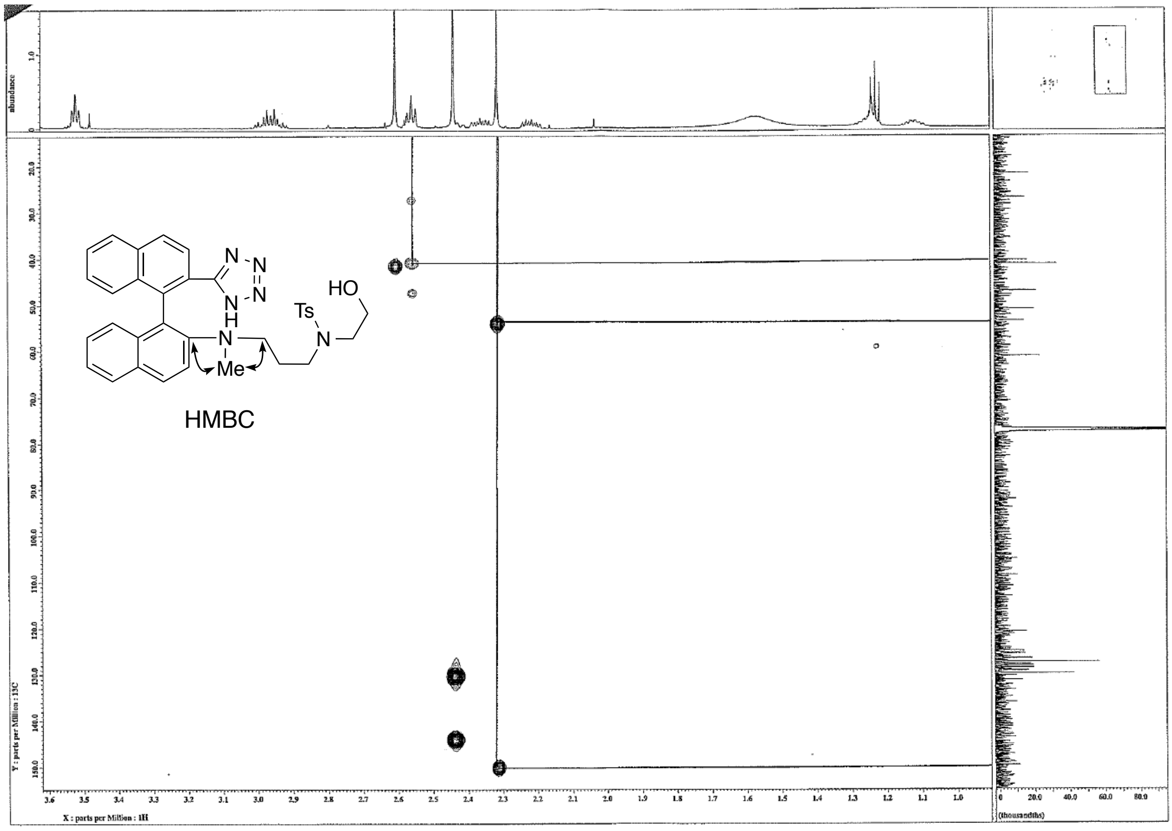

Supplement: Supplementary file 1 [file SC-007-C5SC04594K-s001.pdf]
